# Birth of a Regulatory Long Non-coding RNA/Gene, *linc-UR-UB*

**DOI:** 10.3389/fgene.2021.661425

**Published:** 2021-04-30

**Authors:** Nicholas Delihas

**Affiliations:** Department of Microbiology and Immunology, Renaissance School of Medicine, Stony Brook University, Stony Brook, NY, United States

**Keywords:** gene birth, lincRNA, USP18, evolution, gene structure, 3' UTR

## Abstract

The origin of genes has been a major topic of research for many years, albeit in some cases, it has been a difficult process to elucidate. Insightful is a recent publication that experimentally shows how one gene, *linc-UR-UB* was born. This gene is regulated in a complex manner in male germ cells during spermatogenesis and is believed to participate in the regulation of levels of the ubiquitin specific peptidase 18 (*USP18*) mRNA. The process of formation of *linc-UR-UB* appears relatively simple. It involves a transcription read through from an upstream gene to a downstream functional element, the USP18 3' UTR sequence. This small element also shares the same sequence as the 3' ends of the lincRNA *FAM247* family genes. In addition to *linc-UR-UB*, it is possible that other genes formed in a similar fashion that involves a genomic sequence read through to a functional element.

## Introduction

The recent paper by Sandra Pellegrini and co-workers in *Frontiers in Genetics* (Rubino et al., 2021) is multifaceted. The authors describe non-coding RNA components involved in the regulation of interferon and the JAK/STAT signaling pathway by the ubiquitin specific protease USP18, and they show binding of certain miRNAs to the *USP18* mRNA 3' UTR, which is proposed to regulate *USP18* expression. They also discovered a new long intergenic non-coding RNA (lincRNA) gene termed *linc-UR-B1* that may form part of a network that regulates *USP18* mRNA levels and act by a sponging process. Intriguing is the formation of *linc-UR-B1*, which the authors experimentally elucidated. The study by Rubino and co-workers on the *linc-UR-B1* transcript also touches on the complexity and definition of eukaryotic genes (Carninci and Hayashizaki, 2007; Portin and Wilkins, 2017), and here, we discuss aspects of read through transcripts that may form new genes.

## Structure and Formation of *linc-Ur-B1*

**Figure 1:**
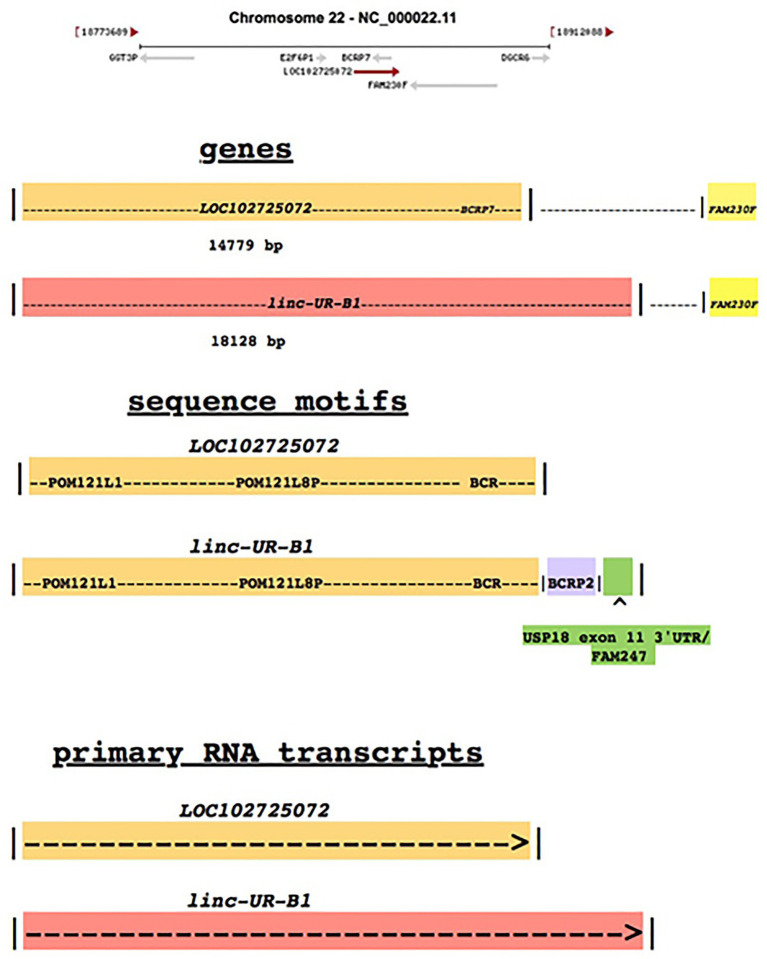
Comparison of *LOC102725072* and *linc-UR-B1*. Top diagram: Annotated genes that neighbor *LOC102725072* in human chr22 with chromosomal coordinates of the genomic segment shown. The schematic is from the NLM/NCBI website: https://www.ncbi.nlm.nih.gov/gene/?term=LOC102725072 (O’Leary et al., 2016). Bottom highlighted diagrams: The *BCRP7* gene is a counter transcript within the *LOC102725072* gene. *LOC102725072* consists of the sequence of the paralogs gene *POM121L8P* and the *POM121L1* (*LOC102724151*) sequence at its 5' end that is carried by *POM121L8P*; and in addition, a segment of the *BCRP2* gene that carries a portion of the 3' end of the *BCR* gene (Supplementary Figure S1). The *BCRP2* sequence extends from *BCRP7* to the 3' end of *LOC102725072* (and beyond) and is contiguous with the *BCRP7* sequence. The lower diagrams represent the *linc-UR-B1* gene and the signature gene/sequence motifs that *LOC102725072* and *linc-UR-B1* contain. Also shown are the bp lengths of the genes. The *BCRP2* gene sequence (of which 2,124 bp of the sequence resides beyond the 3' end of *LOC102725072*) forms part of *linc-UR-B*. In addition, 1,225 bp of a homologous sequence to FAM247 is also in *linc-UR-B1*, which contains the *USP18* exon 11 3' UTR sequence but also contains additional sequences of FAM247. *POM121L1* is the putative POM121 transmembrane nucleoporin like 1 protein gene *LOC10272415* but has not yet been characterized outside of computational methods. The diagrams are not drawn to scale.

*Linc-UR-B1* contains the upstream sequence of gene *LOC102725072* and uses the transcriptional start of *LOC102725072*. In the downstream genomic region of *LOC102725072*, a sequence homologous to the USP18 exon 11 3' UTR/3' end of the lincRNA gene family *FAM247A-D* is present and this element is incorporated into the *linc-UR-B1* sequence as a result of transcription read through (Figure 1). The terminal ends of *FAM247A*, *C*, and *D* carry the *USP18* mRNA exon 11 and 3' UTR sequence. The 3' ends of *USP18* and *FAM247* family genes are nearly identical; there is 99.8% identity between the *USP18* exon 11 3' UTR/3' end of *FAM247A*. Because of this high identity, the original source of the USP18 exon 11 3' UTR/3' end FAM247 sequence downstream of *LOC102725072* is uncertain. A segment of the *BCRP2* pseudogene sequence is also present in this downstream region (Figure 1, highlighted in light blue), but the function of this segment is unknown. Thus, *linc-UR-B1* is formed by read through transcription of the *LOC102725072* gene to include the *BCRP2* segment and the *USP18* exon 11 3' UTR/3' end of the *FAM247* sequence, which may be the functional key player. *Linc-UR-B1* encodes two transcript isoforms termed *TCONS_00029753* and *TCONS_00029754* (Rubino et al., 2021). *LOC102725072* by itself encodes RNA transcripts NR_135922 and NR_170942.1. As *linc-UR-B1* is regulated in a complex fashion in male germ cells, there are specific transcriptional regulatory elements that regulate *linc-UR-B1* expression during spermatogenesis.

Although *linc-UR-B1* and *LOC102725072* use the same transcriptional start site, we consider *linc-UR-B1* to be a separate gene as it is a read through transcript, carries additional sequences not included in either *LOC102725072* or its transcripts, and one added sequence provides a functional element, the USP18 3'UTR. Of interest, Ensembl/GENCODE provides a discussion on how they annotate read through transcripts as genes and the difficulties in this process.^1^

From BLAST searches performed by this author, ~83% of the *LOC102725072* gene sequence is found present in chimpanzee chr 22. Additionally, the *BCRP2* sequence is also present in the downstream region; importantly, however, neighbor sequences, the FAM247 sequence bearing the *USP18* terminal exon 11 3' UTR and the *FAM230F* lincRNA gene are not present (Supplementary Figure S2). Thus, most of the sequence that forms *LOC102725072* and the entire associated *BCRP2* downstream segment are in place in the primate ancestor, but the 3' end of *USP18*/FAM247 is not; thus, the functional *USP* exon11 3' UTR motif was added in the human genome, presumably to enable the formation and the function of *linc-UR-B1*.

The *FAM247A-D* lincRNA gene family is believed to have formed in humans by the process of gene duplication *via* chromosomal segmental duplications or low copy repeats in chromosome 22 (Delihas, 2020). These genes show ubiquitous RNA expression in somatic tissues but major expression in fat, brain, and testes (O’Leary et al., 2016).^2^ The functions of these genes are unknown. The *FAM247A* sequence has been used as a standard for sequence and evolutionary comparisons and termed FAM247 for practical purposes. Different sections of the FAM247 sequence are found to be components of diverse genes, which include two ancient protein genes, one of which is *USP18*.

*Linc-UR-B1* is an experimentally determined RNA gene that is formed by the simple addition of a functional element to the downstream region of an existing gene and a transcriptional read through to the functional element, the 3' UTR USP18/3' end FAM247 (Figure 1). It should be noted that there is an analogy between *linc-UR-B1* and a human neuronal transcript, PTENJ2 that encodes an altered *PTEN* protein (Lerch et al., 2012). This transcript is described by the authors as a diverse or non-conventional isoform of *PTEN* that has novel 5' and 3' UTRs. *PTEN* is a phosphatase and tensin homolog.

## Sequence Motifs in *linc-Ur-B1* Are Related to Those in Genomic Non-Coding Regions With Fam247 as the Consistent Element

**Figure 2:**
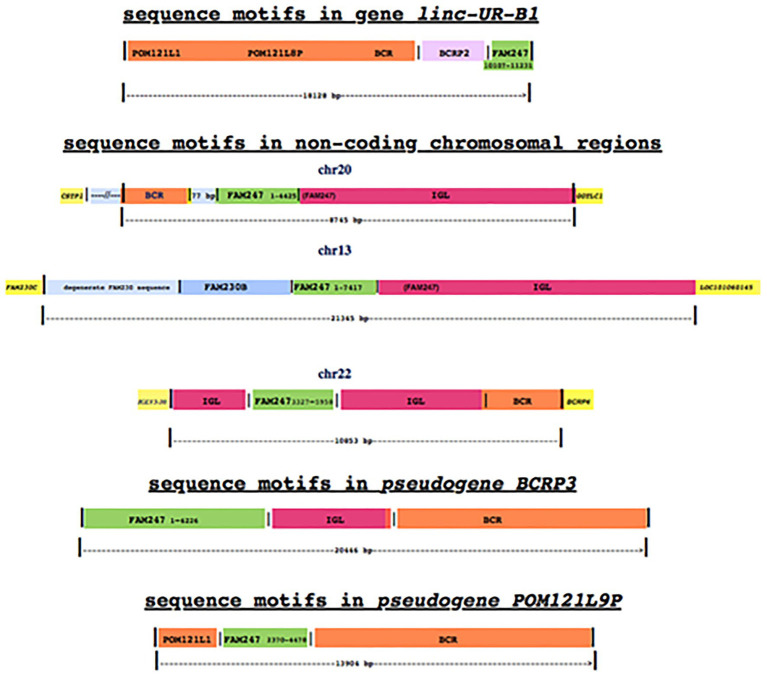
A diagrammatic comparison of non-coding chromosomal loci with *linc-UR-B1* and genes that display similar gene/sequence motifs. The numbers next to FAM247 are the bp positions of the FAM247 sequences that are present in each non-coding region and each gene; the segments of the FAM247 sequence that is present in the genes/non-coding regions differ in each example shown. In chr 20, 4,425 bp of FAM247 includes 3,305 bp present in and contiguous with the immunoglobulin lambda (IGL) sequence; in chr 13, the 7,442 bp shown includes 3,298 bp of FAM247 that are also in the IGL sequence. The genes highlighted in yellow flank the non-coding regions and represent guideposts. Supplementary Figures S3A–C provides an analysis of these regions. The *POM121L9P* and *BCRP3* gene structures are from Delihas (2020).

Three non-coding chromosomal loci have been detected by a BLAT sequence/gene search of the human genome (Madeira et al., 2019) by using a 2.87 kb sequence query from the ancestral primate *Philippine tarsier*, which consists of the chromosomal sequence between genes *GGT1* and *GGT5*. This sequence contains an ancestral homolog to the 5' end of FAM247. The region (between *GGT1* and *GGT5*) is of major evolutionary significance as it shows a very large genomic expansion in the Rhesus monkey, to 216.20 kb (Delihas, 2020). The three non-coding loci are in human chromosomes 20, 13, and 22 (Figure 2); in chr22, the non-coding region is part of the large immunoglobulin lambda (IGL) locus. These regions show no gene expression by RNA-seq analyses in somatic tissues according to RNA expression analyses^3^ but display sequence motifs that represent segments of genes, some of which are similar to those shown for *linc-UR-B1*. What is striking is the similarity in the “cast of characters” consisting of segments of various genes/sequences linked to different segments of FAM247, where FAM247 (highlighted in green) is the motif present in all examples (Figure 2; Supplementary Figures S3A–C). However, the individual segments of FAM247 differ, including the FAM247 sequence present in the two pseudogenes, *BCRP3* and *POM121L9P* (Figure 2, bottom); *linc-UR-B1* is the only example that carries the *USP18* exon 11 3' UTR/3' end of FAM247). Although there are similarities in sequence motifs between the *linc-UR-B1* gene, the three non-coding regions and the two pseudogenes, especially between *linc-UR-B1* and *POM121L9P* (Figure 2, bottom diagram), these non-coding chromosomal regions are not well-understood, e.g., whether or not the FAM247 sequence formed the basis for the addition of gene motifs to the non-coding loci. The regions do date back to the chimpanzee genome, and also to the Rhesus monkey where partial sequences and signatures are also present. Aside from FAM247, functions of other gene motifs in the *linc-UR-B1* gene are not known.

We do not know why gene/sequence signatures in non-coding regions have been maintained during primate evolution; however, one possibility is that they have been reserved for the birth of future genes. Human pseudogenes *BCRP3* and *POM121L9P* (Figure 2, bottom diagrams) as well as *LOC102725072* (Figure 1) may be candidates for genes that were formed in humans from sequences in lower primate non-coding chromosomal regions. More needs to be learned about how fragments of specific genes came together in non-coding regions, their possible functions, and how these phylogenetically conserved sequences might transform into viable genes.

## Conclusion

Although the evolutionary history of the formation of *linc-UR-B1* is incomplete, the work of Rubino et al. (2021) significantly advances our knowledge of gene birth by showing how *linc-UR-B1* was created in humans. And there is beauty in the simplicity of this process-insertion of a small functional unit close to the end of an existing gene, utilizing the existing transcriptional apparatus and setting in place specific regulatory mechanisms for expression of this new gene in specific cells. This basic process of gene formation may also have prevailed in the creation of other lincRNA genes, although, this remains to be determined. At least one other known transcript, PTENJ2 shows some analogous properties. The Rubino et al. (2021) study also adds to the multifaceted properties of 3'UTRs, properties that other investigators have previously described. For example, Mercer et al. (2011) and Kocabas et al. (2015) showed that many 3'UTR sequences are independently expressed in the absence of protein coding sequences and that their expression is regulated during development.

## Data Availability Statement

The original contributions presented in the study are included in the article/Supplementary Material, further inquiries can be directed to the corresponding author.

## Author Contributions

ND initiated the concept of the paper and wrote the paper.

### Conflict of Interest

The author declares that the research was conducted in the absence of any commercial or financial relationships that could be construed as a potential conflict of interest.

## Footnotes

^1^https://www.ensembl.info/2019/02/11/annotating-readthrough-transcription-in-ensembl/

^2^www.ncbi.nlm.nih.gov/gene/?term=Homo+sapiens+FAM247A

^3^www.ncbi.nlm.nih.gov/gene
