# Supplementary material for: Birth of a Regulatory Long Non-coding RNA/Gene, linc-UR-UB
Source: Front Genet. 2021 Apr 30;12:661425. doi: 10.3389/fgene.2021.661425 (PMC8120154; doi:10.3389/fgene.2021.661425)
Supplement: Supplementary file 1 [file Data_Sheet_1.PDF]

Birth of a regulatory long non-coding RNA gene, linc-UR-UB

Nicholas Delihas
Department of Microbiology and Immunology
Renaissance School of Medicine Stony Brook University
Stony Brook, New York, 11794-5222

Email: Nicholas.delihas@stonybook.edu

Figure Supplementary S1. **Nucleotide sequence alignment of genes *LOC102725072, linc-UR-B1, and BCRP2.***
"LOC102725072.start-FAM230F.start.18846286-18865042.NEW.ref" represents the sequence from the 5' start of *LOC102725072* to the 5' side of the *FAM230F* gene and includes the *linc-UR-B1* sequence but does not include *FAM230F*. The presence of *BCRP2* in *linc-UR-B1* that is not part of the *LOC102725072* sequence is highlighted in tan in the alignment below. The EMBL-EBI Clustal Omega alignment programs was used [Madeira, F, Park, YM, Lee, J, Buso, N, Gur, T, Madhusoodanan, N., et al (2019)]. The EMBL-EBI search and sequence analysis tools APIs in 2019. *Nucleic Acids Res.*47(W1):W636-W641. doi: 10.1093/nar/gkz26.

```
CLUSTAL O(1.2.4) multiple sequence alignment


BCRP2.21103016-21122286.NCBI.ref.revcompl                       ------------------------------------------------------------ 0
LOC102725072.has.POM121L1                                       gctcgcctctctccttccagtacctattgcatgaccccccacgtccgcctcccgccattg 60
LOC102725072.start-FAM230F.start.18846286-18865042.NEW.ref      gctcgcctctctccttccagtacctattgcatgaccccccacgtccgcctcccgccattg 60

BCRP2.21103016-21122286.NCBI.ref.revcompl                       ------------------------------------------------------------ 0
LOC102725072.has.POM121L1                                       ccagcaagcgcctcgcgcgggtacctggctgcgcttattaatccgttaagctcgctctgt 120
LOC102725072.start-FAM230F.start.18846286-18865042.NEW.ref      ccagcaagcgcctcgcgcgggtacctggctgcgcttattaatccgttaagctcgctctgt 120

BCRP2.21103016-21122286.NCBI.ref.revcompl                       ------------------------------------------------------------ 0
LOC102725072.has.POM121L1                                       cacgggcgccgtgatgtgctcacgcgcccgctccctcaggtttaaaaggcgcgttgcccg 180
LOC102725072.start-FAM230F.start.18846286-18865042.NEW.ref      cacgggcgccgtgatgtgctcacgcgcccgctccctcaggtttaaaaggcgcgttgcccg 180

BCRP2.21103016-21122286.NCBI.ref.revcompl                       ------------------------------------------------------------ 0
LOC102725072.has.POM121L1                                       gcaacagaagaaactgctggcttagccgttggccgagttggcggctggacgaggacgctc 240
LOC102725072.start-FAM230F.start.18846286-18865042.NEW.ref      gcaacagaagaaactgctggcttagccgttggccgagttggcggctggacgaggacgctc 240

BCRP2.21103016-21122286.NCBI.ref.revcompl                       ------------------------------------------------------------ 0
LOC102725072.has.POM121L1                                       agagcccagctctcgagagttcaagcaaccgacggttccccactgctcccaggagcggtt 300
LOC102725072.start-FAM230F.start.18846286-18865042.NEW.ref      agagcccagctctcgagagttcaagcaaccgacggttccccactgctcccaggagcggtt 300

BCRP2.21103016-21122286.NCBI.ref.revcompl                       ------------------------------------------------------------ 0
LOC102725072.has.POM121L1                                       acctgggcactctgtgcccctccttcctgttcgggcccaggccgaggacctgccagtagg 360
LOC102725072.start-FAM230F.start.18846286-18865042.NEW.ref      acctgggcactctgtgcccctccttcctgttcgggcccaggccgaggacctgccagtagg 360

BCRP2.21103016-21122286.NCBI.ref.revcompl                       ------------------------------------------------------------ 0
LOC102725072.has.POM121L1                                       gctcagttgcctggagcccgttcagcccatcccccagttcactttgcctgtgggatctcc 420
LOC102725072.start-FAM230F.start.18846286-18865042.NEW.ref      gctcagttgcctggagcccgttcagcccatcccccagttcactttgcctgtgggatctcc 420

BCRP2.21103016-21122286.NCBI.ref.revcompl                       ------------------------------------------------------------ 0
LOC102725072.has.POM121L1                                       ccgttgctcctgcccgtggactgagtggcaggccatcctacaagcacccggacacttgac 480
LOC102725072.start-FAM230F.start.18846286-18865042.NEW.ref      ccgttgctcctgcccgtggactgagtggcaggccatcctacaagcacccggacacttgac 480

BCRP2.21103016-21122286.NCBI.ref.revcompl                       ------------------------------------------------------------ 0
LOC102725072.has.POM121L1                                       atcagtggtgtcaagacaactctaagaaggttttccgtgatcctgcaagccctgccttcc 540
LOC102725072.start-FAM230F.start.18846286-18865042.NEW.ref      atcagtggtgtcaagacaactctaagaaggttttccgtgatcctgcaagccctgccttcc 540

BCRP2.21103016-21122286.NCBI.ref.revcompl                       ------------------------------------------------------------ 0
LOC102725072.has.POM121L1                                       ttcctgggatcctgccttcaatttgattgcacaggtaccacagcaagccagtgctgtgtg 600
LOC102725072.start-FAM230F.start.18846286-18865042.NEW.ref      ttcctgggatcctgccttcaatttgattgcacaggtaccacagcaagccagtgctgtgtg 600

BCRP2.21103016-21122286.NCBI.ref.revcompl                       ------------------------------------------------------------ 0
LOC102725072.has.POM121L1                                       ctccgagttccagggcgtcctccagctcagccactgcactgagaacatggactctctgtg 660
LOC102725072.start-FAM230F.start.18846286-18865042.NEW.ref      ctccgagttccagggcgtcctccagctcagccactgcactgagaacatggactctctgtg 660

BCRP2.21103016-21122286.NCBI.ref.revcompl                       ------------------------------------------------------------ 0
LOC102725072.has.POM121L1                                       gggcccaggagccgggagtcacccctttggggtccacaacacccggctgtccccagactt 720
LOC102725072.start-FAM230F.start.18846286-18865042.NEW.ref      gggcccaggagccgggagtcacccctttggggtccacaacacccggctgtccccagactt 720

BCRP2.21103016-21122286.NCBI.ref.revcompl                       ------------------------------------------------------------ 0
LOC102725072.has.POM121L1                                       gtgtccagggaagatagtgttgagggccctcaaggagagcggggcagggatgcctgagca 780
LOC102725072.start-FAM230F.start.18846286-18865042.NEW.ref      gtgtccagggaagatagtgttgagggccctcaaggagagcggggcagggatgcctgagca 780

BCRP2.21103016-21122286.NCBI.ref.revcompl                       ------------------------------------------------------------ 0
LOC102725072.has.POM121L1                                       ggacaaggaccctagagtccaagagaatcctggtgatcagagaagggtccccgaggtcac 840
LOC102725072.start-FAM230F.start.18846286-18865042.NEW.ref      ggacaaggaccctagagtccaagagaatcctggtgatcagagaagggtccccgaggtcac 840

BCRP2.21103016-21122286.NCBI.ref.revcompl                       ------------------------------------------------------------ 0
LOC102725072.has.POM121L1                                       cggggatgcaccgtctgcatttcggcccctgcgggacaatagaggcctctctccctttgt 900
LOC102725072.start-FAM230F.start.18846286-18865042.NEW.ref      cggggatgcaccgtctgcatttcggcccctgcgggacaatagaggcctctctccctttgt 900

BCRP2.21103016-21122286.NCBI.ref.revcompl                       ------------------------------------------------------------ 0
LOC102725072.has.POM121L1                                       gcccgggcccgggcctctgcagacagacctccatgcccagaggtcagaaatcagatataa 960
LOC102725072.start-FAM230F.start.18846286-18865042.NEW.ref      gcccgggcccgggcctctgcagacagacctccatgcccagaggtcagaaatcagatataa 960

BCRP2.21103016-21122286.NCBI.ref.revcompl                       ------------------------------------------------------------ 0
LOC102725072.has.POM121L1                                       ccagacatcccagacctcctggacgagctcctgcaccaaccgaaatgccatctccagctc 1020
LOC102725072.start-FAM230F.start.18846286-18865042.NEW.ref      ccagacatcccagacctcctggacgagctcctgcaccaaccgaaatgccatctccagctc 1020

BCRP2.21103016-21122286.NCBI.ref.revcompl                       ------------------------------------------------------------ 0
LOC102725072.has.POM121L1                                       ctacagctccacgggaggcttgccggggctaaagcggaggagggggccagcctcatccca 1080
LOC102725072.start-FAM230F.start.18846286-18865042.NEW.ref      ctacagctccacgggaggcttgccggggctaaagcggaggagggggccagcctcatccca 1080

BCRP2.21103016-21122286.NCBI.ref.revcompl                       ------------------------------------------------------------ 0
LOC102725072.has.POM121L1                                       ctgccagctgaccctcagttcctcaaagacagtgagtgaggacaggcctcaggctgtctc 1140
LOC102725072.start-FAM230F.start.18846286-18865042.NEW.ref      ctgccagctgaccctcagttcctcaaagacagtgagtgaggacaggcctcaggctgtctc 1140

BCRP2.21103016-21122286.NCBI.ref.revcompl                       ------------------------------------------------------------ 0
LOC102725072.has.POM121L1                                       ttcaggtcacacccagtgtgaaaaggcagcagatatagcaccagggcagacactcgccct 1200
LOC102725072.start-FAM230F.start.18846286-18865042.NEW.ref      ttcaggtcacacccagtgtgaaaaggcagcagatatagcaccagggcagacactcgccct 1200

BCRP2.21103016-21122286.NCBI.ref.revcompl                       ------------------------------------------------------------ 0
LOC102725072.has.POM121L1                                       caggaatgactcctccacatccgaggcctctaggcccagtacacacaagtttcccctgct 1260
LOC102725072.start-FAM230F.start.18846286-18865042.NEW.ref      caggaatgactcctccacatccgaggcctctaggcccagtacacacaagtttcccctgct 1260

BCRP2.21103016-21122286.NCBI.ref.revcompl                       ------------------------------------------------------------ 0
LOC102725072.has.POM121L1                                       gccacgcaggcgaggggagcctttgatgctgccacctcccttagagctggggtaccgggt 1320
LOC102725072.start-FAM230F.start.18846286-18865042.NEW.ref      gccacgcaggcgaggggagcctttgatgctgccacctcccttagagctggggtaccgggt 1320

BCRP2.21103016-21122286.NCBI.ref.revcompl                       ------------------------------------------------------------ 0
LOC102725072.has.POM121L1                                       cactgttgaagacctggaccgggagaaggaggcggcattccagcgcatcaacagtgcact 1380
LOC102725072.start-FAM230F.start.18846286-18865042.NEW.ref      cactgttgaagacctggaccgggagaaggaggcggcattccagcgcatcaacagtgcact 1380

BCRP2.21103016-21122286.NCBI.ref.revcompl                       ------------------------------------------------------------ 0
LOC102725072.has.POM121L1                                       gcaggttgaggacaaggccatctcggactgcagaccctcacgaccttcccacactttgtc 1440
LOC102725072.start-FAM230F.start.18846286-18865042.NEW.ref      gcaggttgaggacaaggccatctcggactgcagaccctcacgaccttcccacactttgtc 1440
```

```
BCRP2.21103016-21122286.NCBI.ref.revcompl                        ------------------------------------------------------------ 0
LOC102725072.has.POM121L1                                        ctcacttgcaacagggacttctggtctgcctgccatttctaaagcacccagtatggatgt 1500
LOC102725072.start-FAM230F.start.18846286-18865042.NEW.ref       ctcacttgcaacagggacttctggtctgcctgccatttctaaagcacccagtatggatgt 1500

BCRP2.21103016-21122286.NCBI.ref.revcompl                        ------------------------------------------------------------ 0
LOC102725072.has.POM121L1                                        acagcaggagacacacaagtcccaagactgcctgggcctactggccccttagcatctgc 1560
LOC102725072.start-FAM230F.start.18846286-18865042.NEW.ref       acagcaggagacacacaagtcccaagactgcctgggcctactggccccttagcatctgc 1560

BCRP2.21103016-21122286.NCBI.ref.revcompl                        ------------------------------------------------------------ 0
LOC102725072.has.POM121L1                                        tgcaggtgtcccctctacagctcccatgtctgggaagaagcacaaaccaccaggcccct 1620
LOC102725072.start-FAM230F.start.18846286-18865042.NEW.ref       tgcaggtgtcccctctacagctcccatgtctgggaagaagcacaaaccaccaggcccct 1620

BCRP2.21103016-21122286.NCBI.ref.revcompl                        ------------------------------------------------------------ 0
LOC102725072.has.POM121L1                                        gttctcctcctcagatccccttcctgccacctcttcccattcccaggactcagcccaggt 1680
LOC102725072.start-FAM230F.start.18846286-18865042.NEW.ref       gttctcctcctcagatccccttcctgccacctcttcccattcccaggactcagcccaggt 1680

BCRP2.21103016-21122286.NCBI.ref.revcompl                        ------------------------------------------------------------ 0
LOC102725072.has.POM121L1                                        cacctcgctgattcctgccccttcccagctgcaagcatggatgtgggcatgagaagaac 1740
LOC102725072.start-FAM230F.start.18846286-18865042.NEW.ref       cacctcgctgattcctgccccttcccagctgcaagcatggatgtgggcatgagaagaac 1740

BCRP2.21103016-21122286.NCBI.ref.revcompl                        ------------------------------------------------------------ 0
LOC102725072.has.POM121L1                                        aaggtgtggcacttctgctcctgcagctgccgcagcagcccctccccctccacattgaa 1800
LOC102725072.start-FAM230F.start.18846286-18865042.NEW.ref       aaggtgtggcacttctgctcctgcagctgccgcagcagcccctccccctccacattgaa 1800

BCRP2.21103016-21122286.NCBI.ref.revcompl                        ------------------------------------------------------------ 0
LOC102725072.has.POM121L1                                        ccccacgttggggtcactactggagtggatggaggcccttcacatttctgggcctcagcc 1860
LOC102725072.start-FAM230F.start.18846286-18865042.NEW.ref       ccccacgttggggtcactactggagtggatggaggcccttcacatttctgggcctcagcc 1860

BCRP2.21103016-21122286.NCBI.ref.revcompl                        ------------------------------------------------------------ 0
LOC102725072.has.POM121L1                                        acagctgcagcaggtgcccagaggtcagaaccagagatcccagacctcccggaccagctc 1920
LOC102725072.start-FAM230F.start.18846286-18865042.NEW.ref       acagctgcagcaggtgcccagaggtcagaaccagagatcccagacctcccggaccagctc 1920

BCRP2.21103016-21122286.NCBI.ref.revcompl                        ------------------------------------------------------------ 0
LOC102725072.has.POM121L1                                        gtgccccaaacgaaatgccatctcgagcccctaccgctctacgggaggcctcccggaacg 1980
LOC102725072.start-FAM230F.start.18846286-18865042.NEW.ref       gtgccccaaacgaaatgccatctcgagcccctaccgctctacgggaggcctcccggaacg 1980

BCRP2.21103016-21122286.NCBI.ref.revcompl                        ------------------------------------------------------------ 0
LOC102725072.has.POM121L1                                        aaagcggagaagggggccagcctcatcccactgccagctgaacctcagttcctgaaacac 2040
LOC102725072.start-FAM230F.start.18846286-18865042.NEW.ref       aaagcggagaagggggccagcctcatcccactgccagctgaacctcagttcctgaaacac 2040

BCRP2.21103016-21122286.NCBI.ref.revcompl                        ------------------------------------------------------------ 0
LOC102725072.has.POM121L1                                        agtgagtgaggacggacctcaggctgtctcttcgggtcacacccagtgtgaaaagatggc 2100
LOC102725072.start-FAM230F.start.18846286-18865042.NEW.ref       agtgagtgaggacggacctcaggctgtctcttcgggtcacacccagtgtgaaaagatggc 2100

BCRP2.21103016-21122286.NCBI.ref.revcompl                        ------------------------------------------------------------ 0
LOC102725072.has.POM121L1                                        agatacagcaccagggcagacactcgcccccaggggtggctcccccagatcccaggcctc 2160
LOC102725072.start-FAM230F.start.18846286-18865042.NEW.ref       agatacagcaccagggcagacactcgcccccaggggtggctcccccagatcccaggcctc 2160

BCRP2.21103016-21122286.NCBI.ref.revcompl                        ------------------------------------------------------------ 0
LOC102725072.has.POM121L1                                        taggccctgtagatgcaagtttcccctgctgccacgcaggcgaggggagcctttgatgct 2220
LOC102725072.start-FAM230F.start.18846286-18865042.NEW.ref       taggccctgtagatgcaagtttcccctgctgccacgcaggcgaggggagcctttgatgct 2220

BCRP2.21103016-21122286.NCBI.ref.revcompl                        ------------------------------------------------------------ 0
LOC102725072.has.POM121L1                                        gccacctcccttagagctggggtaccgggtcactgctgaagacctggaccaggagaagga 2280
LOC102725072.start-FAM230F.start.18846286-18865042.NEW.ref       gccacctcccttagagctggggtaccgggtcactgctgaagacctggaccaggagaagga 2280

BCRP2.21103016-21122286.NCBI.ref.revcompl                        ------------------------------------------------------------ 0
LOC102725072.has.POM121L1                                        ggcggctttccagcgcatcaagagtgcactgcaggttgaggacaaggccatttagtactg 2340
LOC102725072.start-FAM230F.start.18846286-18865042.NEW.ref       ggcggctttccagcgcatcaagagtgcactgcaggttgaggacaaggccatttagtactg 2340

BCRP2.21103016-21122286.NCBI.ref.revcompl                        ------------------------------------------------------------ 0
LOC102725072.has.POM121L1                                        cagaccctcacggccttcccacactttgtcctcacttgcaacagaggcttctggtctgcc 2400
LOC102725072.start-FAM230F.start.18846286-18865042.NEW.ref       cagaccctcacggccttcccacactttgtcctcacttgcaacagaggcttctggtctgcc 2400

BCRP2.21103016-21122286.NCBI.ref.revcompl                        ------------------------------------------------------------ 0
LOC102725072.has.POM121L1                                        tgccatttctaaagcacccagtatggatgcacagcaggagagacacaagtcccaagactg 2460
LOC102725072.start-FAM230F.start.18846286-18865042.NEW.ref       tgccatttctaaagcacccagtatggatgcacagcaggagagacacaagtcccaagactg 2460

BCRP2.21103016-21122286.NCBI.ref.revcompl                        ------------------------------------------------------------ 0
LOC102725072.has.POM121L1                                        cctgggcctagtggcccccagcatctgctgcacaggcctgtagtcccagctactcagga 2520
LOC102725072.start-FAM230F.start.18846286-18865042.NEW.ref       cctgggcctagtggcccccagcatctgctgcacaggcctgtagtcccagctactcagga 2520

BCRP2.21103016-21122286.NCBI.ref.revcompl                        ------------------------------------------------------------ 0
LOC102725072.has.POM121L1                                        ggctgaggcaggagaagggcataaacccgggaggcagagcttgcagtgagctgagatcgc 2580
LOC102725072.start-FAM230F.start.18846286-18865042.NEW.ref       ggctgaggcaggagaagggcataaacccgggaggcagagcttgcagtgagctgagatcgc 2580

BCRP2.21103016-21122286.NCBI.ref.revcompl                        ------------------------------------------------------------ 0
LOC102725072.has.POM121L1                                        gccactgcactccagcctgggtgacagagcgagactccgtctcaaaaaaaaagaaaaaga 2640
LOC102725072.start-FAM230F.start.18846286-18865042.NEW.ref       gccactgcactccagcctgggtgacagagcgagactccgtctcaaaaaaaaagaaaaaga 2640

BCRP2.21103016-21122286.NCBI.ref.revcompl                        ------------------------------------------------------------ 0
LOC102725072.has.POM121L1                                        aaaaaaagttattgtgacatttctgtatgaaatcagccttcactacatggataggaccag 2700
LOC102725072.start-FAM230F.start.18846286-18865042.NEW.ref       aaaaaaagttattgtgacatttctgtatgaaatcagccttcactacatggataggaccag 2700

BCRP2.21103016-21122286.NCBI.ref.revcompl                        ------------------------------------------------------------ 0
LOC102725072.has.POM121L1                                        cacgcttctgcggcacaactctgcaatcatactacattttttttttgtattttttttat 2760
LOC102725072.start-FAM230F.start.18846286-18865042.NEW.ref       cacgcttctgcggcacaactctgcaatcatactacattttttttttgtattttttttat 2760

BCRP2.21103016-21122286.NCBI.ref.revcompl                        ------------------------------------------------------------ 0
LOC102725072.has.POM121L1                                        tccttttgagacagagtctcactctgtcacccaggctgaagtgcagccgagatctcggct 2820
LOC102725072.start-FAM230F.start.18846286-18865042.NEW.ref       tccttttgagacagagtctcactctgtcacccaggctgaagtgcagccgagatctcggct 2820

BCRP2.21103016-21122286.NCBI.ref.revcompl                        ------------------------------------------------------------ 0
LOC102725072.has.POM121L1                                        cactgcaacctccacctcctgggttcaagcaattctcctgtctcagcctcccaagtagct 2880
LOC102725072.start-FAM230F.start.18846286-18865042.NEW.ref       cactgcaacctccacctcctgggttcaagcaattctcctgtctcagcctcccaagtagct 2880

BCRP2.21103016-21122286.NCBI.ref.revcompl                        ------------------------------------------------------------ 0
LOC102725072.has.POM121L1                                        gggactacaggcacacgtcaaaaggcctggctaattttgtatttttagtagagatggag 2940
LOC102725072.start-FAM230F.start.18846286-18865042.NEW.ref       gggactacaggcacacgtcaaaaggcctggctaattttgtatttttagtagagatggag 2940

BCRP2.21103016-21122286.NCBI.ref.revcompl                        ------------------------------------------------------------ 0
LOC102725072.has.POM121L1                                        ttttgccatattggtcaggctggtctcgaactcctgacctcaggtgatctacctgtctta 3000
LOC102725072.start-FAM230F.start.18846286-18865042.NEW.ref       ttttgccatattggtcaggctggtctcgaactcctgacctcaggtgatctacctgtctta 3000

BCRP2.21103016-21122286.NCBI.ref.revcompl                        ------------------------------------------------------------ 0
LOC102725072.has.POM121L1                                        gcctcccgaagtgctaggattacaggtgtatgtttatttatttatttaagatggaatctt 3060
LOC102725072.start-FAM230F.start.18846286-18865042.NEW.ref       gcctcccgaagtgctaggattacaggtgtatgtttatttatttatttaagatggaatctt 3060

BCRP2.21103016-21122286.NCBI.ref.revcompl                        ------------------------------------------------------------ 0
LOC102725072.has.POM121L1                                        gctctgtatttattaatttatttagttgagatggagtcttgctccatcacccaggctagg 3120
LOC102725072.start-FAM230F.start.18846286-18865042.NEW.ref       gctctgtatttattaatttatttagttgagatggagtcttgctccatcacccaggctagg 3120

BCRP2.21103016-21122286.NCBI.ref.revcompl                        ------------------------------------------------------------ 0
LOC102725072.has.POM121L1                                        gtgcagtggtgcaatctcggctcactgcaacctctgacttccagtttcaagcgattctcc 3180
LOC102725072.start-FAM230F.start.18846286-18865042.NEW.ref       gtgcagtggtgcaatctcggctcactgcaacctctgacttccagtttcaagcgattctcc 3180
```

```
BCRP2.21103016-21122286.NCBI.ref.revcompl                      ------------------------------------------------------------ 0
LOC102725072.has.POM121L1                                      tgcctcagtgtcccaagtagctgggattacaggtgcctgccaccacagctggctaatttt 3240
LOC102725072.start-FAM230F.start.18846286-18865042.NEW.ref     tgcctcagtgtcccaagtagctgggattacaggtgcctgccaccacagctggctaatttt 3240

BCRP2.21103016-21122286.NCBI.ref.revcompl                      ------------------------------------------------------------ 0
LOC102725072.has.POM121L1                                      tgtatttttagtagagacagtgtttcaccatcttggccaggctggtctcgggctcctgac 3300
LOC102725072.start-FAM230F.start.18846286-18865042.NEW.ref     tgtatttttagtagagacagtgtttcaccatcttggccaggctggtctcgggctcctgac 3300

BCRP2.21103016-21122286.NCBI.ref.revcompl                      ------------------------------------------------------------ 0
LOC102725072.has.POM121L1                                      ctcatgaaccacctgcctcagcctcccaaagtgttgggattacaggcctaaggcaccatg 3360
LOC102725072.start-FAM230F.start.18846286-18865042.NEW.ref     ctcatgaaccacctgcctcagcctcccaaagtgttgggattacaggcctaaggcaccatg 3360

BCRP2.21103016-21122286.NCBI.ref.revcompl                      ------------------------------------------------------------ 0
LOC102725072.has.POM121L1                                      ctcggccatatttatttaattatttagagacaaagtcttgctctgtcacccaggctggag 3420
LOC102725072.start-FAM230F.start.18846286-18865042.NEW.ref     ctcggccatatttatttaattatttagagacaaagtcttgctctgtcacccaggctggag 3420

BCRP2.21103016-21122286.NCBI.ref.revcompl                      ------------------------------------------------------------ 0
LOC102725072.has.POM121L1                                      tgcagtggcgccatctcagcttactgcagcctccgtctctgaggtttaagcgattctcat 3480
LOC102725072.start-FAM230F.start.18846286-18865042.NEW.ref     tgcagtggcgccatctcagcttactgcagcctccgtctctgaggtttaagcgattctcat 3480

BCRP2.21103016-21122286.NCBI.ref.revcompl                      ------------------------------------------------------------ 0
LOC102725072.has.POM121L1                                      gcctcagcctcctgagtaactgggactacaggtactcaccaccatgcaggatattttttt 3540
LOC102725072.start-FAM230F.start.18846286-18865042.NEW.ref     gcctcagcctcctgagtaactgggactacaggtactcaccaccatgcaggatattttttt 3540

BCRP2.21103016-21122286.NCBI.ref.revcompl                      ------------------------------------------------------------ 0
LOC102725072.has.POM121L1                                      tctattgttttatagagacacggtttcaccatattggccaggctggtctcgaactcctga 3600
LOC102725072.start-FAM230F.start.18846286-18865042.NEW.ref     tctattgttttatagagacacggtttcaccatattggccaggctggtctcgaactcctga 3600

BCRP2.21103016-21122286.NCBI.ref.revcompl                      ------------------------------------------------------------ 0
LOC102725072.has.POM121L1                                      ccttaggtgatctgacagcctcgtcctctcaaagcactgggattacaggcatgagccgcc 3660
LOC102725072.start-FAM230F.start.18846286-18865042.NEW.ref     ccttaggtgatctgacagcctcgtcctctcaaagcactgggattacaggcatgagccgcc 3660

BCRP2.21103016-21122286.NCBI.ref.revcompl                      ------------------------------------------------------------ 0
LOC102725072.has.POM121L1                                      aagcccggcctctcactacatttaagtgacaccatggctcatgcctgtaatcctagcact 3720
LOC102725072.start-FAM230F.start.18846286-18865042.NEW.ref     aagcccggcctctcactacatttaagtgacaccatggctcatgcctgtaatcctagcact 3720

BCRP2.21103016-21122286.NCBI.ref.revcompl                      ------------------------------------------------------------ 0
LOC102725072.has.POM121L1                                      ttgggaggccaaggcaggtggatcacctgatgtcaggagttcgaaacgagcctggccaac 3780
LOC102725072.start-FAM230F.start.18846286-18865042.NEW.ref     ttgggaggccaaggcaggtggatcacctgatgtcaggagttcgaaacgagcctggccaac 3780

BCRP2.21103016-21122286.NCBI.ref.revcompl                      ------------------------------------------------------------ 0
LOC102725072.has.POM121L1                                      atggggaaaccccgtctctagtaaaaatacaaaaattagtcaggtgtggtggtacaagcc 3840
LOC102725072.start-FAM230F.start.18846286-18865042.NEW.ref     atggggaaaccccgtctctagtaaaaatacaaaaattagtcaggtgtggtggtacaagcc 3840

BCRP2.21103016-21122286.NCBI.ref.revcompl                      ------------------------------------------------------------ 0
LOC102725072.has.POM121L1                                      tgtaggcccagctacttggaagactgaggcaggagaatcactttaagcgggaggcagagg 3900
LOC102725072.start-FAM230F.start.18846286-18865042.NEW.ref     tgtaggcccagctacttggaagactgaggcaggagaatcactttaagcgggaggcagagg 3900

BCRP2.21103016-21122286.NCBI.ref.revcompl                      ------------------------------------------------------------ 0
LOC102725072.has.POM121L1                                      ttgcagtgagccaatctcaaaaaagaaagaaaaaaaaaaaagaaaaacatatgatgctggg 3960
LOC102725072.start-FAM230F.start.18846286-18865042.NEW.ref     ttgcagtgagccaatctcaaaaaagaaagaaaaaaaaaaaagaaaaacatatgatgctggg 3960

BCRP2.21103016-21122286.NCBI.ref.revcompl                      ------------------------------------------------------------ 0
LOC102725072.has.POM121L1                                      gcatctcggcctcaatacctgcatgagcacagtcacgtccaggccagggctgctggtcga 4020
LOC102725072.start-FAM230F.start.18846286-18865042.NEW.ref     gcatctcggcctcaatacctgcatgagcacagtcacgtccaggccagggctgctggtcga 4020

BCRP2.21103016-21122286.NCBI.ref.revcompl                      ------------------------------------------------------------ 0
LOC102725072.has.POM121L1                                      ggtccggccccatctcttccagcagaaagggagtaagcttgcagggaggctgggggacaa 4080
LOC102725072.start-FAM230F.start.18846286-18865042.NEW.ref     ggtccggccccatctcttccagcagaaagggagtaagcttgcagggaggctgggggacaa 4080

BCRP2.21103016-21122286.NCBI.ref.revcompl                      ------------------------------------------------------------ 0
LOC102725072.has.POM121L1                                      gatcccaggatctcagcctctgctcatggatcagctctgagaccccgagtgagctggggg 4140
LOC102725072.start-FAM230F.start.18846286-18865042.NEW.ref     gatcccaggatctcagcctctgctcatggatcagctctgagaccccgagtgagctggggg 4140

BCRP2.21103016-21122286.NCBI.ref.revcompl                      ------------------------------------------------------------ 0
LOC102725072.has.POM121L1                                      tgctctgtgcgcattggtttccccagctgtcaagtaaagggattggatgaggaagtcttg 4200
LOC102725072.start-FAM230F.start.18846286-18865042.NEW.ref     tgctctgtgcgcattggtttccccagctgtcaagtaaagggattggatgaggaagtcttg 4200

BCRP2.21103016-21122286.NCBI.ref.revcompl                      ------------------------------------------------------------ 0
LOC102725072.has.POM121L1                                      tcaaggtggaatgatctcagatttggggcagcagtgaatgatcccgctccctgggcccat 4260
LOC102725072.start-FAM230F.start.18846286-18865042.NEW.ref     tcaaggtggaatgatctcagatttggggcagcagtgaatgatcccgctccctgggcccat 4260

BCRP2.21103016-21122286.NCBI.ref.revcompl                      ------------------------------------------------------------ 0
LOC102725072.has.POM121L1                                      gccagtggcctggcctcggctcaacacagccccaacactctggaatggggatgagggggc 4320
LOC102725072.start-FAM230F.start.18846286-18865042.NEW.ref     gccagtggcctggcctcggctcaacacagccccaacactctggaatggggatgagggggc 4320

BCRP2.21103016-21122286.NCBI.ref.revcompl                      ------------------------------------------------------------ 0
LOC102725072.has.POM121L1                                      agtcagctcttgctcctagtaagagagatgcaacagggctctgtggctgagctgggtgcc 4380
LOC102725072.start-FAM230F.start.18846286-18865042.NEW.ref     agtcagctcttgctcctagtaagagagatgcaacagggctctgtggctgagctgggtgcc 4380

BCRP2.21103016-21122286.NCBI.ref.revcompl                      ------------------------------------------------------------ 0
LOC102725072.has.POM121L1                                      ttgcctcacacctgtaatcccaacctttgagaggccaaggcaggaggattgctcgaggcc 4440
LOC102725072.start-FAM230F.start.18846286-18865042.NEW.ref     ttgcctcacacctgtaatcccaacctttgagaggccaaggcaggaggattgctcgaggcc 4440

BCRP2.21103016-21122286.NCBI.ref.revcompl                      ------------------------------------------------------------ 0
LOC102725072.has.POM121L1                                      gggaattttgagaatagcctggacaacatagccagaccccatgtctacaaaataataata 4500
LOC102725072.start-FAM230F.start.18846286-18865042.NEW.ref     gggaattttgagaatagcctggacaacatagccagaccccatgtctacaaaataataata 4500

BCRP2.21103016-21122286.NCBI.ref.revcompl                      ------------------------------------------------------------ 0
LOC102725072.has.POM121L1                                      aaacacacagctatagtccaagctacttggcaggctgaggcaggaaggtcccttgagtcc 4560
LOC102725072.start-FAM230F.start.18846286-18865042.NEW.ref     aaacacacagctatagtccaagctacttggcaggctgaggcaggaaggtcccttgagtcc 4560

BCRP2.21103016-21122286.NCBI.ref.revcompl                      ------------------------------------------------------------ 0
LOC102725072.has.POM121L1                                      gggaattggaggctgcattgagctataatcgcaccactgcactccagcttgggtgacaaa 4620
LOC102725072.start-FAM230F.start.18846286-18865042.NEW.ref     gggaattggaggctgcattgagctataatcgcaccactgcactccagcttgggtgacaaa 4620

BCRP2.21103016-21122286.NCBI.ref.revcompl                      ------------------------------------------------------------ 0
LOC102725072.has.POM121L1                                      gtgagaccctgtctctaaaagaaaaaaaaattggcctgtgagcatgggtttgattttcaa 4680
LOC102725072.start-FAM230F.start.18846286-18865042.NEW.ref     gtgagaccctgtctctaaaagaaaaaaaaattggcctgtgagcatgggtttgattttcaa 4680

BCRP2.21103016-21122286.NCBI.ref.revcompl                      ------------------------------------------------------------ 0
LOC102725072.has.POM121L1                                      acaggacctggagggtagggacagacagtgctgtcacccttaggtgctgaacactcagaa 4740
LOC102725072.start-FAM230F.start.18846286-18865042.NEW.ref     acaggacctggagggtagggacagacagtgctgtcacccttaggtgctgaacactcagaa 4740

BCRP2.21103016-21122286.NCBI.ref.revcompl                      ------------------------------------------------------------ 0
LOC102725072.has.POM121L1                                      acgggccagcggcagcccttccctcacctgcagacaccagattgggcagaacagcacatg 4800
LOC102725072.start-FAM230F.start.18846286-18865042.NEW.ref     acgggccagcggcagcccttccctcacctgcagacaccagattgggcagaacagcacatg 4800

BCRP2.21103016-21122286.NCBI.ref.revcompl                      ------------------------------------------------------------ 0
LOC102725072.has.POM121L1                                      gcacttgcagctcttgcagtgagggcagaacccagtgtcaacccttctgcctgtgggagg 4860
LOC102725072.start-FAM230F.start.18846286-18865042.NEW.ref     gcacttgcagctcttgcagtgagggcagaacccagtgtcaacccttctgcctgtgggagg 4860

BCRP2.21103016-21122286.NCBI.ref.revcompl                      ------------------------------------------------------------ 0
LOC102725072.has.POM121L1                                      ggctgctgaggcctgcggagaggccagggtggaggctcgtccccttgtccagcccttggc 4920
LOC102725072.start-FAM230F.start.18846286-18865042.NEW.ref     ggctgctgaggcctgcggagaggccagggtggaggctcgtccccttgtccagcccttggc 4920
```

```
BCRP2.21103016-21122286.NCBI.ref.revcompl                       ------------------------------------------------------------ 0
LOC102725072.has.POM121L1                                       gtggtctccaccaggtccccagcccaccagtgcagggcgcccctgagcctgctgctgcca 4980
LOC102725072.start-FAM230F.start.18846286-18865042.NEW.ref      gtggtctccaccaggtccccagcccaccagtgcagggcgcccctgagcctgctgctgcca 4980

BCRP2.21103016-21122286.NCBI.ref.revcompl                       ------------------------------------------------------------ 0
LOC102725072.has.POM121L1                                       tgggccctgtctctacccaggacgtccccacaccctcgcagtgtcagggaaatgatcatg 5040
LOC102725072.start-FAM230F.start.18846286-18865042.NEW.ref      tgggccctgtctctacccaggacgtccccacaccctcgcagtgtcagggaaatgatcatg 5040

BCRP2.21103016-21122286.NCBI.ref.revcompl                       ------------------------------------------------------------ 0
LOC102725072.has.POM121L1                                       gtggcggtgacactccgcaggcagggctgctgagagaagctgagaagggtcacactgcag 5100
LOC102725072.start-FAM230F.start.18846286-18865042.NEW.ref      gtggcggtgacactccgcaggcagggctgctgagagaagctgagaagggtcacactgcag 5100

BCRP2.21103016-21122286.NCBI.ref.revcompl                       ------------------------------------------------------------ 0
LOC102725072.has.POM121L1                                       gcaggggcccgtgtgacaagcccctctcaccccgagagagctgaccaggcagctcacgag 5160
LOC102725072.start-FAM230F.start.18846286-18865042.NEW.ref      gcaggggcccgtgtgacaagcccctctcaccccgagagagctgaccaggcagctcacgag 5160

BCRP2.21103016-21122286.NCBI.ref.revcompl                       ------------------------------------------------------------ 0
LOC102725072.has.POM121L1                                       cagagccacatcccgggagtccgagaaaggtcctggctgggctcagccacctcattggcc 5220
LOC102725072.start-FAM230F.start.18846286-18865042.NEW.ref      cagagccacatcccgggagtccgagaaaggtcctggctgggctcagccacctcattggcc 5220

BCRP2.21103016-21122286.NCBI.ref.revcompl                       ------------------------------------------------------------ 0
LOC102725072.has.POM121L1                                       acgggcagcctttgtcatgtgagccttgctctcctggggaggctcaggctgacagctgat 5280
LOC102725072.start-FAM230F.start.18846286-18865042.NEW.ref      acgggcagcctttgtcatgtgagccttgctctcctggggaggctcaggctgacagctgat 5280

BCRP2.21103016-21122286.NCBI.ref.revcompl                       ------------------------------------------------------------ 0
LOC102725072.has.POM121L1                                       gtgggcattgccgaaggtaacccgtggcccagtgtatatggccgggtctcctcaagctgc 5340
LOC102725072.start-FAM230F.start.18846286-18865042.NEW.ref      gtgggcattgccgaaggtaacccgtggcccagtgtatatggccgggtctcctcaagctgc 5340

BCRP2.21103016-21122286.NCBI.ref.revcompl                       ------------------------------------------------------------ 0
LOC102725072.has.POM121L1                                       attcattcaagtaggacccagggtgcgtgcccatctccagcccagggcagctcccctgta 5400
LOC102725072.start-FAM230F.start.18846286-18865042.NEW.ref      attcattcaagtaggacccagggtgcgtgcccatctccagcccagggcagctcccctgta 5400

BCRP2.21103016-21122286.NCBI.ref.revcompl                       ------------------------------------------------------------ 0
LOC102725072.has.POM121L1                                       agctgggtgagctactgaagccaaggcgggaggcagctgacaacacccacagcccatgcg 5460
LOC102725072.start-FAM230F.start.18846286-18865042.NEW.ref      agctgggtgagctactgaagccaaggcgggaggcagctgacaacacccacagcccatgcg 5460

BCRP2.21103016-21122286.NCBI.ref.revcompl                       ------------------------------------------------------------ 0
LOC102725072.has.POM121L1                                       gaggtggtggaaaggctgaactcagcagcaacaccaaatcctggaccaggcaaaaaccac 5520
LOC102725072.start-FAM230F.start.18846286-18865042.NEW.ref      gaggtggtggaaaggctgaactcagcagcaacaccaaatcctggaccaggcaaaaaccac 5520

BCRP2.21103016-21122286.NCBI.ref.revcompl                       ------------------------------------------------------------ 0
LOC102725072.has.POM121L1                                       ccaagactgaggggctcgtgccagagcggtggccacaggtaagaacccgggcccaggctg 5580
LOC102725072.start-FAM230F.start.18846286-18865042.NEW.ref      ccaagactgaggggctcgtgccagagcggtggccacaggtaagaacccgggcccaggctg 5580

BCRP2.21103016-21122286.NCBI.ref.revcompl                       ------------------------------------------------------------ 0
LOC102725072.has.POM121L1                                       tgtggcaggaatcctccatgtcccagggcttagcatagcaaaggaagaccagccgggtca 5640
LOC102725072.start-FAM230F.start.18846286-18865042.NEW.ref      tgtggcaggaatcctccatgtcccagggcttagcatagcaaaggaagaccagccgggtca 5640

BCRP2.21103016-21122286.NCBI.ref.revcompl                       ------------------------------------------------------------ 0
LOC102725072.has.POM121L1                                       ccctggtggccatctgtccctgtcccacctgcagagtcagaacagcctctccccagtggg 5700
LOC102725072.start-FAM230F.start.18846286-18865042.NEW.ref      ccctggtggccatctgtccctgtcccacctgcagagtcagaacagcctctccccagtggg 5700

BCRP2.21103016-21122286.NCBI.ref.revcompl                       ------------------------------------------------------------ 0
LOC102725072.has.POM121L1                                       gatcatctctctctgccaaagcaacagcggtccctgccccaaccagactaccccactcag 5760
LOC102725072.start-FAM230F.start.18846286-18865042.NEW.ref      gatcatctctctctgccaaagcaacagcggtccctgccccaaccagactaccccactcag 5760

BCRP2.21103016-21122286.NCBI.ref.revcompl                       ------------------------------------------------------------ 0
LOC102725072.has.POM121L1                                       tggagttacggatgctgctccagcatcctaacactgcccagctggtgcctgcctgtgctc 5820
LOC102725072.start-FAM230F.start.18846286-18865042.NEW.ref      tggagttacggatgctgctccagcatcctaacactgcccagctggtgcctgcctgtgctc 5820

BCRP2.21103016-21122286.NCBI.ref.revcompl                       ------------------------------------------------------------ 0
LOC102725072.has.POM121L1                                       acccacacccccccaggccggccttccctgcagcctgggcttggccaccttggcctgattg 5880
LOC102725072.start-FAM230F.start.18846286-18865042.NEW.ref      acccacacccccccaggccggccttccctgcagcctgggcttggccaccttggcctgattg 5880

BCRP2.21103016-21122286.NCBI.ref.revcompl                       ------------------------------------------------------------ 0
LOC102725072.has.POM121L1                                       agcactgaggcctcctgggcacccagccccatcactgcacctgctgcttccagccccacc 5940
LOC102725072.start-FAM230F.start.18846286-18865042.NEW.ref      agcactgaggcctcctgggcacccagccccatcactgcacctgctgcttccagccccacc 5940

BCRP2.21103016-21122286.NCBI.ref.revcompl                       ----------------------------------------------------------tc 2
LOC102725072.has.POM121L1                                       ccaccggctcaggggttcttcccagcggcgctgatcatgaagtcaacatgcacgcaagtc 6000
LOC102725072.start-FAM230F.start.18846286-18865042.NEW.ref      ccaccggctcaggggttcttcccagcggcgctgatcatgaagtcaacatgcacgcaagtc 6000
                                                                                                                          **

BCRP2.21103016-21122286.NCBI.ref.revcompl                       gtctcaggaaacttcttaatgaaagtgtcggccacggtggtgtgtaggtggctgagctca 62
LOC102725072.has.POM121L1                                       gtctcaggaaacttcttaatgaaagtgtcggccacggtggtgtgtaggtggctgagctca 6060
LOC102725072.start-FAM230F.start.18846286-18865042.NEW.ref      gtctcaggaaacttcttaatgaaagtgtcggccacggtggtgtgtaggtggctgagctca 6060
                                                                ************************************************************

BCRP2.21103016-21122286.NCBI.ref.revcompl                       gattgcagctgctaagacaccagccacttaccaagagaaagccaggctgcttcaaaccca 122
LOC102725072.has.POM121L1                                       gattgcagctgctaagacaccagccacttaccaagagaaagccaggctgcttcaaaccca 6120
LOC102725072.start-FAM230F.start.18846286-18865042.NEW.ref      gattgcagctgctaagacaccagccacttaccaagagaaagccaggctgcttcaaaccca 6120
                                                                ************************************************************

BCRP2.21103016-21122286.NCBI.ref.revcompl                       gggcccacggcaaaaaagcatcacttccggccggggagtctggaagccacgccttgtggg 182
LOC102725072.has.POM121L1                                       gggcccacggcaaaaaagcatcacttccggccggggagtctggaagccacgccttgtggg 6180
LOC102725072.start-FAM230F.start.18846286-18865042.NEW.ref      gggcccacggcaaaaaagcatcacttccggccggggagtctggaagccacgccttgtggg 6180
                                                                ************************************************************

BCRP2.21103016-21122286.NCBI.ref.revcompl                       aggtcacactggcatctaggccttcgcctgcattgcagaaggagagccgggtcccccctcc 242
LOC102725072.has.POM121L1                                       aggtcacactggcatctaggccttcgcctgcattgcagaaggagagccgggtcccccctcc 6240
LOC102725072.start-FAM230F.start.18846286-18865042.NEW.ref      aggtcacactggcatctaggccttcgcctgcattgcagaaggagagccgggtcccccctcc 6240
                                                                ************************************************************

BCRP2.21103016-21122286.NCBI.ref.revcompl                       tggagaacgctgcgttccccagccccacaccggctttgccaccacacaggctgttgaggc 302
LOC102725072.has.POM121L1                                       tggagaacgctgcgttccccagccccacaccggctttgccaccacacaggctgttgaggc 6300
LOC102725072.start-FAM230F.start.18846286-18865042.NEW.ref      tggagaacgctgcgttccccagccccacaccggctttgccaccacacaggctgttgaggc 6300
                                                                ************************************************************

BCRP2.21103016-21122286.NCBI.ref.revcompl                       aggaggcgggtaagacgtagctgtagacccaaagcaaccaccagccctgggaccctgcgg 362
LOC102725072.has.POM121L1                                       aggaggcgggtaagacgtagctgtagacccaaagcaaccaccagccctgggaccctgcgg 6360
LOC102725072.start-FAM230F.start.18846286-18865042.NEW.ref      aggaggcgggtaagacgtagctgtagacccaaagcaaccaccagccctgggaccctgcgg 6360
                                                                ************************************************************

BCRP2.21103016-21122286.NCBI.ref.revcompl                       gagaggagcacttttagaacatggaaaagtgtggtcatcccatcattagacagcacacat 422
LOC102725072.has.POM121L1                                       gagaggagcacttttagaacatggaaaagtgtggtcatcccatcattagacagcacacat 6420
LOC102725072.start-FAM230F.start.18846286-18865042.NEW.ref      gagaggagcacttttagaacatggaaaagtgtggtcatcccatcattagacagcacacat 6420
                                                                ************************************************************

BCRP2.21103016-21122286.NCBI.ref.revcompl                       cctacataaataaaaagtcgtatggggaaggaggttggggagggaataaaaaattggcac 482
LOC102725072.has.POM121L1                                       cctacataaataaaaagtcgtatggggaaggaggttggggagggaataaaaaattggcac 6480
LOC102725072.start-FAM230F.start.18846286-18865042.NEW.ref      cctacataaataaaaagtcgtatggggaaggaggttggggagggaataaaaaattggcac 6480
                                                                ************************************************************

BCRP2.21103016-21122286.NCBI.ref.revcompl                       agacattgatagactggtttccagtttcaaggtaacagatgcacatcatgagaccagagg 542
LOC102725072.has.POM121L1                                       agacattgatagactggtttccagtttcaaggtaacagatgcacatcatgagaccagagg 6540
LOC102725072.start-FAM230F.start.18846286-18865042.NEW.ref      agacattgatagactggtttccagtttcaaggtaacagatgcacatcatgagaccagagg 6540
                                                                ************************************************************

BCRP2.21103016-21122286.NCBI.ref.revcompl                       aggcagagacaaggctggatttggcttttctaagcaacacgtgttcctgcgcagggctga 602
LOC102725072.has.POM121L1                                       aggcagagacaaggctggatttggcttttctaagcaacatgtgttcctgcgcagggctga 6600
LOC102725072.start-FAM230F.start.18846286-18865042.NEW.ref      aggcagagacaaggctggatttggcttttctaagcaacatgtgttcctgcgcagggctga 6600
                                                                *************************************** ********************

BCRP2.21103016-21122286.NCBI.ref.revcompl                       atggttgctgagacagagatggaagccaggacaagggagcccaccgggcccagataggta 662
LOC102725072.has.POM121L1                                       atggtcgctgagacagagatggaagccaggacaagggagcccaccgggcccagataggta 6660
LOC102725072.start-FAM230F.start.18846286-18865042.NEW.ref      atggtcgctgagacagagatggaagccaggacaagggagcccaccgggcccagataggta 6660
                                                                ***** ******************************************************
```

```
BCRP2.21103016-21122286.NCBI.ref.revcompl                 cagagagcagaggctcctgttctgtcctcgccacccacgagggtgacactgcttgtaaat 722
LOC102725072.has.POM121L1                                 cagagagcagaggctcctgttctgtcctcgccacccacgagggtgacactgcttgtaaat 6720
LOC102725072.start-FAM230F.start.18846286-18865042.NEW.ref cagagagcagaggctcctgttctgtcctcgccacccacgagggtgacactgcttgtaaat 6720
                                                          ************************************************************

BCRP2.21103016-21122286.NCBI.ref.revcompl                 ggtggctgtgctctcccagcaagaaaaaagcacaactaaatccacactgcacacagacgc 782
LOC102725072.has.POM121L1                                 ggtggctgtgctctcccagcaagaaaaaagcacaactaaatccacactgcacacagacgc 6780
LOC102725072.start-FAM230F.start.18846286-18865042.NEW.ref ggtggctgtgctctcccagcaagaaaaaagcacaactaaatccacactgcacacagacgc 6780
                                                          ************************************************************

BCRP2.21103016-21122286.NCBI.ref.revcompl                 agacagaaagccttcaagtggctctgttttctgctccctgccttgccaggtccacaagca 842
LOC102725072.has.POM121L1                                 agacagaaagccttcaagtggctctgttttctgctccctgccttgccaggtccacaagca 6840
LOC102725072.start-FAM230F.start.18846286-18865042.NEW.ref agacagaaagccttcaagtggctctgttttctgctccctgccttgccaggtccacaagca 6840
                                                          ************************************************************

BCRP2.21103016-21122286.NCBI.ref.revcompl                 gagaggagtgtcaggcacatggccccgctgtcaggctccccagtgagctgcgggctcagc 902
LOC102725072.has.POM121L1                                 gagaggagtgtcaggcacatggccccgctgtcaggctccccagtgagctgcgggctcagc 6900
LOC102725072.start-FAM230F.start.18846286-18865042.NEW.ref gagaggagtgtcaggcacatggccccgctgtcaggctccccagtgagctgcgggctcagc 6900
                                                          ************************************************************

BCRP2.21103016-21122286.NCBI.ref.revcompl                 aggagctgcccactgacacacaggggacacccactcctgccaccttgggagcggttgcca 962
LOC102725072.has.POM121L1                                 aggagctgcccactgacacacaggggacacccactcctgccaccttgggagcggttgcca 6960
LOC102725072.start-FAM230F.start.18846286-18865042.NEW.ref aggagctgcccactgacacacaggggacacccactcctgccaccttgggagcggttgcca 6960
                                                          ************************************************************

BCRP2.21103016-21122286.NCBI.ref.revcompl                 gacagagccgtactgggtgctggtgtcatccagggaccccacacacttccttaaatgtga 1022
LOC102725072.has.POM121L1                                 gacagagccgcactgggtgctggtgtcatccagggaccccacacacttccttaaatgtga 7020
LOC102725072.start-FAM230F.start.18846286-18865042.NEW.ref gacagagccgcactgggtgctggtgtcatccagggaccccacacacttccttaaatgtga 7020
                                                          ********** *************************************************

BCRP2.21103016-21122286.NCBI.ref.revcompl                 tcctgcttccctctgcgcagctgcatcctctcctcctgcaggaccgtctggaaacttggc 1082
LOC102725072.has.POM121L1                                 tcctgcttccctctgcgcagctgcatcctctcctcctgcaggaccgtctggaaacttggc 7080
LOC102725072.start-FAM230F.start.18846286-18865042.NEW.ref tcctgcttccctctgcgcagctgcatcctctcctcctgcaggaccgtctggaaacttggc 7080
                                                          ************************************************************

BCRP2.21103016-21122286.NCBI.ref.revcompl                 tctcagtttgctctcccttctctcctctgcctgccccaagcccctctttctaaaaaagtg 1142
LOC102725072.has.POM121L1                                 tctcagtttgctctcccttctctcctctgcctgccccaagcccctctttctaaaaaagtg 7140
LOC102725072.start-FAM230F.start.18846286-18865042.NEW.ref tctcagtttgctctcccttctctcctctgcctgccccaagcccctctttctaaaaaagtg 7140
                                                          ************************************************************

BCRP2.21103016-21122286.NCBI.ref.revcompl                 atgccacgttcatgggattatttcttgaaaatacttggcggcctccatgcttctgttttc 1202
LOC102725072.has.POM121L1                                 atgccatgttcatggggttatttcttgaaaatacttggcggcctccatgcttctgttttc 7200
LOC102725072.start-FAM230F.start.18846286-18865042.NEW.ref atgccatgttcatggggttatttcttgaaaatacttggcggcctccatgcttctgttttc 7200
                                                          ****** ********* *******************************************

BCRP2.21103016-21122286.NCBI.ref.revcompl                 tttgagtcaggtagtcaggagggtttacaaacaatgcctgggctcccccgcaggtgccgg 1262
LOC102725072.has.POM121L1                                 tttgagccaggtggtcaggagggcttacaaacaatgcctgggctcccccgcagctgtcgg 7260
LOC102725072.start-FAM230F.start.18846286-18865042.NEW.ref tttgagccaggtggtcaggagggcttacaaacaatgcctgggctcccccgcagctgtcgg 7260
                                                          ****** ***** ********** ***************************** ** ***

BCRP2.21103016-21122286.NCBI.ref.revcompl                 cagatggggtagcgaatggtcctgtgcctccacctgctccgggagggagtctcccgtctc 1322
LOC102725072.has.POM121L1                                 cagatggggtagcgaatggtcctgtgcctccacctgctccgggagggagtctcccgtctc 7320
LOC102725072.start-FAM230F.start.18846286-18865042.NEW.ref cagatggggtagcgaatggtcctgtgcctccacctgctccgggagggagtctcccgtctc 7320
                                                          ************************************************************

BCRP2.21103016-21122286.NCBI.ref.revcompl                 taggcctggccccttcctaaccctccacgtatcctgttctccagagacttcagaacccac 1382
LOC102725072.has.POM121L1                                 taggcctagccccttcctaaccctccacgtatcctgttctccagagacttcagaacccac 7380
LOC102725072.start-FAM230F.start.18846286-18865042.NEW.ref taggcctagccccttcctaaccctccacgtatcctgttctccagagacttcagaacccac 7380
                                                          ******* ****************************************************

BCRP2.21103016-21122286.NCBI.ref.revcompl                 tcctgagaacagcggagccaggcgcttagaggaagaccaaatgctgccaggacacggatt 1442
LOC102725072.has.POM121L1                                 tcctgagaacagcggagccaggcgcttagaggaagaccaaatgctgccaggacacggatt 7440
LOC102725072.start-FAM230F.start.18846286-18865042.NEW.ref tcctgagaacagcggagccaggcgcttagaggaagaccaaatgctgccaggacacggatt 7440
                                                          ************************************************************

BCRP2.21103016-21122286.NCBI.ref.revcompl                 gtccagggattacattccagcatcttattaggtatctggatctgttggggaaaaaattag 1502
LOC102725072.has.POM121L1                                 gtcca-ggattacattccagcatcttattaggtatctggatctgttggggaaaaaattag 7499
LOC102725072.start-FAM230F.start.18846286-18865042.NEW.ref gtcca-ggattacattccagcatcttattaggtatctggatctgttggggaaaaaattag 7499
                                                          ***** ******************************************************

BCRP2.21103016-21122286.NCBI.ref.revcompl                 aaactatgtataaaacttacaaatattcaagtatcaaaaggttatttaggatgaaagttt 1562
LOC102725072.has.POM121L1                                 aaactatgtataaaacttaaaaatattcaagcatcaaaaggttatttaggatgaaagttt 7559
LOC102725072.start-FAM230F.start.18846286-18865042.NEW.ref aaactatgtataaaacttaaaaatattcaagcatcaaaaggttatttaggatgaaagttt 7559
                                                          ******************* *********** ****************************

BCRP2.21103016-21122286.NCBI.ref.revcompl                 taaaacaagtcatcagcaagctgctaccaccaagtggagacttatacaaaagttgagcga 1622
LOC102725072.has.POM121L1                                 taaaacaagtcatcagcaagctgctaccaccaagtggagacttatacaaaagttgagcga 7619
LOC102725072.start-FAM230F.start.18846286-18865042.NEW.ref taaaacaagtcatcagcaagctgctaccaccaagtggagacttatacaaaagttgagcga 7619
                                                          ************************************************************

BCRP2.21103016-21122286.NCBI.ref.revcompl                 gtccactgagctgagaggacagaaatgaagtcacctgtgctggggcaggggcagggacac 1682
LOC102725072.has.POM121L1                                 gtccactgagctgagaggacagaaatgaagtcacctgtgctggggcaggggcagggacac 7679
LOC102725072.start-FAM230F.start.18846286-18865042.NEW.ref gtccactgagctgagaggacagaaatgaagtcacctgtgctggggcaggggcagggacac 7679
                                                          ************************************************************

BCRP2.21103016-21122286.NCBI.ref.revcompl                 tgggggcagggagtgtgtgggcagagaagccagagaagtccaggcctgtggaagccaaac 1742
LOC102725072.has.POM121L1                                 tgggggcagggagtgtgtgggcagagaagccagagaagtccaggcctgtggaagccaaac 7739
LOC102725072.start-FAM230F.start.18846286-18865042.NEW.ref tgggggcagggagtgtgtgggcagagaagccagagaagtccaggcctgtggaagccaaac 7739
                                                          ************************************************************

BCRP2.21103016-21122286.NCBI.ref.revcompl                 aggagagcgtgggccggaagggcggtcaggatcgggggacgaggtcgctctccctggaga 1802
LOC102725072.has.POM121L1                                 aggagagcgtgggccggaagggcggtcaggatcgggggacgaggtcgctctccctggaga 7799
LOC102725072.start-FAM230F.start.18846286-18865042.NEW.ref aggagagcgtgggccggaagggcggtcaggatcgggggacgaggtcgctctccctggaga 7799
                                                          ************************************************************

BCRP2.21103016-21122286.NCBI.ref.revcompl                 acgaaccctaaagtgcgtagcctgggattccctccctgggggtcctgtcccccgacgttt 1862
LOC102725072.has.POM121L1                                 acgaaccctaaggtgcatagcctgggattccctccctggaggtcctgtcccccgacattt 7859
LOC102725072.start-FAM230F.start.18846286-18865042.NEW.ref acgaaccctaaggtgcatagcctgggattccctccctggaggtcctgtcccccgacattt 7859
                                                          *********** **** ********************** **************** ***

BCRP2.21103016-21122286.NCBI.ref.revcompl                 cacgggccttctgagctgccttccaaggaggactaacacggcaacaaaagacccatttct 1922
LOC102725072.has.POM121L1                                 cacgggccttctgagctgccttccaaggaggactaacacggcaacaaaagaaccatttct 7919
LOC102725072.start-FAM230F.start.18846286-18865042.NEW.ref cacgggccttctgagctgccttccaaggaggactaacacggcaacaaaagaaccatttct 7919
                                                          *************************************************** ********

BCRP2.21103016-21122286.NCBI.ref.revcompl                 gcacaaaaatccctctgggaagaaaaagaagaaagccaagaatggagtcaaaacgctacc 1982
LOC102725072.has.POM121L1                                 gcacaaaaatccttctgggaagaaaaagaagaaagccaagaatggagtcaaaacgctacc 7979
LOC102725072.start-FAM230F.start.18846286-18865042.NEW.ref gcacaaaaatccttctgggaagaaaaagaagaaagccaagaatggagtcaaaacgctacc 7979
                                                          ************ ***********************************************

BCRP2.21103016-21122286.NCBI.ref.revcompl                 cagtgctgaccaagcctctcaaaccctgttctaagtggactgtggtttctaagtcaggga 2042
LOC102725072.has.POM121L1                                 cagtgctgactaagcctctcaaaccctgttctaggtggactgtggtttctaagtcaggga 8039
LOC102725072.start-FAM230F.start.18846286-18865042.NEW.ref cagtgctgactaagcctctcaaaccctgttctaggtggactgtggtttctaagtcaggga 8039
                                                          ********** ********************** **************************

BCRP2.21103016-21122286.NCBI.ref.revcompl                 aatggaagaggccccacccacacagggacagggccatggcccccacaggatgaagcagca 2102
LOC102725072.has.POM121L1                                 aatggaagaggccccacccacacagggacagggccatggcccccacaggatgaagcagca 8099
LOC102725072.start-FAM230F.start.18846286-18865042.NEW.ref aatggaagaggccccacccacacagggacagggccatggcccccacaggatgaagcagca 8099
                                                          ************************************************************

BCRP2.21103016-21122286.NCBI.ref.revcompl                 gcgtttattcaagatacaacagtgagggaatccagtcacgttcccttctccccagagagg 2162
LOC102725072.has.POM121L1                                 gcgtttattcaagatacaacagtgagggaatccggtcacgttcccttctccccagagagg 8159
LOC102725072.start-FAM230F.start.18846286-18865042.NEW.ref gcgtttattcaagatacaacagtgagggaatccggtcacgttcccttctccccagagagg 8159
                                                          ********************************* **************************

BCRP2.21103016-21122286.NCBI.ref.revcompl                 gcgcttcttgacaagtgattcagtagaaatcttttggactctataagttaagttcataaa 2222
LOC102725072.has.POM121L1                                 gcgcttcttgacaagtgattcagtagaaatcttttagactctataagttaagttcataaa 8219
LOC102725072.start-FAM230F.start.18846286-18865042.NEW.ref gcgcttcttgacaagtgattcagtagaaatcttttagactctataagttaagttcataaa 8219
                                                          *********************************** ************************

BCRP2.21103016-21122286.NCBI.ref.revcompl                 aaccactgctttcaccctgtctcccagggccaggcctggactccgagatgaactggtttg 2282
LOC102725072.has.POM121L1                                 aaccactgctttcaccctgtctcccagggccaggcctggactccgagatgaactggtttg 8279
LOC102725072.start-FAM230F.start.18846286-18865042.NEW.ref aaccactgctttcaccctgtctcccagggccaggcctggactccgagatgaactggtttg 8279
                                                          ************************************************************

BCRP2.21103016-21122286.NCBI.ref.revcompl                 gggcgccctcgggtggccacataaaaaacccacagtctgaggccagcctggggctttcag 2342
LOC102725072.has.POM121L1                                 gggcgccctcgggtggccacataaaaaacccacagtctgaggccagcctggggctttcag 8339
LOC102725072.start-FAM230F.start.18846286-18865042.NEW.ref gggcgccctcgggtggccacataaaaaacccacagtctgaggccagcctggggctttcag 8339
                                                          ************************************************************

BCRP2.21103016-21122286.NCBI.ref.revcompl                 acctgggcgggatctgcccaggccacctgtccttctgctttgggccgctgtctcttggca 2402
LOC102725072.has.POM121L1                                 acctgggcgggatctgcccaggccacctgtccttctgctttgggccgctgtctcttggca 8399
LOC102725072.start-FAM230F.start.18846286-18865042.NEW.ref acctgggcgggatctgcccaggccacctgtccttctgctttgggccgctgtctcttggca 8399
                                                          ************************************************************
```

```
BCRP2.21103016-21122286.NCBI.ref.revcompl                  gatggcctgacacctgggggtggcccaaggatgcctcagaaaatcttgattcccactcta 2462
LOC102725072.has.POM121L1                                  gatggcctgacacctgggggtggcccaaggatgcctcagaaaatcttgattcccactcta 8459
LOC102725072.start-FAM230F.start.18846286-18865042.NEW.ref gatggcctgacacctgggggtggcccaaggatgcctcagaaaatcttgattcccactcta 8459
                                                           ************************************************************

BCRP2.21103016-21122286.NCBI.ref.revcompl                  cagatggcctgattagccagaggtttccaggccgtctgtccgcctccaggagatggactg 2522
LOC102725072.has.POM121L1                                  cagatggcctgattagccagaggtttccaggccgtctgtccgcctccaggagatggactg 8519
LOC102725072.start-FAM230F.start.18846286-18865042.NEW.ref cagatggcctgattagccagaggtttccaggccgtctgtccgcctccaggagatggactg 8519
                                                           ************************************************************

BCRP2.21103016-21122286.NCBI.ref.revcompl                  ggacctttagacatcggtggagaacaggatgctctgtcccttgctgtccagggcagggat 2582
LOC102725072.has.POM121L1                                  ggacctttagacatcggtggagaacaggatgctctgtcccttgctgtccagggcagggat 8579
LOC102725072.start-FAM230F.start.18846286-18865042.NEW.ref ggacctttagacatcggtggagaacaggatgctctgtcccttgctgtccagggcagggat 8579
                                                           ************************************************************

BCRP2.21103016-21122286.NCBI.ref.revcompl                  ggcctccagccgcaagaagtacagcagcacctcgacctgccctcgcggagtggggaagag 2642
LOC102725072.has.POM121L1                                  ggcctccagccgcaagaagtacagcagcacctcgacctgccctcgcggagtggggaagag 8639
LOC102725072.start-FAM230F.start.18846286-18865042.NEW.ref ggcctccagccgcaagaagtacagcagcacctcgacctgccctcgcggagtggggaagag 8639
                                                           ************************************************************

BCRP2.21103016-21122286.NCBI.ref.revcompl                  gagagtggctcggaagggggcgcacagctgctggt-gggaggtctttggggcccaagatc 2701
LOC102725072.has.POM121L1                                  gagagtggctcagagcggggctcacagctgctggtggggaggtctttggggcccaagctc 8699
LOC102725072.start-FAM230F.start.18846286-18865042.NEW.ref gagagtggctcagagcggggctcacagctgctggtggggaggtctttggggcccaagctc 8699
                                                           *********** **  ***** ************* ********************* **

BCRP2.21103016-21122286.NCBI.ref.revcompl                  ccaagtccacctcaggtgctagaaacccctgctggtgtcatgaaccccttacagtgagac 2761
LOC102725072.has.POM121L1                                  ccaagtccacctcaggtgctagaaacccctgctggtgtcatgaaccccttacagtgggac 8759
LOC102725072.start-FAM230F.start.18846286-18865042.NEW.ref ccaagtccacctcaggtgctagaaacccctgctggtgtcatgaaccccttacagtgggac 8759
                                                           ******************************************************** ***

BCRP2.21103016-21122286.NCBI.ref.revcompl                  gggggtggggtggggtcctgacaaggcatgacttgttgggtgggggtggttatttattt 2821
LOC102725072.has.POM121L1                                  gggggtggggtggggtcctgacaaggcatgacttgttgggtgaggggtggttatttattt 8819
LOC102725072.start-FAM230F.start.18846286-18865042.NEW.ref gggggtggggtggggtcctgacaaggcatgacttgttgggtgaggggtggttatttattt 8819
                                                           ****************************************** *****************

BCRP2.21103016-21122286.NCBI.ref.revcompl                  tagagatgcacagggccttgctctgtcccccaggctggagtacagtggctccatcatgga 2881
LOC102725072.has.POM121L1                                  tagagatgcacagggccttgctctgtcccccaggctggagtacagtggctccatcatgga 8879
LOC102725072.start-FAM230F.start.18846286-18865042.NEW.ref tagagatgcacagggccttgctctgtcccccaggctggagtacagtggctccatcatgga 8879
                                                           ************************************************************

BCRP2.21103016-21122286.NCBI.ref.revcompl                  tcactgcagcctctaactcctgggctcaagcaatcctcctgtgtcagcctcccagatacc 2941
LOC102725072.has.POM121L1                                  tcactgcagcctctaactcctgggctcaagcaatcctcctgtgtcagcctcccagatacc 8939
LOC102725072.start-FAM230F.start.18846286-18865042.NEW.ref tcactgcagcctctaactcctgggctcaagcaatcctcctgtgtcagcctcccagatacc 8939
                                                           ************************************************************

BCRP2.21103016-21122286.NCBI.ref.revcompl                  taggattacagatatgtgccccaatgcctgcctaatttttctttgtatttttttctggaga 3001
LOC102725072.has.POM121L1                                  taggattacagatgtgtgccccaatgcctgcctaatttttctttgtatttttttctggaga 8999
LOC102725072.start-FAM230F.start.18846286-18865042.NEW.ref taggattacagatgtgtgccccaatgcctgcctaatttttctttgtatttttttctggaga 8999
                                                           ************* **********************************************

BCRP2.21103016-21122286.NCBI.ref.revcompl                  tggggtttgctacattgcccagactggtctcaaacacctgggttcagttgtcctgcctcg 3061
LOC102725072.has.POM121L1                                  tggggtttgctacattgcccagactggtctcaaacacctgggttcagttgtcctgcctcg 9059
LOC102725072.start-FAM230F.start.18846286-18865042.NEW.ref tggggtttgctacattgcccagactggtctcaaacacctgggttcagttgtcctgcctcg 9059
                                                           ************************************************************

BCRP2.21103016-21122286.NCBI.ref.revcompl                  gcctcccaaagtgctgggattacaggcatgagccaccacactcgaacacttggggtggtt 3121
LOC102725072.has.POM121L1                                  gcctcccaaagtgctgggattacaggcatgagccaccacactcgaacacttggggtggtt 9119
LOC102725072.start-FAM230F.start.18846286-18865042.NEW.ref gcctcccaaagtgctgggattacaggcatgagccaccacactcgaacacttggggtggtt 9119
                                                           ************************************************************

BCRP2.21103016-21122286.NCBI.ref.revcompl                  ttaagcccccagcaaggtgcaccagcaggaccaggaggtggcctaggcaccccctatcac 3181
LOC102725072.has.POM121L1                                  ttaagcccccagcaaggtgcaccagcaggaccaggaggtggcctgggcaccccctatcac 9179
LOC102725072.start-FAM230F.start.18846286-18865042.NEW.ref ttaagcccccagcaaggtgcaccagcaggaccaggaggtggcctgggcaccccctatcac 9179
                                                           ******************************************** ***************

BCRP2.21103016-21122286.NCBI.ref.revcompl                  tcccatccatgcaaacctaggcaagtccctgtctctgaatctcagccaccaccacataca 3241
LOC102725072.has.POM121L1                                  tcccatccatgcaaacctaggcaagtccctgtctctgaatctcagccaccaccacataca 9239
LOC102725072.start-FAM230F.start.18846286-18865042.NEW.ref tcccatccatgcaaacctaggcaagtccctgtctctgaatctcagccaccaccacataca 9239
                                                           ************************************************************

BCRP2.21103016-21122286.NCBI.ref.revcompl                  atgcaagtcggaagatgggcaggactgggggtggggcaggcagaggccacctctgtcagg 3301
LOC102725072.has.POM121L1                                  atgcaagtgggaagatgggcaggactgggggtggggcaggcagaggccacctctgtcagg 9299
LOC102725072.start-FAM230F.start.18846286-18865042.NEW.ref atgcaagtgggaagatgggcaggactgggggtggggcaggcagaggccacctctgtcagg 9299
                                                           ******** ***************************************************

BCRP2.21103016-21122286.NCBI.ref.revcompl                  ctggggttgcatgggctggaggctgtcttcccatacctgggacatgacctccaaggacca 3361
LOC102725072.has.POM121L1                                  ctggggttgcatgggctggaggctgtcttcccatacctgggacatgacctccaaggacca 9359
LOC102725072.start-FAM230F.start.18846286-18865042.NEW.ref ctggggttgcatgggctggaggctgtcttcccatacctgggacatgacctccaaggacca 9359
                                                           ************************************************************

BCRP2.21103016-21122286.NCBI.ref.revcompl                  gctgtcagtcatggtgatgggctggctggggttggcagggagcttgctctccttctcgga 3421
LOC102725072.has.POM121L1                                  gctgtcagtcatggtgatgggctggctggggttggcagggagcttgctctccttctcgga 9419
LOC102725072.start-FAM230F.start.18846286-18865042.NEW.ref gctgtcagtcatggtgatgggctggctggggttggcagggagcttgctctccttctcgga 9419
                                                           ************************************************************

BCRP2.21103016-21122286.NCBI.ref.revcompl                  gggccggagcagcgtggggccaaacaccgtgccaaggttgtgcagggacatcttattgac 3481
LOC102725072.has.POM121L1                                  gggccggagcagcgtggggccaaacaccgtgccaaggttgtgcagggacatcttattgac 9479
LOC102725072.start-FAM230F.start.18846286-18865042.NEW.ref gggccggagcagcgtggggccaaacaccgtgccaaggttgtgcagggacatcttattgac 9479
                                                           ************************************************************

BCRP2.21103016-21122286.NCBI.ref.revcompl                  tgcctccttctctgccatcctgtagaggaccgaagcagagggtgctgtttcaacgccacc 3541
LOC102725072.has.POM121L1                                  tgcctccttctctgccatcctgtagaggaccgaagcagagggtgctgtttcaacgccacc 9539
LOC102725072.start-FAM230F.start.18846286-18865042.NEW.ref tgcctccttctctgccatcctgtagaggaccgaagcagagggtgctgtttcaacgccacc 9539
                                                           ************************************************************

BCRP2.21103016-21122286.NCBI.ref.revcompl                  accaggagagaggcagaggggctgtgccgtgctagagtcctcagggagggagtgacctcg 3601
LOC102725072.has.POM121L1                                  accaggagagaggcagaggggctgtgccgtgctagagtcctcagggagggagtgacctcg 9599
LOC102725072.start-FAM230F.start.18846286-18865042.NEW.ref accaggagagaggcagaggggctgtgccgtgctagagtcctcagggagggagtgacctcg 9599
                                                           ************************************************************

BCRP2.21103016-21122286.NCBI.ref.revcompl                  accctggctgtgctgcaagctgactccagccttggtacttctgggtctcagtggcccagg 3661
LOC102725072.has.POM121L1                                  accctggctgtgctgcaagctgactccagccttggtacttctgggtctcagtggcccagg 9659
LOC102725072.start-FAM230F.start.18846286-18865042.NEW.ref accctggctgtgctgcaagctgactccagccttggtacttctgggtctcagtggcccagg 9659
                                                           ************************************************************

BCRP2.21103016-21122286.NCBI.ref.revcompl                  acaaggggccagctctgggctgatggggaggtcttcctgatgtgcttgggagggaagggg 3721
LOC102725072.has.POM121L1                                  acaaggggccagctctgggctgatggggaggtcttcatgatgtgcttgggagggaagggg 9719
LOC102725072.start-FAM230F.start.18846286-18865042.NEW.ref acaaggggccagctctgggctgatggggaggtcttcatgatgtgcttgggagggaagggg 9719
                                                           ************************************ ***********************

BCRP2.21103016-21122286.NCBI.ref.revcompl                  gggcggtccaaatgcactgctggccacggccaaagctctgagctctttgttaaggccaca 3781
LOC102725072.has.POM121L1                                  gggcggtccaaatgcactgctggccacggccaaagctctgagctctttgttaaggccaca 9779
LOC102725072.start-FAM230F.start.18846286-18865042.NEW.ref gggcggtccaaatgcactgctggccacggccaaagctctgagctctttgttaaggccaca 9779
                                                           ************************************************************

BCRP2.21103016-21122286.NCBI.ref.revcompl                  gtgcagagggaggagggtggcaaagaggagaggcaggggcgggggtggcagtggtgctag 3841
LOC102725072.has.POM121L1                                  gtgcagagggaggagggtggcaaagaggagaggcaggggcgggggtggcagtggtgctag 9839
LOC102725072.start-FAM230F.start.18846286-18865042.NEW.ref gtgcagagggaggagggtggcaaagaggagaggcaggggcgggggtggcagtggtgctag 9839
                                                           ************************************************************

BCRP2.21103016-21122286.NCBI.ref.revcompl                  tccttagaagcagtgagttactgcagacaggggtcgggggaaaaggtccttggtgctggg 3901
LOC102725072.has.POM121L1                                  tccttagaagcagtgagttactgcagacaggggtcagggg-ataggtccgtggtgctggg 9898
LOC102725072.start-FAM230F.start.18846286-18865042.NEW.ref tccttagaagcagtgagttactgcagacaggggtcagggg-ataggtccgtggtgctggg 9898
                                                           *********************************** **** * ****** **********

BCRP2.21103016-21122286.NCBI.ref.revcompl                  ggtctggtgggagcagaggggcaccccacggcctggagacctggagtcctgggcagccac 3961
LOC102725072.has.POM121L1                                  ggtctggtgggagcagaggggcaccccacggcctggagacctggtgtcctgggcagccac 9958
LOC102725072.start-FAM230F.start.18846286-18865042.NEW.ref ggtctggtgggagcagaggggcaccccacggcctggagacctggtgtcctgggcagccac 9958
                                                           ******************************************** ***************

BCRP2.21103016-21122286.NCBI.ref.revcompl                  aagagagctgggctacctttccaggtggtctagaaggaaaaggaaggtgagcaggttggc 4021
LOC102725072.has.POM121L1                                  aagagagctgggctacctttccaggcggtctagaaggaaaaggaaggtgagcaggttggc 10018
LOC102725072.start-FAM230F.start.18846286-18865042.NEW.ref aagagagctgggctacctttccaggcggtctagaaggaaaaggaaggtgagcaggttggc 10018
                                                           ************************* **********************************

BCRP2.21103016-21122286.NCBI.ref.revcompl                  ctccggcagggacgacagcaggttgagcatgcagctcttctttgcaactgggtctgaaag 4081
LOC102725072.has.POM121L1                                  ctccggcagggacgacaacaggttgagcatgcagctcttctttgcaactgggtctgaaag 10078
LOC102725072.start-FAM230F.start.18846286-18865042.NEW.ref ctccggcagggacgacaacaggttgagcatgcagctcttctttgcaactgggtctgaaag 10078
                                                           ***************** ******************************************

BCRP2.21103016-21122286.NCBI.ref.revcompl                  agctgcaggaggcagtgggtcactcccctgggttacgacaagccggagacctctcccgag 4141
LOC102725072.has.POM121L1                                  agctgcaggaggcagtgggtcactcccctgggttacgacaagccggagacctctcccgag 10138
LOC102725072.start-FAM230F.start.18846286-18865042.NEW.ref agctgcaggaggcagtgggtcactcccctgggttacgacaagccggagacctctcccgag 10138
                                                           ************************************************************
```

```
BCRP2.21103016-21122286.NCBI.ref.revcompl                  gtggtcacatggagcgccagggacacgagtccttgcgcagtttaggcttgtcatcatcgt 4201
LOC102725072.has.POM121L1                                  gtggtcacatggagcgcccgggacacgagtccttgcgcagtttaggcttgtcatcatcgt 10198
LOC102725072.start-FAM230F.start.18846286-18865042.NEW.ref gtggtcacatggagcgcccgggacacgagtccttgcgcagtttaggcttgtcatcatcgt 10198
                                                           ****************** *****************************************

BCRP2.21103016-21122286.NCBI.ref.revcompl                  cacacccacagcgctggccgccagtgaggaccctgtgaggggcacctgtgtggggtgtga 4261
LOC102725072.has.POM121L1                                  cacacccacagcgctggccgccagtgaggaccctgtgaggggcacctgtgtggggtgtga 10258
LOC102725072.start-FAM230F.start.18846286-18865042.NEW.ref cacacccacagcgctggccgccagtgaggaccctgtgaggggcacctgtgtggggtgtga 10258
                                                           ************************************************************

BCRP2.21103016-21122286.NCBI.ref.revcompl                  accacctgaacgccttttctctacctcgcaggggtcagcagcacccggcaaacagcagca 4321
LOC102725072.has.POM121L1                                  accacctgaacgccttttctctgcctcgcaggggtcagcagcacccggcaaacagcagca 10318
LOC102725072.start-FAM230F.start.18846286-18865042.NEW.ref accacctgaacgccttttctctgcctcgcaggggtcagcagcacccggcaaacagcagca 10318
                                                           ********************** *************************************

BCRP2.21103016-21122286.NCBI.ref.revcompl                  ggaggagccgctagagcagctgctcatgggcagagctgccctcgggcaactcctgccacc 4381
LOC102725072.has.POM121L1                                  ggaggagccgctagagcagctgctcatgggcagagctgccctcgggcaactcctgccacc 10378
LOC102725072.start-FAM230F.start.18846286-18865042.NEW.ref ggaggagccgctagagcagctgctcatgggcagagctgccctcgggcaactcctgccacc 10378
                                                           ************************************************************

BCRP2.21103016-21122286.NCBI.ref.revcompl                  acccccctccccagggagcccaaggcaggggaggctcagcatggaatgaaacaggggagtg 4441
LOC102725072.has.POM121L1                                  accccctccccagggagcccaaggcaggggaggctcagcatggaatgaaacaggggagtg 10438
LOC102725072.start-FAM230F.start.18846286-18865042.NEW.ref accccctccccagggagcccaaggcaggggaggctcagcatggaatgaaacaggggagtg 10438
                                                           ************************************************************

BCRP2.21103016-21122286.NCBI.ref.revcompl                  agggacacaaggaggtgggaagtgggagggtcccagccccaccaagtacgcagagacccc 4501
LOC102725072.has.POM121L1                                  agggacacaaggaggtgggaagtgggagggtcccagccccaccaagtacgcagagaaccc 10498
LOC102725072.start-FAM230F.start.18846286-18865042.NEW.ref agggacacaaggaggtgggaagtgggagggtcccagccccaccaagtacgcagagaaccc 10498
                                                           ******************************************************** ***

BCRP2.21103016-21122286.NCBI.ref.revcompl                  ctcgacatcctggacaccacaggggcacctgcaggctgggagaccaggtcctctgtgcat 4561
LOC102725072.has.POM121L1                                  ctcgttgtcctggacaccacaggggcacctgcaggctgggagaccaggtcctctgtgcat 10558
LOC102725072.start-FAM230F.start.18846286-18865042.NEW.ref ctcgttgtcctggacaccacaggggcacctgcaggctgggagaccaggtcctctgtgcat 10558
                                                           ****   *****************************************************

BCRP2.21103016-21122286.NCBI.ref.revcompl                  gggcccgggaggcagacctgccctaagggtgatgcacaggctacaggtgctgcacgctcc 4621
LOC102725072.has.POM121L1                                  gggcccgggaggcagacctgccctaagggtgatgcacaggctacaggtgctgcacgctcc 10618
LOC102725072.start-FAM230F.start.18846286-18865042.NEW.ref gggcccgggaggcagacctgccctaagggtgatgcacaggctacaggtgctgcacgctcc 10618
                                                           ************************************************************

BCRP2.21103016-21122286.NCBI.ref.revcompl                  agcgcccactctagacatcagcctccaggttgactaagggtcaggtcatgtttgaaacca 4681
LOC102725072.has.POM121L1                                  agcgcccactctagacatcagcctccaggttgactaagggtcaggtcatgtttgaaacca 10678
LOC102725072.start-FAM230F.start.18846286-18865042.NEW.ref agcgcccactctagacatcagcctccaggttgactaagggtcaggtcatgtttgaaacca 10678
                                                           ************************************************************

BCRP2.21103016-21122286.NCBI.ref.revcompl                  tgcttggctggaccaggacccatggcaagagcacctgggcaccagtgtttagccctggtc 4741
LOC102725072.has.POM121L1                                  tgcttggctggaccaggacccatggcaagagcacctgggcaccagtgtttagccctggtc 10738
LOC102725072.start-FAM230F.start.18846286-18865042.NEW.ref tgcttggctggaccaggacccatggcaagagcacctgggcaccagtgtttagccctggtc 10738
                                                           ************************************************************

BCRP2.21103016-21122286.NCBI.ref.revcompl                  tgcaggaaggaggacagcagactttaggaccccacagcacggcagtgctgaccatttcac 4801
LOC102725072.has.POM121L1                                  tgcaggaaggaggacagcagactttaggaccccacagcacggcagtgctgaccatttcac 10798
LOC102725072.start-FAM230F.start.18846286-18865042.NEW.ref tgcaggaaggaggacagcagactttaggaccccacagcacggcagtgctgaccatttcac 10798
                                                           ************************************************************

BCRP2.21103016-21122286.NCBI.ref.revcompl                  ccacttggcctccttgagaaatatggatggggagccctctggggatgggcaaggccttcc 4861
LOC102725072.has.POM121L1                                  ccacttggcctccttgagaaatatggatggggacccctctggggatgggcaaggccttcc 10858
LOC102725072.start-FAM230F.start.18846286-18865042.NEW.ref ccacttggcctccttgagaaatatggatggggacccctctggggatgggcaaggccttcc 10858
                                                           ********************************* **************************

BCRP2.21103016-21122286.NCBI.ref.revcompl                  aggataggctcagttttggtcccctgctttttgaggttgggttaaaattccgaccatggc 4921
LOC102725072.has.POM121L1                                  aggataggctcagttttggtcccctgctttttgaggttgggttaaaattccgaccatggc 10918
LOC102725072.start-FAM230F.start.18846286-18865042.NEW.ref aggataggctcagttttggtcccctgctttttgaggttgggttaaaattccgaccatggc 10918
                                                           ************************************************************

BCRP2.21103016-21122286.NCBI.ref.revcompl                  agaggaagcacagctcgggttcccacacctcacttttcacagcctctgagggcagcagtg 4981
LOC102725072.has.POM121L1                                  agaggaagcacagctcaggttcccacacctcacttttcacagcctctgagggcagcagtg 10978
LOC102725072.start-FAM230F.start.18846286-18865042.NEW.ref agaggaagcacagctcaggttcccacacctcacttttcacagcctctgagggcagcagtg 10978
                                                           **************** *******************************************

BCRP2.21103016-21122286.NCBI.ref.revcompl                  cacgtggaggagacgtctcccatgaggccaaggcctccagtgctcaccgatgccctctgc 5041
LOC102725072.has.POM121L1                                  cacgtggaggagacgtctcccatgaggccaaggcctccagtgctcaccgatgccctctgc 11038
LOC102725072.start-FAM230F.start.18846286-18865042.NEW.ref cacgtggaggagacgtctcccatgaggccaaggcctccagtgctcaccgatgccctctgc 11038
                                                           ************************************************************

BCRP2.21103016-21122286.NCBI.ref.revcompl                  gaagttggggtagaactcgtcagtgaagaggggctcgggcagctcacggaagtacagctt 5101
LOC102725072.has.POM121L1                                  gaagttggggtagaactcgtcagtgaagaggggctcgggcagctcacggaaatacagctt 11098
LOC102725072.start-FAM230F.start.18846286-18865042.NEW.ref gaagttggggtagaactcgtcagtgaagaggggctcgggcagctcacggaaatacagctt 11098
                                                           *************************************************** ********

BCRP2.21103016-21122286.NCBI.ref.revcompl                  cagcatgcctgcgatggcgttcacgtccatctcgctcatcatcactgacacgtccttgtt 5161
LOC102725072.has.POM121L1                                  cagcgtgcctgcgatggcgttcacgtccatctcgctcatcatcaccgacacgtccttgtt 11158
LOC102725072.start-FAM230F.start.18846286-18865042.NEW.ref cagcgtgcctgcgatggcgttcacgtccatctcgctcatcatcaccgacacgtccttgtt 11158
                                                           **** **************************************** **************

BCRP2.21103016-21122286.NCBI.ref.revcompl                  atctggaaagagcacggaaatgcagcggcctccttgaagatcctgagtgagtcacccacc 5221
LOC102725072.has.POM121L1                                  atctggaaagagcacggaaatgcagcggcctccttgaagatcctgagtgagtcacccacc 11218
LOC102725072.start-FAM230F.start.18846286-18865042.NEW.ref atctggaaagagcacggaaatgcagcggcctccttgaagatcctgagtgagtcacccacc 11218
                                                           ************************************************************

BCRP2.21103016-21122286.NCBI.ref.revcompl                  atccctgccttggctaaagcaccgtccctgccatgctgaccactgtgtgggtccctcctg 5281
LOC102725072.has.POM121L1                                  atccctgccttggctaaagcaccgtccctgccatcctgaccactgtgtgggtccctcctg 11278
LOC102725072.start-FAM230F.start.18846286-18865042.NEW.ref atccctgccttggctaaagcaccgtccctgccatcctgaccactgtgtgggtccctcctg 11278
                                                           ********************************** *************************

BCRP2.21103016-21122286.NCBI.ref.revcompl                  ggctttgagcagctcatctgactcctcccaagagctgtgcatggttctgtgtctgcagag 5341
LOC102725072.has.POM121L1                                  ggctttgagcagctcatctgactcctcccaagagctgtgcatggttctgtgtctgcagag 11338
LOC102725072.start-FAM230F.start.18846286-18865042.NEW.ref ggctttgagcagctcatctgactcctcccaagagctgtgcatggttctgtgtctgcagag 11338
                                                           ************************************************************

BCRP2.21103016-21122286.NCBI.ref.revcompl                  ttgataggggtgcgtgggcattcccattcctctcccctgcttggcctgatgtgatggcca 5401
LOC102725072.has.POM121L1                                  ttgataggggtgcgtgggcattcccattcctctcccctgcttggcctgatgtgatggcca 11398
LOC102725072.start-FAM230F.start.18846286-18865042.NEW.ref ttgataggggtgcgtgggcattcccattcctctcccctgcttggcctgatgtgatggcca 11398
                                                           ************************************************************

BCRP2.21103016-21122286.NCBI.ref.revcompl                  ggaggaggccagcatggcaggacacagcgcctgcgtggggattgggtggctctgccctgt 5461
LOC102725072.has.POM121L1                                  ggaggaggccagcatggcaggacacagcgcctgcgtggggattgggtggctctgccctgt 11458
LOC102725072.start-FAM230F.start.18846286-18865042.NEW.ref ggaggaggccagcatggcaggacacagcgcctgcgtggggattgggtggctctgccctgt 11458
                                                           ************************************************************

BCRP2.21103016-21122286.NCBI.ref.revcompl                  acatagcaaccacccctgcaccagtgtcttctgatagcaggaaggccgtgggagaatctg 5521
LOC102725072.has.POM121L1                                  acatagcaaccacccctgcaccagtgtcatctgatagcaggaaggccgtgggagaatctg 11518
LOC102725072.start-FAM230F.start.18846286-18865042.NEW.ref acatagcaaccacccctgcaccagtgtcatctgatagcaggaaggccgtgggagaatctg 11518
                                                           **************************** *******************************

BCRP2.21103016-21122286.NCBI.ref.revcompl                  attggtttcagtgtttgaaccggtgtcttcctttggacccaattggccattggtgcttac 5581
LOC102725072.has.POM121L1                                  attggtttcagtgtttgaaccggtgtcttcctttggacccaattggccattggtgcttac 11578
LOC102725072.start-FAM230F.start.18846286-18865042.NEW.ref attggtttcagtgtttgaaccggtgtcttcctttggacccaattggccattggtgcttac 11578
                                                           ************************************************************

BCRP2.21103016-21122286.NCBI.ref.revcompl                  atcctcaccacaggccaggttcattctgggccctcagagggagctgaaactaccacaggg 5641
LOC102725072.has.POM121L1                                  atcctcaccacaggccaggttcattctgggccctcagagggagctgaaactaccacaggg 11638
LOC102725072.start-FAM230F.start.18846286-18865042.NEW.ref atcctcaccacaggccaggttcattctgggccctcagagggagctgaaactaccacaggg 11638
                                                           ************************************************************

BCRP2.21103016-21122286.NCBI.ref.revcompl                  ccctcccagggatgctgggcattctaggggtcctggtcagggtgggtggtgtgtgctgca 5701
LOC102725072.has.POM121L1                                  ccctcccagggatgctgggcattctaggggtcctggtcagggtgggtggtctgtgctgca 11698
LOC102725072.start-FAM230F.start.18846286-18865042.NEW.ref ccctcccagggatgctgggcattctaggggtcctggtcagggtgggtggtctgtgctgca 11698
                                                           ************************************************** *********

BCRP2.21103016-21122286.NCBI.ref.revcompl                  aagaaggatctgcaggcacaaaatcctgttgctttgaagatgctgggaaggaccctctgg 5761
LOC102725072.has.POM121L1                                  aagaagggtctgcaggcacaaaatcctgttgctttgaagatgctgggaaggaccctctgg 11758
LOC102725072.start-FAM230F.start.18846286-18865042.NEW.ref aagaagggtctgcaggcacaaaatcctgttgctttgaagatgctgggaaggaccctctgg 11758
                                                           ******* ****************************************************

BCRP2.21103016-21122286.NCBI.ref.revcompl                  ggtctcagtgccctcccctggcatttgaggcaggtccgggtccttcaaagcctgtgaggg 5821
LOC102725072.has.POM121L1                                  ggtctcagtgccctcccctggcatttgaggcaggtccgggtccttcaaagcctgtgaggg 11818
LOC102725072.start-FAM230F.start.18846286-18865042.NEW.ref ggtctcagtgccctcccctggcatttgaggcaggtccgggtccttcaaagcctgtgaggg 11818
                                                           ************************************************************

BCRP2.21103016-21122286.NCBI.ref.revcompl                  ttggtgagatggaggcggagaggctgcagccccggcctgcgctgaatttcatcagtgccc 5881
LOC102725072.has.POM121L1                                  ttggtgagatggaggcggagaggctgcagccccggcctgcgctgaatttcatcagtgccc 11878
LOC102725072.start-FAM230F.start.18846286-18865042.NEW.ref ttggtgagatggaggcggagaggctgcagccccggcctgcgctgaatttcatcagtgccc 11878
                                                           ************************************************************
```

```
BCRP2.21103016-21122286.NCBI.ref.revcompl                    tctgcccaccacatcctcatacagggcagtggacagaccgcactgagtcctgggcttcca 5941
LOC102725072.has.POM121L1                                    tctgcccaccacatcctcatacagggcagtggacagaccgcactgagtcctgggcttcca 11938
LOC102725072.start-FAM230F.start.18846286-18865042.NEW.ref   tctgcccaccacatcctcatacagggcagtggacagaccgcactgagtcctgggcttcca 11938
                                                             ************************************************************

BCRP2.21103016-21122286.NCBI.ref.revcompl                    cctcctgtccaaccccaaggcaggaaggccaaggccccgcagaagcccctggtccactgc 6001
LOC102725072.has.POM121L1                                    cctcctgtccaaccccaaggcaggaaggccaaggccccgcagaagcccctggtccactgc 11998
LOC102725072.start-FAM230F.start.18846286-18865042.NEW.ref   cctcctgtccaaccccaaggcaggaaggccaaggccccgcagaagcccctggtccactgc 11998
                                                             ************************************************************

BCRP2.21103016-21122286.NCBI.ref.revcompl                    accaagtggcacgagtgggtacgatggtgtaaaaactggcttctatagaagctgtttgta 6061
LOC102725072.has.POM121L1                                    accaagtggcacgagtgggtacgatggtgtaaaaactggcttctatagaagctgtttgta 12058
LOC102725072.start-FAM230F.start.18846286-18865042.NEW.ref   accaagtggcacgagtgggtacgatggtgtaaaaactggcttctatagaagctgtttgta 12058
                                                             ************************************************************

BCRP2.21103016-21122286.NCBI.ref.revcompl                    caactcttgttttctctttttttaaaaataataaaacagtaaatgaagaaaagacacagag 6121
LOC102725072.has.POM121L1                                    caactcttgttttctctttttttaaaaataataaaacagtaaatgaagaaaagacacagag 12118
LOC102725072.start-FAM230F.start.18846286-18865042.NEW.ref   caactcttgttttctctttttttaaaaataataaaacagtaaatgaagaaaagacacagag 12118
                                                             ************************************************************

BCRP2.21103016-21122286.NCBI.ref.revcompl                    aaggatgtgacatgcctgggccatggagcactctgagatctcatcgtggacaccactgcc 6181
LOC102725072.has.POM121L1                                    aaggatgtgacatgcctgggccgtggagcactctgagatctcatcgcggacaccactgcc 12178
LOC102725072.start-FAM230F.start.18846286-18865042.NEW.ref   aaggatgtgacatgcctgggccgtggagcactctgagatctcatcgcggacaccactgcc 12178
                                                             ********************** *********************** *************

BCRP2.21103016-21122286.NCBI.ref.revcompl                    cacacctccatcccgtcctgcgcaggccgacactcactgacgttgaagcctgccttcagt 6241
LOC102725072.has.POM121L1                                    cacacctccatcccgtcctgcgcaggccgacactcactgacgttgaaggctgccttcagt 12238
LOC102725072.start-FAM230F.start.18846286-18865042.NEW.ref   cacacctccatcccgtcctgcgcaggccgacactcactgacgttgaaggctgccttcagt 12238
                                                             ************************************************ ***********

BCRP2.21103016-21122286.NCBI.ref.revcompl                    gcctggatgtctgcggccaccccagacatgcggtagatgcccacctcctccatgcctcgg 6301
LOC102725072.has.POM121L1                                    gcctggatgtctgcggccaccccagacatgcggtagatgcccacctcctccatgcctcgg 12298
LOC102725072.start-FAM230F.start.18846286-18865042.NEW.ref   gcctggatgtctgcggccaccccagacatgcggtagatgcccacctcctccatgcctcgg 12298
                                                             ************************************************************

BCRP2.21103016-21122286.NCBI.ref.revcompl                    cgctcgatctcctccacgcactgacgcacgatgtagggcaccttggacctctctctcctg 6361
LOC102725072.has.POM121L1                                    cgctcgatctcctccacgcactggcgcacgatgtagggcaccttggacctctctctcctg 12358
LOC102725072.start-FAM230F.start.18846286-18865042.NEW.ref   cgctcgatctcctccacgcactggcgcacgatgtagggcaccttggacctctctctcctg 12358
                                                             *********************** ************************************

BCRP2.21103016-21122286.NCBI.ref.revcompl                    cgggaggagggaatgttctcagtgtcctaacagccctgcttgggccataacacaggagac 6421
LOC102725072.has.POM121L1                                    cgggaggagggaatgttctcagtgtcctaacagccctgcttgggccataacacaggagac 12418
LOC102725072.start-FAM230F.start.18846286-18865042.NEW.ref   cgggaggagggaatgttctcagtgtcctaacagccctgcttgggccataacacaggagac 12418
                                                             ************************************************************

BCRP2.21103016-21122286.NCBI.ref.revcompl                    ctgctccctatctgcgcacccggaggtggggtgaggacggtgacgaaggtacccaggtct 6481
LOC102725072.has.POM121L1                                    ctgctccctatgtgcacacccggaggtggggtgaggacggtgatgaaggtacccaggtct 12478
LOC102725072.start-FAM230F.start.18846286-18865042.NEW.ref   ctgctccctatgtgcacacccggaggtggggtgaggacggtgatgaaggtacccaggtct 12478
                                                             *********** *** *************************** ****************

BCRP2.21103016-21122286.NCBI.ref.revcompl                    ggggctgcacacagagccttctgcatgcctgtcctccctctgcaagctctgtcctcattg 6541
LOC102725072.has.POM121L1                                    ggggctgcacacagagccttctgcatgcctgtcctccctctgcaagctctgtcctcattg 12538
LOC102725072.start-FAM230F.start.18846286-18865042.NEW.ref   ggggctgcacacagagccttctgcatgcctgtcctccctctgcaagctctgtcctcattg 12538
                                                             ************************************************************

BCRP2.21103016-21122286.NCBI.ref.revcompl                    catgtactttctcaggaacctttcaagcggccagaacccctgcgaatcacacatgacctt 6601
LOC102725072.has.POM121L1                                    catgtgctttctcaggaacctttcaagcggccagaacccctgcaaatcacacatgacctt 12598
LOC102725072.start-FAM230F.start.18846286-18865042.NEW.ref   catgtgctttctcaggaacctttcaagcggccagaacccctgcaaatcacacatgacctt 12598
                                                             ***** ************************************* ****************

BCRP2.21103016-21122286.NCBI.ref.revcompl                    tgtgggaaggtcaggaggcctgtctaagtcaagtcagcacgggaagggcatctgacagat 6661
LOC102725072.has.POM121L1                                    tgtgggaaggtcaggaggcctgtctaagtcaagtcagcacgggaagggcatctgacagat 12658
LOC102725072.start-FAM230F.start.18846286-18865042.NEW.ref   tgtgggaaggtcaggaggcctgtctaagtcaagtcagcacgggaagggcatctgacagat 12658
                                                             ************************************************************

BCRP2.21103016-21122286.NCBI.ref.revcompl                    tccaggcctggggttagcagcctgtgcccccggctgggaggtcagacccggtgttggtcc 6721
LOC102725072.has.POM121L1                                    tccaggcctggggtgagcagcctgtgcccccggctgggaggtcagacccggtgttggtcc 12718
LOC102725072.start-FAM230F.start.18846286-18865042.NEW.ref   tccaggcctggggtgagcagcctgtgcccccggctgggaggtcagacccggtgttggtcc 12718
                                                             ************** *********************************************

BCRP2.21103016-21122286.NCBI.ref.revcompl                    tgccacccacgtgctgtgtgagaggagaatccctgacccctgccctgggccttaacacac 6781
LOC102725072.has.POM121L1                                    tgccacccacgtgctgtgtgagaggagaatccgtgacccctgccctgggccttaacacac 12778
LOC102725072.start-FAM230F.start.18846286-18865042.NEW.ref   tgccacccacgtgctgtgtgagaggagaatccgtgacccctgccctgggccttaacacac 12778
                                                             ******************************** ***************************

BCRP2.21103016-21122286.NCBI.ref.revcompl                    atccgacgaatgaatgaagggttgcctcagcaccggtgctccaagtcctgcgatgctaag 6841
LOC102725072.has.POM121L1                                    atccgacgaatgaatgaagggttgcctcagcaccggtgctccaagtcctgcgatgctaag 12838
LOC102725072.start-FAM230F.start.18846286-18865042.NEW.ref   atccgacgaatgaatgaagggttgcctcagcaccggtgctccaagtcctgcgatgctaag 12838
                                                             ************************************************************

BCRP2.21103016-21122286.NCBI.ref.revcompl                    tgcttttctcctctgagtcttagcaatggacaattccaatacctccacacaggacactag 6901
LOC102725072.has.POM121L1                                    tgcttttctcctctgagtcttagcaatggacaattccaatacctccacacaggacactag 12898
LOC102725072.start-FAM230F.start.18846286-18865042.NEW.ref   tgcttttctcctctgagtcttagcaatggacaattccaatacctccacacaggacactag 12898
                                                             ************************************************************

BCRP2.21103016-21122286.NCBI.ref.revcompl                    agtaagaatccttcacagttagaacgcagtgctgtgcggaggccttaacttgagttctgt 6961
LOC102725072.has.POM121L1                                    agtaagaatccttcacagttagaacgcagtgctgtgcggaggccttaacttgagttctgt 12958
LOC102725072.start-FAM230F.start.18846286-18865042.NEW.ref   agtaagaatccttcacagttagaacgcagtgctgtgcggaggccttaacttgagttctgt 12958
                                                             ************************************************************

BCRP2.21103016-21122286.NCBI.ref.revcompl                    tttgcacctggatttaccagcacatcaaagctgcttcgcaagccccctcatcagcagggc 7021
LOC102725072.has.POM121L1                                    tttgcacctggatttaccagcacatcaaagctgcttcgcaagccccctcatcagcagggc 13018
LOC102725072.start-FAM230F.start.18846286-18865042.NEW.ref   tttgcacctggatttaccagcacatcaaagctgcttcgcaagccccctcatcagcagggc 13018
                                                             ************************************************************

BCRP2.21103016-21122286.NCBI.ref.revcompl                    ttatgtgggggagctgctgatggagtcctcgctgctcatgcccacagccctcccagagtg 7081
LOC102725072.has.POM121L1                                    tcatgtgggggagctgctgatggagtcctcgctgctcatgcccacagccctcccagagtg 13078
LOC102725072.start-FAM230F.start.18846286-18865042.NEW.ref   tcatgtgggggagctgctgatggagtcctcgctgctcatgcccacagccctcccagagtg 13078
                                                             * **********************************************************

BCRP2.21103016-21122286.NCBI.ref.revcompl                    ctatgcgagtggctgccgtgcagttgggggtggggcgtggtgtttagacacagataggag 7141
LOC102725072.has.POM121L1                                    ctatgcgagtggctgccgtgcagttgggggtggggcgtggtgtttagacacagataggag 13138
LOC102725072.start-FAM230F.start.18846286-18865042.NEW.ref   ctatgcgagtggctgccgtgcagttgggggtggggcgtggtgtttagacacagataggag 13138
                                                             ************************************************************

BCRP2.21103016-21122286.NCBI.ref.revcompl                    tccagggtatgactgatggaggccccggcccacgtgaccagcaaggtcagaggcccagcc 7201
LOC102725072.has.POM121L1                                    tccagggtatgactgatggaggccccggcccacgtgaccagcaaggtcagaggcccagcc 13198
LOC102725072.start-FAM230F.start.18846286-18865042.NEW.ref   tccagggtatgactgatggaggccccggcccacgtgaccagcaaggtcagaggcccagcc 13198
                                                             ************************************************************

BCRP2.21103016-21122286.NCBI.ref.revcompl                    agattccatcctggggaagcaaatgaattctcagaggaagtggtctgtgtctgtatgaac 7261
LOC102725072.has.POM121L1                                    agattccatcctgaggaagcaaatgaattctcagaggaagtggtctgtgtctgcatgaac 13258
LOC102725072.start-FAM230F.start.18846286-18865042.NEW.ref   agattccatcctgaggaagcaaatgaattctcagaggaagtggtctgtgtctgcatgaac 13258
                                                             ************* *************************************** ******

BCRP2.21103016-21122286.NCBI.ref.revcompl                    tgctctcaaaccaacaaataggcttctcttggcaactgactcgtgacaaagggttcaaga 7321
LOC102725072.has.POM121L1                                    tgctctcaaaccaacaaataggcttctcttggcaactgactcgtgacaaagggttcaaga 13318
LOC102725072.start-FAM230F.start.18846286-18865042.NEW.ref   tgctctcaaaccaacaaataggcttctcttggcaactgactcgtgacaaagggttcaaga 13318
                                                             ************************************************************

BCRP2.21103016-21122286.NCBI.ref.revcompl                    ttgtttgaaaaaaaaaaa-aaagggggggaacagggaggcagtaggtcctggaaaagtaa 7380
LOC102725072.has.POM121L1                                    ttgtttgaaaaaaaaaaaagggggggggggacagggaggcagtaggtcctggaaaagtaa 13378
LOC102725072.start-FAM230F.start.18846286-18865042.NEW.ref   ttgtttgaaaaaaaaaaaagggggggggggacagggaggcagtaggtcctggaaaagtaa 13378
                                                             ******************    ******* ******************************

BCRP2.21103016-21122286.NCBI.ref.revcompl                    attctttattttacaataagaaagtgattacatatttta-ttttttttacaatggtggaa 7439
LOC102725072.has.POM121L1                                    attctttattttacaataagaaagtgattacatattttattttttttttacaatggtggaa 13438
LOC102725072.start-FAM230F.start.18846286-18865042.NEW.ref   attctttattttacaataagaaagtgattacatattttattttttttttacaatggtggaa 13438
                                                             *************************************** ********************

BCRP2.21103016-21122286.NCBI.ref.revcompl                    aattagaagtgattgtgaaaatgatgtctacccgccttgctgatgagtaggatgtgattt 7499
LOC102725072.has.POM121L1                                    aattagaagtgattgtgaaaatgatgtctacccgccttgctgatgagtaggatgtgattt 13498
LOC102725072.start-FAM230F.start.18846286-18865042.NEW.ref   aattagaagtgattgtgaaaatgatgtctacccgccttgctgatgagtaggatgtgattt 13498
                                                             ************************************************************

BCRP2.21103016-21122286.NCBI.ref.revcompl                    ggctctttaggaaactgaatttgcagaacttaagaatattgatttataaagggcatggcc 7559
LOC102725072.has.POM121L1                                    ggctctttaggaaactgaatttgcagaacttaagaatattgatttataaagggcatggcc 13558
LOC102725072.start-FAM230F.start.18846286-18865042.NEW.ref   ggctctttaggaaactgaatttgcagaacttaagaatattgatttataaagggcatggcc 13558
                                                             ************************************************************

BCRP2.21103016-21122286.NCBI.ref.revcompl                    attgacccagtccatcttatgcaaatctggatgccataaataatatttaaaaatgaaagt 7619
LOC102725072.has.POM121L1                                    attgacccagtccatcttatgcaaatctggatgccataaataatatttaaaaatgaaagt 13618
LOC102725072.start-FAM230F.start.18846286-18865042.NEW.ref   attgacccagtccatcttatgcaaatctggatgccataaataatatttaaaaatgaaagt 13618
                                                             ************************************************************
```

```
BCRP2.21103016-21122286.NCBI.ref.revcompl                   attggggtggaggttgcagtgagcggagatcgccccactacactccagcctggtcaacag 7679
LOC102725072.has.POM121L1                                   attggggtggaggttgcagtgagcggagatcgccccactacactccagcctggtcaacag 13678
LOC102725072.start-FAM230F.start.18846286-18865042.NEW.ref  attggggtggaggttgcagtgagcggagatcgccccactacactccagcctggtcaacag 13678
                                                            ************************************************************

BCRP2.21103016-21122286.NCBI.ref.revcompl                   agtgagactctgtctcaaaacaaaaaaaaaaaaaaaaaaaggaagaagtcattcccaaca 7739
LOC102725072.has.POM121L1                                   agtgagactctgtctcaaaacaaaaaaaaaaaaaaaaaaa-gaagaagtcattcccaaca 13737
LOC102725072.start-FAM230F.start.18846286-18865042.NEW.ref  agtgagactctgtctcaaaacaaaaaaaaaaaaaaaaaaa-gaagaagtcattcccaaca 13737
                                                            **************************************** *******************

BCRP2.21103016-21122286.NCBI.ref.revcompl                   ttgctcatcaaattatgaccataaatttccaggatcagactaacggctaaagagactgat 7799
LOC102725072.has.POM121L1                                   ttgctcatcaaattatgaccataaatttccaggatcagactaacggctaaagagactgat 13797
LOC102725072.start-FAM230F.start.18846286-18865042.NEW.ref  ttgctcatcaaattatgaccataaatttccaggatcagactaacggctaaagagactgat 13797
                                                            ************************************************************

BCRP2.21103016-21122286.NCBI.ref.revcompl                   gcatcaacaccaggcagagaataaagcagattttttttgttttgttttggagagcctcta 7859
LOC102725072.has.POM121L1                                   gcatcaacaccaggcagagaataaagcagattttttttgttttgttttggagagcctcta 13857
LOC102725072.start-FAM230F.start.18846286-18865042.NEW.ref  gcatcaacaccaggcagagaataaagcagattttttttgttttgttttggagagcctcta 13857
                                                            ************************************************************

BCRP2.21103016-21122286.NCBI.ref.revcompl                   ggaacttgaaaaatacatatgccacactcttaagacccggtggttcttaatcagggatgt 7919
LOC102725072.has.POM121L1                                   ggaacttgaaaaatacatatgccacactcttaagacccggtggttcttaatcagggatgt 13917
LOC102725072.start-FAM230F.start.18846286-18865042.NEW.ref  ggaacttgaaaaatacatatgccacactcttaagacccggtggttcttaatcagggatgt 13917
                                                            ************************************************************

BCRP2.21103016-21122286.NCBI.ref.revcompl                   tcattaaaatgctggaaaactgtaaagacttccaggtcccatccaaggagattttgcttc 7979
LOC102725072.has.POM121L1                                   tcattaaaatgctggaaaactctaaagatttccaggtcccatccaaggagattttgcttc 13977
LOC102725072.start-FAM230F.start.18846286-18865042.NEW.ref  tcattaaaatgctggaaaactctaaagatttccaggtcccatccaaggagattttgcttc 13977
                                                            ********************* ****** *******************************

BCRP2.21103016-21122286.NCBI.ref.revcompl                   tgattgactggctagtggcctggccattggtattttgaaaaatccctccaagtgattctt 8039
LOC102725072.has.POM121L1                                   tgattgactggctagtggcctggccattggtattttgaaaaatccctccaagtgattctt 14037
LOC102725072.start-FAM230F.start.18846286-18865042.NEW.ref  tgattgactggctagtggcctggccattggtattttgaaaaatccctccaagtgattctt 14037
                                                            ************************************************************

BCRP2.21103016-21122286.NCBI.ref.revcompl                   ttacatcccagctagaaaaccctaaattaaaggtgaaaaaccagacaccaagtggcattt 8099
LOC102725072.has.POM121L1                                   ttacatcccagctagaaaaccctaaattaaaggtgaaaaaccagacaccaagtggcattt 14097
LOC102725072.start-FAM230F.start.18846286-18865042.NEW.ref  ttacatcccagctagaaaaccctaaattaaaggtgaaaaaccagacaccaagtggcattt 14097
                                                            ************************************************************

BCRP2.21103016-21122286.NCBI.ref.revcompl                   aaataaatgtcaactttaactccacaaagcatctggttgcacgtggacagaaagagaagg 8159
LOC102725072.has.POM121L1                                   aaataaatgtcaactttaactccacaaagcatctggttgcatgtggacagaaagagaagg 14157
LOC102725072.start-FAM230F.start.18846286-18865042.NEW.ref  aaataaatgtcaactttaactccacaaagcatctggttgcatgtggacagaaagagaagg 14157
                                                            ***************************************** ******************

BCRP2.21103016-21122286.NCBI.ref.revcompl                   aaagagggccctatatctggataacttggaaatgtgctcccccctagcaagatatctacca 8219
LOC102725072.has.POM121L1                                   aaagagggccctatatctggataacttggaaatgtgctcccccctagcaagatatctacca 14217
LOC102725072.start-FAM230F.start.18846286-18865042.NEW.ref  aaagagggccctatatctggataacttggaaatgtgctcccccctagcaagatatctacca 14217
                                                            ************************************************************

BCRP2.21103016-21122286.NCBI.ref.revcompl                   aaattaaaaccatatttgaggatgctggcactgcgagcaatatataaatgatgcatgtaa 8279
LOC102725072.has.POM121L1                                   aaattaaaaccatatttgaggatgctggcactgtgagcaatatataaatgatgcatgtaa 14277
LOC102725072.start-FAM230F.start.18846286-18865042.NEW.ref  aaattaaaaccatatttgaggatgctggcactgtgagcaatatataaatgatgcatgtaa 14277
                                                            ********************************* **************************

BCRP2.21103016-21122286.NCBI.ref.revcompl                   catcatttaatatgatcttatttttaaaaatgagtagagtggtgttttctagctgttagt 8339
LOC102725072.has.POM121L1                                   catcatttaatatgatcttatttttaaaaatgagtagagtggtgttttctagctgttagt 14337
LOC102725072.start-FAM230F.start.18846286-18865042.NEW.ref  catcatttaatatgatcttatttttaaaaatgagtagagtggtgttttctagctgttagt 14337
                                                            ************************************************************

BCRP2.21103016-21122286.NCBI.ref.revcompl                   gtttccaaatatcaatgtagaaattagccttctgcagctgcagaggcaattcagtttgca 8399
LOC102725072.has.POM121L1                                   gtttccaaatatcaatgtagaaattagccttctgcagctgcagaggcaattcagtttgca 14397
LOC102725072.start-FAM230F.start.18846286-18865042.NEW.ref  gtttccaaatatcaatgtagaaattagccttctgcagctgcagaggcaattcagtttgca 14397
                                                            ************************************************************

BCRP2.21103016-21122286.NCBI.ref.revcompl                   gcttgcttgcatgtggcctagagccacccagcctgatatgtactaattttt--tgtttaa 8457
LOC102725072.has.POM121L1                                   gcttgcttgcatgtggcctagagccacccagcctgatatgtactaattttttatgtttaa 14457
LOC102725072.start-FAM230F.start.18846286-18865042.NEW.ref  gcttgcttgcatgtggcctagagccacccagcctgatatgtactaattttttatgtttaa 14457
                                                            ***************************************************  *******

BCRP2.21103016-21122286.NCBI.ref.revcompl                   cttgccagagtagaaactcagtttctgggccaggcatagtggctcatgcctgtaatccca 8517
LOC102725072.has.POM121L1                                   cttgccagagtagaaactcagtttctgggcgaggcatagtggctcatgcctgtaatccca 14517
LOC102725072.start-FAM230F.start.18846286-18865042.NEW.ref  cttgccagagtagaaactcagtttctgggcgaggcatagtggctcatgcctgtaatccca 14517
                                                            ****************************** *****************************

BCRP2.21103016-21122286.NCBI.ref.revcompl                   gaactttgggaggccaaggcaagcagatcacaaggtcgggagttcaagaccagcctggct 8577
LOC102725072.has.POM121L1                                   gaactttgggaggccaaggcaagcagatcacaaggtcgggagttcaagaccagcctggct 14577
LOC102725072.start-FAM230F.start.18846286-18865042.NEW.ref  gaactttgggaggccaaggcaagcagatcacaaggtcgggagttcaagaccagcctggct 14577
                                                            ************************************************************

BCRP2.21103016-21122286.NCBI.ref.revcompl                   gacatagtgaaaccctatctttgctaaaaatacaaaaattagcccggcatgctggcaggc 8637
LOC102725072.has.POM121L1                                   gacatagtgaaaccctatctttgctaaaaatacaaaaattagcccggcatggtggcaggc 14637
LOC102725072.start-FAM230F.start.18846286-18865042.NEW.ref  gacatagtgaaaccctatctttgctaaaaatacaaaaattagcccggcatggtggcaggc 14637
                                                            *************************************************** ********

BCRP2.21103016-21122286.NCBI.ref.revcompl                   acctgtagtcccagctacttgggaggctgaggcaggagaatcacttgaacctgggaggca 8697
LOC102725072.has.POM121L1                                   acctgtagtcccagctacttgggaggctgaggcaggagaatcacttgaacctgggaggca 14697
LOC102725072.start-FAM230F.start.18846286-18865042.NEW.ref  acctgtagtcccagctacttgggaggctgaggcaggagaatcacttgaacctgggaggca 14697
                                                            ************************************************************

BCRP2.21103016-21122286.NCBI.ref.revcompl                   gaggttgtggtgaactgagatcatgcccctgcactccagcctgggcaacagagtgagact 8757
LOC102725072.has.POM121L1                                   gaggttgtggtgaactgagatcatgcccctgcactccagcctgggcaacagagtgagact 14757
LOC102725072.start-FAM230F.start.18846286-18865042.NEW.ref  gaggttgtggtgaactgagatcatgcccctgcactccagcctgggcaacagagtgagact 14757
                                                            ************************************************************

BCRP2.21103016-21122286.NCBI.ref.revcompl                   ccatctc-aaaaaaaaaaaaaagaaactcagtttctggttacatctgatctttatttttt 8816
LOC102725072.has.POM121L1                                   ccatctcaaaaaaaaaaaaaaa-------------------------------------- 14779
LOC102725072.start-FAM230F.start.18846286-18865042.NEW.ref  ccatctcaaaaaaaaaaaaaaagaaactcagtttctggttacatctgatctttatttttt 14817
                                                            ******* **************
```

BCRP2 sequence present in *linc-UR-B1, beyond the 3' end of LOC102725072*

```
BCRP2.21103016-21122286.NCBI.ref.revcompl                   atatatcatctaagctataaagttatattccctatttgtgatcttaaaagaaggactcca 8876
LOC102725072.has.POM121L1                                   ------------------------------------------------------------ 14779
LOC102725072.start-FAM230F.start.18846286-18865042.NEW.ref  atatatcatctaagctataaagttatattccctatttgtgatcttaaaagaaggactcca 14877

BCRP2.21103016-21122286.NCBI.ref.revcompl                   ggaaagtgttcaaatattcatatatctaaactggaacatatgtttatattttaaaagta 8936
LOC102725072.has.POM121L1                                   ------------------------------------------------------------ 14779
LOC102725072.start-FAM230F.start.18846286-18865042.NEW.ref  ggaaagtgttcaaatattcatatatctaaactggaacatatgtttatattttaaaagta 14937

BCRP2.21103016-21122286.NCBI.ref.revcompl                   gcctgagaggttggcaactaaagtcatatgttgaatgatcatttctcaagagtttcattt 8996
LOC102725072.has.POM121L1                                   ------------------------------------------------------------ 14779
LOC102725072.start-FAM230F.start.18846286-18865042.NEW.ref  gcctgagaggttggcaactaaagtcatatgttgaatgatcatttctcaagagtttcattt 14997

BCRP2.21103016-21122286.NCBI.ref.revcompl                   tatggtctttctcttgttctgtaaaatgtgggcatggatagatataaagtgcctggtgtc 9056
LOC102725072.has.POM121L1                                   ------------------------------------------------------------ 14779
LOC102725072.start-FAM230F.start.18846286-18865042.NEW.ref  tatggtctttctcttgttctgtaaaatgtgggcatggatagatataaagtgcctggtgtc 15057

BCRP2.21103016-21122286.NCBI.ref.revcompl                   catgcttttgtgaaatcccttcctcttccatgtgaatgggacctgtgactttcttctaac 9116
LOC102725072.has.POM121L1                                   ------------------------------------------------------------ 14779
LOC102725072.start-FAM230F.start.18846286-18865042.NEW.ref  catgcttttgtgaaatcccttcctcttccatgtgaatgggacctgtgactttcttctaac 15117

BCRP2.21103016-21122286.NCBI.ref.revcompl                   ccagagaacacagcaaaaatgatgtgatttatctgagtccattgatgacattgattacga 9176
LOC102725072.has.POM121L1                                   ------------------------------------------------------------ 14779
LOC102725072.start-FAM230F.start.18846286-18865042.NEW.ref  ccagagaacacagcaaaaatgatgtgatttatctgagtccattgatgacattgattacga 15177

BCRP2.21103016-21122286.NCBI.ref.revcompl                   cttcccttcaccacattatttaggactgcgtcgtaggagactgggacacatatccacttt 9236
LOC102725072.has.POM121L1                                   ------------------------------------------------------------ 14779
LOC102725072.start-FAM230F.start.18846286-18865042.NEW.ref  cttcccttcaccacattatttaggactgcgtcgtaggagactgggacacatatccacttt 15237

BCRP2.21103016-21122286.NCBI.ref.revcompl                   gctggcttgatgaagtaaactgctaagttgaggaagcccacatggcaaggaactgtgggc 9296
LOC102725072.has.POM121L1                                   ------------------------------------------------------------ 14779
LOC102725072.start-FAM230F.start.18846286-18865042.NEW.ref  gctggcttgatgaagtaaactgctaagttgaggaagcccacatggcaaggaactgtgggc 15297

BCRP2.21103016-21122286.NCBI.ref.revcompl                   agccttcagccaacaggcagcaaaaagctgagctccttggagctacagcctcaaggaagt 9356
LOC102725072.has.POM121L1                                   ------------------------------------------------------------ 14779
LOC102725072.start-FAM230F.start.18846286-18865042.NEW.ref  agccttcagccaacaggcagcaaaaagctgagctcctcggagctacagcctcaaggaagt 15357
```

```
BCRP2.21103016-21122286.NCBI.ref.revcompl                tacttctgctaacaacctgaaaaagcttggaagctagtttctctctagtggaatttttag 9416
LOC102725072.has.POM121L1                                ------------------------------------------------------------ 14779
LOC102725072.start-FAM230F.start.18846286-18865042.NEW.ref     tacttctgctaacaacctgaaaaagcttggaagctagtttctctctagtggaatttttag 15417

BCRP2.21103016-21122286.NCBI.ref.revcompl                ggaagagcatggcccaggcaagataaataatgtaagtggaaaaacctgtcaacaataaag 9476
LOC102725072.has.POM121L1                                ------------------------------------------------------------ 14779
LOC102725072.start-FAM230F.start.18846286-18865042.NEW.ref     ggaagagcatggcccaggcaagataaataatgtaagtggaaaaacctgtcaacaataaag 15477

BCRP2.21103016-21122286.NCBI.ref.revcompl                gttttaaaggaaaaaacaaaaaacatgtaatttagaaagtaactgccagg-aaaaaaaaa 9535
LOC102725072.has.POM121L1                                ------------------------------------------------------------ 14779
LOC102725072.start-FAM230F.start.18846286-18865042.NEW.ref     gttttaaaggaaaaaacaaaaaacatgtaatttagaaagtaactgccaggaaaaaaaaaa 15537

BCRP2.21103016-21122286.NCBI.ref.revcompl                aaagagactgggccagtggcttactacacctgtaatcccagcacttggggaggctgaggc 9595
LOC102725072.has.POM121L1                                ------------------------------------------------------------ 14779
LOC102725072.start-FAM230F.start.18846286-18865042.NEW.ref     aaagagactgggccagtggcttactacacctgtaatcccagcacttggggaggctgaggc 15597

BCRP2.21103016-21122286.NCBI.ref.revcompl                gggtgaatcacaaggtcaggagatcgagaccatcctggctaacacggtgaaaccccgtct 9655
LOC102725072.has.POM121L1                                ------------------------------------------------------------ 14779
LOC102725072.start-FAM230F.start.18846286-18865042.NEW.ref     gggtgaatcacaaggtcaggagatcgagaccatcctggctaacacggtgaaaccccgtct 15657

BCRP2.21103016-21122286.NCBI.ref.revcompl                ctactaaaaatacaaaaaaaaaaaaaaaaaattagccgggcgtggtggtgggcgcctgtgg 9715
LOC102725072.has.POM121L1                                ------------------------------------------------------------ 14779
LOC102725072.start-FAM230F.start.18846286-18865042.NEW.ref     ctactaaaaatac-aaaaaaaaaaaaaaaaattagccgggcgtggtggtgggcgcctgtgg 15716

BCRP2.21103016-21122286.NCBI.ref.revcompl                tcccagctactggggaggttgagacaggagaatggcgtgaaccccgggacgtggagcttg 9775
LOC102725072.has.POM121L1                                ------------------------------------------------------------ 14779
LOC102725072.start-FAM230F.start.18846286-18865042.NEW.ref     tcccagctactggggaggttgagacaggagaatggcgtgaaccccgggacgtggagcttg 15776

BCRP2.21103016-21122286.NCBI.ref.revcompl                cagtgagccgagatcatgccactgcactccagcctgggcaacagagcgagactccatctc 9835
LOC102725072.has.POM121L1                                ------------------------------------------------------------ 14779
LOC102725072.start-FAM230F.start.18846286-18865042.NEW.ref     cagtgagccgagatcatgccactgcactccagcctgggcaacagagcgagactccatctc 15836

BCRP2.21103016-21122286.NCBI.ref.revcompl                aaaataaataaataaataaaatataaggtctcaggaacgtaaagattgacatttactccc 9895
LOC102725072.has.POM121L1                                ------------------------------------------------------------ 14779
LOC102725072.start-FAM230F.start.18846286-18865042.NEW.ref     aaaataaataaataaataaaatataaggtctcaggaacgtaaagattgacatttactccc 15896

BCRP2.21103016-21122286.NCBI.ref.revcompl                aaactattaatatatgtccacccacctttcttgtagcaaaatcttaacttgacgtttgtt 9955
LOC102725072.has.POM121L1                                ------------------------------------------------------------ 14779
LOC102725072.start-FAM230F.start.18846286-18865042.NEW.ref     aaactattaatatatgtccacccacctttcttgtagcaaaatcttaacttgacgtttgtt 15956

BCRP2.21103016-21122286.NCBI.ref.revcompl                tcaatagttattaaatttaattataatgtcctagcccaaaatacagtagaggtaaacatc 10015
LOC102725072.has.POM121L1                                ------------------------------------------------------------ 14779
LOC102725072.start-FAM230F.start.18846286-18865042.NEW.ref     tcaatagttattaaatttaattataatgtcctagcccaaaatacagtagaggtaaacatc 16016

BCRP2.21103016-21122286.NCBI.ref.revcompl                caaggtactggcttgaggccactggccctgtatctataaaggagagggagaccatcaggg 10075
LOC102725072.has.POM121L1                                ------------------------------------------------------------ 14779
LOC102725072.start-FAM230F.start.18846286-18865042.NEW.ref     caaggtactggcttgaggccactggccctgtatctataaaggagagggagaccatcaggg 16076

BCRP2.21103016-21122286.NCBI.ref.revcompl                gagggagggtaagaaagggaggaagagggcagacaagttatcaaaaaacaacagtaggca 10135
LOC102725072.has.POM121L1                                ------------------------------------------------------------ 14779
LOC102725072.start-FAM230F.start.18846286-18865042.NEW.ref     gagggagggtaagaaagggaggaagagggcaaacaagttatcaaaaaacaacagtaggca 16136

BCRP2.21103016-21122286.NCBI.ref.revcompl                gggcactatggctcatgcttgtaatcctagcattttgggaagccaaggtgggcaaattgc 10195
LOC102725072.has.POM121L1                                ------------------------------------------------------------ 14779
LOC102725072.start-FAM230F.start.18846286-18865042.NEW.ref     gggcactatggctcatgcttgtaatcctagcattttgggaagccaaggtgggcaaattgc 16196

BCRP2.21103016-21122286.NCBI.ref.revcompl                ttgcactcaggagttcaagaccagcctggacaacatggcaaaaccccatctctacaaaaa 10255
LOC102725072.has.POM121L1                                ------------------------------------------------------------ 14779
LOC102725072.start-FAM230F.start.18846286-18865042.NEW.ref     ttgcactcaggagttcaagaccagcctggacaacatggcaaaaccccatctctacaaaaa 16256

BCRP2.21103016-21122286.NCBI.ref.revcompl                atcggccaggcctggtggtgtatgcttgtaattccagctacttgggaggctgaggtagga 10315
LOC102725072.has.POM121L1                                ------------------------------------------------------------ 14779
LOC102725072.start-FAM230F.start.18846286-18865042.NEW.ref     atcggccaggcctggtggtgtacgcttgtaattccagctacttgggaggctgaggtagga 16316

BCRP2.21103016-21122286.NCBI.ref.revcompl                ggatcacttgagcctggaaggcagaggttgcagtgagccgacatcatgccactgcacttc 10375
LOC102725072.has.POM121L1                                ------------------------------------------------------------ 14779
LOC102725072.start-FAM230F.start.18846286-18865042.NEW.ref     ggatcacttgagcctggaaggcagaggttgcagtgagccgacatcatgccactgcacttc 16376

BCRP2.21103016-21122286.NCBI.ref.revcompl                agcctgagtgacagagtaagaccctgtctcaaaaacaaacacatcagttattcatatttc 10435
LOC102725072.has.POM121L1                                ------------------------------------------------------------ 14779
LOC102725072.start-FAM230F.start.18846286-18865042.NEW.ref     agcctgagtgacagagtaagaccctgtctcaaaaacaaacacatcagttattcatatttc 16436

BCRP2.21103016-21122286.NCBI.ref.revcompl                agagtaaggacaaaacatttttaagtagctggcaaaggacatccctatatttcagagtaa 10495
LOC102725072.has.POM121L1                                ------------------------------------------------------------ 14779
LOC102725072.start-FAM230F.start.18846286-18865042.NEW.ref     agagtaaggacaaaacatttttaagtagctggcaaaggacatcactatatttcagagtaa 16496

BCRP2.21103016-21122286.NCBI.ref.revcompl                aacaaataggcaatgcttatcatttgacatatttttaaacattgtatctgaaaagtgaac 10555
LOC102725072.has.POM121L1                                ------------------------------------------------------------ 14779
LOC102725072.start-FAM230F.start.18846286-18865042.NEW.ref     aacaaataggaaatgcttatcatttgacatatttttaaacattgtatctgaaaagtgaac 16556

BCRP2.21103016-21122286.NCBI.ref.revcompl                aaagaaatgaatgtgcttatgattaaattgactttgttactttgtaaacttgtagcttta 10615
LOC102725072.has.POM121L1                                ------------------------------------------------------------ 14779
LOC102725072.start-FAM230F.start.18846286-18865042.NEW.ref     aaagaagtgaatgtgcttatgattaaattgactttgttactttgtaaacttgtagcttta 16616

BCRP2.21103016-21122286.NCBI.ref.revcompl                gacctgtctcttagcatcaccaagccttgatcttttcatctataaaatgggcatggtaat 10675
LOC102725072.has.POM121L1                                ------------------------------------------------------------ 14779
LOC102725072.start-FAM230F.start.18846286-18865042.NEW.ref     gacctgtctcttagcatcaccaagccttgatcttttcatctataaaatgggcatggtaat 16676

BCRP2.21103016-21122286.NCBI.ref.revcompl                gccagccttgctatgtttataggtcacttaggaatgaggtatgtatggtgttgaccatgg 10735
LOC102725072.has.POM121L1                                ------------------------------------------------------------ 14779
LOC102725072.start-FAM230F.start.18846286-18865042.NEW.ref     gccagccttgctatgtttataggtcacttaggaatgaggtatgtatggtgttgaccatgg 16736

BCRP2.21103016-21122286.NCBI.ref.revcompl                tttctggcaagtggcatatattcattataccatagctcttttcagaaagctaagtcacca 10795
LOC102725072.has.POM121L1                                ------------------------------------------------------------ 14779
LOC102725072.start-FAM230F.start.18846286-18865042.NEW.ref     tttctggcaagtggcatatattcattataccatagctcttttcagaaagctaagtcacca 16796

BCRP2.21103016-21122286.NCBI.ref.revcompl                cgtacatgtcaatgcaacctgctgaaaataggcatggaaaaactagaaaatctagaaaaa 10855
LOC102725072.has.POM121L1                                ------------------------------------------------------------ 14779
LOC102725072.start-FAM230F.start.18846286-18865042.NEW.ref     cgtacatgttaatgcaacctgctgaaaataggcatggaaaaactagaaaatctagaaaaa 16856
```

end of *BCRP2* sequence present in *linc-UR-B1*

```
BCRP2.21103016-21122286.NCBI.ref.revcompl                ttagaaaatcactgaagaggattttctttaaaaaaatacatactttagtgtttgagtcat 10915
LOC102725072.has.POM121L1                                ------------------------------------------------------------ 14779
LOC102725072.start-FAM230F.start.18846286-18865042.NEW.ref     ttagaaaatcactgaagaggattttctttaaaaaaatacatactttaa------------ 16904

BCRP2.21103016-21122286.NCBI.ref.revcompl                gaagtccttgcccgtgcctatgtcctaagtggtattgcctgggttttcttctagggtttt 10975
LOC102725072.has.POM121L1                                ------------------------------------------------------------ 14779
LOC102725072.start-FAM230F.start.18846286-18865042.NEW.ref     ------------------------------------------------------------ 16904

BCRP2.21103016-21122286.NCBI.ref.revcompl                tatggttttaggtctaacatttaagtctttaatccatcttgaattaatttttgtataagg 11035
LOC102725072.has.POM121L1                                ------------------------------------------------------------ 14779
LOC102725072.start-FAM230F.start.18846286-18865042.NEW.ref     ------------------------------------------------------------ 16904

BCRP2.21103016-21122286.NCBI.ref.revcompl                tgtaaggaagggatccagtttcagctttctaaatatggctagccagtttagttcatgtcc 11095
LOC102725072.has.POM121L1                                ------------------------------------------------------------ 14779
LOC102725072.start-FAM230F.start.18846286-18865042.NEW.ref     ------------------------------------------------------------ 16904
```

```
BCRP2.21103016-21122286.NCBI.ref.revcompl                       tttgtagcgacagggatgaagctggaaaccatcattctgagcaaactatcgcaaggacag 11155
LOC102725072.has.POM121L1                                       ------------------------------------------------------------ 14779
LOC102725072.start-FAM230F.start.18846286-18865042.NEW.ref      ------------------------------------------------------------ 16904

BCRP2.21103016-21122286.NCBI.ref.revcompl                       aaaaccaaacagcgcatgttctcactcataggtgggaattaaacaataagaacacgtgga 11215
LOC102725072.has.POM121L1                                       ------------------------------------------------------------ 14779
LOC102725072.start-FAM230F.start.18846286-18865042.NEW.ref      ------------------------------------------------------------ 16904

BCRP2.21103016-21122286.NCBI.ref.revcompl                       cacagggtggggaacatcacacaccggggcctgtcgtggggtgggggatagggggaggga 11275
LOC102725072.has.POM121L1                                       ------------------------------------------------------------ 14779
LOC102725072.start-FAM230F.start.18846286-18865042.NEW.ref      ------------------------------------------------------------ 16904

BCRP2.21103016-21122286.NCBI.ref.revcompl                       tagcattagaataaatacctaatgtaaatgatgagttaatgggtgcagcaaaccaacaca 11335
LOC102725072.has.POM121L1                                       ------------------------------------------------------------ 14779
LOC102725072.start-FAM230F.start.18846286-18865042.NEW.ref      ------------------------------------------------------------ 16904

BCRP2.21103016-21122286.NCBI.ref.revcompl                       gcacatgtatacatatgtaacaaatctgcacgttgtgcacatgtaccctaggacttaaaa 11395
LOC102725072.has.POM121L1                                       ------------------------------------------------------------ 14779
LOC102725072.start-FAM230F.start.18846286-18865042.NEW.ref      ------------------------------------------------------------ 16904

BCRP2.21103016-21122286.NCBI.ref.revcompl                       ggataataatatatatatacatacacacatacactttatccatgcatctgttgatagaca 11455
LOC102725072.has.POM121L1                                       ------------------------------------------------------------ 14779
LOC102725072.start-FAM230F.start.18846286-18865042.NEW.ref      ------------------------------------------------------------ 16904

BCRP2.21103016-21122286.NCBI.ref.revcompl                       cttaggttgttccctatcttggctgttgtgaataatgtgccataaatatgggggtgcagg 11515
LOC102725072.has.POM121L1                                       ------------------------------------------------------------ 14779
LOC102725072.start-FAM230F.start.18846286-18865042.NEW.ref      ------------------------------------------------------------ 16904

BCRP2.21103016-21122286.NCBI.ref.revcompl                       tacctctctgacatactgatttcaatttccttggatatagccagaagtaggattgctggg 11575
LOC102725072.has.POM121L1                                       ------------------------------------------------------------ 14779
LOC102725072.start-FAM230F.start.18846286-18865042.NEW.ref      ------------------------------------------------------------ 16904

BCRP2.21103016-21122286.NCBI.ref.revcompl                       tcatgtggtaatttttttttttttttttttgagacaaagtcttgctctgttgcccaggct 11635
LOC102725072.has.POM121L1                                       ------------------------------------------------------------ 14779
LOC102725072.start-FAM230F.start.18846286-18865042.NEW.ref      ------------------------------------------------------------ 16904

BCRP2.21103016-21122286.NCBI.ref.revcompl                       ggagtgcagtggcatgatctcggctcactgcagcctccacctcacgggctcaagcagtcc 11695
LOC102725072.has.POM121L1                                       ------------------------------------------------------------ 14779
LOC102725072.start-FAM230F.start.18846286-18865042.NEW.ref      ------------------------------------------------------------ 16904

BCRP2.21103016-21122286.NCBI.ref.revcompl                       tcccacctcagcctcccgaagtgctgggattacagtgtgagccactgcacctgacctcat 11755
LOC102725072.has.POM121L1                                       ------------------------------------------------------------ 14779
LOC102725072.start-FAM230F.start.18846286-18865042.NEW.ref      ------------------------------------------------------------ 16904

BCRP2.21103016-21122286.NCBI.ref.revcompl                       gtggtaattgtattttagttatttgattaattttgtgccgtttttcataatgcctgta 11815
LOC102725072.has.POM121L1                                       ------------------------------------------------------------ 14779
LOC102725072.start-FAM230F.start.18846286-18865042.NEW.ref      ------------------------------------------------------------ 16904

BCRP2.21103016-21122286.NCBI.ref.revcompl                       ctgatttacattcactccaacatgtactagggttccattttctccacatcctcttcaaca 11875
LOC102725072.has.POM121L1                                       ------------------------------------------------------------ 14779
LOC102725072.start-FAM230F.start.18846286-18865042.NEW.ref      ------------------------------------------------------------ 16904

BCRP2.21103016-21122286.NCBI.ref.revcompl                       cttgttacctttctttttttaatattaatcattctaacagatataaagtgatattgttgt 11935
LOC102725072.has.POM121L1                                       ------------------------------------------------------------ 14779
LOC102725072.start-FAM230F.start.18846286-18865042.NEW.ref      ------------------------------------------------------------ 16904

BCRP2.21103016-21122286.NCBI.ref.revcompl                       ggttttaatttgcatttccctaatgattagtgctgctgggcatttttttaaaagaaggaa 11995
LOC102725072.has.POM121L1                                       ------------------------------------------------------------ 14779
LOC102725072.start-FAM230F.start.18846286-18865042.NEW.ref      ------------------------------------------------------------ 16904

BCRP2.21103016-21122286.NCBI.ref.revcompl                       attatgtcatttgcaacaaaatgaataaacctggagaacattacactaagtgaaagaagc 12055
LOC102725072.has.POM121L1                                       ------------------------------------------------------------ 14779
LOC102725072.start-FAM230F.start.18846286-18865042.NEW.ref      ------------------------------------------------------------ 16904

BCRP2.21103016-21122286.NCBI.ref.revcompl                       cagacaggatagataagtacatgatctcatgtatatgtggaatctaaaaaagccaaattt 12115
LOC102725072.has.POM121L1                                       ------------------------------------------------------------ 14779
LOC102725072.start-FAM230F.start.18846286-18865042.NEW.ref      ------------------------------------------------------------ 16904

BCRP2.21103016-21122286.NCBI.ref.revcompl                       atagaaataacaggtagaaaaggggttatgagaggctggggtgtgttgggggggtacagca 12175
LOC102725072.has.POM121L1                                       ------------------------------------------------------------ 14779
LOC102725072.start-FAM230F.start.18846286-18865042.NEW.ref      ------------------------------------------------------------ 16904

BCRP2.21103016-21122286.NCBI.ref.revcompl                       gacagggagatgctgttcaaagggtacaaagttttcattaggaggaataaagttttgaga 12235
LOC102725072.has.POM121L1                                       ------------------------------------------------------------ 14779
LOC102725072.start-FAM230F.start.18846286-18865042.NEW.ref      ------------------------------------------------------------ 16904

BCRP2.21103016-21122286.NCBI.ref.revcompl                       tctattgcacagtatggtggctatagtaaataataatctatcatatatttcaaaattgct 12295
LOC102725072.has.POM121L1                                       ------------------------------------------------------------ 14779
LOC102725072.start-FAM230F.start.18846286-18865042.NEW.ref      ------------------------------------------------------------ 16904

BCRP2.21103016-21122286.NCBI.ref.revcompl                       aagagtaaatttcaaatgtcccaccacaaaggtgagctggatgatggatatgttaattag 12355
LOC102725072.has.POM121L1                                       ------------------------------------------------------------ 14779
LOC102725072.start-FAM230F.start.18846286-18865042.NEW.ref      ------------------------------------------------------------ 16904

BCRP2.21103016-21122286.NCBI.ref.revcompl                       ctttatttaatcattccacattgtgtacacatatcaaaacatcacattgtactcaagaca 12415
LOC102725072.has.POM121L1                                       ------------------------------------------------------------ 14779
LOC102725072.start-FAM230F.start.18846286-18865042.NEW.ref      ------------------------------------------------------------ 16904

BCRP2.21103016-21122286.NCBI.ref.revcompl                       tctatacaattgacttgtcaaccaaaataataaaaataaagtctataaaatgtaaaatag 12475
LOC102725072.has.POM121L1                                       ------------------------------------------------------------ 14779
LOC102725072.start-FAM230F.start.18846286-18865042.NEW.ref      ------------------------------------------------------------ 16904

BCRP2.21103016-21122286.NCBI.ref.revcompl                       ttttgttacacagtttagtgttctttaaaccatattggctgagcttcctctttgccttgc 12535
LOC102725072.has.POM121L1                                       ------------------------------------------------------------ 14779
LOC102725072.start-FAM230F.start.18846286-18865042.NEW.ref      ------------------------------------------------------------ 16904

BCRP2.21103016-21122286.NCBI.ref.revcompl                       ccctttctcaagtctgggtgctgaatggtaaatatagaatcagttaatgtttactgagga 12595
LOC102725072.has.POM121L1                                       ------------------------------------------------------------ 14779
LOC102725072.start-FAM230F.start.18846286-18865042.NEW.ref      ------------------------------------------------------------ 16904

BCRP2.21103016-21122286.NCBI.ref.revcompl                       tctagtatgtgcctcacactgacctaaacattttataactaatttttcaataccctggt 12655
LOC102725072.has.POM121L1                                       ------------------------------------------------------------ 14779
LOC102725072.start-FAM230F.start.18846286-18865042.NEW.ref      ------------------------------------------------------------ 16904

BCRP2.21103016-21122286.NCBI.ref.revcompl                       gaggtggtttttatgattatcccatttggcttgagctagatgatctcagagaggttaaga 12715
LOC102725072.has.POM121L1                                       ------------------------------------------------------------ 14779
LOC102725072.start-FAM230F.start.18846286-18865042.NEW.ref      ------------------------------------------------------------ 16904

BCRP2.21103016-21122286.NCBI.ref.revcompl                       aatcttcaaatcttcacaaggctgcagaactactaaaaggcaaggcctgtataattactt 12775
LOC102725072.has.POM121L1                                       ------------------------------------------------------------ 14779
LOC102725072.start-FAM230F.start.18846286-18865042.NEW.ref      ------------------------------------------------------------ 16904

BCRP2.21103016-21122286.NCBI.ref.revcompl                       gttgaaggaatgaatgatatgagactgtttattgttttaaattatggaacaatttatact 12835
LOC102725072.has.POM121L1                                       ------------------------------------------------------------ 14779
LOC102725072.start-FAM230F.start.18846286-18865042.NEW.ref      ------------------------------------------------------------ 16904
```

```
BCRP2.21103016-21122286.NCBI.ref.revcompl                        ttgtaaaatgcagaagttttaaatgttcacttctgtgagctttgacagttatagaccctc 12895
LOC102725072.has.POM121L1                                        ------------------------------------------------------------ 14779
LOC102725072.start-FAM230F.start.18846286-18865042.NEW.ref       ------------------------------------------------------------ 16904

BCRP2.21103016-21122286.NCBI.ref.revcompl                        atgttactatcccagtcaaaatatagaacatttccagaaaatcctctcacatctctttcc 12955
LOC102725072.has.POM121L1                                        ------------------------------------------------------------ 14779
LOC102725072.start-FAM230F.start.18846286-18865042.NEW.ref       ------------------------------------------------------------ 16904

BCRP2.21103016-21122286.NCBI.ref.revcompl                        agtcagtaccttcatgggtcaaaaacaactaccttctgagctctgtcaccaaagataaat 13015
LOC102725072.has.POM121L1                                        ------------------------------------------------------------ 14779
LOC102725072.start-FAM230F.start.18846286-18865042.NEW.ref       ------------------------------------------------------------ 16904

BCRP2.21103016-21122286.NCBI.ref.revcompl                        ttttcttatggctgcatttcaaataaatggaatcacacatccacacttttcaatgtctgg 13075
LOC102725072.has.POM121L1                                        ------------------------------------------------------------ 14779
LOC102725072.start-FAM230F.start.18846286-18865042.NEW.ref       ------------------------------------------------------------ 16904

BCRP2.21103016-21122286.NCBI.ref.revcompl                        cttccttctctaaatacattttctgttttgagattcacccatgctgttgtgcgattcta 13135
LOC102725072.has.POM121L1                                        ------------------------------------------------------------ 14779
LOC102725072.start-FAM230F.start.18846286-18865042.NEW.ref       ------------------------------------------------------------ 16904

BCRP2.21103016-21122286.NCBI.ref.revcompl                        tagttctcttttaatcactgaatatatactgttgtgtgatacaccatggtttatttattt 13195
LOC102725072.has.POM121L1                                        ------------------------------------------------------------ 14779
LOC102725072.start-FAM230F.start.18846286-18865042.NEW.ref       ------------------------------------------------------------ 16904

BCRP2.21103016-21122286.NCBI.ref.revcompl                        attcactgtcaaaggacattggctcatttccaagtttgggttaacgtaaataagactgct 13255
LOC102725072.has.POM121L1                                        ------------------------------------------------------------ 14779
LOC102725072.start-FAM230F.start.18846286-18865042.NEW.ref       ------------------------------------------------------------ 16904

BCRP2.21103016-21122286.NCBI.ref.revcompl                        atgttagattctagtacaagtctacgtaggtgtacgtgtgtatacatacacatacataca 13315
LOC102725072.has.POM121L1                                        ------------------------------------------------------------ 14779
LOC102725072.start-FAM230F.start.18846286-18865042.NEW.ref       ------------------------------------------------------------ 16904

BCRP2.21103016-21122286.NCBI.ref.revcompl                        catacatacacacatagacttgtactagaaaaaatgtactaaatgtactaaaaaatgtac 13375
LOC102725072.has.POM121L1                                        ------------------------------------------------------------ 14779
LOC102725072.start-FAM230F.start.18846286-18865042.NEW.ref       ------------------------------------------------------------ 16904

BCRP2.21103016-21122286.NCBI.ref.revcompl                        taaaatgtacattttatttttcttggatcaacagcttggagtaaaagtgctgagtcata 13435
LOC102725072.has.POM121L1                                        ------------------------------------------------------------ 14779
LOC102725072.start-FAM230F.start.18846286-18865042.NEW.ref       ------------------------------------------------------------ 16904

BCRP2.21103016-21122286.NCBI.ref.revcompl                        ggttaagtgtatatttaatattacaagaaattgctacaccatttccaaagtagttgtttt 13495
LOC102725072.has.POM121L1                                        ------------------------------------------------------------ 14779
LOC102725072.start-FAM230F.start.18846286-18865042.NEW.ref       ------------------------------------------------------------ 16904

BCRP2.21103016-21122286.NCBI.ref.revcompl                        acaattccaccagttttatgagagtgttggttaattcttattttcatcaacatttagtat 13555
LOC102725072.has.POM121L1                                        ------------------------------------------------------------ 14779
LOC102725072.start-FAM230F.start.18846286-18865042.NEW.ref       ------------------------------------------------------------ 16904

BCRP2.21103016-21122286.NCBI.ref.revcompl                        tatcagtcgttttgattttgccattacagtggctaaaatgtgaaatcaaatgaaattgta 13615
LOC102725072.has.POM121L1                                        ------------------------------------------------------------ 14779
LOC102725072.start-FAM230F.start.18846286-18865042.NEW.ref       ------------------------------------------------------------ 16904

BCRP2.21103016-21122286.NCBI.ref.revcompl                        tcttgagattttaacttgcatcttcctgactgttagtgatgttgaccattgtttaatata 13675
LOC102725072.has.POM121L1                                        ------------------------------------------------------------ 14779
LOC102725072.start-FAM230F.start.18846286-18865042.NEW.ref       ------------------------------------------------------------ 16904

BCRP2.21103016-21122286.NCBI.ref.revcompl                        cccactggctatttgtatgtcttgctttgcaaactgtctgttcaagttttctactttt 13735
LOC102725072.has.POM121L1                                        ------------------------------------------------------------ 14779
LOC102725072.start-FAM230F.start.18846286-18865042.NEW.ref       ------------------------------------------------------------ 16904

BCRP2.21103016-21122286.NCBI.ref.revcompl                        aattggcctgtttgcctctgtattgttaacttgtaaaagttctttatatgagatgaatta 13795
LOC102725072.has.POM121L1                                        ------------------------------------------------------------ 14779
LOC102725072.start-FAM230F.start.18846286-18865042.NEW.ref       ------------------------------------------------------------ 16904

BCRP2.21103016-21122286.NCBI.ref.revcompl                        tttgacaaatgcatgtattacatatatttccccaagtctgtggcttgcatattcatgttt 13855
LOC102725072.has.POM121L1                                        ------------------------------------------------------------ 14779
LOC102725072.start-FAM230F.start.18846286-18865042.NEW.ref       ------------------------------------------------------------ 16904

BCRP2.21103016-21122286.NCBI.ref.revcompl                        atagtgtcttttgataacctttaattttcactgaagtttaacctataaatgtctttatgt 13915
LOC102725072.has.POM121L1                                        ------------------------------------------------------------ 14779
LOC102725072.start-FAM230F.start.18846286-18865042.NEW.ref       ------------------------------------------------------------ 16904

BCRP2.21103016-21122286.NCBI.ref.revcompl                        agggttagagctttctgtgtccttacaagaaacttctacctgttccaaggttagaaagaa 13975
LOC102725072.has.POM121L1                                        ------------------------------------------------------------ 14779
LOC102725072.start-FAM230F.start.18846286-18865042.NEW.ref       ------------------------------------------------------------ 16904

BCRP2.21103016-21122286.NCBI.ref.revcompl                        gtacccatttgttttccgtatggagtgagaatgggatgcaaatatttatattttgttatt 14035
LOC102725072.has.POM121L1                                        ------------------------------------------------------------ 14779
LOC102725072.start-FAM230F.start.18846286-18865042.NEW.ref       ------------------------------------------------------------ 16904

BCRP2.21103016-21122286.NCBI.ref.revcompl                        caagcagcatttggtacaaaaattcttcttccctctttgaattcccttgtcaacttctga 14095
LOC102725072.has.POM121L1                                        ------------------------------------------------------------ 14779
LOC102725072.start-FAM230F.start.18846286-18865042.NEW.ref       ------------------------------------------------------------ 16904

BCRP2.21103016-21122286.NCBI.ref.revcompl                        gttcacttttcctcatgcacattattggtaagcattttgtgtaaaataaactgccttta 14155
LOC102725072.has.POM121L1                                        ------------------------------------------------------------ 14779
LOC102725072.start-FAM230F.start.18846286-18865042.NEW.ref       ------------------------------------------------------------ 16904

BCRP2.21103016-21122286.NCBI.ref.revcompl                        tcagaagtgtacaatttttcagatatctttccataatcctctagatttgaaaggtgatt 14215
LOC102725072.has.POM121L1                                        ------------------------------------------------------------ 14779
LOC102725072.start-FAM230F.start.18846286-18865042.NEW.ref       ------------------------------------------------------------ 16904

BCRP2.21103016-21122286.NCBI.ref.revcompl                        tttggttctcttgctgacatgttataattatagctcccttctgctgccatgtgatttgca 14275
LOC102725072.has.POM121L1                                        ------------------------------------------------------------ 14779
LOC102725072.start-FAM230F.start.18846286-18865042.NEW.ref       ------------------------------------------------------------ 16904

BCRP2.21103016-21122286.NCBI.ref.revcompl                        atccttttaatctttccatttaagacctttcttactcctatataaaaactataattgtg 14335
LOC102725072.has.POM121L1                                        ------------------------------------------------------------ 14779
LOC102725072.start-FAM230F.start.18846286-18865042.NEW.ref       ------------------------------------------------------------ 16904

BCRP2.21103016-21122286.NCBI.ref.revcompl                        gccaggcatggtggctcacacctgtaatcccagcactttgggaggccgaggtgggtggat 14395
LOC102725072.has.POM121L1                                        ------------------------------------------------------------ 14779
LOC102725072.start-FAM230F.start.18846286-18865042.NEW.ref       ------------------------------------------------------------ 16904

BCRP2.21103016-21122286.NCBI.ref.revcompl                        catctgaggtcaggagttcgagaccagcctgggcaacatggtgaaaccctgtctctacta 14455
LOC102725072.has.POM121L1                                        ------------------------------------------------------------ 14779
LOC102725072.start-FAM230F.start.18846286-18865042.NEW.ref       ------------------------------------------------------------ 16904

BCRP2.21103016-21122286.NCBI.ref.revcompl                        aaaataccaaaataagctgggtgtggtgtgtgcctgtagtcccagctactcgggaggc 14515
LOC102725072.has.POM121L1                                        ------------------------------------------------------------ 14779
LOC102725072.start-FAM230F.start.18846286-18865042.NEW.ref       --------------------------------------------------actcgggaggc 16915

BCRP2.21103016-21122286.NCBI.ref.revcompl                        tgaggcaggagaatcgcttaaagccaggaggtggaggttgcagtgagtcaagatcgcgcc 14575
LOC102725072.has.POM121L1                                        ------------------------------------------------------------ 14779
LOC102725072.start-FAM230F.start.18846286-18865042.NEW.ref       tgaggagggagaatcgtttgaacctgggaggcggaggttgcagtgagccaagattgtgcc 16975
```

```
BCRP2.21103016-21122286.NCBI.ref.revcompl                   cctgcactcaggcctgggtgacagagcaagattctgtctcaaaaaaaaaaaaaaaaaaaa 14635
LOC102725072.has.POM121L1                                   ------------------------------------------------------------ 14779
LOC102725072.start-FAM230F.start.18846286-18865042.NEW.ref  actgcactctagcctgggctacagggcaagactccattaaaaaaaaaaaaaaaaaccagca 17035

BCRP2.21103016-21122286.NCBI.ref.revcompl                   atcctataattgctttagtcatgcatatattgaaacactgtgtacaataattaggcaatt 14695
LOC102725072.has.POM121L1                                   ------------------------------------------------------------ 14779
LOC102725072.start-FAM230F.start.18846286-18865042.NEW.ref  aaaaacaa---------------------------------------------------- 17043

BCRP2.21103016-21122286.NCBI.ref.revcompl                   gaatttttgtatttttctaaaattcaaaaggagaaaagctaatctagtttgtaatgatat 14755
LOC102725072.has.POM121L1                                   ------------------------------------------------------------ 14779
LOC102725072.start-FAM230F.start.18846286-18865042.NEW.ref  ------------------------------------------------------------ 17043

BCRP2.21103016-21122286.NCBI.ref.revcompl                   agattcactaagacacttgtttctgtctttaggatagagcattcatactaacagtgtgag 14815
LOC102725072.has.POM121L1                                   ------------------------------------------------------------ 14779
LOC102725072.start-FAM230F.start.18846286-18865042.NEW.ref  ------------------------------------------------------------ 17043

BCRP2.21103016-21122286.NCBI.ref.revcompl                   tagtatggataaaccagcatatatatttttttattatgttgaatttcaatatccagccgaa 14875
LOC102725072.has.POM121L1                                   ------------------------------------------------------------ 14779
LOC102725072.start-FAM230F.start.18846286-18865042.NEW.ref  ------------------------------------------------------------ 17043

BCRP2.21103016-21122286.NCBI.ref.revcompl                   ttctgtctagttgcattacttacaacattgtgttacagtttgaaatcaagaagaaacttg 14935
LOC102725072.has.POM121L1                                   ------------------------------------------------------------ 14779
LOC102725072.start-FAM230F.start.18846286-18865042.NEW.ref  ------------------------------------------------------------ 17043

BCRP2.21103016-21122286.NCBI.ref.revcompl                   tgaatgacttttctacaatttctctgtaaaacatagatttgtcagataacatagtacctt 14995
LOC102725072.has.POM121L1                                   ------------------------------------------------------------ 14779
LOC102725072.start-FAM230F.start.18846286-18865042.NEW.ref  -------------------------------------------acaaaacataatgcatg 17060

BCRP2.21103016-21122286.NCBI.ref.revcompl                   atgtggtaattaaagaaattgttacaatgagaacacatggacacagagaggggaacatca 15055
LOC102725072.has.POM121L1                                   ------------------------------------------------------------ 14779
LOC102725072.start-FAM230F.start.18846286-18865042.NEW.ref  ttctctcttataaatgggagctaaacatggggactcattgacttaaagatggcaacaact 17120

BCRP2.21103016-21122286.NCBI.ref.revcompl                   cacaccatggcatgtcaggggggttgggggcaaggggagggagagcattagggcacatacc 15115
LOC102725072.has.POM121L1                                   ------------------------------------------------------------ 14779
LOC102725072.start-FAM230F.start.18846286-18865042.NEW.ref  gggaactgctggatggggagggaggggaggggtgaaaggccaactgttggggagtatgct 17180

BCRP2.21103016-21122286.NCBI.ref.revcompl                   taatgcataccaggcttgaaacctagatgacgggttgatagatgcagcaaaccaccatgg 15175
LOC102725072.has.POM121L1                                   ------------------------------------------------------------ 14779
LOC102725072.start-FAM230F.start.18846286-18865042.NEW.ref  ------------------------------------------------------------ 17180

BCRP2.21103016-21122286.NCBI.ref.revcompl                   cacatgtatacctatgtaacaaacctgcacattctgcacatgtatcccaggacttaaagt 15235
LOC102725072.has.POM121L1                                   ------------------------------------------------------------ 14779
LOC102725072.start-FAM230F.start.18846286-18865042.NEW.ref  ------catatccacgtgacaaacctgcacatgtgcccgctgaatc--------taaaat 17226

BCRP2.21103016-21122286.NCBI.ref.revcompl                   aaaatttaaaaagaaattgttacataaatgttttagtggctttaatttttaattaaattg 15295
LOC102725072.has.POM121L1                                   ------------------------------------------------------------ 14779
LOC102725072.start-FAM230F.start.18846286-18865042.NEW.ref  aaaagttgaaagtagatt------------------------------------------ 17244

BCRP2.21103016-21122286.NCBI.ref.revcompl                   tgttaacttttttaggacacgaaaaattaaaatatgaaaagtaaaattttactatatgcac 15355
LOC102725072.has.POM121L1                                   ------------------------------------------------------------ 14779
LOC102725072.start-FAM230F.start.18846286-18865042.NEW.ref  ------------------------------------------------------------ 17244

BCRP2.21103016-21122286.NCBI.ref.revcompl                   agaaaaatctcatttacttctgtgaggcaggaagtcttatactctgagtggggaaatata 15415
LOC102725072.has.POM121L1                                   ------------------------------------------------------------ 14779
LOC102725072.start-FAM230F.start.18846286-18865042.NEW.ref  ------------------------------------------------------------ 17244

BCRP2.21103016-21122286.NCBI.ref.revcompl                   aatggcacctaattcctgcatttgattattaccgttcttggctgttgccacaataggttt 15475
LOC102725072.has.POM121L1                                   ------------------------------------------------------------ 14779
LOC102725072.start-FAM230F.start.18846286-18865042.NEW.ref  ------------------------------------------------------------ 17244

BCRP2.21103016-21122286.NCBI.ref.revcompl                   atctttttacacatcaaaaaaaggaaattatgaggaatcatatatctgaaagggagtgtt 15535
LOC102725072.has.POM121L1                                   ------------------------------------------------------------ 14779
LOC102725072.start-FAM230F.start.18846286-18865042.NEW.ref  ------------------------------------------------------------ 17244

BCRP2.21103016-21122286.NCBI.ref.revcompl                   atctgacaacagcagaaactaacactacagagggacaaaacatacaagacccaatacccc 15595
LOC102725072.has.POM121L1                                   ------------------------------------------------------------ 14779
LOC102725072.start-FAM230F.start.18846286-18865042.NEW.ref  ------------------------------------------------------------ 17244

BCRP2.21103016-21122286.NCBI.ref.revcompl                   caaaaaaactgggcaaaaaaacctgaataacagacatttctgaaaagaaggtatgatatt 15655
LOC102725072.has.POM121L1                                   ------------------------------------------------------------ 14779
LOC102725072.start-FAM230F.start.18846286-18865042.NEW.ref  ------------------------------------------------------------ 17244

BCRP2.21103016-21122286.NCBI.ref.revcompl                   gattaaaaggtaaaaaaggtgtttctcaacatcactggtcatcacaaaactgtaaataaa 15715
LOC102725072.has.POM121L1                                   ------------------------------------------------------------ 14779
LOC102725072.start-FAM230F.start.18846286-18865042.NEW.ref  ------------------------------------------------------------ 17244

BCRP2.21103016-21122286.NCBI.ref.revcompl                   gaccacactcagatactatctaacgctaagtaaaatgaataataccaaaacagaaataat 15775
LOC102725072.has.POM121L1                                   ------------------------------------------------------------ 14779
LOC102725072.start-FAM230F.start.18846286-18865042.NEW.ref  ------------------------------------------------------------ 17244

BCRP2.21103016-21122286.NCBI.ref.revcompl                   ttaaaaataaaagttcagggtgctgttagatatagagatcctcttatacactattgggag 15835
LOC102725072.has.POM121L1                                   ------------------------------------------------------------ 14779
LOC102725072.start-FAM230F.start.18846286-18865042.NEW.ref  ------------------------------------------------------------ 17244

BCRP2.21103016-21122286.NCBI.ref.revcompl                   aaatttcaatgatgaaacgcactgagaaaaccacttggagcaccctcagaaattaaaaat 15895
LOC102725072.has.POM121L1                                   ------------------------------------------------------------ 14779
LOC102725072.start-FAM230F.start.18846286-18865042.NEW.ref  ------------------------------------------------------------ 17244

BCRP2.21103016-21122286.NCBI.ref.revcompl                   gcaagaactgtttcatctagtaattctaaccgtggttgtgagcttaaagaaaatgagatc 15955
LOC102725072.has.POM121L1                                   ------------------------------------------------------------ 14779
LOC102725072.start-FAM230F.start.18846286-18865042.NEW.ref  ------------------------------------------------------------ 17244

BCRP2.21103016-21122286.NCBI.ref.revcompl                   ggtctgttgaagcaacagctgccttctgaggtttagtgacctactactcactaacaatga 16015
LOC102725072.has.POM121L1                                   ------------------------------------------------------------ 14779
LOC102725072.start-FAM230F.start.18846286-18865042.NEW.ref  ------------------------------------------------------------ 17244

BCRP2.21103016-21122286.NCBI.ref.revcompl                   gtaaccaaggtatgaaatcaacctacctgtccaccaaccaatgaaaggatgaagggactt 16075
LOC102725072.has.POM121L1                                   ------------------------------------------------------------ 14779
LOC102725072.start-FAM230F.start.18846286-18865042.NEW.ref  ------------------------------------------------------------ 17244

BCRP2.21103016-21122286.NCBI.ref.revcompl                   cagcatacagacatgatgaaatatgcctcatgcataaaaattcatgacaacatgtcattt 16135
LOC102725072.has.POM121L1                                   ------------------------------------------------------------ 14779
LOC102725072.start-FAM230F.start.18846286-18865042.NEW.ref  ------------------------------------------------------------ 17244

BCRP2.21103016-21122286.NCBI.ref.revcompl                   gcaaaaacacagacgaacctggaagacattacattaaatgaaatcgactaggtagagaaa 16195
LOC102725072.has.POM121L1                                   ------------------------------------------------------------ 14779
LOC102725072.start-FAM230F.start.18846286-18865042.NEW.ref  ------------------------------------------------------------ 17244

BCRP2.21103016-21122286.NCBI.ref.revcompl                   ggcaaacactatgtgatctcacttatatggaatccaaaaaactttaccttcaagcagaag 16255
LOC102725072.has.POM121L1                                   ------------------------------------------------------------ 14779
LOC102725072.start-FAM230F.start.18846286-18865042.NEW.ref  ------------------------------------------------------------ 17244

BCRP2.21103016-21122286.NCBI.ref.revcompl                   gtacagtagcggtttcctgaggagaggggaaggaggggatagagggggatggaggga 16315
LOC102725072.has.POM121L1                                   ------------------------------------------------------------ 14779
LOC102725072.start-FAM230F.start.18846286-18865042.NEW.ref  ------------------------------------------------------------ 17244
```

```
BCRP2.21103016-21122286.NCBI.ref.revcompl                        tcggttatggaaacaaagttacagtgagtttggcgaaacacattttggttttctatgcca 16375
LOC102725072.has.POM121L1                                        ------------------------------------------------------------ 14779
LOC102725072.start-FAM230F.start.18846286-18865042.NEW.ref       ------------------------------------------------------------ 17244

BCRP2.21103016-21122286.NCBI.ref.revcompl                        cagctgggtaactctggttaacaaaatattgtatttttcaaaaactagaagggagcaat 16435
LOC102725072.has.POM121L1                                        ------------------------------------------------------------ 14779
LOC102725072.start-FAM230F.start.18846286-18865042.NEW.ref       ------------------------------------------------------------ 17244

BCRP2.21103016-21122286.NCBI.ref.revcompl                        ttgaatgtactcacaacaaagaaataatacctcaatgagggaatagttatgataagtacc 16495
LOC102725072.has.POM121L1                                        ------------------------------------------------------------ 14779
LOC102725072.start-FAM230F.start.18846286-18865042.NEW.ref       ------------------------------------------------------------ 17244

BCRP2.21103016-21122286.NCBI.ref.revcompl                        ctgatttgatcattactcaaagtatacatgtatcaaaatgtccccaaagaaaaaaacatg 16555
LOC102725072.has.POM121L1                                        ------------------------------------------------------------ 14779
LOC102725072.start-FAM230F.start.18846286-18865042.NEW.ref       -------------------------------------------taaaaaaccccaagagg 17261

BCRP2.21103016-21122286.NCBI.ref.revcompl                        tcccagcataacttctaattgtgtatgttattatacatggcaaaaatttaagaaatatga 16615
LOC102725072.has.POM121L1                                        ------------------------------------------------------------ 14779
LOC102725072.start-FAM230F.start.18846286-18865042.NEW.ref       gctgggtttggcttgtgtgtccatagcttgttaacctccgctttagatattaactaatag 17321

BCRP2.21103016-21122286.NCBI.ref.revcompl                        atataaaatgctaatattcacatagaaccactaatggccctgaacctctaaggaatcctg 16675
LOC102725072.has.POM121L1                                        ------------------------------------------------------------ 14779
LOC102725072.start-FAM230F.start.18846286-18865042.NEW.ref       aaacctagtgcttatcttcccaggccacctattttgttcct----ctccaaggtgatgga 17377

BCRP2.21103016-21122286.NCBI.ref.revcompl                        agaaatacaaactaagttgggagactcacaattcctgatagcaaattacattgcaaagct 16735
LOC102725072.has.POM121L1                                        ------------------------------------------------------------ 14779
LOC102725072.start-FAM230F.start.18846286-18865042.NEW.ref       tagatgaaggcctaatccagccgcctggaagtt--tgctgacgcttgtcctgtcacagat 17435

BCRP2.21103016-21122286.NCBI.ref.revcompl                        gtaggtagccaatatatatggtcatg---------tgatgtgctactgtg----ctctcc 16782
LOC102725072.has.POM121L1                                        ------------------------------------------------------------ 14779
LOC102725072.start-FAM230F.start.18846286-18865042.NEW.ref       taatgaagcattgttttctgatgaagctttcatgccgctgtgctgatgtgtcttctcttc 17495

BCRP2.21103016-21122286.NCBI.ref.revcompl                        agcctgggtgagagaatgagactctgtctcaaaaaaaaaaaaag-------aaagggaat 16835
LOC102725072.has.POM121L1                                        ------------------------------------------------------------ 14779
LOC102725072.start-FAM230F.start.18846286-18865042.NEW.ref       tctctaggcaggaaactgcatatcttctggtttacatgaagatggagtgctaatggaaat 17555

BCRP2.21103016-21122286.NCBI.ref.revcompl                        catgtaagcgataactcaatcactggttgttatcaaacatatacacagaaaaaaaataca 16895
LOC102725072.has.POM121L1                                        ------------------------------------------------------------ 14779
LOC102725072.start-FAM230F.start.18846286-18865042.NEW.ref       gcccaaaaccttcagagattgacacgctgtcattttccatttcc---------------- 17599

BCRP2.21103016-21122286.NCBI.ref.revcompl                        atgtagcaaaatacatatccaaaccaatacaaagaacttgagtagacatctctgcaaaga 16955
LOC102725072.has.POM121L1                                        ------------------------------------------------------------ 14779
LOC102725072.start-FAM230F.start.18846286-18865042.NEW.ref       ------------------------------------------------------------ 17599

BCRP2.21103016-21122286.NCBI.ref.revcompl                        tgacacaaaattgaccaacatgtaagagaaacggtcctcaacgtctctcatcagaaaaaa 17015
LOC102725072.has.POM121L1                                        ------------------------------------------------------------ 14779
LOC102725072.start-FAM230F.start.18846286-18865042.NEW.ref       ------------------------------------------------------------ 17599

BCRP2.21103016-21122286.NCBI.ref.revcompl                        aatgtacctcaaaaccacattcagatgccgtttcacttttattgaaataaatgttactaa 17075
LOC102725072.has.POM121L1                                        ------------------------------------------------------------ 14779
LOC102725072.start-FAM230F.start.18846286-18865042.NEW.ref       ------------------------------------------------------------ 17599

BCRP2.21103016-21122286.NCBI.ref.revcompl                        aaagtttttttttaatttttttgagacagggactcagactttgtcacccaggctgaagtgc 17135
LOC102725072.has.POM121L1                                        ------------------------------------------------------------ 14779
LOC102725072.start-FAM230F.start.18846286-18865042.NEW.ref       ------------------------------------------------------------ 17599

BCRP2.21103016-21122286.NCBI.ref.revcompl                        agtgtcgtgatcccagctcactgcagctttgacctccctagctcaggtgatcatcccact 17195
LOC102725072.has.POM121L1                                        ------------------------------------------------------------ 14779
LOC102725072.start-FAM230F.start.18846286-18865042.NEW.ref       ------------------------------------------------------------ 17599

BCRP2.21103016-21122286.NCBI.ref.revcompl                        tcagcctctcaagtatctgggactacaggtgcacaccaccatgcctcatcctcccaaagt 17255
LOC102725072.has.POM121L1                                        ------------------------------------------------------------ 14779
LOC102725072.start-FAM230F.start.18846286-18865042.NEW.ref       ------------------------------------------------------------ 17599

BCRP2.21103016-21122286.NCBI.ref.revcompl                        gctgggattacaggcatgagccactgttcctggccacaaaattttaaaaagaatgctggt 17315
LOC102725072.has.POM121L1                                        ------------------------------------------------------------ 14779
LOC102725072.start-FAM230F.start.18846286-18865042.NEW.ref       ------------------------------------------------------------ 17599

BCRP2.21103016-21122286.NCBI.ref.revcompl                        atggatttggagaaaagggaactcttataccctgttgatgggaagataaattagtacata 17375
LOC102725072.has.POM121L1                                        ------------------------------------------------------------ 14779
LOC102725072.start-FAM230F.start.18846286-18865042.NEW.ref       ------------------------------------------------------------ 17599

BCRP2.21103016-21122286.NCBI.ref.revcompl                        ttctatggtaaaaagttggaagttgccattttcaaaaaactgaaaatacaattaccagaa 17435
LOC102725072.has.POM121L1                                        ------------------------------------------------------------ 14779
LOC102725072.start-FAM230F.start.18846286-18865042.NEW.ref       ------------------------------------------------------------ 17599

BCRP2.21103016-21122286.NCBI.ref.revcompl                        ggtcaatgtgggcagattacttgagctcaggagttcgagaccagcctgggcaacatggca 17495
LOC102725072.has.POM121L1                                        ------------------------------------------------------------ 14779
LOC102725072.start-FAM230F.start.18846286-18865042.NEW.ref       ---------------attcctggatctacggagtcttctaagagattttgcaatgaggag 17644

BCRP2.21103016-21122286.NCBI.ref.revcompl                        aaaccctgtctctacaaaaaatttaaaaacttagcc------------------------ 17531
LOC102725072.has.POM121L1                                        ------------------------------------------------------------ 14779
LOC102725072.start-FAM230F.start.18846286-18865042.NEW.ref       aagcattgttttcaaactatat-----aactgagccttatttataattaggatattatc 17699

BCRP2.21103016-21122286.NCBI.ref.revcompl                        ------------------------------------------------------------ 17531
LOC102725072.has.POM121L1                                        ------------------------------------------------------------ 14779
LOC102725072.start-FAM230F.start.18846286-18865042.NEW.ref       aaaatatgtaaccatgaggcccctcaggtcctgatcagtcagaatggatgctttcaccag 17759

BCRP2.21103016-21122286.NCBI.ref.revcompl                        -------------------------------------------------------aggca 17536
LOC102725072.has.POM121L1                                        ------------------------------------------------------------ 14779
LOC102725072.start-FAM230F.start.18846286-18865042.NEW.ref       cagacccggccatgtggctgctcggtcctgggtgctcgctgctgtgcgagacattagccc 17819

BCRP2.21103016-21122286.NCBI.ref.revcompl                        tggtggtgtgtgcctgtggtctcagctaccatagactgaggtgggagggctgcttgggct 17596
LOC102725072.has.POM121L1                                        ------------------------------------------------------------ 14779
LOC102725072.start-FAM230F.start.18846286-18865042.NEW.ref       tttagttatgagcctgtgggaacttcaggggttcccagtggggagagcagtggcagtggg 17879

BCRP2.21103016-21122286.NCBI.ref.revcompl                        gtggaggtagaggccacagtgagctgtgattgc--accactgcactccagcctgggtgac 17654
LOC102725072.has.POM121L1                                        ------------------------------------------------------------ 14779
LOC102725072.start-FAM230F.start.18846286-18865042.NEW.ref       aggcatctgggggccaaaggtcagtggcagggggtacttcagtattatacaactgctgtg 17939

BCRP2.21103016-21122286.NCBI.ref.revcompl                        agaagaaaccccgtctcaaagaaaatgacagaaaatgcaaat-----actgtaagatcta 17709
LOC102725072.has.POM121L1                                        ------------------------------------------------------------ 14779
LOC102725072.start-FAM230F.start.18846286-18865042.NEW.ref       accagacttgtatactggccgaatatcagtgctgtttgtaatttttcactttgagaacca 17999

BCRP2.21103016-21122286.NCBI.ref.revcompl                        gcaatcccactactgggtataactccagagcaaatgaaatcggcaccagagatcagaaag 17769
LOC102725072.has.POM121L1                                        ------------------------------------------------------------ 14779
LOC102725072.start-FAM230F.start.18846286-18865042.NEW.ref       acattaattccatatgaatcaagtgttttgtaactgctattc------------------ 18041

BCRP2.21103016-21122286.NCBI.ref.revcompl                        acacaatggggacaggagagtttcttcatgaaatcattttgaggaaactgatatccatat 17829
LOC102725072.has.POM121L1                                        ------------------------------------------------------------ 14779
LOC102725072.start-FAM230F.start.18846286-18865042.NEW.ref       -------------------atttattcagcaaatatttattgatcatctcttctccataa 18082

BCRP2.21103016-21122286.NCBI.ref.revcompl                        gcaaaaaagaaaagaaaagaaataggacccttctgttacacgatacacaaaaatcaactc 17889
LOC102725072.has.POM121L1                                        ------------------------------------------------------------ 14779
LOC102725072.start-FAM230F.start.18846286-18865042.NEW.ref       gatagtgtgataaacacagtcatgaataaagttattttccacaaaaggactttgcagttt 18142
```

```
BCRP2.21103016-21122286.NCBI.ref.revcompl                    aaaatgg----attaaagatttcaacacaaaacctgaaaccacaaaacattattaaattc 17945
LOC102725072.has.POM121L1                                    ------------------------------------------------------------ 14779
LOC102725072.start-FAM230F.start.18846286-18865042.NEW.ref   taacgggggcagtagggattgtgctatagaaattcaaagg---caagggaagtcacttc 18199

BCRP2.21103016-21122286.NCBI.ref.revcompl                    ctgtaagaaaagatagggggagcttactttcaggacaacttgaa---------------- 17989
LOC102725072.has.POM121L1                                    ------------------------------------------------------------ 14779
LOC102725072.start-FAM230F.start.18846286-18865042.NEW.ref   tgttgtggggccctgggaggagcctacaggctggaaagggttaaggtggaggtctccgat 18259

BCRP2.21103016-21122286.NCBI.ref.revcompl                    ------------------------------------------------------------ 17989
LOC102725072.has.POM121L1                                    ------------------------------------------------------------ 14779
LOC102725072.start-FAM230F.start.18846286-18865042.NEW.ref   aggggcagcgtacacagtggactggctgcaaaaggccgtgctcagcattcagacagcatc 18319

BCRP2.21103016-21122286.NCBI.ref.revcompl                    ------------------------------------------------------------ 17989
LOC102725072.has.POM121L1                                    ------------------------------------------------------------ 14779
LOC102725072.start-FAM230F.start.18846286-18865042.NEW.ref   acacactccgcttttctctaccagggaggcaggtggggaaggatagcgatgggaaggcag 18379

BCRP2.21103016-21122286.NCBI.ref.revcompl                    tccatgctcatcagttggaaagagtttcaaaaagaaaaagaggaatacat---------- 18039
LOC102725072.has.POM121L1                                    ------------------------------------------------------------ 14779
LOC102725072.start-FAM230F.start.18846286-18865042.NEW.ref   gcggagctcagaatgtggaaggggatccagtaaggcttggaagtttgcacctgatctggt 18439

BCRP2.21103016-21122286.NCBI.ref.revcompl                    -------------------aaggggtaaaaacctcatggagaatggaatggtttgtgaat 18080
LOC102725072.has.POM121L1                                    ------------------------------------------------------------ 14779
LOC102725072.start-FAM230F.start.18846286-18865042.NEW.ref   gggtggtgaggagcccttgaaggggcaaggaggtgagaagcactcagctgtgttcttaca 18499

BCRP2.21103016-21122286.NCBI.ref.revcompl                    gatttttttaaattggggaccaaaatcacaagcaaggaaagcaagaacaaccatgtgagg 18140
LOC102725072.has.POM121L1                                    ------------------------------------------------------------ 14779
LOC102725072.start-FAM230F.start.18846286-18865042.NEW.ref   ctgatctgccactggggttagagacaaacgtggtgggaatggaaagccacgca------- 18552

BCRP2.21103016-21122286.NCBI.ref.revcompl                    ctacaagaaactgaaaagcttctgtgccacaacggaaaaaatgagcccgaagtttggacg 18200
LOC102725072.has.POM121L1                                    ------------------------------------------------------------ 14779
LOC102725072.start-FAM230F.start.18846286-18865042.NEW.ref   ------------------------------------------------------------ 18552

BCRP2.21103016-21122286.NCBI.ref.revcompl                    aaagttttgaggaatcatataactgctaaggggttcttaccgaaaatatacaacacatta 18260
LOC102725072.has.POM121L1                                    ------------------------------------------------------------ 14779
LOC102725072.start-FAM230F.start.18846286-18865042.NEW.ref   ------------------------------------------------------------ 18552

BCRP2.21103016-21122286.NCBI.ref.revcompl                    acagtactaaatagcaatttaaaacaacaacaaaaaataatccaccccaaactgggcaga 18320
LOC102725072.has.POM121L1                                    ------------------------------------------------------------ 14779
LOC102725072.start-FAM230F.start.18846286-18865042.NEW.ref   ------------------------------------------cagtcactagcgcctctg 18570

BCRP2.21103016-21122286.NCBI.ref.revcompl                    ggcccagaacagacatttctgcaaagaggacaaaaaacccacaccgaggagtccctcgcc 18380
LOC102725072.has.POM121L1                                    ------------------------------------------------------------ 14779
LOC102725072.start-FAM230F.start.18846286-18865042.NEW.ref   gggggagaatggatgtggctggtgagagaacagggggggcccagggagagtccggcaccaa 18630

BCRP2.21103016-21122286.NCBI.ref.revcompl                    cctggtcaggcggccacggtgggaggga-------cacgagaggacacgcgctgcccaaa 18433
LOC102725072.has.POM121L1                                    ------------------------------------------------------------ 14779
LOC102725072.start-FAM230F.start.18846286-18865042.NEW.ref   cctggcgggggggagcccagtgggtgtgagcacccccactttagagatgaagtgatggaga 18690

BCRP2.21103016-21122286.NCBI.ref.revcompl                    agtgaagaagagtgacccctgggtcctgatggcaggaacgaagatttaaaaatgcagcaa 18493
LOC102725072.has.POM121L1                                    ------------------------------------------------------------ 14779
LOC102725072.start-FAM230F.start.18846286-18865042.NEW.ref   cattcagatgtttaaccccttgttcaagattccatacttgataaatggcagatcaaactc 18750

BCRP2.21103016-21122286.NCBI.ref.revcompl                    ggcccaatgcaaatcctccctctgaggcttggacacgaggcagccagccagaactgggcc 18553
LOC102725072.has.POM121L1                                    ------------------------------------------------------------ 14779
LOC102725072.start-FAM230F.start.18846286-18865042.NEW.ref   ccaacat----------------------------------------------------- 18757

BCRP2.21103016-21122286.NCBI.ref.revcompl                    tggccctgactccaacctgagcggggcttccctctctctccctctacacaggggaggttc 18613
LOC102725072.has.POM121L1                                    ------------------------------------------------------------ 14779
LOC102725072.start-FAM230F.start.18846286-18865042.NEW.ref   ------------------------------------------------------------ 18757

BCRP2.21103016-21122286.NCBI.ref.revcompl                    cctcagaaaaagtcttttctgttgttggtttggtttttttttttttttcagaactaga 18673
LOC102725072.has.POM121L1                                    ------------------------------------------------------------ 14779
LOC102725072.start-FAM230F.start.18846286-18865042.NEW.ref   ------------------------------------------------------------ 18757

BCRP2.21103016-21122286.NCBI.ref.revcompl                    aatgtatttatttatttatttattttaataaaaaccaagatttttttattattattata 18733
LOC102725072.has.POM121L1                                    ------------------------------------------------------------ 14779
LOC102725072.start-FAM230F.start.18846286-18865042.NEW.ref   ------------------------------------------------------------ 18757

BCRP2.21103016-21122286.NCBI.ref.revcompl                    ctttaaagttttagggtacatgtgcacaacatgcaggtttgttacatatgtatacatgtg 18793
LOC102725072.has.POM121L1                                    ------------------------------------------------------------ 14779
LOC102725072.start-FAM230F.start.18846286-18865042.NEW.ref   ------------------------------------------------------------ 18757

BCRP2.21103016-21122286.NCBI.ref.revcompl                    ccatgttggtgtgctgcacccatcaactcgtcatttagcatcaggtatatctcctaatgc 18853
LOC102725072.has.POM121L1                                    ------------------------------------------------------------ 14779
LOC102725072.start-FAM230F.start.18846286-18865042.NEW.ref   ------------------------------------------------------------ 18757

BCRP2.21103016-21122286.NCBI.ref.revcompl                    tatccctccccccctccccccccacccagaaaaagtcttaaacccctaaaaataccacagtc 18913
LOC102725072.has.POM121L1                                    ------------------------------------------------------------ 14779
LOC102725072.start-FAM230F.start.18846286-18865042.NEW.ref   ------------------------------------------------------------ 18757

BCRP2.21103016-21122286.NCBI.ref.revcompl                    tgctacacttgacaccctcaaaagccttcagagaatgaataccactgtaaaataaatact 18973
LOC102725072.has.POM121L1                                    ------------------------------------------------------------ 14779
LOC102725072.start-FAM230F.start.18846286-18865042.NEW.ref   ------------------------------------------------------------ 18757

BCRP2.21103016-21122286.NCBI.ref.revcompl                    aaaacactacaagagaaagaggaaaaccagggagaacaggagaggaatgaacagaacata 19033
LOC102725072.has.POM121L1                                    ------------------------------------------------------------ 14779
LOC102725072.start-FAM230F.start.18846286-18865042.NEW.ref   ------------------------------------------------------------ 18757

BCRP2.21103016-21122286.NCBI.ref.revcompl                    ccagaaaactaaagcttctgtactcaccatgcaggaaaaatgccagggaaaagtgcatgg 19093
LOC102725072.has.POM121L1                                    ------------------------------------------------------------ 14779
LOC102725072.start-FAM230F.start.18846286-18865042.NEW.ref   ------------------------------------------------------------ 18757

BCRP2.21103016-21122286.NCBI.ref.revcompl                    cagcttcctcctcagcactcagggcccagacaggagcggttttctccccaggacacagct 19153
LOC102725072.has.POM121L1                                    ------------------------------------------------------------ 14779
LOC102725072.start-FAM230F.start.18846286-18865042.NEW.ref   ------------------------------------------------------------ 18757

BCRP2.21103016-21122286.NCBI.ref.revcompl                    ccaagtcgtccagaagcctccgtgatgcccctcctggcgggtcctgagggtatcagagct 19213
LOC102725072.has.POM121L1                                    ------------------------------------------------------------ 14779
LOC102725072.start-FAM230F.start.18846286-18865042.NEW.ref   ------------------------------------------------------------ 18757

BCRP2.21103016-21122286.NCBI.ref.revcompl                    cctctcctcctcacggtggatgccgccactcactccatgcagcacctgcggggagaac   19271
LOC102725072.has.POM121L1                                    ----------------------------------------------------------   14779
LOC102725072.start-FAM230F.start.18846286-18865042.NEW.ref   ----------------------------------------------------------   18757
```
