# Supplementary material for: Birth of a Regulatory Long Non-coding RNA/Gene, linc-UR-UB
Source: Front Genet. 2021 Apr 30;12:661425. doi: 10.3389/fgene.2021.661425 (PMC8120154; doi:10.3389/fgene.2021.661425)
Supplement: Supplementary file 2 [file Data_Sheet_2.PDF]

Birth of a regulatory long non-coding RNA gene, *linc-UR-UB*

Nicholas Delihas

Department of Microbiology and Immunology
Renaissance School of Medicine
Stony Brook University
Stony Brook, New York, 11794-5222

Email: Nicholas.delihas@stonybook.edu

Supplementary Figure S2. Alignment of nucleotide sequences from the chimpanzee genome chr 22, with human chr 22 genomic sequences that contain the *USP18* exon11 3' UTR and BCRP2. The green highlighted region shows the exon11.USP18 3'UTR sequence in *linc-UR-B1* (which is within the *LOC102725072-FAM230F* sequence), and it is the absence in chimp sequence. The section representing the identity of genomic sequences with the *BCRP2* gene is in purple.The chimpanzee sequence is from Pan troglodytes isolate Yerkes chimp pedigree #C0471 (Clint) chromosome 22, Clint_PTRv2 and can be accessed from the sequence ID: NC_036901.1, length: 33698415 bp. The "LOC102725072.start-FAM230F.start.18846286-18865042.NEW.ref" sequence again includes the *linc-UR-B1* sequence. The *BCRP2* sequence is the reverse complement. Alignments are by EMBL-EBI program at: https://www.ebi.ac.uk/Tools/msa/clustalo/.

```
CLUSTAL O(1.2.4) multiple sequence alignment


exon11.USP18.human                                                            ------------------------------------------------------------  0
LOC102725072.(18846286-18861064new.ref                                        ------------------------------------------------------------  0
LOC102725072.start-FAM230F.start.18846286-18865042.NEW.ref                    ------------------------------------------------------------  0
chimp.100000-160000.Pan.troglodytes.isolate.Yerkes.chimp.pedigree.#C0471      cagttatatcacaatagtaaatatcttaaacatggaaaagatacagtaaaaacatagtac  60
BCRP2.ref.rev.compl                                                           ------------------------------------------------------------  0

exon11.USP18.human                                                            ------------------------------------------------------------  0
LOC102725072.(18846286-18861064new.ref                                        ------------------------------------------------------------  0
LOC102725072.start-FAM230F.start.18846286-18865042.NEW.ref                    ------------------------------------------------------------  0
chimp.100000-160000.Pan.troglodytes.isolate.Yerkes.chimp.pedigree.#C0471      attggagaccgaggcgggcggatcacctgaggtcagaagttcgagaccagcctgaccaac  120
BCRP2.ref.rev.compl                                                           ------------------------------------------------------------  0

exon11.USP18.human                                                            ------------------------------------------------------------  0
LOC102725072.(18846286-18861064new.ref                                        ------------------------------------------------------------  0
LOC102725072.start-FAM230F.start.18846286-18865042.NEW.ref                    ------------------------------------------------------------  0
chimp.100000-160000.Pan.troglodytes.isolate.Yerkes.chimp.pedigree.#C0471      atggagaaactccgtctgtactaaaaatacaaaattagctgggatgctggcgtgcgcctg  180
BCRP2.ref.rev.compl                                                           ------------------------------------------------------------  0

exon11.USP18.human                                                            ------------------------------------------------------------  0
LOC102725072.(18846286-18861064new.ref                                        ------------------------------------------------------------  0
LOC102725072.start-FAM230F.start.18846286-18865042.NEW.ref                    ------------------------------------------------------------  0
chimp.100000-160000.Pan.troglodytes.isolate.Yerkes.chimp.pedigree.#C0471      taatcccagctactcgtgaggctgaggcaggagaatcacttgaaccccggaggcagaggt  240
BCRP2.ref.rev.compl                                                           ------------------------------------------------------------  0

exon11.USP18.human                                                            ------------------------------------------------------------  0
LOC102725072.(18846286-18861064new.ref                                        ------------------------------------------------------------  0
LOC102725072.start-FAM230F.start.18846286-18865042.NEW.ref                    ------------------------------------------------------------  0
chimp.100000-160000.Pan.troglodytes.isolate.Yerkes.chimp.pedigree.#C0471      tgcagtgagccaagatcgtgccattgcactccagcctggcaacaaaataaaactccttct  300
BCRP2.ref.rev.compl                                                           ------------------------------------------------------------  0

exon11.USP18.human                                                            ------------------------------------------------------------  0
LOC102725072.(18846286-18861064new.ref                                        ------------------------------------------------------------  0
LOC102725072.start-FAM230F.start.18846286-18865042.NEW.ref                    ------------------------------------------------------------  0
chimp.100000-160000.Pan.troglodytes.isolate.Yerkes.chimp.pedigree.#C0471      ccaaaaaagaatttaaaatatatatatattacaaaagttaagagagtggtacaccagtat  360
BCRP2.ref.rev.compl                                                           ------------------------------------------------------------  0

exon11.USP18.human                                                            ------------------------------------------------------------  0
LOC102725072.(18846286-18861064new.ref                                        ------------------------------------------------------------  0
LOC102725072.start-FAM230F.start.18846286-18865042.NEW.ref                    ------------------------------------------------------------  0
chimp.100000-160000.Pan.troglodytes.isolate.Yerkes.chimp.pedigree.#C0471      agggacacttagcatgaacagaggttgcaggactggcagttgctctcatgagtcgtgagt  420
BCRP2.ref.rev.compl                                                           ------------------------------------------------------------  0

exon11.USP18.human                                                            ------------------------------------------------------------  0
LOC102725072.(18846286-18861064new.ref                                        ------------------------------------------------------------  0
LOC102725072.start-FAM230F.start.18846286-18865042.NEW.ref                    ------------------------------------------------------------  0
chimp.100000-160000.Pan.troglodytes.isolate.Yerkes.chimp.pedigree.#C0471      acttggtgatgtgaatgaaaaggcctaggatattactgtacataactatagactttatat  480
BCRP2.ref.rev.compl                                                           ------------------------------------------------------------  0

exon11.USP18.human                                                            ------------------------------------------------------------  0
LOC102725072.(18846286-18861064new.ref                                        ------------------------------------------------------------  0
LOC102725072.start-FAM230F.start.18846286-18865042.NEW.ref                    ------------------------------------------------------------  0
chimp.100000-160000.Pan.troglodytes.isolate.Yerkes.chimp.pedigree.#C0471      gcactgtacacttaggctacactaaatgtatttaaatttttttcttctttaacaagttcgt  540
BCRP2.ref.rev.compl                                                           ------------------------------------------------------------  0

exon11.USP18.human                                                            ------------------------------------------------------------  0
LOC102725072.(18846286-18861064new.ref                                        ------------------------------------------------------------  0
LOC102725072.start-FAM230F.start.18846286-18865042.NEW.ref                    ------------------------------------------------------------  0
chimp.100000-160000.Pan.troglodytes.isolate.Yerkes.chimp.pedigree.#C0471      cttagcttatcataactttataaacttttaatttttaatttttgattctttcataata  600
BCRP2.ref.rev.compl                                                           ------------------------------------------------------------  0

exon11.USP18.human                                                            ------------------------------------------------------------  0
LOC102725072.(18846286-18861064new.ref                                        ------------------------------------------------------------  0
LOC102725072.start-FAM230F.start.18846286-18865042.NEW.ref                    ------------------------------------------------------------  0
chimp.100000-160000.Pan.troglodytes.isolate.Yerkes.chimp.pedigree.#C0471      acacatcttaaaaaaaaacacattgaacagctgtacagaaatactttatatccttatttg  660
BCRP2.ref.rev.compl                                                           ------------------------------------------------------------  0

exon11.USP18.human                                                            ------------------------------------------------------------  0
LOC102725072.(18846286-18861064new.ref                                        ------------------------------------------------------------  0
LOC102725072.start-FAM230F.start.18846286-18865042.NEW.ref                    ------------------------------------------------------------  0
chimp.100000-160000.Pan.troglodytes.isolate.Yerkes.chimp.pedigree.#C0471      ataagctttatttattttatttttatgtttttaaactttttgttaaaaactaagataca  720
BCRP2.ref.rev.compl                                                           ------------------------------------------------------------  0

exon11.USP18.human                                                            ------------------------------------------------------------  0
LOC102725072.(18846286-18861064new.ref                                        ------------------------------------------------------------  0
LOC102725072.start-FAM230F.start.18846286-18865042.NEW.ref                    ------------------------------------------------------------  0
chimp.100000-160000.Pan.troglodytes.isolate.Yerkes.chimp.pedigree.#C0471      aacacacacactagcttagccctgtaagggtcaggataatcaatatcactgtcttccatc  780
BCRP2.ref.rev.compl                                                           ------------------------------------------------------------  0

exon11.USP18.human                                                            ------------------------------------------------------------  0
LOC102725072.(18846286-18861064new.ref                                        ------------------------------------------------------------  0
LOC102725072.start-FAM230F.start.18846286-18865042.NEW.ref                    ------------------------------------------------------------  0
chimp.100000-160000.Pan.troglodytes.isolate.Yerkes.chimp.pedigree.#C0471      tccaaatcttctcccagtgaaaggtcttcagggtcttcagggcaataacatacatgcagc  840
BCRP2.ref.rev.compl                                                           ------------------------------------------------------------  0

exon11.USP18.human                                                            ------------------------------------------------------------  0
LOC102725072.(18846286-18861064new.ref                                        ------------------------------------------------------------  0
LOC102725072.start-FAM230F.start.18846286-18865042.NEW.ref                    ------------------------------------------------------------  0
chimp.100000-160000.Pan.troglodytes.isolate.Yerkes.chimp.pedigree.#C0471      tgtcattttctatgataacaaggctttcttctggaagaactcctgaaaagaccttcctga  900
BCRP2.ref.rev.compl                                                           ------------------------------------------------------------  0

exon11.USP18.human                                                            ------------------------------------------------------------  0
LOC102725072.(18846286-18861064new.ref                                        ------------------------------------------------------------  0
LOC102725072.start-FAM230F.start.18846286-18865042.NEW.ref                    ------------------------------------------------------------  0
chimp.100000-160000.Pan.troglodytes.isolate.Yerkes.chimp.pedigree.#C0471      ggctgttttaatagtaatttttttaatgagtagaagtactacactaaaatatgaataaaa  960
BCRP2.ref.rev.compl                                                           ------------------------------------------------------------  0
```

```
exon11.USP18.human                                                        ------------------------------------------------------------  0
LOC102725072.(18846286-18861064new.ref                                    ------------------------------------------------------------  0
LOC102725072.start-FAM230F.start.18846286-18865042.NEW.ref                ------------------------------------------------------------  0
chimp.100000-160000.Pan.troglodytes.isolate.Yerkes.chimp.pedigree.#C0471  tgtatagtattgtaaatactgttgatccttgaacagggcaagggttagggaccagctcct  1020
BCRP2.ref.rev.compl                                                       ------------------------------------------------------------  0

exon11.USP18.human                                                        ------------------------------------------------------------  0
LOC102725072.(18846286-18861064new.ref                                    ------------------------------------------------------------  0
LOC102725072.start-FAM230F.start.18846286-18865042.NEW.ref                ------------------------------------------------------------  0
chimp.100000-160000.Pan.troglodytes.isolate.Yerkes.chimp.pedigree.#C0471  gtgcagttgaaaatccatatataattctgggctatccccaaacttaatatacgatatata  1080
BCRP2.ref.rev.compl                                                       ------------------------------------------------------------  0

exon11.USP18.human                                                        ------------------------------------------------------------  0
LOC102725072.(18846286-18861064new.ref                                    ------------------------------------------------------------  0
LOC102725072.start-FAM230F.start.18846286-18865042.NEW.ref                ------------------------------------------------------------  0
chimp.100000-160000.Pan.troglodytes.isolate.Yerkes.chimp.pedigree.#C0471  gatatatattatatatgatatagatatatatatgatatatatgatatatactatatatat  1140
BCRP2.ref.rev.compl                                                       ------------------------------------------------------------  0

exon11.USP18.human                                                        ------------------------------------------------------------  0
LOC102725072.(18846286-18861064new.ref                                    ------------------------------------------------------------  0
LOC102725072.start-FAM230F.start.18846286-18865042.NEW.ref                ------------------------------------------------------------  0
chimp.100000-160000.Pan.troglodytes.isolate.Yerkes.chimp.pedigree.#C0471  atatatactatatatcaattaatgtatataaatatatatatataaatgatatatattata  1200
BCRP2.ref.rev.compl                                                       ------------------------------------------------------------  0

exon11.USP18.human                                                        ------------------------------------------------------------  0
LOC102725072.(18846286-18861064new.ref                                    ------------------------------------------------------------  0
LOC102725072.start-FAM230F.start.18846286-18865042.NEW.ref                ------------------------------------------------------------  0
chimp.100000-160000.Pan.troglodytes.isolate.Yerkes.chimp.pedigree.#C0471  tactaaatgatataaatatataatatatatatatttataataaatatatatatattata  1260
BCRP2.ref.rev.compl                                                       ------------------------------------------------------------  0

exon11.USP18.human                                                        ------------------------------------------------------------  0
LOC102725072.(18846286-18861064new.ref                                    ------------------------------------------------------------  0
LOC102725072.start-FAM230F.start.18846286-18865042.NEW.ref                ------------------------------------------------------------  0
chimp.100000-160000.Pan.troglodytes.isolate.Yerkes.chimp.pedigree.#C0471  ttatatatatatattatatatattatataaattatattatattatatataattatattat  1320
BCRP2.ref.rev.compl                                                       ------------------------------------------------------------  0

exon11.USP18.human                                                        ------------------------------------------------------------  0
LOC102725072.(18846286-18861064new.ref                                    ------------------------------------------------------------  0
LOC102725072.start-FAM230F.start.18846286-18865042.NEW.ref                ------------------------------------------------------------  0
chimp.100000-160000.Pan.troglodytes.isolate.Yerkes.chimp.pedigree.#C0471  tttatataatatatattaatatattatatatattatattatatattatattatattatat  1380
BCRP2.ref.rev.compl                                                       ------------------------------------------------------------  0

exon11.USP18.human                                                        ------------------------------------------------------------  0
LOC102725072.(18846286-18861064new.ref                                    ------------------------------------------------------------  0
LOC102725072.start-FAM230F.start.18846286-18865042.NEW.ref                ------------------------------------------------------------  0
chimp.100000-160000.Pan.troglodytes.isolate.Yerkes.chimp.pedigree.#C0471  ataatattttatatattaattatatattaatatttatataatattatatatatatatatt  1440
BCRP2.ref.rev.compl                                                       ------------------------------------------------------------  0

exon11.USP18.human                                                        ------------------------------------------------------------  0
LOC102725072.(18846286-18861064new.ref                                    ------------------------------------------------------------  0
LOC102725072.start-FAM230F.start.18846286-18865042.NEW.ref                ------------------------------------------------------------  0
chimp.100000-160000.Pan.troglodytes.isolate.Yerkes.chimp.pedigree.#C0471  atatataatattatatatgtaatatttatgatatttatatattattatattattataaat  1500
BCRP2.ref.rev.compl                                                       ------------------------------------------------------------  0

exon11.USP18.human                                                        ------------------------------------------------------------  0
LOC102725072.(18846286-18861064new.ref                                    ------------------------------------------------------------  0
LOC102725072.start-FAM230F.start.18846286-18865042.NEW.ref                ------------------------------------------------------------  0
chimp.100000-160000.Pan.troglodytes.isolate.Yerkes.chimp.pedigree.#C0471  aatatatataatatatattatataatatattatatataataattaaatatattattatat  1560
BCRP2.ref.rev.compl                                                       ------------------------------------------------------------  0

exon11.USP18.human                                                        ------------------------------------------------------------  0
LOC102725072.(18846286-18861064new.ref                                    ------------------------------------------------------------  0
LOC102725072.start-FAM230F.start.18846286-18865042.NEW.ref                ------------------------------------------------------------  0
chimp.100000-160000.Pan.troglodytes.isolate.Yerkes.chimp.pedigree.#C0471  atattattatattacatatataattatatattcttatattacacatataataatatacat  1620
BCRP2.ref.rev.compl                                                       ------------------------------------------------------------  0

exon11.USP18.human                                                        ------------------------------------------------------------  0
LOC102725072.(18846286-18861064new.ref                                    ------------------------------------------------------------  0
LOC102725072.start-FAM230F.start.18846286-18865042.NEW.ref                ------------------------------------------------------------  0
chimp.100000-160000.Pan.troglodytes.isolate.Yerkes.chimp.pedigree.#C0471  aattataattatatataattatacattatattatatataatattaatatataattatata  1680
BCRP2.ref.rev.compl                                                       ------------------------------------------------------------  0

exon11.USP18.human                                                        ------------------------------------------------------------  0
LOC102725072.(18846286-18861064new.ref                                    ------------------------------------------------------------  0
LOC102725072.start-FAM230F.start.18846286-18865042.NEW.ref                ------------------------------------------------------------  0
chimp.100000-160000.Pan.troglodytes.isolate.Yerkes.chimp.pedigree.#C0471  tattatattatatataattaattataattatattatatataatattatatataattatat  1740
BCRP2.ref.rev.compl                                                       ------------------------------------------------------------  0

exon11.USP18.human                                                        ------------------------------------------------------------  0
LOC102725072.(18846286-18861064new.ref                                    ------------------------------------------------------------  0
LOC102725072.start-FAM230F.start.18846286-18865042.NEW.ref                ------------------------------------------------------------  0
chimp.100000-160000.Pan.troglodytes.isolate.Yerkes.chimp.pedigree.#C0471  tatatatatgtattatatatattatattatatatatatgtattatatatattatattata  1800
BCRP2.ref.rev.compl                                                       ------------------------------------------------------------  0

exon11.USP18.human                                                        ------------------------------------------------------------  0
LOC102725072.(18846286-18861064new.ref                                    ------------------------------------------------------------  0
LOC102725072.start-FAM230F.start.18846286-18865042.NEW.ref                ------------------------------------------------------------  0
chimp.100000-160000.Pan.troglodytes.isolate.Yerkes.chimp.pedigree.#C0471  taatgtatatatattatattatatatatgtattatatattatattatatatgatgtatta  1860
BCRP2.ref.rev.compl                                                       ------------------------------------------------------------  0

exon11.USP18.human                                                        ------------------------------------------------------------  0
LOC102725072.(18846286-18861064new.ref                                    ------------------------------------------------------------  0
LOC102725072.start-FAM230F.start.18846286-18865042.NEW.ref                ------------------------------------------------------------  0
chimp.100000-160000.Pan.troglodytes.isolate.Yerkes.chimp.pedigree.#C0471  tatataatataaggatgcaggatgtaaaaggaaattttgctgtgtgcagatggaggcagt  1920
BCRP2.ref.rev.compl                                                       ------------------------------------------------------------  0

exon11.USP18.human                                                        ------------------------------------------------------------  0
LOC102725072.(18846286-18861064new.ref                                    ------------------------------------------------------------  0
LOC102725072.start-FAM230F.start.18846286-18865042.NEW.ref                ------------------------------------------------------------  0
chimp.100000-160000.Pan.troglodytes.isolate.Yerkes.chimp.pedigree.#C0471  ggctggagtgaatgaacggcaacacttgctggcaaccggcagaagctgagagacagggaa  1980
BCRP2.ref.rev.compl                                                       ------------------------------------------------------------  0

exon11.USP18.human                                                        ------------------------------------------------------------  0
LOC102725072.(18846286-18861064new.ref                                    ------------------------------------------------------------  0
LOC102725072.start-FAM230F.start.18846286-18865042.NEW.ref                ------------------------------------------------------------  0
chimp.100000-160000.Pan.troglodytes.isolate.Yerkes.chimp.pedigree.#C0471  caggctctcctccagagcctccaggagccaggcctttggacaccttgaatgtgggcttct  2040
BCRP2.ref.rev.compl                                                       ------------------------------------------------------------  0

exon11.USP18.human                                                        ------------------------------------------------------------  0
LOC102725072.(18846286-18861064new.ref                                    ------------------------------------------------------------  0
LOC102725072.start-FAM230F.start.18846286-18865042.NEW.ref                ------------------------------------------------------------  0
chimp.100000-160000.Pan.troglodytes.isolate.Yerkes.chimp.pedigree.#C0471  gggagaccatgcgtttctgttataagcagcccagtctctggcagtttttacggctgcccc  2100
BCRP2.ref.rev.compl                                                       ------------------------------------------------------------  0

exon11.USP18.human                                                        ------------------------------------------------------------  0
LOC102725072.(18846286-18861064new.ref                                    ------------------------------------------------------------  0
LOC102725072.start-FAM230F.start.18846286-18865042.NEW.ref                ------------------------------------------------------------  0
chimp.100000-160000.Pan.troglodytes.isolate.Yerkes.chimp.pedigree.#C0471  cgaacactcatctatacctgtctgaccaggtcaagctccaaggaagggattctctacata  2160
BCRP2.ref.rev.compl                                                       ------------------------------------------------------------  0

exon11.USP18.human                                                        ------------------------------------------------------------  0
LOC102725072.(18846286-18861064new.ref                                    ------------------------------------------------------------  0
LOC102725072.start-FAM230F.start.18846286-18865042.NEW.ref                ------------------------------------------------------------  0
chimp.100000-160000.Pan.troglodytes.isolate.Yerkes.chimp.pedigree.#C0471  tctacattgtttgcagattttacaataatcatttattcttgcatggctgatcattgttaa  2220
BCRP2.ref.rev.compl                                                       ------------------------------------------------------------  0
```

```
exon11.USP18.human                                                   ------------------------------------------------------------  0
LOC102725072.(18846286-18861064new.ref                               ------------------------------------------------------------  0
LOC102725072.start-FAM230F.start.18846286-18865042.NEW.ref           ------------------------------------------------------------  0
chimp.100000-160000.Pan.troglodytes.isolate.Yerkes.chimp.pedigree.#C0471   ccaatacaaataaaataataaagaaatgacccacatttatgttgggagtttgatctgcc  2280
BCRP2.ref.rev.compl                                                  ------------------------------------------------------------  0


exon11.USP18.human                                                   ------------------------------------------------------------  0
LOC102725072.(18846286-18861064new.ref                               ------------------------------------------------------------  0
LOC102725072.start-FAM230F.start.18846286-18865042.NEW.ref           ------------------------------------------------------------  0
chimp.100000-160000.Pan.troglodytes.isolate.Yerkes.chimp.pedigree.#C0471   atttatcaagtatggaatcttgaacaaggggttaaacatctgaatgtctccatcacttca  2340
BCRP2.ref.rev.compl                                                  ------------------------------------------------------------  0


exon11.USP18.human                                                   ------------------------------------------------------------  0
LOC102725072.(18846286-18861064new.ref                               ------------------------------------------------------------  0
LOC102725072.start-FAM230F.start.18846286-18865042.NEW.ref           ------------------------------------------------------------  0
chimp.100000-160000.Pan.troglodytes.isolate.Yerkes.chimp.pedigree.#C0471   tctctaaagtgggggtgctcacacccactgggctccccccacaggttggtgccggactct  2400
BCRP2.ref.rev.compl                                                  ------------------------------------------------------------  0


exon11.USP18.human                                                   ------------------------------------------------------------  0
LOC102725072.(18846286-18861064new.ref                               ------------------------------------------------------------  0
LOC102725072.start-FAM230F.start.18846286-18865042.NEW.ref           ------------------------------------------------------------  0
chimp.100000-160000.Pan.troglodytes.isolate.Yerkes.chimp.pedigree.#C0471   ccctgggccccccctgttctctcaccagccacatccattctcccccgaagggcgctagtga  2460
BCRP2.ref.rev.compl                                                  ------------------------------------------------------------  0


exon11.USP18.human                                                   ------------------------------------------------------------  0
LOC102725072.(18846286-18861064new.ref                               ------------------------------------------------------------  0
LOC102725072.start-FAM230F.start.18846286-18865042.NEW.ref           ------------------------------------------------------------  0
chimp.100000-160000.Pan.troglodytes.isolate.Yerkes.chimp.pedigree.#C0471   ctgtgcgtggctttccattcccaccacgtttgtctctaaccccagtggcagatcagtgta  2520
BCRP2.ref.rev.compl                                                  ------------------------------------------------------------  0


exon11.USP18.human                                                   ------------------------------------------------------------  0
LOC102725072.(18846286-18861064new.ref                               ------------------------------------------------------------  0
LOC102725072.start-FAM230F.start.18846286-18865042.NEW.ref           ------------------------------------------------------------  0
chimp.100000-160000.Pan.troglodytes.isolate.Yerkes.chimp.pedigree.#C0471   agaacacagctgagtgctcctcgcctcctagccccttcaagggctcctcaccacccacca  2580
BCRP2.ref.rev.compl                                                  ------------------------------------------------------------  0


exon11.USP18.human                                                   ------------------------------------------------------------  0
LOC102725072.(18846286-18861064new.ref                               gctcgcctctctccttccagtacctattgcatgaccccccacgtccgcctcccgccattg  60
LOC102725072.start-FAM230F.start.18846286-18865042.NEW.ref           gctcgcctctctccttccagtacctattgcatgaccccccacgtccgcctcccgccattg  60
chimp.100000-160000.Pan.troglodytes.isolate.Yerkes.chimp.pedigree.#C0471   gatcaggtgcaaacttccaagccttactggatccccttccacattctgagctctgcctgc  2640
BCRP2.ref.rev.compl                                                  ------------------------------------------------------------  0


exon11.USP18.human                                                   ------------------------------------------------------------  0
LOC102725072.(18846286-18861064new.ref                               ccagcaagcgcctcgcgcgggtacctggctgcg---------------------------  93
LOC102725072.start-FAM230F.start.18846286-18865042.NEW.ref           ccagcaagcgcctcgcgcgggtacctggctgcg---------------------------  93
chimp.100000-160000.Pan.troglodytes.isolate.Yerkes.chimp.pedigree.#C0471   cttcccatcgctatccttccccacctgcctccctggtagagaaaagcagagtgtgtgatg  2700
BCRP2.ref.rev.compl                                                  ------------------------------------------------------------  0


exon11.USP18.human                                                   ------------------------------------------------------------  0
LOC102725072.(18846286-18861064new.ref                               ------------------------------------------------------------  93
LOC102725072.start-FAM230F.start.18846286-18865042.NEW.ref           ------------------------------------------------------------  93
chimp.100000-160000.Pan.troglodytes.isolate.Yerkes.chimp.pedigree.#C0471   ctgtctgaatgctgagcacggccttttgcagccagtccactgtggacgctgcccctatca  2760
BCRP2.ref.rev.compl                                                  ------------------------------------------------------------  0


exon11.USP18.human                                                   ------------------------------------------------------------  0
LOC102725072.(18846286-18861064new.ref                               ------------------------------------------------------------  93
LOC102725072.start-FAM230F.start.18846286-18865042.NEW.ref           ------------------------------------------------------------  93
chimp.100000-160000.Pan.troglodytes.isolate.Yerkes.chimp.pedigree.#C0471   gagacctccaccttaaccctttccggcctggaggctcctcccagggccccacaacagaag  2820
BCRP2.ref.rev.compl                                                  ------------------------------------------------------------  0


exon11.USP18.human                                                   ------------------------------------------------------------  0
LOC102725072.(18846286-18861064new.ref                               ------------------------------------------------------------  93
LOC102725072.start-FAM230F.start.18846286-18865042.NEW.ref           ------------------------------------------------------------  93
chimp.100000-160000.Pan.troglodytes.isolate.Yerkes.chimp.pedigree.#C0471   tgacttcccttgcctttgaatttctatagcacaatccctactgccccccgttaaaactgc  2880
BCRP2.ref.rev.compl                                                  ------------------------------------------------------------  0


exon11.USP18.human                                                   ------------------------------------------------------------  0
LOC102725072.(18846286-18861064new.ref                               ------------------------------------------------------------  93
LOC102725072.start-FAM230F.start.18846286-18865042.NEW.ref           ------------------------------------------------------------  93
chimp.100000-160000.Pan.troglodytes.isolate.Yerkes.chimp.pedigree.#C0471   aaagtccttttgtggaaaataactttattcatgactgtgtttatcacactatcttatgga  2940
BCRP2.ref.rev.compl                                                  ------------------------------------------------------------  0


exon11.USP18.human                                                   ------------------------------------------------------------  0
LOC102725072.(18846286-18861064new.ref                               ------------------------------------------------------------  93
LOC102725072.start-FAM230F.start.18846286-18865042.NEW.ref           ------------------------------------------------------------  93
chimp.100000-160000.Pan.troglodytes.isolate.Yerkes.chimp.pedigree.#C0471   gaagagatgatcaataaatatttgctgaataaatgaatagcagttacaaaacacttgatt  3000
BCRP2.ref.rev.compl                                                  ------------------------------------------------------------  0


exon11.USP18.human                                                   ------------------------------------------------------------  0
LOC102725072.(18846286-18861064new.ref                               -------------------------------------------------cttattaatcc  104
LOC102725072.start-FAM230F.start.18846286-18865042.NEW.ref           -------------------------------------------------cttattaatcc  104
chimp.100000-160000.Pan.troglodytes.isolate.Yerkes.chimp.pedigree.#C0471   catatggaattaatgttggttctcaaagtgaaaaattacaaacagcactgatattcagcc  3060
BCRP2.ref.rev.compl                                                  ------------------------------------------------------------  0


exon11.USP18.human                                                   ------------------------------------------------------------  0
LOC102725072.(18846286-18861064new.ref                               gttaagctcgctctgtcacgggcgccgtgatgtgctcacgcgcccgctccctcaggttta  164
LOC102725072.start-FAM230F.start.18846286-18865042.NEW.ref           gttaagctcgctctgtcacgggcgccgtgatgtgctcacgcgcccgctccctcaggttta  164
chimp.100000-160000.Pan.troglodytes.isolate.Yerkes.chimp.pedigree.#C0471   agtatacaagtctggtcacagcagttgtataatactgaaatacccctgccactg-----  3115
BCRP2.ref.rev.compl                                                  ------------------------------------------------------------  0


exon11.USP18.human                                                   ------------------------------------------------------------  0
LOC102725072.(18846286-18861064new.ref                               aaaggcgcgttgcccggcaacagaagaaactgctggcttagccgttggccgagttggcgg  224
LOC102725072.start-FAM230F.start.18846286-18865042.NEW.ref           aaaggcgcgttgcccggcaacagaagaaactgctggcttagccgttggccgagttggcgg  224
chimp.100000-160000.Pan.troglodytes.isolate.Yerkes.chimp.pedigree.#C0471   ------------------------------------------------------------  3115
BCRP2.ref.rev.compl                                                  ------------------------------------------------------------  0


exon11.USP18.human                                                   ------------------------------------------------------------  0
LOC102725072.(18846286-18861064new.ref                               ctggacgaggacgctcagagcccagctctcgagagttcaagcaaccgacggttccccact  284
LOC102725072.start-FAM230F.start.18846286-18865042.NEW.ref           ctggacgaggacgctcagagcccagctctcgagagttcaagcaaccgacggttccccact  284
chimp.100000-160000.Pan.troglodytes.isolate.Yerkes.chimp.pedigree.#C0471   ------------------------------------------acctttggcccccagatg  3133
BCRP2.ref.rev.compl                                                  ------------------------------------------------------------  0


exon11.USP18.human                                                   ------------------------------------------------------------  0
LOC102725072.(18846286-18861064new.ref                               gctcccaggagcggttacctgggcactctgtgcccctccttcctgttcgggcccaggccg  344
LOC102725072.start-FAM230F.start.18846286-18865042.NEW.ref           gctcccaggagcggttacctgggcactctgtgcccctccttcctgttcgggcccaggccg  344
chimp.100000-160000.Pan.troglodytes.isolate.Yerkes.chimp.pedigree.#C0471   cctcccactgccactgctctccccactgggaacccctgaagttcccacaggctcataact  3193
BCRP2.ref.rev.compl                                                  ------------------------------------------------------------  0


exon11.USP18.human                                                   ------------------------------------------------------------  0
LOC102725072.(18846286-18861064new.ref                               aggacctgccagtagggctcagttgcctggagcccgttcagcccatcccccagttcactt  404
LOC102725072.start-FAM230F.start.18846286-18865042.NEW.ref           aggacctgccagtagggctcagttgcctggagcccgttcagcccatcccccagttcactt  404
chimp.100000-160000.Pan.troglodytes.isolate.Yerkes.chimp.pedigree.#C0471   aaagggctaatgtcttgcacagcagcgagcacccaggaccgagcagccacatggccgggt  3253
BCRP2.ref.rev.compl                                                  ------------------------------------------------------------  0


exon11.USP18.human                                                   ------------------------------------------------------------  0
LOC102725072.(18846286-18861064new.ref                               tgcctgtgggatctccccgttgctcctgcccgtggactgagtggc---------------  449
LOC102725072.start-FAM230F.start.18846286-18865042.NEW.ref           tgcctgtgggatctccccgttgctcctgcccgtggactgagtggc---------------  449
chimp.100000-160000.Pan.troglodytes.isolate.Yerkes.chimp.pedigree.#C0471   ctgctggtgaaagcatccattctgactgatcaggacctgaggggcctcatggttacatat  3313
BCRP2.ref.rev.compl                                                  ------------------------------------------------------------  0


exon11.USP18.human                                                   ------------------------------------------------------------  0
LOC102725072.(18846286-18861064new.ref                               ------------------------------------------------------------  449
LOC102725072.start-FAM230F.start.18846286-18865042.NEW.ref           ------------------------------------------------------------  449
```

```
chimp.100000-160000.Pan.troglodytes.isolate.Yerkes.chimp.pedigree.#C0471      tttgataatatccctaattataaataaggctcagttatatagtttgaaaacaatgcttct  3373
BCRP2.ref.rev.compl                                                            ------------------------------------------------------------  0


exon11.USP18.human                                                             ------------------------------------------------------------  0
LOC102725072.(18846286-18861064new.ref                                         ------------------------------------------------------------  449
LOC102725072.start-FAM230F.start.18846286-18865042.NEW.ref                     ------------------------------------------------------------  449
chimp.100000-160000.Pan.troglodytes.isolate.Yerkes.chimp.pedigree.#C0471      cctcattgcaaaatctcttagaagactccgtagatccaggaacggaaatggaaaatgaca  3433
BCRP2.ref.rev.compl                                                            ------------------------------------------------------------  0


exon11.USP18.human                                                             ------------------------------------------------------------  0
LOC102725072.(18846286-18861064new.ref                                         ------------------------------------------------------------  449
LOC102725072.start-FAM230F.start.18846286-18865042.NEW.ref                     ------------------------------------------------------------  449
chimp.100000-160000.Pan.troglodytes.isolate.Yerkes.chimp.pedigree.#C0471      gcgtgtcaatctctgaaggttttgggcatttccattagcactccatcttcatgtaaacca  3493
BCRP2.ref.rev.compl                                                            ------------------------------------------------------------  0


exon11.USP18.human                                                             ------------------------------------------------------------  0
LOC102725072.(18846286-18861064new.ref                                         ------------------------aggccatcctacaagcacccggacacttgacatcag  485
LOC102725072.start-FAM230F.start.18846286-18865042.NEW.ref                     ------------------------aggccatcctacaagcacccggacacttgacatcag  485
chimp.100000-160000.Pan.troglodytes.isolate.Yerkes.chimp.pedigree.#C0471      gaagatatgcagtttcctgcctagagagaagagaagacacatcagcacagcggcatgaaa  3553
BCRP2.ref.rev.compl                                                            ------------------------------------------------------------  0


exon11.USP18.human                                                             ------------------------------------------------------------  0
LOC102725072.(18846286-18861064new.ref                                         tggtgtcaagacaactctaagaaggttttccgtgatcctgcaagccctgccttccttcct  545
LOC102725072.start-FAM230F.start.18846286-18865042.NEW.ref                     tggtgtcaagacaactctaagaaggttttccgtgatcctgcaagccctgccttccttcct  545
chimp.100000-160000.Pan.troglodytes.isolate.Yerkes.chimp.pedigree.#C0471      ccttcatcagaaaacaatgcttcattaatccctgacaggacaagcgtcagcaaactt---  3610
BCRP2.ref.rev.compl                                                            ------------------------------------------------------------  0


exon11.USP18.human                                                             ------------------------------------------------------------  0
LOC102725072.(18846286-18861064new.ref                                         gggatcctgccttcaatttgattgcacaggtaccacagcaagccagtgctgtgtgctccg  605
LOC102725072.start-FAM230F.start.18846286-18865042.NEW.ref                     gggatcctgccttcaatttgattgcacaggtaccacagcaagccagtgctgtgtgctccg  605
chimp.100000-160000.Pan.troglodytes.isolate.Yerkes.chimp.pedigree.#C0471      ------------------------------------------------------------  3610
BCRP2.ref.rev.compl                                                            ------------------------------------------------------------  0


exon11.USP18.human                                                             ------------------------------------------------------------  0
LOC102725072.(18846286-18861064new.ref                                         agttccagggcgtcctccagctcagccactgcactgagaacatggactctctgtggggcc  665
LOC102725072.start-FAM230F.start.18846286-18865042.NEW.ref                     agttccagggcgtcctccagctcagccactgcactgagaacatggactctctgtggggcc  665
chimp.100000-160000.Pan.troglodytes.isolate.Yerkes.chimp.pedigree.#C0471      -----------------------------------------------------------c  3611
BCRP2.ref.rev.compl                                                            ------------------------------------------------------------  0


exon11.USP18.human                                                             ------------------------------------------------------------  0
LOC102725072.(18846286-18861064new.ref                                         caggagccgggagtcacccctttggggtccacaacacccggctgtccccagacttgtgtc  725
LOC102725072.start-FAM230F.start.18846286-18865042.NEW.ref                     caggagccgggagtcacccctttggggtccacaacacccggctgtccccagacttgtgtc  725
chimp.100000-160000.Pan.troglodytes.isolate.Yerkes.chimp.pedigree.#C0471      caggccactggattaagccttcatctatccatcaccttggagaggaacaaaataggtggc  3671
BCRP2.ref.rev.compl                                                            ------------------------------------------------------------  0


exon11.USP18.human                                                             ------------------------------------------------------------  0
LOC102725072.(18846286-18861064new.ref                                         cagggaagatagtgttgagggccctcaaggagagcggggcagggatgcctgagcaggaca  785
LOC102725072.start-FAM230F.start.18846286-18865042.NEW.ref                     cagggaagatagtgttgagggccctcaaggagagcggggcagggatgcctgagcaggaca  785
chimp.100000-160000.Pan.troglodytes.isolate.Yerkes.chimp.pedigree.#C0471      ctgggaagataagcactatgtttctattagttaatatctaaagcggaggttaacaagcta  3731
BCRP2.ref.rev.compl                                                            ------------------------------------------------------------  0


exon11.USP18.human                                                             ------------------------------------------------------------  0
LOC102725072.(18846286-18861064new.ref                                         aggaccctagagtccaagagaatcct-----ggtgatcagagaagggtccccgaggtcac  840
LOC102725072.start-FAM230F.start.18846286-18865042.NEW.ref                     aggaccctagagtccaagagaatcct-----ggtgatcagagaagggtccccgaggtcac  840
chimp.100000-160000.Pan.troglodytes.isolate.Yerkes.chimp.pedigree.#C0471      tggacacacaagccaaacccagccctcttggggtttttaaatctactttcaacttttat  3791
BCRP2.ref.rev.compl                                                            ------------------------------------------------------------  0


exon11.USP18.human                                                             ------------------------------------------------------------  0
LOC102725072.(18846286-18861064new.ref                                         cggggatgcaccgtctgcatttcggcccctgcgggacaatagaggcctctctccctttgt  900
LOC102725072.start-FAM230F.start.18846286-18865042.NEW.ref                     cggggatgcaccgtctgcatttcggcccctgcgggacaatagaggcctctctccctttgt  900
chimp.100000-160000.Pan.troglodytes.isolate.Yerkes.chimp.pedigree.#C0471      tttagattcagcggcacat--------gtgcaggtttgtcacgtggatatgagcatact  3843
BCRP2.ref.rev.compl                                                            ------------------------------------------------------------  0


exon11.USP18.human                                                             ------------------------------------------------------------  0
LOC102725072.(18846286-18861064new.ref                                         gcccgggcccgggcctctgcagacagacctccatgcccagaggtcagaaatcagatataa  960
LOC102725072.start-FAM230F.start.18846286-18865042.NEW.ref                     gcccgggcccgggcctctgcagacagacctccatgcccagaggtcagaaatcagatataa  960
chimp.100000-160000.Pan.troglodytes.isolate.Yerkes.chimp.pedigree.#C0471      ccccaacagttggcctttcacccctcccctccctccccatccagtagttcccagttgttg  3903
BCRP2.ref.rev.compl                                                            ------------------------------------------------------------  0


exon11.USP18.human                                                             ------------------------------------------------------------  0
LOC102725072.(18846286-18861064new.ref                                         ccagacatcccagacctcctggacgagctcctgcaccaaccgaaatgccatctccagctc  1020
LOC102725072.start-FAM230F.start.18846286-18865042.NEW.ref                     ccagacatcccagacctcctggacgagctcctgcaccaaccgaaatgccatctccagctc  1020
chimp.100000-160000.Pan.troglodytes.isolate.Yerkes.chimp.pedigree.#C0471      cca-------------------------tctttaagtcaatgagtccccatgtttagctc  3938
BCRP2.ref.rev.compl                                                            ------------------------------------------------------------  0


exon11.USP18.human                                                             ------------------------------------------------------------  0
LOC102725072.(18846286-18861064new.ref                                         ctacagctccacggga--------------------------------------------  1036
LOC102725072.start-FAM230F.start.18846286-18865042.NEW.ref                     ctacagctccacggga--------------------------------------------  1036
chimp.100000-160000.Pan.troglodytes.isolate.Yerkes.chimp.pedigree.#C0471      ccatttataagagagaatatgcattatgttttgtttggtttttgctgggttttttttttt  3998
BCRP2.ref.rev.compl                                                            ------------------------------------------------------------  0


exon11.USP18.human                                                             ------------------------------------------------------------  0
LOC102725072.(18846286-18861064new.ref                                         -----------------ggcttgccggggctaaagcggaggagggggccagcctcatccc  1079
LOC102725072.start-FAM230F.start.18846286-18865042.NEW.ref                     -----------------ggcttgccggggctaaagcggaggagggggccagcctcatccc  1079
chimp.100000-160000.Pan.troglodytes.isolate.Yerkes.chimp.pedigree.#C0471      tttaatggagtcttgccctgtagcccaggctagagt---gaagtggcacaatcttggctc  4055
BCRP2.ref.rev.compl                                                            ------------------------------------------------------------  0


exon11.USP18.human                                                             ------------------------------------------------------------  0
LOC102725072.(18846286-18861064new.ref                                         actgccagctgaccctcagttcctcaaagacagtgagtgaggacaggcctcaggctgtct  1139
LOC102725072.start-FAM230F.start.18846286-18865042.NEW.ref                     actgccagctgaccctcagttcctcaaagacagtgagtgaggacaggcctcaggctgtct  1139
chimp.100000-160000.Pan.troglodytes.isolate.Yerkes.chimp.pedigree.#C0471      actgcaacctccgcctcc------------------------------------------  4073
BCRP2.ref.rev.compl                                                            ------------------------------------------------------------  0


exon11.USP18.human                                                             ------------------------------------------------------------  0
LOC102725072.(18846286-18861064new.ref                                         cttcaggtcacacccagtgtgaaaaggcagcagatatagcaccagggcagacactcgccc  1199
LOC102725072.start-FAM230F.start.18846286-18865042.NEW.ref                     cttcaggtcacacccagtgtgaaaaggcagcagatatagcaccagggcagacactcgccc  1199
chimp.100000-160000.Pan.troglodytes.isolate.Yerkes.chimp.pedigree.#C0471      ---caggttcaaacaattctccctcctcagcctccggagtagctgggactacaggtgccc  4130
BCRP2.ref.rev.compl                                                            ------------------------------------------------------------  0


exon11.USP18.human                                                             ------------------------------------------------------------  0
LOC102725072.(18846286-18861064new.ref                                         tcaggaatgac-------------------------------------------------  1210
LOC102725072.start-FAM230F.start.18846286-18865042.NEW.ref                     tcaggaatgac-------------------------------------------------  1210
chimp.100000-160000.Pan.troglodytes.isolate.Yerkes.chimp.pedigree.#C0471      gccaccacgcccagctaacttttgtatttttagtagagaaagggtttcaccgtgttagc  4190
BCRP2.ref.rev.compl                                                            ------------------------------------------------------------  0


exon11.USP18.human                                                             ------------------------------------------------------------  0
LOC102725072.(18846286-18861064new.ref                                         -----tcctccacatccgaggcctctaggcccagtacacacaagtttcccctgctgccac  1265
LOC102725072.start-FAM230F.start.18846286-18865042.NEW.ref                     -----tcctccacatccgaggcctctaggcccagtacacacaagtttcccctgctgccac  1265
chimp.100000-160000.Pan.troglodytes.isolate.Yerkes.chimp.pedigree.#C0471      caggatggtctcaatctcctgacctcatgatctgcccacctcagtctcccaaagtgc---  4247
BCRP2.ref.rev.compl                                                            ------------------------------------------------------------  0


exon11.USP18.human                                                             ------------------------------------------------------------  0
LOC102725072.(18846286-18861064new.ref                                         gcaggcgaggggagcctttgatgctgccacctcccttagagctggggtaccgggtcactg  1325
LOC102725072.start-FAM230F.start.18846286-18865042.NEW.ref                     gcaggcgaggggagcctttgatgctgccacctcccttagagctggggtaccgggtcactg  1325
chimp.100000-160000.Pan.troglodytes.isolate.Yerkes.chimp.pedigree.#C0471      -------agggattacaggcatgagccaccgtgcccagccttttgtttattttttgacga  4300
BCRP2.ref.rev.compl                                                            ------------------------------------------------------------  0


exon11.USP18.human                                                             ------------------------------------------------------------  0
LOC102725072.(18846286-18861064new.ref                                         ttgaagacctggaccgggagaaggaggcggcattccagcgcatcaacagtgcactgcagg  1385
LOC102725072.start-FAM230F.start.18846286-18865042.NEW.ref                     ttgaagacctggaccgggagaaggaggcggcattccagcgcatcaacagtgcactgcagg  1385
chimp.100000-160000.Pan.troglodytes.isolate.Yerkes.chimp.pedigree.#C0471      gaccgttcttgctctgtcaccaggctggagtgcactggcacagtaatagctcaccacagc  4360
BCRP2.ref.rev.compl                                                            ------------------------------------------------------------  0


exon11.USP18.human                                                             ------------------------------------------------------------  0
```

```
LOC102725072.(18846286-18861064new.ref                                       ttgaggacaaggccatctcggactgcagaccctcacgaccttcccacactttgtcctcac  1445
LOC102725072.start-FAM230F.start.18846286-18865042.NEW.ref                   ttgaggacaaggccatctcggactgcagaccctcacgaccttcccacactttgtcctcac  1445
chimp.100000-160000.Pan.troglodytes.isolate.Yerkes.chimp.pedigree.#C0471     ctcgtgctcctgggctc-----------aactgaccctcctgcctcagttttagcttcct  4409
BCRP2.ref.rev.compl                                                          ------------------------------------------------------------  0

exon11.USP18.human                                                           ------------------------------------------------------------  0
LOC102725072.(18846286-18861064new.ref                                       ttgcaacagggacttctggtctg-cctgccatttctaaagcacccagtatggatgtacag  1504
LOC102725072.start-FAM230F.start.18846286-18865042.NEW.ref                   ttgcaacagggacttctggtctg-cctgccatttctaaagcacccagtatggatgtacag  1504
chimp.100000-160000.Pan.troglodytes.isolate.Yerkes.chimp.pedigree.#C0471     gagtagctaggactacgggtgtgtaccaccatgcctagctataataatttttattttttt  4469
BCRP2.ref.rev.compl                                                          ------------------------------------------------------------  0

exon11.USP18.human                                                           ------------------------------------------------------------  0
LOC102725072.(18846286-18861064new.ref                                       caggagacacacaagtcccaagactgcctgggcctactggccccccttagcatctgctgca  1564
LOC102725072.start-FAM230F.start.18846286-18865042.NEW.ref                   caggagacacacaagtcccaagactgcctgggcctactggccccccttagcatctgctgca  1564
chimp.100000-160000.Pan.troglodytes.isolate.Yerkes.chimp.pedigree.#C0471     gtagagatggagtcttgctttgttgcccaggctggtcttgaactcctggcttaaagtgat  4529
BCRP2.ref.rev.compl                                                          ------------------------------------------------------------  0

exon11.USP18.human                                                           ------------------------------------------------------------  0
LOC102725072.(18846286-18861064new.ref                                       ggtgtcccctctacagctcccatgtctgggaagaa-------------------------  1599
LOC102725072.start-FAM230F.start.18846286-18865042.NEW.ref                   ggtgtcccctctacagctcccatgtctgggaagaa-------------------------  1599
chimp.100000-160000.Pan.troglodytes.isolate.Yerkes.chimp.pedigree.#C0471     cctcctgcctcggcctcccaaagtgctgggattaaaggtgtgagatcacacccagtctcc  4589
BCRP2.ref.rev.compl                                                          ------------------------------------------------------------  0

exon11.USP18.human                                                           ------------------------------------------------------------  0
LOC102725072.(18846286-18861064new.ref                                       ------------------------------------------------------------  1599
LOC102725072.start-FAM230F.start.18846286-18865042.NEW.ref                   ------------------------------------------------------------  1599
chimp.100000-160000.Pan.troglodytes.isolate.Yerkes.chimp.pedigree.#C0471     aaccctctttttgcaagtaaatgtaactggaccccagccatgctcatctgcccatgtact  4649
BCRP2.ref.rev.compl                                                          ------------------------------------------------------------  0

exon11.USP18.human                                                           ------------------------------------------------------------  0
LOC102725072.(18846286-18861064new.ref                                       ------------------------------------------------------gcacaa  1605
LOC102725072.start-FAM230F.start.18846286-18865042.NEW.ref                   ------------------------------------------------------gcacaa  1605
chimp.100000-160000.Pan.troglodytes.isolate.Yerkes.chimp.pedigree.#C0471     gtctacggctgcttttgctctatagggcagagttaagtggttgcaacagacactgcacag  4709
BCRP2.ref.rev.compl                                                          ------------------------------------------------------------  0

exon11.USP18.human                                                           ------------------------------------------------------------  0
LOC102725072.(18846286-18861064new.ref                                       accaccaggccccctgttctcctcctcagatccccttcctgccacctcttcccattccca  1665
LOC102725072.start-FAM230F.start.18846286-18865042.NEW.ref                   accaccaggccccctgttctcctcctcagatccccttcctgccacctcttcccattccca  1665
chimp.100000-160000.Pan.troglodytes.isolate.Yerkes.chimp.pedigree.#C0471     accacaaagtctgaagtactttctctccagccctttacagagaaagtctgccaacctcta  4769
BCRP2.ref.rev.compl                                                          ------------------------------------------------------------  0

exon11.USP18.human                                                           ------------------------------------------------------------  0
LOC102725072.(18846286-18861064new.ref                                       ggactcagcccaggtca-------------------------------------------  1682
LOC102725072.start-FAM230F.start.18846286-18865042.NEW.ref                   ggactcagcccaggtca-------------------------------------------  1682
chimp.100000-160000.Pan.troglodytes.isolate.Yerkes.chimp.pedigree.#C0471     atctcaataacagggaaatcaatgacaaccacaaagtgacaaagattgggtgtctaagat  4829
BCRP2.ref.rev.compl                                                          ------------------------------------------------------------  0

exon11.USP18.human                                                           ------------------------------------------------------------  0
LOC102725072.(18846286-18861064new.ref                                       ------------------------------------------------------------  1682
LOC102725072.start-FAM230F.start.18846286-18865042.NEW.ref                   ------------------------------------------------------------  1682
chimp.100000-160000.Pan.troglodytes.isolate.Yerkes.chimp.pedigree.#C0471     ggatggtcagaataaacaagagagaaagatgaaaagtagaaggaggatttcaagcgcaag  4889
BCRP2.ref.rev.compl                                                          ------------------------------------------------------------  0

exon11.USP18.human                                                           ------------------------------------------------------------  0
LOC102725072.(18846286-18861064new.ref                                       cctcgctgattcctgccccttcccagctgcaagcatggatgtgggcatgagaagaacaa  1742
LOC102725072.start-FAM230F.start.18846286-18865042.NEW.ref                   cctcgctgattcctgccccttcccagctgcaagcatggatgtgggcatgagaagaacaa  1742
chimp.100000-160000.Pan.troglodytes.isolate.Yerkes.chimp.pedigree.#C0471     cttcacctaatccgttatttttcaaatgaccaggcctatctctgtagctgaaaatcacct  4949
BCRP2.ref.rev.compl                                                          ------------------------------------------------------------  0

exon11.USP18.human                                                           ------------------------------------------------------------  0
LOC102725072.(18846286-18861064new.ref                                       ggtgtggcacttctgctcctgcagctgccgcagcagcc----------------------  1780
LOC102725072.start-FAM230F.start.18846286-18865042.NEW.ref                   ggtgtggcacttctgctcctgcagctgccgcagcagcc----------------------  1780
chimp.100000-160000.Pan.troglodytes.isolate.Yerkes.chimp.pedigree.#C0471     caaataggatctctgatatacagtctcaaagctcagccaagaaacttacaaagtctctct  5009
BCRP2.ref.rev.compl                                                          ------------------------------------------------------------  0

exon11.USP18.human                                                           ------------------------------------------------------------  0
LOC102725072.(18846286-18861064new.ref                                       ------cctcccccctccacattgaaccccacgttggggtcactactgga-gtggatgga  1833
LOC102725072.start-FAM230F.start.18846286-18865042.NEW.ref                   ------cctcccccctccacattgaaccccacgttggggtcactactgga-gtggatgga  1833
chimp.100000-160000.Pan.troglodytes.isolate.Yerkes.chimp.pedigree.#C0471     gccttaacttcatccacctttttctctccagcttctcctcggtagttaatgattataaa  5069
BCRP2.ref.rev.compl                                                          ------------------------------------------------------------  0

exon11.USP18.human                                                           ------------------------------------------------------------  0
LOC102725072.(18846286-18861064new.ref                                       ggcccttcacatttctgggcctcagccacagctgcagcaggtgcccagaggtcagaacca  1893
LOC102725072.start-FAM230F.start.18846286-18865042.NEW.ref                   ggcccttcacatttctgggcctcagccacagctgcagcaggtgcccagaggtcagaacca  1893
chimp.100000-160000.Pan.troglodytes.isolate.Yerkes.chimp.pedigree.#C0471     aatatttattggctcatgcctgtaatcccagcactttgaggcgggcagatcacgaggtca  5129
BCRP2.ref.rev.compl                                                          ------------------------------------------------------------  0

exon11.USP18.human                                                           ------------------------------------------------------------  0
LOC102725072.(18846286-18861064new.ref                                       gagatcccagacctcccggaccagctcg--------tgccccaaacgaaatgccatctcg  1945
LOC102725072.start-FAM230F.start.18846286-18865042.NEW.ref                   gagatcccagacctcccggaccagctcg--------tgccccaaacgaaatgccatctcg  1945
chimp.100000-160000.Pan.troglodytes.isolate.Yerkes.chimp.pedigree.#C0471     ggagatcgagaccatcctggctaacacggtgaaatccgtctctactaaaaatacaaaaaa  5189
BCRP2.ref.rev.compl                                                          ------------------------------------------------------------  0

exon11.USP18.human                                                           ------------------------------------------------------------  0
LOC102725072.(18846286-18861064new.ref                                       agcccctaccgctctacgggaggcctcccggaacgaaagcggagaaggggccagcctca  2005
LOC102725072.start-FAM230F.start.18846286-18865042.NEW.ref                   agcccctaccgctctacgggaggcctcccggaacgaaagcggagaaggggccagcctca  2005
chimp.100000-160000.Pan.troglodytes.isolate.Yerkes.chimp.pedigree.#C0471     ttagccaggcgtgttggcgggagcctgtaatcccagctactcgggaggctgaggcaggag  5249
BCRP2.ref.rev.compl                                                          ------------------------------------------------------------  0

exon11.USP18.human                                                           ------------------------------------------------------------  0
LOC102725072.(18846286-18861064new.ref                                       tcccactgccagctgaacctcagttcctgaaacacagtgagtgaggac----ggacctca  2061
LOC102725072.start-FAM230F.start.18846286-18865042.NEW.ref                   tcccactgccagctgaacctcagttcctgaaacacagtgagtgaggac----ggacctca  2061
chimp.100000-160000.Pan.troglodytes.isolate.Yerkes.chimp.pedigree.#C0471     aatggcgtgaacccacaaggcagagcttgcaatgaggtgagatcccactactgcactcca  5309
BCRP2.ref.rev.compl                                                          ------------------------------------------------------------  0

exon11.USP18.human                                                           ------------------------------------------------------------  0
LOC102725072.(18846286-18861064new.ref                                       ggctgtctcttcgggtcacacccagtgtgaaaagatggcagatacagcaccagggcagac  2121
LOC102725072.start-FAM230F.start.18846286-18865042.NEW.ref                   ggctgtctcttcgggtcacacccagtgtgaaaagatggcagatacagcaccagggcagac  2121
chimp.100000-160000.Pan.troglodytes.isolate.Yerkes.chimp.pedigree.#C0471     gcctgggcgacagagcaagactccatctcaaaacaaaccaacaaacaaacaaaaaaacag  5369
BCRP2.ref.rev.compl                                                          ------------------------------------------------------------  0

exon11.USP18.human                                                           ------------------------------------------------------------  0
LOC102725072.(18846286-18861064new.ref                                       actcgcccccaggggtgg-----ctcccccagatcccaggcctctaggccctgtagatgc  2176
LOC102725072.start-FAM230F.start.18846286-18865042.NEW.ref                   actcgcccccaggggtgg-----ctcccccagatcccaggcctctaggccctgtagatgc  2176
chimp.100000-160000.Pan.troglodytes.isolate.Yerkes.chimp.pedigree.#C0471     tgtgatggccaggcgtggtgctcatgcctataatccaagcactttgggaggctgaaatgg  5429
BCRP2.ref.rev.compl                                                          ------------------------------------------------------------  0

exon11.USP18.human                                                           ------------------------------------------------------------  0
LOC102725072.(18846286-18861064new.ref                                       aagtttcccctgctgccacgcaggcgaggggagcctttgatgctgccacctcc-------  2229
LOC102725072.start-FAM230F.start.18846286-18865042.NEW.ref                   aagtttcccctgctgccacgcaggcgaggggagcctttgatgctgccacctcc-------  2229
chimp.100000-160000.Pan.troglodytes.isolate.Yerkes.chimp.pedigree.#C0471     atggatggcttgagcccagtagtttgagacaagcctggcaacatagcgagacctcatctc  5489
BCRP2.ref.rev.compl                                                          ------------------------------------------------------------  0

exon11.USP18.human                                                           ------------------------------------------------------------  0
LOC102725072.(18846286-18861064new.ref                                       ------------------------------------------------------------  2229
LOC102725072.start-FAM230F.start.18846286-18865042.NEW.ref                   ------------------------------------------------------------  2229
chimp.100000-160000.Pan.troglodytes.isolate.Yerkes.chimp.pedigree.#C0471     tacaaacatttttaaaatatgccaggcatggtggtgcatgccgtagtcccagctattcgg  5549
BCRP2.ref.rev.compl                                                          ------------------------------------------------------------  0

exon11.USP18.human                                                           ------------------------------------------------------------  0
LOC102725072.(18846286-18861064new.ref                                       ---------------------------------------------cttagagctggggta  2244
LOC102725072.start-FAM230F.start.18846286-18865042.NEW.ref                   ---------------------------------------------cttagagctggggta  2244
chimp.100000-160000.Pan.troglodytes.isolate.Yerkes.chimp.pedigree.#C0471     gaggctgaggtgggaggatcacctgtgcccgggagttcaaggctgcagtgagctatgatc  5609
BCRP2.ref.rev.compl                                                          ------------------------------------------------------------  0
```

```
exon11.USP18.human                                                          ------------------------------------------------------------  0
LOC102725072.(18846286-18861064new.ref                                      ccgggtcactgctgaagacctggaccaggagaaggaggc---------------------  2283
LOC102725072.start-FAM230F.start.18846286-18865042.NEW.ref                  ccgggtcactgctgaagacctggaccaggagaaggaggc---------------------  2283
chimp.100000-160000.Pan.troglodytes.isolate.Yerkes.chimp.pedigree.#C0471    acaccacagtgctccagcctgggcaacaaagcaagactccatctctaaaaataaaataaa  5669
BCRP2.ref.rev.compl                                                         ------------------------------------------------------------  0

exon11.USP18.human                                                          ------------------------------------------------------------  0
LOC102725072.(18846286-18861064new.ref                                      ------------------------------------------------------------  2283
LOC102725072.start-FAM230F.start.18846286-18865042.NEW.ref                  ------------------------------------------------------------  2283
chimp.100000-160000.Pan.troglodytes.isolate.Yerkes.chimp.pedigree.#C0471    atttaaaacaaagatcttcgctgtaaaagaggtacgctcaaatgcaataaaagcatataa  5729
BCRP2.ref.rev.compl                                                         ------------------------------------------------------------  0

exon11.USP18.human                                                          ------------------------------------------------------------  0
LOC102725072.(18846286-18861064new.ref                                      ---------------------------ggctttccagcgcatcaagagtgcactgcaggt  2316
LOC102725072.start-FAM230F.start.18846286-18865042.NEW.ref                  ---------------------------ggctttccagcgcatcaagagtgcactgcaggt  2316
chimp.100000-160000.Pan.troglodytes.isolate.Yerkes.chimp.pedigree.#C0471    gaaggccgggtgtggtggctcatgcctgtaatcccagcactttgggaggccgagacgggc  5789
BCRP2.ref.rev.compl                                                         ------------------------------------------------------------  0

exon11.USP18.human                                                          ------------------------------------------------------------  0
LOC102725072.(18846286-18861064new.ref                                      tgaggacaaggccatttagtactgcagaccct------cacggccttcccacactttgtc  2370
LOC102725072.start-FAM230F.start.18846286-18865042.NEW.ref                  tgaggacaaggccatttagtactgcagaccct------cacggccttcccacactttgtc  2370
chimp.100000-160000.Pan.troglodytes.isolate.Yerkes.chimp.pedigree.#C0471    ggatcacgaggtcaggagatcgagactatcctggctaacgcggagaaaccccatctcctc  5849
BCRP2.ref.rev.compl                                                         ------------------------------------------------------------  0

exon11.USP18.human                                                          ------------------------------------------------------------  0
LOC102725072.(18846286-18861064new.ref                                      ctcacttgcaacagaggcttctggtctgcctgccatttctaaagcacccagtatggatgc  2430
LOC102725072.start-FAM230F.start.18846286-18865042.NEW.ref                  ctcacttgcaacagaggcttctggtctgcctgccatttctaaagcacccagtatggatgc  2430
chimp.100000-160000.Pan.troglodytes.isolate.Yerkes.chimp.pedigree.#C0471    taaaagtacaaaaaaattagctgggctaggtg----------------------------  5881
BCRP2.ref.rev.compl                                                         ------------------------------------------------------------  0

exon11.USP18.human                                                          ------------------------------------------------------------  0
LOC102725072.(18846286-18861064new.ref                                      acagcaggagagacacaagtcccaagactgcctgggcctagtggcccccagcatctgct  2490
LOC102725072.start-FAM230F.start.18846286-18865042.NEW.ref                  acagcaggagagacacaagtcccaagactgcctgggcctagtggcccccagcatctgct  2490
chimp.100000-160000.Pan.troglodytes.isolate.Yerkes.chimp.pedigree.#C0471    ------------------------------------------------------------  5881
BCRP2.ref.rev.compl                                                         ------------------------------------------------------------  0

exon11.USP18.human                                                          ------------------------------------------------------------  0
LOC102725072.(18846286-18861064new.ref                                      gcacaggcctgtagtcccagctactcaggaggctgaggcaggagaagggcataaacccgg  2550
LOC102725072.start-FAM230F.start.18846286-18865042.NEW.ref                  gcacaggcctgtagtcccagctactcaggaggctgaggcaggagaagggcataaacccgg  2550
chimp.100000-160000.Pan.troglodytes.isolate.Yerkes.chimp.pedigree.#C0471    gcaggcgcctgtagtcccagctactcaggaggctgaggcaggagaatggcataaacccgg  5941
BCRP2.ref.rev.compl                                                         ------------------------------------------------------------  0
```

start of identity of LOC102725072 in chimp sequence

```
exon11.USP18.human                                                          ------------------------------------------------------------  0
LOC102725072.(18846286-18861064new.ref                                      gaggcagagcttgcagtgagctgagatcgcgccactgcactccagcctgggtgacagagc  2610
LOC102725072.start-FAM230F.start.18846286-18865042.NEW.ref                  gaggcagagcttgcagtgagctgagatcgcgccactgcactccagcctgggtgacagagc  2610
chimp.100000-160000.Pan.troglodytes.isolate.Yerkes.chimp.pedigree.#C0471    gaggcagagcttgcagtgagcccagatcacaccactgcactccagcctgggtgacagagc  6001
BCRP2.ref.rev.compl                                                         ------------------------------------------------------------  0

exon11.USP18.human                                                          ------------------------------------------------------------  0
LOC102725072.(18846286-18861064new.ref                                      gagactccgtctcaaaaaaaaagaaaaagaaaa-------aaaagttattgtgacatttc  2663
LOC102725072.start-FAM230F.start.18846286-18865042.NEW.ref                  gagactccgtctcaaaaaaaaagaaaaagaaaa-------aaaagttattgtgacatttc  2663
chimp.100000-160000.Pan.troglodytes.isolate.Yerkes.chimp.pedigree.#C0471    gagactccgtctcaaaaaaaaaaaaaaagaaaaagaaaagaaaagttcttgtgacatttg  6061
BCRP2.ref.rev.compl                                                         ------------------------------------------------------------  0

exon11.USP18.human                                                          ------------------------------------------------------------  0
LOC102725072.(18846286-18861064new.ref                                      tgtatgaaatcagccttcactacatggataggaccagcacgcttctgcggcacaactctg  2723
LOC102725072.start-FAM230F.start.18846286-18865042.NEW.ref                  tgtatgaaatcagccttcactacatggataggaccagcacgcttctgcggcacaactctg  2723
chimp.100000-160000.Pan.troglodytes.isolate.Yerkes.chimp.pedigree.#C0471    tgtatgaaatcagccttcactacatggataggaccagcacacttccgcggcacgactctg  6121
BCRP2.ref.rev.compl                                                         ------------------------------------------------------------  0

exon11.USP18.human                                                          ------------------------------------------------------------  0
LOC102725072.(18846286-18861064new.ref                                      caatcatactacattttttt----tttgtatttttttttattccttttgagacagagtct  2779
LOC102725072.start-FAM230F.start.18846286-18865042.NEW.ref                  caatcatactacattttttt----tttgtatttttttttattccttttgagacagagtct  2779
chimp.100000-160000.Pan.troglodytes.isolate.Yerkes.chimp.pedigree.#C0471    caatcttactacatttttttttttactttatatttatttattccttttgagacagagtct  6181
BCRP2.ref.rev.compl                                                         ------------------------------------------------------------  0

exon11.USP18.human                                                          ------------------------------------------------------------  0
LOC102725072.(18846286-18861064new.ref                                      cactctgtcacccaggctgaagtgcagccgagatctcggctcactgcaacctccacctcc  2839
LOC102725072.start-FAM230F.start.18846286-18865042.NEW.ref                  cactctgtcacccaggctgaagtgcagccgagatctcggctcactgcaacctccacctcc  2839
chimp.100000-160000.Pan.troglodytes.isolate.Yerkes.chimp.pedigree.#C0471    cactctgtcacccaggctgaagtgcagccgagatctcggctcactgcaacctccacctcc  6241
BCRP2.ref.rev.compl                                                         ------------------------------------------------------------  0

exon11.USP18.human                                                          ------------------------------------------------------------  0
LOC102725072.(18846286-18861064new.ref                                      tgggttcaagcaattctcctgtctcagcctcccaagtagctgggactacaggcacacgtc  2899
LOC102725072.start-FAM230F.start.18846286-18865042.NEW.ref                  tgggttcaagcaattctcctgtctcagcctcccaagtagctgggactacaggcacacgtc  2899
chimp.100000-160000.Pan.troglodytes.isolate.Yerkes.chimp.pedigree.#C0471    tgggttcaagcaattctcttgtctcagcctcccaagtagctgggactacaggcacacgtc  6301
BCRP2.ref.rev.compl                                                         ------------------------------------------------------------  0

exon11.USP18.human                                                          ------------------------------------------------------------  0
LOC102725072.(18846286-18861064new.ref                                      aaaaggcctggctaatttttgtatttttagtagagatggagttttgccatattggtcagg  2959
LOC102725072.start-FAM230F.start.18846286-18865042.NEW.ref                  aaaaggcctggctaatttttgtatttttagtagagatggagttttgccatattggtcagg  2959
chimp.100000-160000.Pan.troglodytes.isolate.Yerkes.chimp.pedigree.#C0471    aaaacgcccggctaatttttgtatttttagtagagatggagttttgccatattggtcagg  6361
BCRP2.ref.rev.compl                                                         ------------------------------------------------------------  0

exon11.USP18.human                                                          ------------------------------------------------------------  0
LOC102725072.(18846286-18861064new.ref                                      ctggtctcgaactcctgacctcaggtgatctacctgtcttagcctcccgaagtgctagga  3019
LOC102725072.start-FAM230F.start.18846286-18865042.NEW.ref                  ctggtctcgaactcctgacctcaggtgatctacctgtcttagcctcccgaagtgctagga  3019
chimp.100000-160000.Pan.troglodytes.isolate.Yerkes.chimp.pedigree.#C0471    ctggtctcgaactcctgacctcaggtgatcgacctgtcttagcctcccaaagtgctagga  6421
BCRP2.ref.rev.compl                                                         ------------------------------------------------------------  0

exon11.USP18.human                                                          ------------------------------------------------------------  0
LOC102725072.(18846286-18861064new.ref                                      ttacaggtgtatgtttatttatttatttaagatggaatcttgctctgtatttattaattt  3079
LOC102725072.start-FAM230F.start.18846286-18865042.NEW.ref                  ttacaggtgtatgtttatttatttatttaagatggaatcttgctctgtatttattaattt  3079
chimp.100000-160000.Pan.troglodytes.isolate.Yerkes.chimp.pedigree.#C0471    ttacaggtgtacatttatttatttatttgagatggaatcttgctctgtatttattaattt  6481
BCRP2.ref.rev.compl                                                         ------------------------------------------------------------  0

exon11.USP18.human                                                          ------------------------------------------------------------  0
LOC102725072.(18846286-18861064new.ref                                      atttagttgagatggagtcttgctccatcacccaggctagggtgcagtggtgcaatctcg  3139
LOC102725072.start-FAM230F.start.18846286-18865042.NEW.ref                  atttagttgagatggagtcttgctccatcacccaggctagggtgcagtggtgcaatctcg  3139
chimp.100000-160000.Pan.troglodytes.isolate.Yerkes.chimp.pedigree.#C0471    atttatttgagatggagtcttgctccatcgcccaggctagagtgcagtggtgcaatctcg  6541
BCRP2.ref.rev.compl                                                         ------------------------------------------------------------  0

exon11.USP18.human                                                          ------------------------------------------------------------  0
LOC102725072.(18846286-18861064new.ref                                      gctcactgcaacctctgacttccagtttcaagcgattctcctgcctcagtgtcccaagta  3199
LOC102725072.start-FAM230F.start.18846286-18865042.NEW.ref                  gctcactgcaacctctgacttccagtttcaagcgattctcctgcctcagtgtcccaagta  3199
chimp.100000-160000.Pan.troglodytes.isolate.Yerkes.chimp.pedigree.#C0471    gctcattgcaacctctgccttccaggttcaagcgattctcctgcctcagtgtcccaagta  6601
BCRP2.ref.rev.compl                                                         ------------------------------------------------------------  0

exon11.USP18.human                                                          ------------------------------------------------------------  0
LOC102725072.(18846286-18861064new.ref                                      gctgggattacaggtgcctgccaccacagctggctaatttttgtatttttagtagagaca  3259
LOC102725072.start-FAM230F.start.18846286-18865042.NEW.ref                  gctgggattacaggtgcctgccaccacagctggctaatttttgtatttttagtagagaca  3259
chimp.100000-160000.Pan.troglodytes.isolate.Yerkes.chimp.pedigree.#C0471    gctgggattacaggtgcctgccaccacagctggctaatttttgtatttttagtagagaca  6661
BCRP2.ref.rev.compl                                                         ------------------------------------------------------------  0

exon11.USP18.human                                                          ------------------------------------------------------------  0
LOC102725072.(18846286-18861064new.ref                                      gtgtttcaccatcttggccaggctggtctcgggctcctgacctcatgaaccacctgcctc  3319
LOC102725072.start-FAM230F.start.18846286-18865042.NEW.ref                  gtgtttcaccatcttggccaggctggtctcgggctcctgacctcatgaaccacctgcctc  3319
chimp.100000-160000.Pan.troglodytes.isolate.Yerkes.chimp.pedigree.#C0471    gtgtttcaccatcttggccaggctggtctcgggctcctgacctcatgaaccacctgcctc  6721
BCRP2.ref.rev.compl                                                         ------------------------------------------------------------  0

exon11.USP18.human                                                          ------------------------------------------------------------  0
LOC102725072.(18846286-18861064new.ref                                      agcctcccaaagtgttgggattacaggcctaaggcaccatgctcggcca----tatttat  3375
LOC102725072.start-FAM230F.start.18846286-18865042.NEW.ref                  agcctcccaaagtgttgggattacaggcctaaggcaccatgctcggcca----tatttat  3375
```

```
chimp.100000-160000.Pan.troglodytes.isolate.Yerkes.chimp.pedigree.#C0471      agcctcccaaagtgttgggattacaggcctaaggcaccatgctcggccatatttatttat  6781
BCRP2.ref.rev.compl                                                           ------------------------------------------------------------  0

exon11.USP18.human                                                            ------------------------------------------------------------  0
LOC102725072.(18846286-18861064new.ref                                        ttaattatttagagacaaagtcttgctctgtcacccaggctggagtgcagtggcgccatc  3435
LOC102725072.start-FAM230F.start.18846286-18865042.NEW.ref                    ttaattatttagagacaaagtcttgctctgtcacccaggctggagtgcagtggcgccatc  3435
chimp.100000-160000.Pan.troglodytes.isolate.Yerkes.chimp.pedigree.#C0471      ttaattatttagagacaaagtcttgctctgtcacccaggctggagtgcagtggcgccatc  6841
BCRP2.ref.rev.compl                                                           ------------------------------------------------------------  0

exon11.USP18.human                                                            ------------------------------------------------------------  0
LOC102725072.(18846286-18861064new.ref                                        tcagcttactgcagcctccgtctctgaggtttaagcgattctcatgcctcagcctcctga  3495
LOC102725072.start-FAM230F.start.18846286-18865042.NEW.ref                    tcagcttactgcagcctccgtctctgaggtttaagcgattctcatgcctcagcctcctga  3495
chimp.100000-160000.Pan.troglodytes.isolate.Yerkes.chimp.pedigree.#C0471      tcagctcactgcagcctccgcctctgaggtttaagcgattctcatgcctcagcctcctga  6901
BCRP2.ref.rev.compl                                                           ------------------------------------------------------------  0

exon11.USP18.human                                                            ------------------------------------------------------------  0
LOC102725072.(18846286-18861064new.ref                                        gtaactgggactacaggtactcaccaccatgcagggatatttttttctattgttttatag  3555
LOC102725072.start-FAM230F.start.18846286-18865042.NEW.ref                    gtaactgggactacaggtactcaccaccatgcagggatatttttttctattgttttatag  3555
chimp.100000-160000.Pan.troglodytes.isolate.Yerkes.chimp.pedigree.#C0471      gtaactaggactacagatactcgccaccacgcagggatttttttttctatttttttgtag  6961
BCRP2.ref.rev.compl                                                           ------------------------------------------------------------  0

exon11.USP18.human                                                            ------------------------------------------------------------  0
LOC102725072.(18846286-18861064new.ref                                        agacacggtttcaccatattggccaggctggtctcgaactcctgaccttaggtgatctga  3615
LOC102725072.start-FAM230F.start.18846286-18865042.NEW.ref                    agacacggtttcaccatattggccaggctggtctcgaactcctgaccttaggtgatctga  3615
chimp.100000-160000.Pan.troglodytes.isolate.Yerkes.chimp.pedigree.#C0471      agacatagtttcaccatgttggccaggctggtctcgaactcctgaccttaggtgatctga  7021
BCRP2.ref.rev.compl                                                           ------------------------------------------------------------  0

exon11.USP18.human                                                            ------------------------------------------------------------  0
LOC102725072.(18846286-18861064new.ref                                        cagcctcgtcctctcaaagcactgggattacaggcatgagccgccaagcccggcctctca  3675
LOC102725072.start-FAM230F.start.18846286-18865042.NEW.ref                    cagcctcgtcctctcaaagcactgggattacaggcatgagccgccaagcccggcctctca  3675
chimp.100000-160000.Pan.troglodytes.isolate.Yerkes.chimp.pedigree.#C0471      cagcctcgtcctctctaagtactgggattacaggcatgagcc-ccttgcccggcctctca  7080
BCRP2.ref.rev.compl                                                           ------------------------------------------------------------  0

exon11.USP18.human                                                            ------------------------------------------------------------  0
LOC102725072.(18846286-18861064new.ref                                        ctacatttaagtgacaccatggctcatgcctgtaatcctagcactttgggaggccaaggc  3735
LOC102725072.start-FAM230F.start.18846286-18865042.NEW.ref                    ctacatttaagtgacaccatggctcatgcctgtaatcctagcactttgggaggccaaggc  3735
chimp.100000-160000.Pan.troglodytes.isolate.Yerkes.chimp.pedigree.#C0471      ctacatttaagtgacgccatggctcgtgcctgtaatcctagcactttgggaggccaaggc  7140
BCRP2.ref.rev.compl                                                           ------------------------------------------------------------  0

exon11.USP18.human                                                            ------------------------------------------------------------  0
LOC102725072.(18846286-18861064new.ref                                        aggtggatcacctgatgtcaggagttcgaaacgagcctggccaacatggggaaaccccgt  3795
LOC102725072.start-FAM230F.start.18846286-18865042.NEW.ref                    aggtggatcacctgatgtcaggagttcgaaacgagcctggccaacatggggaaaccccgt  3795
chimp.100000-160000.Pan.troglodytes.isolate.Yerkes.chimp.pedigree.#C0471      aggtggatcacctgaggtcaggagttcgacacgagcctggccaacatggggaaaccccgt  7200
BCRP2.ref.rev.compl                                                           ------------------------------------------------------------  0

exon11.USP18.human                                                            ------------------------------------------------------------  0
LOC102725072.(18846286-18861064new.ref                                        ctctagtaaaaatacaaaaattagtcaggtgtggtggtacaagcctgtaggcccagctac  3855
LOC102725072.start-FAM230F.start.18846286-18865042.NEW.ref                    ctctagtaaaaatacaaaaattagtcaggtgtggtggtacaagcctgtaggcccagctac  3855
chimp.100000-160000.Pan.troglodytes.isolate.Yerkes.chimp.pedigree.#C0471      ctctagtaaaaatacaaaaattagtcacgtgtggtggtacaagcctgtaggcccagctac  7260
BCRP2.ref.rev.compl                                                           ------------------------------------------------------------  0

exon11.USP18.human                                                            ------------------------------------------------------------  0
LOC102725072.(18846286-18861064new.ref                                        ttggaagactgaggcaggagaatcactttaagcgggaggcagaggttgcagtgagccaat  3915
LOC102725072.start-FAM230F.start.18846286-18865042.NEW.ref                    ttggaagactgaggcaggagaatcactttaagcgggaggcagaggttgcagtgagccaat  3915
chimp.100000-160000.Pan.troglodytes.isolate.Yerkes.chimp.pedigree.#C0471      ttggaagactgaggcaggagaatcactttaactgggaggcagaggttgcagtgagccaat  7320
BCRP2.ref.rev.compl                                                           ------------------------------------------------------------  0

exon11.USP18.human                                                            ------------------------------------------------------------  0
LOC102725072.(18846286-18861064new.ref                                        ctc-----------------------------------------------aaaaaagaaa  3928
LOC102725072.start-FAM230F.start.18846286-18865042.NEW.ref                    ctc-----------------------------------------------aaaaaagaaa  3928
chimp.100000-160000.Pan.troglodytes.isolate.Yerkes.chimp.pedigree.#C0471      atcatgccactgcactccagcttgggtgacagagtgagacactgtctcaaaaaaaaaaaa  7380
BCRP2.ref.rev.compl                                                           ------------------------------------------------------------  0

exon11.USP18.human                                                            ------------------------------------------------------------  0
LOC102725072.(18846286-18861064new.ref                                        gaaaaaaaaaaagaaaaacatatgatgctggggcatctcggcctcaatacctgcatgagc  3988
LOC102725072.start-FAM230F.start.18846286-18865042.NEW.ref                    gaaaaaaaaaaagaaaaacatatgatgctggggcatctcggcctcaatacctgcatgagc  3988
chimp.100000-160000.Pan.troglodytes.isolate.Yerkes.chimp.pedigree.#C0471      gaaaaaaaaagagaaaaaaatatgatgccggggcatctcggcctcaatacctgcgtgagc  7440
BCRP2.ref.rev.compl                                                           ------------------------------------------------------------  0

exon11.USP18.human                                                            ------------------------------------------------------------  0
LOC102725072.(18846286-18861064new.ref                                        acagtcacgtccaggccagggctgctggtcgaggtccggccccatctcttccagcagaaa  4048
LOC102725072.start-FAM230F.start.18846286-18865042.NEW.ref                    acagtcacgtccaggccagggctgctggtcgaggtccggccccatctcttccagcagaaa  4048
chimp.100000-160000.Pan.troglodytes.isolate.Yerkes.chimp.pedigree.#C0471      acagtcatgtccaggccagggctgctgatcgaggtccggccccatctctcccagcagaaa  7500
BCRP2.ref.rev.compl                                                           ------------------------------------------------------------  0

exon11.USP18.human                                                            ------------------------------------------------------------  0
LOC102725072.(18846286-18861064new.ref                                        gggagtaagcttgcagggaggctggggacaagatcccaggatctcagcctctgctcatg  4108
LOC102725072.start-FAM230F.start.18846286-18865042.NEW.ref                    gggagtaagcttgcagggaggctggggacaagatcccaggatctcagcctctgctcatg  4108
chimp.100000-160000.Pan.troglodytes.isolate.Yerkes.chimp.pedigree.#C0471      gggagtaagcttacagggcggctggggacaatatcccaggatctcggcctctgctcatg  7560
BCRP2.ref.rev.compl                                                           ------------------------------------------------------------  0

exon11.USP18.human                                                            ------------------------------------------------------------  0
LOC102725072.(18846286-18861064new.ref                                        gatcagctctgagaccccgagtgagctgggggtgctctgtgcgcattggtttccccagct  4168
LOC102725072.start-FAM230F.start.18846286-18865042.NEW.ref                    gatcagctctgagaccccgagtgagctgggggtgctctgtgcgcattggtttccccagct  4168
chimp.100000-160000.Pan.troglodytes.isolate.Yerkes.chimp.pedigree.#C0471      gatcagctctgagaccccgagtgagctgggggtgctctgtgcgcattggtttccccagct  7620
BCRP2.ref.rev.compl                                                           ------------------------------------------------------------  0

exon11.USP18.human                                                            ------------------------------------------------------------  0
LOC102725072.(18846286-18861064new.ref                                        gtcaagtaaagggattggatgaggaagtcttgtcaaggtggaatgatctcagatttgggg  4228
LOC102725072.start-FAM230F.start.18846286-18865042.NEW.ref                    gtcaagtaaagggattggatgaggaagtcttgtcaaggtggaatgatctcagatttgggg  4228
chimp.100000-160000.Pan.troglodytes.isolate.Yerkes.chimp.pedigree.#C0471      gtcaagtaaagggattggatgaggaagtcttgtcaaggtggaatgatctcagatttgggg  7680
BCRP2.ref.rev.compl                                                           ------------------------------------------------------------  0

exon11.USP18.human                                                            ------------------------------------------------------------  0
LOC102725072.(18846286-18861064new.ref                                        cagcagtgaatgatcccgctccctgggcccatgccagtggcctggcctcggctcaacaca  4288
LOC102725072.start-FAM230F.start.18846286-18865042.NEW.ref                    cagcagtgaatgatcccgctccctgggcccatgccagtggcctggcctcggctcaacaca  4288
chimp.100000-160000.Pan.troglodytes.isolate.Yerkes.chimp.pedigree.#C0471      cagcagtgaatgatcccgcttcctggg-ccatgccagtggcccggcctcggctgaacaca  7739
BCRP2.ref.rev.compl                                                           ------------------------------------------------------------  0

exon11.USP18.human                                                            ------------------------------------------------------------  0
LOC102725072.(18846286-18861064new.ref                                        gccccaacactctggaatggggatgagggggcagtcagctcttgctcctagtaagagaga  4348
LOC102725072.start-FAM230F.start.18846286-18865042.NEW.ref                    gccccaacactctggaatggggatgagggggcagtcagctcttgctcctagtaagagaga  4348
chimp.100000-160000.Pan.troglodytes.isolate.Yerkes.chimp.pedigree.#C0471      gccccaacactctggaatggggatgagggagcagtcagctcttgctcctattaagagaga  7799
BCRP2.ref.rev.compl                                                           ------------------------------------------------------------  0

exon11.USP18.human                                                            ------------------------------------------------------------  0
LOC102725072.(18846286-18861064new.ref                                        tgcaacagggctctgtggctgagctgggtgccttgcctcacacctgtaatcccaaccttt  4408
LOC102725072.start-FAM230F.start.18846286-18865042.NEW.ref                    tgcaacagggctctgtggctgagctgggtgccttgcctcacacctgtaatcccaaccttt  4408
chimp.100000-160000.Pan.troglodytes.isolate.Yerkes.chimp.pedigree.#C0471      tgcaacagggctctgtggctgagctgggtgccttgcctcacacctgtaatcccaaccttt  7859
BCRP2.ref.rev.compl                                                           ------------------------------------------------------------  0

exon11.USP18.human                                                            ------------------------------------------------------------  0
LOC102725072.(18846286-18861064new.ref                                        gagaggccaaggcaggaggattgctcgaggccgggaattttgagaatagcctggacaaca  4468
LOC102725072.start-FAM230F.start.18846286-18865042.NEW.ref                    gagaggccaaggcaggaggattgctcgaggccgggaattttgagaatagcctggacaaca  4468
chimp.100000-160000.Pan.troglodytes.isolate.Yerkes.chimp.pedigree.#C0471      gagaggccgaggcaggaggattgcttgaggctgggaattttgagaatagcctggacaaca  7919
BCRP2.ref.rev.compl                                                           ------------------------------------------------------------  0

exon11.USP18.human                                                            ------------------------------------------------------------  0
LOC102725072.(18846286-18861064new.ref                                        tagccagaccccatgtctacaaaataataataaaacacacagctatagtccaagctactt  4528
LOC102725072.start-FAM230F.start.18846286-18865042.NEW.ref                    tagccagaccccatgtctacaaaataataataaaacacacagctatagtccaagctactt  4528
chimp.100000-160000.Pan.troglodytes.isolate.Yerkes.chimp.pedigree.#C0471      tagccagacaccatgtctacaaaataataataaaacatacagctatagtccaagctactt  7979
BCRP2.ref.rev.compl                                                           ------------------------------------------------------------  0

exon11.USP18.human                                                            ------------------------------------------------------------  0
```

```
LOC102725072.(18846286-18861064new.ref                                ggcaggctgaggcaggaaggtcccttgagtccgggaattggaggctgcattgagctataa  4588
LOC102725072.start-FAM230F.start.18846286-18865042.NEW.ref            ggcaggctgaggcaggaaggtcccttgagtccgggaattggaggctgcattgagctataa  4588
chimp.100000-160000.Pan.troglodytes.isolate.Yerkes.chimp.pedigree.#C0471   agcaggctgaggcaggaaggtcccttgagtccaggaattggaggctgcattgagctataa  8039
BCRP2.ref.rev.compl                                                   ------------------------------------------------------------  0

exon11.USP18.human                                                    ------------------------------------------------------------  0
LOC102725072.(18846286-18861064new.ref                                tcgcaccactgcactccagcttgggtgacaaagtgagaccctgtctctaaaagaaaaaaa  4648
LOC102725072.start-FAM230F.start.18846286-18865042.NEW.ref            tcgcaccactgcactccagcttgggtgacaaagtgagaccctgtctctaaaagaaaaaaa  4648
chimp.100000-160000.Pan.troglodytes.isolate.Yerkes.chimp.pedigree.#C0471   tcgcaccactgcactccagcttgggtgacaaagtgagaccctgtctctaaaagaaaaaaa  8099
BCRP2.ref.rev.compl                                                   ------------------------------------------------------------  0

exon11.USP18.human                                                    ------------------------------------------------------------  0
LOC102725072.(18846286-18861064new.ref                                aattggcctgtgagcatgggtttgattttcaaacaggacctggagggtagggacagacag  4708
LOC102725072.start-FAM230F.start.18846286-18865042.NEW.ref            aattggcctgtgagcatgggtttgattttcaaacaggacctggagggtagggacagacag  4708
chimp.100000-160000.Pan.troglodytes.isolate.Yerkes.chimp.pedigree.#C0471   aattggcctgtgagcatgggtttgattttcaaacaggacccagtgggtagggacagacag  8159
BCRP2.ref.rev.compl                                                   ------------------------------------------------------------  0

exon11.USP18.human                                                    ------------------------------------------------------------  0
LOC102725072.(18846286-18861064new.ref                                tgctgtcacccttaggtgctgaacactcagaaacgggccagcggcagcccttccctcacc  4768
LOC102725072.start-FAM230F.start.18846286-18865042.NEW.ref            tgctgtcacccttaggtgctgaacactcagaaacgggccagcggcagcccttccctcacc  4768
chimp.100000-160000.Pan.troglodytes.isolate.Yerkes.chimp.pedigree.#C0471   tgctgtcacccttaggtgctgaacactcagaaatgggccagcggcagccctttcctcacc  8219
BCRP2.ref.rev.compl                                                   ------------------------------------------------------------  0

exon11.USP18.human                                                    ------------------------------------------------------------  0
LOC102725072.(18846286-18861064new.ref                                tgcagacaccagattgggcagaacagcacatggcacttgcagctcttgcagtgagggcag  4828
LOC102725072.start-FAM230F.start.18846286-18865042.NEW.ref            tgcagacaccagattgggcagaacagcacatggcacttgcagctcttgcagtgagggcag  4828
chimp.100000-160000.Pan.troglodytes.isolate.Yerkes.chimp.pedigree.#C0471   tgcagacaccagattgggcagaacagcacgtggcacttgcagctcttgcagtgagggcag  8279
BCRP2.ref.rev.compl                                                   ------------------------------------------------------------  0

exon11.USP18.human                                                    ------------------------------------------------------------  0
LOC102725072.(18846286-18861064new.ref                                aacccagtgtcaacccttctgcctgtgggaggggctgctgaggcctgcggagaggccagg  4888
LOC102725072.start-FAM230F.start.18846286-18865042.NEW.ref            aacccagtgtcaacccttctgcctgtgggaggggctgctgaggcctgcggagaggccagg  4888
chimp.100000-160000.Pan.troglodytes.isolate.Yerkes.chimp.pedigree.#C0471   aacccagtgtcaacccttctgcctgtgggaggggctgctgaggcctgcggagaggccagg  8339
BCRP2.ref.rev.compl                                                   ------------------------------------------------------------  0

exon11.USP18.human                                                    ------------------------------------------------------------  0
LOC102725072.(18846286-18861064new.ref                                gtggaggctcgtccccttgtccagcccttggcgtggtctccaccaggtccccagcccacc  4948
LOC102725072.start-FAM230F.start.18846286-18865042.NEW.ref            gtggaggctcgtccccttgtccagcccttggcgtggtctccaccaggtccccagcccacc  4948
chimp.100000-160000.Pan.troglodytes.isolate.Yerkes.chimp.pedigree.#C0471   gtggaggctcgtccccttgtccagcccttggcatggtctccaccaggtccccagcccacc  8399
BCRP2.ref.rev.compl                                                   ------------------------------------------------------------  0

exon11.USP18.human                                                    ------------------------------------------------------------  0
LOC102725072.(18846286-18861064new.ref                                agtgcagggcgcccctgagcctgctgctgccatgggccctgtctctacccaggacgtccc  5008
LOC102725072.start-FAM230F.start.18846286-18865042.NEW.ref            agtgcagggcgcccctgagcctgctgctgccatgggccctgtctctacccaggacgtccc  5008
chimp.100000-160000.Pan.troglodytes.isolate.Yerkes.chimp.pedigree.#C0471   agtgcagggcgcccctgagcctgctgctgccacgggccctgtctctacccaggacgtccc  8459
BCRP2.ref.rev.compl                                                   ------------------------------------------------------------  0

exon11.USP18.human                                                    ------------------------------------------------------------  0
LOC102725072.(18846286-18861064new.ref                                cacaccctcgcagtgtcagggaaatgatcatggtggcggtgacactccgcaggcagggct  5068
LOC102725072.start-FAM230F.start.18846286-18865042.NEW.ref            cacaccctcgcagtgtcagggaaatgatcatggtggcggtgacactccgcaggcagggct  5068
chimp.100000-160000.Pan.troglodytes.isolate.Yerkes.chimp.pedigree.#C0471   cacaccctcgcagtgtcagggaaatgatcatggtggcggtgacactccgcaggcagggct  8519
BCRP2.ref.rev.compl                                                   ------------------------------------------------------------  0

exon11.USP18.human                                                    ------------------------------------------------------------  0
LOC102725072.(18846286-18861064new.ref                                gctgagagaagctgagaagggtcacactgcaggcaggggcccgtgtgacaagcccctctc  5128
LOC102725072.start-FAM230F.start.18846286-18865042.NEW.ref            gctgagagaagctgagaagggtcacactgcaggcaggggcccgtgtgacaagcccctctc  5128
chimp.100000-160000.Pan.troglodytes.isolate.Yerkes.chimp.pedigree.#C0471   gcggagagaagctgagaagggtcacactgcaggcagaggcccgtgtgacaagcccctctc  8579
BCRP2.ref.rev.compl                                                   ------------------------------------------------------------  0

exon11.USP18.human                                                    ------------------------------------------------------------  0
LOC102725072.(18846286-18861064new.ref                                accccgagagagctgaccaggcagctcacgagcagagccacatcccgggagtccgagaaa  5188
LOC102725072.start-FAM230F.start.18846286-18865042.NEW.ref            accccgagagagctgaccaggcagctcacgagcagagccacatcccgggagtccgagaaa  5188
chimp.100000-160000.Pan.troglodytes.isolate.Yerkes.chimp.pedigree.#C0471   accctgagagagctgaccaggcagctcatgagcagagccacaacccgggagtccgagaaa  8639
BCRP2.ref.rev.compl                                                   ------------------------------------------------------------  0

exon11.USP18.human                                                    ------------------------------------------------------------  0
LOC102725072.(18846286-18861064new.ref                                ggtcctggctgggctcagccacctcattggccacgggcagcctttgtcatgtgagccttg  5248
LOC102725072.start-FAM230F.start.18846286-18865042.NEW.ref            ggtcctggctgggctcagccacctcattggccacgggcagcctttgtcatgtgagccttg  5248
chimp.100000-160000.Pan.troglodytes.isolate.Yerkes.chimp.pedigree.#C0471   ggtcctggctgggctcagccacctcactggccagggcagcctttgtcgtgtgagccttg  8699
BCRP2.ref.rev.compl                                                   ------------------------------------------------------------  0

exon11.USP18.human                                                    ------------------------------------------------------------  0
LOC102725072.(18846286-18861064new.ref                                ctctcctggggaggctcaggctgacagctgatgtgggcattgccgaaggtaacccgtggc  5308
LOC102725072.start-FAM230F.start.18846286-18865042.NEW.ref            ctctcctggggaggctcaggctgacagctgatgtgggcattgccgaaggtaacccgtggc  5308
chimp.100000-160000.Pan.troglodytes.isolate.Yerkes.chimp.pedigree.#C0471   ctctcctggggaggctcaggctgacggctgatgtgggcattgccgagggtaacctgtggc  8759
BCRP2.ref.rev.compl                                                   ------------------------------------------------------------  0

exon11.USP18.human                                                    ------------------------------------------------------------  0
LOC102725072.(18846286-18861064new.ref                                ccagtgtatatggccgggtctcctcaagctgcattcattcaagtaggacccagggtgcgt  5368
LOC102725072.start-FAM230F.start.18846286-18865042.NEW.ref            ccagtgtatatggccgggtctcctcaagctgcattcattcaagtaggacccagggtgcgt  5368
chimp.100000-160000.Pan.troglodytes.isolate.Yerkes.chimp.pedigree.#C0471   ccagtggatatggctgggtctcctcaagctgcattcattcaagtaggacccagggtgcgt  8819
BCRP2.ref.rev.compl                                                   ------------------------------------------------------------  0

exon11.USP18.human                                                    ------------------------------------------------------------  0
LOC102725072.(18846286-18861064new.ref                                gcccatctccagcccagggcagctcccctgtaagctgggtgagctactgaagccaaggcg  5428
LOC102725072.start-FAM230F.start.18846286-18865042.NEW.ref            gcccatctccagcccagggcagctcccctgtaagctgggtgagctactgaagccaaggcg  5428
chimp.100000-160000.Pan.troglodytes.isolate.Yerkes.chimp.pedigree.#C0471   gcccatctccagcccagggcagctcccctgtaagctgggtgagctactgaagccaaggcg  8879
BCRP2.ref.rev.compl                                                   ------------------------------------------------------------  0

exon11.USP18.human                                                    ------------------------------------------------------------  0
LOC102725072.(18846286-18861064new.ref                                ggaggcagctgacaacacccacagcccatgcggaggtggtggaaaggctgaactcagcag  5488
LOC102725072.start-FAM230F.start.18846286-18865042.NEW.ref            ggaggcagctgacaacacccacagcccatgcggaggtggtggaaaggctgaactcagcag  5488
chimp.100000-160000.Pan.troglodytes.isolate.Yerkes.chimp.pedigree.#C0471   ggaggcagctgacaacacccacggcccatgcagagctggtggaaaggctggactcagcag  8939
BCRP2.ref.rev.compl                                                   ------------------------------------------------------------  0

exon11.USP18.human                                                    ------------------------------------------------------------  0
LOC102725072.(18846286-18861064new.ref                                caacaccaaatcctggaccaggcaaaaaccacccaagactgaggggctcgtgccagagcg  5548
LOC102725072.start-FAM230F.start.18846286-18865042.NEW.ref            caacaccaaatcctggaccaggcaaaaaccacccaagactgaggggctcgtgccagagcg  5548
chimp.100000-160000.Pan.troglodytes.isolate.Yerkes.chimp.pedigree.#C0471   caacaccaaatcccggaccaggcagaaaccacccaagactgaggggctcgtgccagagcg  8999
BCRP2.ref.rev.compl                                                   ------------------------------------------------------------  0

exon11.USP18.human                                                    ------------------------------------------------------------  0
LOC102725072.(18846286-18861064new.ref                                gtggccacaggtaagaacccgggcccaggctgtgtggcaggaatcctccatgtcccaggg  5608
LOC102725072.start-FAM230F.start.18846286-18865042.NEW.ref            gtggccacaggtaagaacccgggcccaggctgtgtggcaggaatcctccatgtcccaggg  5608
chimp.100000-160000.Pan.troglodytes.isolate.Yerkes.chimp.pedigree.#C0471   gtggccacaggtaagaacctgggcccaggctgtgtggcaggaatcctccatgtcccaggg  9059
BCRP2.ref.rev.compl                                                   ------------------------------------------------------------  0

exon11.USP18.human                                                    ------------------------------------------------------------  0
LOC102725072.(18846286-18861064new.ref                                cttagcatagcaaaggaagaccagccgggtcaccctggtggccatctgtccctgtcccac  5668
LOC102725072.start-FAM230F.start.18846286-18865042.NEW.ref            cttagcatagcaaaggaagaccagccgggtcaccctggtggccatctgtccctgtcccac  5668
chimp.100000-160000.Pan.troglodytes.isolate.Yerkes.chimp.pedigree.#C0471   cttagcatagcaaaggaagaccagccgggtcaccctggtggccatctgtccctgtcccac  9119
BCRP2.ref.rev.compl                                                   ------------------------------------------------------------  0

exon11.USP18.human                                                    ------------------------------------------------------------  0
LOC102725072.(18846286-18861064new.ref                                ctgcagagtcagaacagcctctccccagtggggatcatctctctctgccaaagcaacagc  5728
LOC102725072.start-FAM230F.start.18846286-18865042.NEW.ref            ctgcagagtcagaacagcctctccccagtggggatcatctctctctgccaaagcaacagc  5728
chimp.100000-160000.Pan.troglodytes.isolate.Yerkes.chimp.pedigree.#C0471   ctgcagagtcagaacagcctctccccagtggggatcatctctctctgccaaagcaacagc  9179
BCRP2.ref.rev.compl                                                   ------------------------------------------------------------  0

exon11.USP18.human                                                    ------------------------------------------------------------  0
LOC102725072.(18846286-18861064new.ref                                ggtccctgccccaaccagactaccccactcagtggagttacggatgctgctccagcatcc  5788
LOC102725072.start-FAM230F.start.18846286-18865042.NEW.ref            ggtccctgccccaaccagactaccccactcagtggagttacggatgctgctccagcatcc  5788
chimp.100000-160000.Pan.troglodytes.isolate.Yerkes.chimp.pedigree.#C0471   ggtccctgccccaaccagactaacccactcagtggagttacggatgctgctccagcatcc  9239
BCRP2.ref.rev.compl                                                   ------------------------------------------------------------  0
```

```
exon11.USP18.human                                                             ------------------------------------------------------------  0
LOC102725072.(18846286-18861064new.ref                                         taacactgcccagctggtgcctgcctgtgctcacccacacccccaggccggccttccct  5848
LOC102725072.start-FAM230F.start.18846286-18865042.NEW.ref                     taacactgcccagctggtgcctgcctgtgctcacccacacccccaggccggccttccct  5848
chimp.100000-160000.Pan.troglodytes.isolate.Yerkes.chimp.pedigree.#C0471       taacactgcccagctggtgcctgcctgtgctcacccacacccccaggctggccttccct  9299
BCRP2.ref.rev.compl                                                            ------------------------------------------------------------  0


exon11.USP18.human                                                             ------------------------------------------------------------  0
LOC102725072.(18846286-18861064new.ref                                         gcagcctgggcttggccaccttggcctgattgagcactgaggcctcctgggcacccagcc  5908
LOC102725072.start-FAM230F.start.18846286-18865042.NEW.ref                     gcagcctgggcttggccaccttggcctgattgagcactgaggcctcctgggcacccagcc  5908
chimp.100000-160000.Pan.troglodytes.isolate.Yerkes.chimp.pedigree.#C0471       gcagcctgggcttggccaccttggcctgattgagcactgaggcctcctaggcacccagcc  9359
BCRP2.ref.rev.compl                                                            ------------------------------------------------------------  0


exon11.USP18.human                                                             ------------------------------------------------------------  0
LOC102725072.(18846286-18861064new.ref                                         ccatcactgcacctgctgcttccagccccaccccaccggctcaggggttcttcccagcgg  5968
LOC102725072.start-FAM230F.start.18846286-18865042.NEW.ref                     ccatcactgcacctgctgcttccagccccaccccaccggctcaggggttcttcccagcgg  5968
chimp.100000-160000.Pan.troglodytes.isolate.Yerkes.chimp.pedigree.#C0471       ccatcactgcacctgctgcttccagccccaccccaccggctcaggggttcttcccagcgg  9419
BCRP2.ref.rev.compl                                                            ------------------------------------------------------------  0


exon11.USP18.human                                                             ------------------------------------------------------------  0
LOC102725072.(18846286-18861064new.ref                                         cgctgatcatgaagtcaacatgcacgcaagtcgtctcaggaaacttcttaatgaaagtgt  6028
LOC102725072.start-FAM230F.start.18846286-18865042.NEW.ref                     cgctgatcatgaagtcaacatgcacgcaagtcgtctcaggaaacttcttaatgaaagtgt  6028
chimp.100000-160000.Pan.troglodytes.isolate.Yerkes.chimp.pedigree.#C0471       tgctgatcatgaaatcaacatgcacacaagtcgtctcaggaaactttttaatgaaagtgt  9479
BCRP2.ref.rev.compl                                                            ------------------------------tcgtctcaggaaacttcttaatgaaagtgt  30
```

start of identity of BCRP2 in LOC102725072

```
exon11.USP18.human                                                             ------------------------------------------------------------  0
LOC102725072.(18846286-18861064new.ref                                         cggccacggtggtgtgtaggtggctgagctcagattgcagctgctaagacaccagccact  6088
LOC102725072.start-FAM230F.start.18846286-18865042.NEW.ref                     cggccacggtggtgtgtaggtggctgagctcagattgcagctgctaagacaccagccact  6088
chimp.100000-160000.Pan.troglodytes.isolate.Yerkes.chimp.pedigree.#C0471       tggccacggtggtgtgtaggtggctgagctcagattgcagctgctaagacaccagccact  9539
BCRP2.ref.rev.compl                                                            cggccacggtggtgtgtaggtggctgagctcagattgcagctgctaagacaccagccact  90


exon11.USP18.human                                                             ------------------------------------------------------------  0
LOC102725072.(18846286-18861064new.ref                                         taccaagagaaagccaggctgcttcaaacccagggcccacggcaaaaaagcatcacttcc  6148
LOC102725072.start-FAM230F.start.18846286-18865042.NEW.ref                     taccaagagaaagccaggctgcttcaaacccagggcccacggcaaaaaagcatcacttcc  6148
chimp.100000-160000.Pan.troglodytes.isolate.Yerkes.chimp.pedigree.#C0471       taccaagagaaagccaggctgcttcaaacccagggcccgaggcaaaaaagcatcacttct  9599
BCRP2.ref.rev.compl                                                            taccaagagaaagccaggctgcttcaaacccagggcccacggcaaaaaagcatcacttcc  150


exon11.USP18.human                                                             ------------------------------------------------------------  0
LOC102725072.(18846286-18861064new.ref                                         ggccggggagtctggaagccacgccttgtgggaggtcacactggcatctaggccttcgcc  6208
LOC102725072.start-FAM230F.start.18846286-18865042.NEW.ref                     ggccggggagtctggaagccacgccttgtgggaggtcacactggcatctaggccttcgcc  6208
chimp.100000-160000.Pan.troglodytes.isolate.Yerkes.chimp.pedigree.#C0471       ggtcggggagtctggaagccatgccttgtgggaggtcacactggcatctaggccttcgcc  9659
BCRP2.ref.rev.compl                                                            ggccggggagtctggaagccacgccttgtgggaggtcacactggcatctaggccttcgcc  210


exon11.USP18.human                                                             ------------------------------------------------------------  0
LOC102725072.(18846286-18861064new.ref                                         tgcattgcagaaggagagccgggtcccccctcctggagaacgctgcgttccccagccccac  6268
LOC102725072.start-FAM230F.start.18846286-18865042.NEW.ref                     tgcattgcagaaggagagccgggtcccccctcctggagaacgctgcgttccccagccccac  6268
chimp.100000-160000.Pan.troglodytes.isolate.Yerkes.chimp.pedigree.#C0471       tgcattgcagaaggagagccgggtcccccctcctggagaacgctgcgttccccagccccac  9719
BCRP2.ref.rev.compl                                                            tgcattgcagaaggagagccgggtcccccctcctggagaacgctgcgttccccagccccac  270


exon11.USP18.human                                                             ------------------------------------------------------------  0
LOC102725072.(18846286-18861064new.ref                                         accggctttgccaccacacaggctgttgaggcaggaggcgggtaagacgtagctgtagac  6328
LOC102725072.start-FAM230F.start.18846286-18865042.NEW.ref                     accggctttgccaccacacaggctgttgaggcaggaggcgggtaagacgtagctgtagac  6328
chimp.100000-160000.Pan.troglodytes.isolate.Yerkes.chimp.pedigree.#C0471       accggctttgccaccacacaggcttttgaggcaggaggtgggtaagacgtagctgtagac  9779
BCRP2.ref.rev.compl                                                            accggctttgccaccacacaggctgttgaggcaggaggcgggtaagacgtagctgtagac  330


exon11.USP18.human                                                             ------------------------------------------------------------  0
LOC102725072.(18846286-18861064new.ref                                         ccaaagcaaccaccagccctgggaccctgcgggagaggagcacttttagaacatggaaaa  6388
LOC102725072.start-FAM230F.start.18846286-18865042.NEW.ref                     ccaaagcaaccaccagccctgggaccctgcgggagaggagcacttttagaacatggaaaa  6388
chimp.100000-160000.Pan.troglodytes.isolate.Yerkes.chimp.pedigree.#C0471       ccaaagcaaccaccagccctgggaccctgcgggagaggagcacttttagaacatggaaaa  9839
BCRP2.ref.rev.compl                                                            ccaaagcaaccaccagccctgggaccctgcgggagaggagcacttttagaacatggaaaa  390


exon11.USP18.human                                                             ------------------------------------------------------------  0
LOC102725072.(18846286-18861064new.ref                                         gtgtggtcatcccatcattagacagcacacatcctacataaataaaaagtcgtatgggga  6448
LOC102725072.start-FAM230F.start.18846286-18865042.NEW.ref                     gtgtggtcatcccatcattagacagcacacatcctacataaataaaaagtcgtatgggga  6448
chimp.100000-160000.Pan.troglodytes.isolate.Yerkes.chimp.pedigree.#C0471       gtgtggtcatcccatcattagacagcacacatcctacataaataaaaagtcatatgggga  9899
BCRP2.ref.rev.compl                                                            gtgtggtcatcccatcattagacagcacacatcctacataaataaaaagtcgtatgggga  450


exon11.USP18.human                                                             ------------------------------------------------------------  0
LOC102725072.(18846286-18861064new.ref                                         aggaggttggggagggaataaaaaattggcacagacattgatagactggtttccagtttc  6508
LOC102725072.start-FAM230F.start.18846286-18865042.NEW.ref                     aggaggttggggagggaataaaaaattggcacagacattgatagactggtttccagtttc  6508
chimp.100000-160000.Pan.troglodytes.isolate.Yerkes.chimp.pedigree.#C0471       aggaggtcggggagggaataaaaaattggcacagacattgatagactggtttccagtttc  9959
BCRP2.ref.rev.compl                                                            aggaggttggggagggaataaaaaattggcacagacattgatagactggtttccagtttc  510


exon11.USP18.human                                                             ------------------------------------------------------------  0
LOC102725072.(18846286-18861064new.ref                                         aaggtaacagatgcacatcatgagaccagaggaggcagagacaa-ggctggatttggctt  6567
LOC102725072.start-FAM230F.start.18846286-18865042.NEW.ref                     aaggtaacagatgcacatcatgagaccagaggaggcagagacaa-ggctggatttggctt  6567
chimp.100000-160000.Pan.troglodytes.isolate.Yerkes.chimp.pedigree.#C0471       atggtaacagatgcacatcatgagaccagaggaggcagagacaagggctggatttggctt  10019
BCRP2.ref.rev.compl                                                            aaggtaacagatgcacatcatgagaccagaggaggcagagacaa-ggctggatttggctt  569


exon11.USP18.human                                                             ------------------------------------------------------------  0
LOC102725072.(18846286-18861064new.ref                                         ttctaagcaacatgtgttcctgcgcagggctgaatggtcgctgagacagagatggaagcc  6627
LOC102725072.start-FAM230F.start.18846286-18865042.NEW.ref                     ttctaagcaacatgtgttcctgcgcagggctgaatggtcgctgagacagagatggaagcc  6627
chimp.100000-160000.Pan.troglodytes.isolate.Yerkes.chimp.pedigree.#C0471       ttctaagcaacatgtgttcctgtgcagggctgaatggtcgctgagacagagatggaagcc  10079
BCRP2.ref.rev.compl                                                            ttctaagcaacacgtgttcctgcgcagggctgaatggttgctgagacagagatggaagcc  629


exon11.USP18.human                                                             ------------------------------------------------------------  0
LOC102725072.(18846286-18861064new.ref                                         aggacaagggagcccaccgggcccagataggtacagagagcagaggctcctgttctgtcc  6687
LOC102725072.start-FAM230F.start.18846286-18865042.NEW.ref                     aggacaagggagcccaccgggcccagataggtacagagagcagaggctcctgttctgtcc  6687
chimp.100000-160000.Pan.troglodytes.isolate.Yerkes.chimp.pedigree.#C0471       aggacaggggagcccaccgggcccagataggtacagagagcagaggctcctgttctgtcc  10139
BCRP2.ref.rev.compl                                                            aggacaagggagcccaccgggcccagataggtacagagagcagaggctcctgttctgtcc  689


exon11.USP18.human                                                             ------------------------------------------------------------  0
LOC102725072.(18846286-18861064new.ref                                         tcgccacccacgagggtgacactgcttgtaaatggtggctgtgctctcccagcaagaaaa  6747
LOC102725072.start-FAM230F.start.18846286-18865042.NEW.ref                     tcgccacccacgagggtgacactgcttgtaaatggtggctgtgctctcccagcaagaaaa  6747
chimp.100000-160000.Pan.troglodytes.isolate.Yerkes.chimp.pedigree.#C0471       tcgccacccatgagggtgacactgcttgtaaatggtggctgtgctctcccagcaagaaaa  10199
BCRP2.ref.rev.compl                                                            tcgccacccacgagggtgacactgcttgtaaatggtggctgtgctctcccagcaagaaaa  749


exon11.USP18.human                                                             ------------------------------------------------------------  0
LOC102725072.(18846286-18861064new.ref                                         aagcacaactaaatccacactgcacacagacgcagacagaaagccttcaagtggctctgt  6807
LOC102725072.start-FAM230F.start.18846286-18865042.NEW.ref                     aagcacaactaaatccacactgcacacagacgcagacagaaagccttcaagtggctctgt  6807
chimp.100000-160000.Pan.troglodytes.isolate.Yerkes.chimp.pedigree.#C0471       aagcacaactaaatccacactgcacaaagacgcagacagaaagccttcaagtgg--ctgt  10257
BCRP2.ref.rev.compl                                                            aagcacaactaaatccacactgcacacagacgcagacagaaagccttcaagtggctctgt  809


exon11.USP18.human                                                             ------------------------------------------------------------  0
LOC102725072.(18846286-18861064new.ref                                         tttctgctccctgccttgccaggtccacaagcagagaggagtgtcaggcacatggccccg  6867
LOC102725072.start-FAM230F.start.18846286-18865042.NEW.ref                     tttctgctccctgccttgccaggtccacaagcagagaggagtgtcaggcacatggccccg  6867
chimp.100000-160000.Pan.troglodytes.isolate.Yerkes.chimp.pedigree.#C0471       tttctgctccctgcctcatcaggtccacaagcagagaggagtgtcaggcacatggccccg  10317
BCRP2.ref.rev.compl                                                            tttctgctccctgccttgccaggtccacaagcagagaggagtgtcaggcacatggccccg  869


exon11.USP18.human                                                             ------------------------------------------------------------  0
LOC102725072.(18846286-18861064new.ref                                         ctgtcaggctccccagtgagctgcgggctcagcaggagctgcccactgacacacaggggga  6927
LOC102725072.start-FAM230F.start.18846286-18865042.NEW.ref                     ctgtcaggctccccagtgagctgcgggctcagcaggagctgcccactgacacacaggggga  6927
chimp.100000-160000.Pan.troglodytes.isolate.Yerkes.chimp.pedigree.#C0471       ctgtcaggctcaccagtgagctgcgggctcagcaggagctgcccactgacacacaggggga  10377
BCRP2.ref.rev.compl                                                            ctgtcaggctccccagtgagctgcgggctcagcaggagctgcccactgacacacaggggga  929


exon11.USP18.human                                                             ------------------------------------------------------------  0
LOC102725072.(18846286-18861064new.ref                                         cacccactcctgccaccttgggagcggttgccagacagagccgcactgggtgctggtgtc  6987
LOC102725072.start-FAM230F.start.18846286-18865042.NEW.ref                     cacccactcctgccaccttgggagcggttgccagacagagccgcactgggtgctggtgtc  6987
chimp.100000-160000.Pan.troglodytes.isolate.Yerkes.chimp.pedigree.#C0471       cacccactcctgccgccttgggagcagttgccagacagagccgcactgggtgctggtgtc  10437
BCRP2.ref.rev.compl                                                            cacccactcctgccaccttgggagcggttgccagacagagccgtactgggtgctggtgtc  989


exon11.USP18.human                                                             ------------------------------------------------------------  0
LOC102725072.(18846286-18861064new.ref                                         atccagggaccccacacacttccttaaatgtgatcctgcttccctctgcgcagctgcatc  7047
LOC102725072.start-FAM230F.start.18846286-18865042.NEW.ref                     atccagggaccccacacacttccttaaatgtgatcctgcttccctctgcgcagctgcatc  7047
```

```
chimp.100000-160000.Pan.troglodytes.isolate.Yerkes.chimp.pedigree.#C0471      atccagggaccccacacacttccttaaatgtgatcctgcttccctctgggcagctgcatc  10497
BCRP2.ref.rev.compl                                                           atccagggaccccacacacttccttaaatgtgatcctgcttccctctgcgcagctgcatc  1049


exon11.USP18.human                                                            ------------------------------------------------------------  0
LOC102725072.(18846286-18861064new.ref                                        ctctcctcctgcaggaccgtctggaaacttggctctcagtttgctctcccttctctcctc  7107
LOC102725072.start-FAM230F.start.18846286-18865042.NEW.ref                    ctctcctcctgcaggaccgtctggaaacttggctctcagtttgctctcccttctctcctc  7107
chimp.100000-160000.Pan.troglodytes.isolate.Yerkes.chimp.pedigree.#C0471      ctctcctcttgcaggactgtctggaaacttggctctcagtttgctctcccttctctcctc  10557
BCRP2.ref.rev.compl                                                           ctctcctcctgcaggaccgtctggaaacttggctctcagtttgctctcccttctctcctc  1109


exon11.USP18.human                                                            ------------------------------------------------------------  0
LOC102725072.(18846286-18861064new.ref                                        tgcctgccccaagcccctctttctaaaaaagtgatgccatgttcatggggttatttcttg  7167
LOC102725072.start-FAM230F.start.18846286-18865042.NEW.ref                    tgcctgccccaagcccctctttctaaaaaagtgatgccatgttcatggggttatttcttg  7167
chimp.100000-160000.Pan.troglodytes.isolate.Yerkes.chimp.pedigree.#C0471      tgcctgccccaagcccctctttctaaaaaagtgatgccatgttcatggggttatttcttg  10617
BCRP2.ref.rev.compl                                                           tgcctgccccaagcccctctttctaaaaaagtgatgccacgttcatgggattatttcttg  1169


exon11.USP18.human                                                            ------------------------------------------------------------  0
LOC102725072.(18846286-18861064new.ref                                        aaaatacttggcggcctccatgcttctgttttctttgagccaggtggtcaggagggctta  7227
LOC102725072.start-FAM230F.start.18846286-18865042.NEW.ref                    aaaatacttggcggcctccatgcttctgttttctttgagccaggtggtcaggagggctta  7227
chimp.100000-160000.Pan.troglodytes.isolate.Yerkes.chimp.pedigree.#C0471      aaaatacttggcggcctccatgcttctgttttctttgagccaggtggtcaggagggctta  10677
BCRP2.ref.rev.compl                                                           aaaatacttggcggcctccatgcttctgttttctttgagtcaggtagtcaggagggttta  1229


exon11.USP18.human                                                            ------------------------------------------------------------  0
LOC102725072.(18846286-18861064new.ref                                        caaacaatgcctgggctcccccgcagctgtcggcagatggggtagcgaatggtcctgtgc  7287
LOC102725072.start-FAM230F.start.18846286-18865042.NEW.ref                    caaacaatgcctgggctcccccgcagctgtcggcagatggggtagcgaatggtcctgtgc  7287
chimp.100000-160000.Pan.troglodytes.isolate.Yerkes.chimp.pedigree.#C0471      caaagaatgcctgggctcccccacaggtgtcggcagatggggtagcgaatggtcctgtgc  10737
BCRP2.ref.rev.compl                                                           caaacaatgcctgggctcccccgcaggtgccggcagatggggtagcgaatggtcctgtgc  1289


exon11.USP18.human                                                            ------------------------------------------------------------  0
LOC102725072.(18846286-18861064new.ref                                        ctccacctgctccgggagggagtctcccgtctctaggcctagccccttcctaaccctcca  7347
LOC102725072.start-FAM230F.start.18846286-18865042.NEW.ref                    ctccacctgctccgggagggagtctcccgtctctaggcctagccccttcctaaccctcca  7347
chimp.100000-160000.Pan.troglodytes.isolate.Yerkes.chimp.pedigree.#C0471      ctccacctgctccgggagggagtctcccgtctctaggcctggccccttcctaaccctcca  10797
BCRP2.ref.rev.compl                                                           ctccacctgctccgggagggagtctcccgtctctaggcctggccccttcctaaccctcca  1349


exon11.USP18.human                                                            ------------------------------------------------------------  0
LOC102725072.(18846286-18861064new.ref                                        cgtatcctgttctccagagacttcagaacccactcctgagaacagcggagccaggcgctt  7407
LOC102725072.start-FAM230F.start.18846286-18865042.NEW.ref                    cgtatcctgttctccagagacttcagaacccactcctgagaacagcggagccaggcgctt  7407
chimp.100000-160000.Pan.troglodytes.isolate.Yerkes.chimp.pedigree.#C0471      cgtattctgttctccagagacttcagaacccactcctgagaacagcggagccaggcgctt  10857
BCRP2.ref.rev.compl                                                           cgtatcctgttctccagagacttcagaacccactcctgagaacagcggagccaggcgctt  1409


exon11.USP18.human                                                            ------------------------------------------------------------  0
LOC102725072.(18846286-18861064new.ref                                        agaggaagaccaaatgctgccaggacacggattgtcca-ggattacattccagcatctta  7466
LOC102725072.start-FAM230F.start.18846286-18865042.NEW.ref                    agaggaagaccaaatgctgccaggacacggattgtcca-ggattacattccagcatctta  7466
chimp.100000-160000.Pan.troglodytes.isolate.Yerkes.chimp.pedigree.#C0471      agaggaagaccaaatgctgccaggacacggagtgtccagggattacattccagcatctta  10917
BCRP2.ref.rev.compl                                                           agaggaagaccaaatgctgccaggacacggattgtccagggattacattccagcatctta  1469


exon11.USP18.human                                                            ------------------------------------------------------------  0
LOC102725072.(18846286-18861064new.ref                                        ttaggtatctggatctgttggggaaaaaattagaaactatgtataaaacttaaaaatatt  7526
LOC102725072.start-FAM230F.start.18846286-18865042.NEW.ref                    ttaggtatctggatctgttggggaaaaaattagaaactatgtataaaacttaaaaatatt  7526
chimp.100000-160000.Pan.troglodytes.isolate.Yerkes.chimp.pedigree.#C0471      ttaggtatctggatctgttggggaaaaaattagaaactatgtataaaacttaaaaatatt  10977
BCRP2.ref.rev.compl                                                           ttaggtatctggatctgttggggaaaaaattagaaactatgtataaaacttacaaatatt  1529


exon11.USP18.human                                                            ------------------------------------------------------------  0
LOC102725072.(18846286-18861064new.ref                                        caagcatcaaaaggttatttaggatgaaagttttaaaacaagtcatcagcaagctgctac  7586
LOC102725072.start-FAM230F.start.18846286-18865042.NEW.ref                    caagcatcaaaaggttatttaggatgaaagttttaaaacaagtcatcagcaagctgctac  7586
chimp.100000-160000.Pan.troglodytes.isolate.Yerkes.chimp.pedigree.#C0471      caagtatcaaaaggttatttaggatgaaagttttaaaacaagtcatcagcaagctgctac  11037
BCRP2.ref.rev.compl                                                           caagtatcaaaaggttatttaggatgaaagttttaaaacaagtcatcagcaagctgctac  1589


exon11.USP18.human                                                            ------------------------------------------------------------  0
LOC102725072.(18846286-18861064new.ref                                        caccaagtggagacttatacaaaagttgagcgagtccactgagctgagaggacagaaatg  7646
LOC102725072.start-FAM230F.start.18846286-18865042.NEW.ref                    caccaagtggagacttatacaaaagttgagcgagtccactgagctgagaggacagaaatg  7646
chimp.100000-160000.Pan.troglodytes.isolate.Yerkes.chimp.pedigree.#C0471      caccaagtggagacttatacaaaagttgagcgagtccactgagctgagaggacagaaatg  11097
BCRP2.ref.rev.compl                                                           caccaagtggagacttatacaaaagttgagcgagtccactgagctgagaggacagaaatg  1649


exon11.USP18.human                                                            ------------------------------------------------------------  0
LOC102725072.(18846286-18861064new.ref                                        aagtcacctgtgctggggcaggggcagggacactgggggcagggagtgtgtgggcagaga  7706
LOC102725072.start-FAM230F.start.18846286-18865042.NEW.ref                    aagtcacctgtgctggggcaggggcagggacactgggggcagggagtgtgtgggcagaga  7706
chimp.100000-160000.Pan.troglodytes.isolate.Yerkes.chimp.pedigree.#C0471      acgtcacctgtgctggggcaggggcagggacactgggggcagggagtgtgtgggcagaga  11157
BCRP2.ref.rev.compl                                                           aagtcacctgtgctggggcaggggcagggacactgggggcagggagtgtgtgggcagaga  1709


exon11.USP18.human                                                            ------------------------------------------------------------  0
LOC102725072.(18846286-18861064new.ref                                        agccagagaagtccaggcctgtggaagccaaacaggagagcgtgggccggaagggcggtc  7766
LOC102725072.start-FAM230F.start.18846286-18865042.NEW.ref                    agccagagaagtccaggcctgtggaagccaaacaggagagcgtgggccggaagggcggtc  7766
chimp.100000-160000.Pan.troglodytes.isolate.Yerkes.chimp.pedigree.#C0471      agccagagaagtccaggcctgtggaagccaaacaggagagcgtgggccggaagggcggtc  11217
BCRP2.ref.rev.compl                                                           agccagagaagtccaggcctgtggaagccaaacaggagagcgtgggccggaagggcggtc  1769


exon11.USP18.human                                                            ------------------------------------------------------------  0
LOC102725072.(18846286-18861064new.ref                                        aggatcgggggacgaggtcgctctccctggagaacgaaccctaaggtgcatagcctggga  7826
LOC102725072.start-FAM230F.start.18846286-18865042.NEW.ref                    aggatcgggggacgaggtcgctctccctggagaacgaaccctaaggtgcatagcctggga  7826
chimp.100000-160000.Pan.troglodytes.isolate.Yerkes.chimp.pedigree.#C0471      aggatcgggggacgaggtcgctctccctggagaacgaaccctaaagtgcgtagcctggga  11277
BCRP2.ref.rev.compl                                                           aggatcgggggacgaggtcgctctccctggagaacgaaccctaaagtgcgtagcctggga  1829


exon11.USP18.human                                                            ------------------------------------------------------------  0
LOC102725072.(18846286-18861064new.ref                                        ttccctccctggaggtcctgtcccccgacatttcacgggccttctgagctgccttccaag  7886
LOC102725072.start-FAM230F.start.18846286-18865042.NEW.ref                    ttccctccctggaggtcctgtcccccgacatttcacgggccttctgagctgccttccaag  7886
chimp.100000-160000.Pan.troglodytes.isolate.Yerkes.chimp.pedigree.#C0471      ttccctccctgagggtcctgtcccctgacatttcacgggccttctgagctgccttccaag  11337
BCRP2.ref.rev.compl                                                           ttccctccctgggggtcctgtcccccgacgtttcacgggccttctgagctgccttccaag  1889


exon11.USP18.human                                                            ------------------------------------------------------------  0
LOC102725072.(18846286-18861064new.ref                                        gaggactaacacggcaacaaaagaaccatttctgcacaaaaatccttctgggaagaaaaa  7946
LOC102725072.start-FAM230F.start.18846286-18865042.NEW.ref                    gaggactaacacggcaacaaaagaaccatttctgcacaaaaatccttctgggaagaaaaa  7946
chimp.100000-160000.Pan.troglodytes.isolate.Yerkes.chimp.pedigree.#C0471      gaggactaacacggcaacaaaagacccatttctgcacaaaaatccttctggaaagaaaaa  11397
BCRP2.ref.rev.compl                                                           gaggactaacacggcaacaaaagacccatttctgcacaaaaatccctctgggaagaaaaa  1949


exon11.USP18.human                                                            ------------------------------------------------------------  0
LOC102725072.(18846286-18861064new.ref                                        gaagaaagccaagaatggagtcaaaacgctacccagtgctgactaagcctctcaaaccct  8006
LOC102725072.start-FAM230F.start.18846286-18865042.NEW.ref                    gaagaaagccaagaatggagtcaaaacgctacccagtgctgactaagcctctcaaaccct  8006
chimp.100000-160000.Pan.troglodytes.isolate.Yerkes.chimp.pedigree.#C0471      gaagaaagccaagaatggagtcaaaacgctacccagtgctgactaagcctctcaaaccct  11457
BCRP2.ref.rev.compl                                                           gaagaaagccaagaatggagtcaaaacgctacccagtgctgaccaagcctctcaaaccct  2009


exon11.USP18.human                                                            ------------------------------------------------------------  0
LOC102725072.(18846286-18861064new.ref                                        gttctaggtggactgtggtttctaagtcagggaaatggaagaggccccacccacacaggg  8066
LOC102725072.start-FAM230F.start.18846286-18865042.NEW.ref                    gttctaggtggactgtggtttctaagtcagggaaatggaagaggccccacccacacaggg  8066
chimp.100000-160000.Pan.troglodytes.isolate.Yerkes.chimp.pedigree.#C0471      gttctaagtggagtgtggtttctaagtcagggaaatggaagaggccccacccacacaggg  11517
BCRP2.ref.rev.compl                                                           gttctaagtggactgtggtttctaagtcagggaaatggaagaggccccacccacacaggg  2069


exon11.USP18.human                                                            ------------------------------------------------------------  0
LOC102725072.(18846286-18861064new.ref                                        acagggccatggcccccacaggatgaagcagcagcgtttattcaagatacaacagtgagg  8126
LOC102725072.start-FAM230F.start.18846286-18865042.NEW.ref                    acagggccatggcccccacaggatgaagcagcagcgtttattcaagatacaacagtgagg  8126
chimp.100000-160000.Pan.troglodytes.isolate.Yerkes.chimp.pedigree.#C0471      acagggccatggcccccacaggatgaagcagcagcgtttattcaagatacaacagtgagg  11577
BCRP2.ref.rev.compl                                                           acagggccatggcccccacaggatgaagcagcagcgtttattcaagatacaacagtgagg  2129


exon11.USP18.human                                                            ------------------------------------------------------------  0
LOC102725072.(18846286-18861064new.ref                                        gaatccggtcacgttcccttctccccagagagggcgcttcttgacaagtgattcagtaga  8186
LOC102725072.start-FAM230F.start.18846286-18865042.NEW.ref                    gaatccggtcacgttcccttctccccagagagggcgcttcttgacaagtgattcagtaga  8186
chimp.100000-160000.Pan.troglodytes.isolate.Yerkes.chimp.pedigree.#C0471      gaatccagtcacgttcccttctccccagagagggcgcttcttgacaagtgattcagtaga  11637
BCRP2.ref.rev.compl                                                           gaatccagtcacgttcccttctccccagagagggcgcttcttgacaagtgattcagtaga  2189


exon11.USP18.human                                                            ------------------------------------------------------------  0
LOC102725072.(18846286-18861064new.ref                                        aatcttttagactctataagttaagttcataaaaaccactgctttcaccctgtctcccag  8246
LOC102725072.start-FAM230F.start.18846286-18865042.NEW.ref                    aatcttttagactctataagttaagttcataaaaaccactgctttcaccctgtctcccag  8246
chimp.100000-160000.Pan.troglodytes.isolate.Yerkes.chimp.pedigree.#C0471      aatcttttagactctataagttaagttcataaaaaccactgctttcaccctgtctcccag  11697
BCRP2.ref.rev.compl                                                           aatcttttggactctataagttaagttcataaaaaccactgctttcaccctgtctcccag  2249


exon11.USP18.human                                                            ------------------------------------------------------------  0
```

```
LOC102725072.(18846286-18861064new.ref                                      ggccaggcctggactccgagatgaactggtttggggcgccctcgggtggccacataaaaa  8306
LOC102725072.start-FAM230F.start.18846286-18865042.NEW.ref                  ggccaggcctggactccgagatgaactggtttggggcgccctcgggtggccacataaaaa  8306
chimp.100000-160000.Pan.troglodytes.isolate.Yerkes.chimp.pedigree.#C0471    ggccaggcctggactccgagatgaactggtttggggcgccctcgggtggccacataaaaa  11757
BCRP2.ref.rev.compl                                                         ggccaggcctggactccgagatgaactggtttggggcgccctcgggtggccacataaaaa  2309


exon11.USP18.human                                                          ------------------------------------------------------------  0
LOC102725072.(18846286-18861064new.ref                                      acccacagtctgaggccagcctggggctttcagacctgggcgggatctgcccaggccacc  8366
LOC102725072.start-FAM230F.start.18846286-18865042.NEW.ref                  acccacagtctgaggccagcctggggctttcagacctgggcgggatctgcccaggccacc  8366
chimp.100000-160000.Pan.troglodytes.isolate.Yerkes.chimp.pedigree.#C0471    acccacagtctgaggccagcctggggctttcagacctgggcgggatctgcccaggccacc  11817
BCRP2.ref.rev.compl                                                         acccacagtctgaggccagcctggggctttcagacctgggcgggatctgcccaggccacc  2369


exon11.USP18.human                                                          ------------------------------------------------------------  0
LOC102725072.(18846286-18861064new.ref                                      tgtccttctgctttgggccgctgtctcttggcagatggcctgacacctgggggtggccca  8426
LOC102725072.start-FAM230F.start.18846286-18865042.NEW.ref                  tgtccttctgctttgggccgctgtctcttggcagatggcctgacacctgggggtggccca  8426
chimp.100000-160000.Pan.troglodytes.isolate.Yerkes.chimp.pedigree.#C0471    tgtccttctgctttgggccgctgtctcttggcagatggcctgacacctgggggtggtcca  11877
BCRP2.ref.rev.compl                                                         tgtccttctgctttgggccgctgtctcttggcagatggcctgacacctgggggtggccca  2429


exon11.USP18.human                                                          ------------------------------------------------------------  0
LOC102725072.(18846286-18861064new.ref                                      aggatgcctcagaaaatcttgattcccactctacagatggcctgattagccagaggtttc  8486
LOC102725072.start-FAM230F.start.18846286-18865042.NEW.ref                  aggatgcctcagaaaatcttgattcccactctacagatggcctgattagccagaggtttc  8486
chimp.100000-160000.Pan.troglodytes.isolate.Yerkes.chimp.pedigree.#C0471    aggatgcctcagaaaatcttgattcccactctacagatggccggattagccagaggtttc  11937
BCRP2.ref.rev.compl                                                         aggatgcctcagaaaatcttgattcccactctacagatggcctgattagccagaggtttc  2489


exon11.USP18.human                                                          ------------------------------------------------------------  0
LOC102725072.(18846286-18861064new.ref                                      caggccgtctgtccgcctccaggagatggactgggacctttagacatcggtggagaacag  8546
LOC102725072.start-FAM230F.start.18846286-18865042.NEW.ref                  caggccgtctgtccgcctccaggagatggactgggacctttagacatcggtggagaacag  8546
chimp.100000-160000.Pan.troglodytes.isolate.Yerkes.chimp.pedigree.#C0471    caggccgtctgtccgcctccaggagatggactgggacctttagacatcggtggacaacag  11997
BCRP2.ref.rev.compl                                                         caggccgtctgtccgcctccaggagatggactgggacctttagacatcggtggagaacag  2549


exon11.USP18.human                                                          ------------------------------------------------------------  0
LOC102725072.(18846286-18861064new.ref                                      gatgctctgtcccttgctgtccagggcagggatggcctccagccgcaagaagtacagcag  8606
LOC102725072.start-FAM230F.start.18846286-18865042.NEW.ref                  gatgctctgtcccttgctgtccagggcagggatggcctccagccgcaagaagtacagcag  8606
chimp.100000-160000.Pan.troglodytes.isolate.Yerkes.chimp.pedigree.#C0471    gatgctctgtcccttgctgtccagggcagggatggcctccagccgcaagaagtacagcag  12057
BCRP2.ref.rev.compl                                                         gatgctctgtcccttgctgtccagggcagggatggcctccagccgcaagaagtacagcag  2609


exon11.USP18.human                                                          ------------------------------------------------------------  0
LOC102725072.(18846286-18861064new.ref                                      cacctcgacctgccctcgcggagtggggaagaggagagtggctcagagcggggctcacag  8666
LOC102725072.start-FAM230F.start.18846286-18865042.NEW.ref                  cacctcgacctgccctcgcggagtggggaagaggagagtggctcagagcggggctcacag  8666
chimp.100000-160000.Pan.troglodytes.isolate.Yerkes.chimp.pedigree.#C0471    cacctcgacctgccctcgcagagtggggaagaggagagtggctcagagggggctcacag  12117
BCRP2.ref.rev.compl                                                         cacctcgacctgccctcgcggagtggggaagaggagagtggctcggaaggggcgcacag  2669


exon11.USP18.human                                                          ------------------------------------------------------------  0
LOC102725072.(18846286-18861064new.ref                                      ctgctggtggggaggtctttggggcccaagctcccaagtccacctcaggtgctagaaacc  8726
LOC102725072.start-FAM230F.start.18846286-18865042.NEW.ref                  ctgctggtggggaggtctttggggcccaagctcccaagtccacctcaggtgctagaaacc  8726
chimp.100000-160000.Pan.troglodytes.isolate.Yerkes.chimp.pedigree.#C0471    ctgctggtggggaggtctttggggcccaagcccccaagtccacctcaggtgctagaaacc  12177
BCRP2.ref.rev.compl                                                         ctgctggt-gggaggtctttggggcccaagatcccaagtccacctcaggtgctagaaacc  2728


exon11.USP18.human                                                          ------------------------------------------------------------  0
LOC102725072.(18846286-18861064new.ref                                      cctgctggtgtcatgaaccccttacagtgggacgggggtggggtggggtcctgacaaggc  8786
LOC102725072.start-FAM230F.start.18846286-18865042.NEW.ref                  cctgctggtgtcatgaaccccttacagtgggacgggggtggggtggggtcctgacaaggc  8786
chimp.100000-160000.Pan.troglodytes.isolate.Yerkes.chimp.pedigree.#C0471    cctgctggtgtcatgaaccccttacagtgagacgggggtggggtggggtcctgacaaggc  12237
BCRP2.ref.rev.compl                                                         cctgctggtgtcatgaaccccttacagtgagacgggggtggggtggggtcctgacaaggc  2788


exon11.USP18.human                                                          ------------------------------------------------------------  0
LOC102725072.(18846286-18861064new.ref                                      atgacttgttgggtgaggggtggttatttattttagagatgcacagggccttgctctgtc  8846
LOC102725072.start-FAM230F.start.18846286-18865042.NEW.ref                  atgacttgttgggtgaggggtggttatttattttagagatgcacagggccttgctctgtc  8846
chimp.100000-160000.Pan.troglodytes.isolate.Yerkes.chimp.pedigree.#C0471    atgacttgttgggtgggggggtggttatttattttagagatgcacagggccttgctctgtc  12297
BCRP2.ref.rev.compl                                                         atgacttgttgggtgggggggtggttatttattttagagatgcacagggccttgctctgtc  2848


exon11.USP18.human                                                          ------------------------------------------------------------  0
LOC102725072.(18846286-18861064new.ref                                      ccccaggctggagtacagtggctccatcatggatcactgcagcctctaactcctgggctc  8906
LOC102725072.start-FAM230F.start.18846286-18865042.NEW.ref                  ccccaggctggagtacagtggctccatcatggatcactgcagcctctaactcctgggctc  8906
chimp.100000-160000.Pan.troglodytes.isolate.Yerkes.chimp.pedigree.#C0471    ccccaggctggagtacagtggctccatcatggatcactgcagcctctaactcctgggctc  12357
BCRP2.ref.rev.compl                                                         ccccaggctggagtacagtggctccatcatggatcactgcagcctctaactcctgggctc  2908


exon11.USP18.human                                                          ------------------------------------------------------------  0
LOC102725072.(18846286-18861064new.ref                                      aagcaatcctcctgtgtcagcctcccagatacctaggattacagatgtgtgccccaatgc  8966
LOC102725072.start-FAM230F.start.18846286-18865042.NEW.ref                  aagcaatcctcctgtgtcagcctcccagatacctaggattacagatgtgtgccccaatgc  8966
chimp.100000-160000.Pan.troglodytes.isolate.Yerkes.chimp.pedigree.#C0471    aagcaatcctcctgtgtcagcctcccaggtacctaggattacagatgtgtgccacaatgc  12417
BCRP2.ref.rev.compl                                                         aagcaatcctcctgtgtcagcctcccagatacctaggattacagatatgtgccccaatgc  2968


exon11.USP18.human                                                          ------------------------------------------------------------  0
LOC102725072.(18846286-18861064new.ref                                      ctgcctaatttttctttgtatttttctggagatggggtttgctacattgcccagactgg  9026
LOC102725072.start-FAM230F.start.18846286-18865042.NEW.ref                  ctgcctaatttttctttgtatttttctggagatggggtttgctacattgcccagactgg  9026
chimp.100000-160000.Pan.troglodytes.isolate.Yerkes.chimp.pedigree.#C0471    ctgcctaatttttctttgtatttttctggagatggggtttgctacattgcccagactgg  12477
BCRP2.ref.rev.compl                                                         ctgcctaatttttctttgtatttttctggagatggggtttgctacattgcccagactgg  3028


exon11.USP18.human                                                          ------------------------------------------------------------  0
LOC102725072.(18846286-18861064new.ref                                      tctcaaacacctgggttcagttgtcctgcctcggcctcccaaagtgctgggattacaggc  9086
LOC102725072.start-FAM230F.start.18846286-18865042.NEW.ref                  tctcaaacacctgggttcagttgtcctgcctcggcctcccaaagtgctgggattacaggc  9086
chimp.100000-160000.Pan.troglodytes.isolate.Yerkes.chimp.pedigree.#C0471    tctcaaacacctgggttcagttgtcctgcctcggcctcccaaagtgctgggattacaggc  12537
BCRP2.ref.rev.compl                                                         tctcaaacacctgggttcagttgtcctgcctcggcctcccaaagtgctgggattacaggc  3088


exon11.USP18.human                                                          ------------------------------------------------------------  0
LOC102725072.(18846286-18861064new.ref                                      atgagccaccacactcgaacacttggggtggttttaagcccccagcaaggtgcaccagca  9146
LOC102725072.start-FAM230F.start.18846286-18865042.NEW.ref                  atgagccaccacactcgaacacttggggtggttttaagcccccagcaaggtgcaccagca  9146
chimp.100000-160000.Pan.troglodytes.isolate.Yerkes.chimp.pedigree.#C0471    gtgagccaccacacccgaacacttggggtggttttaagcccccagcaaggtgcaccagca  12597
BCRP2.ref.rev.compl                                                         atgagccaccacactcgaacacttggggtggttttaagcccccagcaaggtgcaccagca  3148


exon11.USP18.human                                                          ------------------------------------------------------------  0
LOC102725072.(18846286-18861064new.ref                                      ggaccaggaggtggcctgggcacccccctatcactcccatccatgcaaacctaggcaagtc  9206
LOC102725072.start-FAM230F.start.18846286-18865042.NEW.ref                  ggaccaggaggtggcctgggcacccccctatcactcccatccatgcaaacctaggcaagtc  9206
chimp.100000-160000.Pan.troglodytes.isolate.Yerkes.chimp.pedigree.#C0471    ggaccaggaggtggcctaggctcccccctatcactcccatccatgcaaacctaggcaagtc  12657
BCRP2.ref.rev.compl                                                         ggaccaggaggtggcctaggcacccccctatcactcccatccatgcaaacctaggcaagtc  3208


exon11.USP18.human                                                          ------------------------------------------------------------  0
LOC102725072.(18846286-18861064new.ref                                      cctgtctctgaatctcagccaccaccacatacaatgcaagtgggaagatgggcaggactg  9266
LOC102725072.start-FAM230F.start.18846286-18865042.NEW.ref                  cctgtctctgaatctcagccaccaccacatacaatgcaagtgggaagatgggcaggactg  9266
chimp.100000-160000.Pan.troglodytes.isolate.Yerkes.chimp.pedigree.#C0471    cctgtctctgaatctcagccaccaccacatacaatgcaagtcggaagatgggcaggactg  12717
BCRP2.ref.rev.compl                                                         cctgtctctgaatctcagccaccaccacatacaatgcaagtcggaagatgggcaggactg  3268


exon11.USP18.human                                                          ------------------------------------------------------------  0
LOC102725072.(18846286-18861064new.ref                                      ggggtggggcaggcagaggccacctctgtcaggctggggttgcatgggctggaggctgtc  9326
LOC102725072.start-FAM230F.start.18846286-18865042.NEW.ref                  ggggtggggcaggcagaggccacctctgtcaggctggggttgcatgggctggaggctgtc  9326
chimp.100000-160000.Pan.troglodytes.isolate.Yerkes.chimp.pedigree.#C0471    ggggtggggcaggcagaggccacctctgtcaggctggggttgcatgggctggaggctgtc  12777
BCRP2.ref.rev.compl                                                         ggggtggggcaggcagaggccacctctgtcaggctggggttgcatgggctggaggctgtc  3328


exon11.USP18.human                                                          ------------------------------------------------------------  0
LOC102725072.(18846286-18861064new.ref                                      ttcccatacctgggacatgacctccaaggaccagctgtcagtcatggtgatgggctggct  9386
LOC102725072.start-FAM230F.start.18846286-18865042.NEW.ref                  ttcccatacctgggacatgacctccaaggaccagctgtcagtcatggtgatgggctggct  9386
chimp.100000-160000.Pan.troglodytes.isolate.Yerkes.chimp.pedigree.#C0471    ttcccatacttggggcatgacctccaaggaccagctgtcagtcatggcgatgggctggct  12837
BCRP2.ref.rev.compl                                                         ttcccatacctgggacatgacctccaaggaccagctgtcagtcatggtgatgggctggct  3388


exon11.USP18.human                                                          ------------------------------------------------------------  0
LOC102725072.(18846286-18861064new.ref                                      ggggttggcagggagcttgctctccttctcggagggccggagcagcgtggggccaaacac  9446
LOC102725072.start-FAM230F.start.18846286-18865042.NEW.ref                  ggggttggcagggagcttgctctccttctcggagggccggagcagcgtggggccaaacac  9446
chimp.100000-160000.Pan.troglodytes.isolate.Yerkes.chimp.pedigree.#C0471    ggggttggcagggagcttgctctccttctcggagggccagagcagcatggggccaaagac  12897
BCRP2.ref.rev.compl                                                         ggggttggcagggagcttgctctccttctcggagggccggagcagcgtggggccaaacac  3448


exon11.USP18.human                                                          ------------------------------------------------------------  0
LOC102725072.(18846286-18861064new.ref                                      cgtgccaaggttgtgcagggacatcttattgactgcctccttctctgccatcctgtagag  9506
LOC102725072.start-FAM230F.start.18846286-18865042.NEW.ref                  cgtgccaaggttgtgcagggacatcttattgactgcctccttctctgccatcctgtagag  9506
chimp.100000-160000.Pan.troglodytes.isolate.Yerkes.chimp.pedigree.#C0471    cgtgccgaggttgtgcagggacatcttattgaccgcctccttctctgccaccctgtagag  12957
BCRP2.ref.rev.compl                                                         cgtgccaaggttgtgcagggacatcttattgactgcctccttctctgccatcctgtagag  3508
```

```
exon11.USP18.human                                                              ------------------------------------------------------------  0
LOC102725072.(18846286-18861064new.ref                                          gaccgaagcagagggtgctgtttcaacgccaccaccaggagagaggcagaggggctgtgc  9566
LOC102725072.start-FAM230F.start.18846286-18865042.NEW.ref                      gaccgaagcagagggtgctgtttcaacgccaccaccaggagagaggcagaggggctgtgc  9566
chimp.100000-160000.Pan.troglodytes.isolate.Yerkes.chimp.pedigree.#C0471        gaccgaagcagagggtgctgtttcaacaccaccaccaggagagaggcagaggggctgtgc  13017
BCRP2.ref.rev.compl                                                             gaccgaagcagagggtgctgtttcaacgccaccaccaggagagaggcagaggggctgtgc  3568

exon11.USP18.human                                                              ------------------------------------------------------------  0
LOC102725072.(18846286-18861064new.ref                                          cgtgctagagtcctcagggagggagtgacctcgaccctggctgtgctgcaagctgactcc  9626
LOC102725072.start-FAM230F.start.18846286-18865042.NEW.ref                      cgtgctagagtcctcagggagggagtgacctcgaccctggctgtgctgcaagctgactcc  9626
chimp.100000-160000.Pan.troglodytes.isolate.Yerkes.chimp.pedigree.#C0471        cgtgctagagtcctcagggagggagtgacctcgaccctggctgtgctgcaagctgactcc  13077
BCRP2.ref.rev.compl                                                             cgtgctagagtcctcagggagggagtgacctcgaccctggctgtgctgcaagctgactcc  3628

exon11.USP18.human                                                              ------------------------------------------------------------  0
LOC102725072.(18846286-18861064new.ref                                          agccttggtacttctgggtctcagtggcccaggacaaggggccagctctgggctgatggg  9686
LOC102725072.start-FAM230F.start.18846286-18865042.NEW.ref                      agccttggtacttctgggtctcagtggcccaggacaaggggccagctctgggctgatggg  9686
chimp.100000-160000.Pan.troglodytes.isolate.Yerkes.chimp.pedigree.#C0471        agccttggtacttctgggtctcagtggcccaggacaaggggccagctctgggctgatggg  13137
BCRP2.ref.rev.compl                                                             agccttggtacttctgggtctcagtggcccaggacaaggggccagctctgggctgatggg  3688

exon11.USP18.human                                                              ------------------------------------------------------------  0
LOC102725072.(18846286-18861064new.ref                                          gaggtcttcatgatgtgcttgggagggaagggggggcggtccaaatgcactgctggccac  9746
LOC102725072.start-FAM230F.start.18846286-18865042.NEW.ref                      gaggtcttcatgatgtgcttgggagggaagggggggcggtccaaatgcactgctggccac  9746
chimp.100000-160000.Pan.troglodytes.isolate.Yerkes.chimp.pedigree.#C0471        gaggtcttcatgatgtgcttgggagggaagagggg--ggtccaagtgcactgctggccac  13195
BCRP2.ref.rev.compl                                                             gaggtcttcctgatgtgcttgggagggaagggggggcggtccaaatgcactgctggccac  3748

exon11.USP18.human                                                              ------------------------------------------------------------  0
LOC102725072.(18846286-18861064new.ref                                          ggccaaagctctgagctctttgttaaggccacagtgcagagggaggagggtggcaaagag  9806
LOC102725072.start-FAM230F.start.18846286-18865042.NEW.ref                      ggccaaagctctgagctctttgttaaggccacagtgcagagggaggagggtggcaaagag  9806
chimp.100000-160000.Pan.troglodytes.isolate.Yerkes.chimp.pedigree.#C0471        ggccaaagctctgagctctttgttaaggccacagtgcagagggaggagggtggcaaagag  13255
BCRP2.ref.rev.compl                                                             ggccaaagctctgagctctttgttaaggccacagtgcagagggaggagggtggcaaagag  3808

exon11.USP18.human                                                              ------------------------------------------------------------  0
LOC102725072.(18846286-18861064new.ref                                          gagaggcaggggcgggggtggcagtggtgctagtccttagaagcagtgagttactgcaga  9866
LOC102725072.start-FAM230F.start.18846286-18865042.NEW.ref                      gagaggcaggggcgggggtggcagtggtgctagtccttagaagcagtgagttactgcaga  9866
chimp.100000-160000.Pan.troglodytes.isolate.Yerkes.chimp.pedigree.#C0471        gagaggcaggggcgggggtggcagtggtgctagtccttagaagcagtgagttactgtaga  13315
BCRP2.ref.rev.compl                                                             gagaggcaggggcgggggtggcagtggtgctagtccttagaagcagtgagttactgcaga  3868

exon11.USP18.human                                                              ------------------------------------------------------------  0
LOC102725072.(18846286-18861064new.ref                                          caggggtcagggg-ataggtccgtggtgctgggggtctggtgggagcagaggggcacccc  9925
LOC102725072.start-FAM230F.start.18846286-18865042.NEW.ref                      caggggtcagggg-ataggtccgtggtgctgggggtctggtgggagcagaggggcacccc  9925
chimp.100000-160000.Pan.troglodytes.isolate.Yerkes.chimp.pedigree.#C0471        caggggtcaggggaaaaggtccttggtgctgggggtctggtgggagcagaggggcacccc  13375
BCRP2.ref.rev.compl                                                             caggggtcgggggaaaaggtccttggtgctgggggtctggtgggagcagaggggcacccc  3928

exon11.USP18.human                                                              ------------------------------------------------------------  0
LOC102725072.(18846286-18861064new.ref                                          acggcctggagacctggtgtcctgggcagccacaagagagctgggctacctttccaggcg  9985
LOC102725072.start-FAM230F.start.18846286-18865042.NEW.ref                      acggcctggagacctggtgtcctgggcagccacaagagagctgggctacctttccaggcg  9985
chimp.100000-160000.Pan.troglodytes.isolate.Yerkes.chimp.pedigree.#C0471        acggcctggagacctggagtcctgggcagccacaagcgagctgggctacctttccaggtg  13435
BCRP2.ref.rev.compl                                                             acggcctggagacctggagtcctgggcagccacaagagagctgggctacctttccaggtg  3988

exon11.USP18.human                                                              ------------------------------------------------------------  0
LOC102725072.(18846286-18861064new.ref                                          gtctagaaggaaaaggaaggtgagcaggttggcctccggcagggacgacaacaggttgag  10045
LOC102725072.start-FAM230F.start.18846286-18865042.NEW.ref                      gtctagaaggaaaaggaaggtgagcaggttggcctccggcagggacgacaacaggttgag  10045
chimp.100000-160000.Pan.troglodytes.isolate.Yerkes.chimp.pedigree.#C0471        gtctagaaggaaaaggaaggtgagcaggttggcctccggcagggacgacagcaggttgag  13495
BCRP2.ref.rev.compl                                                             gtctagaaggaaaaggaaggtgagcaggttggcctccggcagggacgacagcaggttgag  4048

exon11.USP18.human                                                              ------------------------------------------------------------  0
LOC102725072.(18846286-18861064new.ref                                          catgcagctcttctttgcaactgggtctgaaagagctgcaggaggcagtgggtcactccc  10105
LOC102725072.start-FAM230F.start.18846286-18865042.NEW.ref                      catgcagctcttctttgcaactgggtctgaaagagctgcaggaggcagtgggtcactccc  10105
chimp.100000-160000.Pan.troglodytes.isolate.Yerkes.chimp.pedigree.#C0471        catgcagctcttctttgcaactgggtctgaaagagctgcaggaggcagtgggttactcct  13555
BCRP2.ref.rev.compl                                                             catgcagctcttctttgcaactgggtctgaaagagctgcaggaggcagtgggtcactccc  4108

exon11.USP18.human                                                              ------------------------------------------------------------  0
LOC102725072.(18846286-18861064new.ref                                          ctgggttacgacaagccggagacctctcccgaggtggtcacatggagcgcccgggacacg  10165
LOC102725072.start-FAM230F.start.18846286-18865042.NEW.ref                      ctgggttacgacaagccggagacctctcccgaggtggtcacatggagcgcccgggacacg  10165
chimp.100000-160000.Pan.troglodytes.isolate.Yerkes.chimp.pedigree.#C0471        ctgggttacgacaagccagagacctctcccgaggtggtcacatggagtgcctgggacacg  13615
BCRP2.ref.rev.compl                                                             ctgggttacgacaagccggagacctctcccgaggtggtcacatggagcgccagggacacg  4168

exon11.USP18.human                                                              ------------------------------------------------------------  0
LOC102725072.(18846286-18861064new.ref                                          agtccttgcgcagtttaggcttgtcatcatcgtcacacccacagcgctggccgccagtga  10225
LOC102725072.start-FAM230F.start.18846286-18865042.NEW.ref                      agtccttgcgcagtttaggcttgtcatcatcgtcacacccacagcgctggccgccagtga  10225
chimp.100000-160000.Pan.troglodytes.isolate.Yerkes.chimp.pedigree.#C0471        agtccttgcacagtttaggcttgtcatcattgtcacacccacagcgctggccaccagtga  13675
BCRP2.ref.rev.compl                                                             agtccttgcgcagtttaggcttgtcatcatcgtcacacccacagcgctggccgccagtga  4228

exon11.USP18.human                                                              ------------------------------------------------------------  0
LOC102725072.(18846286-18861064new.ref                                          ggaccctgtgaggggcacctgtgtggggtgtgaaccacctgaacgccttttctctgcctc  10285
LOC102725072.start-FAM230F.start.18846286-18865042.NEW.ref                      ggaccctgtgaggggcacctgtgtggggtgtgaaccacctgaacgccttttctctgcctc  10285
chimp.100000-160000.Pan.troglodytes.isolate.Yerkes.chimp.pedigree.#C0471        ggaccctgtgaggggcacctgtgtggggtgtgaaccacctgaacgccttttctctacctc  13735
BCRP2.ref.rev.compl                                                             ggaccctgtgaggggcacctgtgtggggtgtgaaccacctgaacgccttttctctacctc  4288

exon11.USP18.human                                                              ------------------------------------------------------------  0
LOC102725072.(18846286-18861064new.ref                                          gcaggggtcagcagcacccggcaaacagcagcaggaggagccgctagagcagctgctcat  10345
LOC102725072.start-FAM230F.start.18846286-18865042.NEW.ref                      gcaggggtcagcagcacccggcaaacagcagcaggaggagccgctagagcagctgctcat  10345
chimp.100000-160000.Pan.troglodytes.isolate.Yerkes.chimp.pedigree.#C0471        acaggggtcagcagcacccggtgaacagcagcaggaggagccgctagagcagctgctgat  13795
BCRP2.ref.rev.compl                                                             gcaggggtcagcagcacccggcaaacagcagcaggaggagccgctagagcagctgctcat  4348

exon11.USP18.human                                                              ------------------------------------------------------------  0
LOC102725072.(18846286-18861064new.ref                                          gggcagagctgccctcgggcaactcctgccaccacccctccccagggagcccaaggcag  10405
LOC102725072.start-FAM230F.start.18846286-18865042.NEW.ref                      gggcagagctgccctcgggcaactcctgccaccacccctccccagggagcccaaggcag  10405
chimp.100000-160000.Pan.troglodytes.isolate.Yerkes.chimp.pedigree.#C0471        gggcagagctgcccttgggcaactcctgccaccacccctccccagggagctcaagacag  13855
BCRP2.ref.rev.compl                                                             gggcagagctgccctcgggcaactcctgccaccacccctccccagggagcccaaggcag  4408

exon11.USP18.human                                                              ------------------------------------------------------------  0
LOC102725072.(18846286-18861064new.ref                                          gggaggctcagcatggaatgaaacaggggagtgagggacacaaggaggtgggaagtggga  10465
LOC102725072.start-FAM230F.start.18846286-18865042.NEW.ref                      gggaggctcagcatggaatgaaacaggggagtgagggacacaaggaggtgggaagtggga  10465
chimp.100000-160000.Pan.troglodytes.isolate.Yerkes.chimp.pedigree.#C0471        gggaggctcagcatggaatgaaacaggggagtgagggacacaaggaggtgggaagtggga  13915
BCRP2.ref.rev.compl                                                             gggaggctcagcatggaatgaaacaggggagtgagggacacaaggaggtgggaagtggga  4468

exon11.USP18.human                                                              ------------------------------------------------------------  0
LOC102725072.(18846286-18861064new.ref                                          gggtcccagccccaccaagtacgcagagaacccctcgttgtcctggacaccacaggggca  10525
LOC102725072.start-FAM230F.start.18846286-18865042.NEW.ref                      gggtcccagccccaccaagtacgcagagaacccctcgttgtcctggacaccacaggggca  10525
chimp.100000-160000.Pan.troglodytes.isolate.Yerkes.chimp.pedigree.#C0471        gggtcccagccccaccaagtacgcagagacccccctcgttgtcctggacaccacaggggca  13975
BCRP2.ref.rev.compl                                                             gggtcccagccccaccaagtacgcagagacccccctcgacatcctggacaccacaggggca  4528

exon11.USP18.human                                                              ------------------------------------------------------------  0
LOC102725072.(18846286-18861064new.ref                                          cctgcaggctgggagaccaggtcctctgtgcatgggcccgggaggcagacctgccctaag  10585
LOC102725072.start-FAM230F.start.18846286-18865042.NEW.ref                      cctgcaggctgggagaccaggtcctctgtgcatgggcccgggaggcagacctgccctaag  10585
chimp.100000-160000.Pan.troglodytes.isolate.Yerkes.chimp.pedigree.#C0471        cctgcaggctgggagaccaggtcctctgtgcatgggcccgggaggcagacctgccctaag  14035
BCRP2.ref.rev.compl                                                             cctgcaggctgggagaccaggtcctctgtgcatgggcccgggaggcagacctgccctaag  4588

exon11.USP18.human                                                              ------------------------------------------------------------  0
LOC102725072.(18846286-18861064new.ref                                          ggtgatgcacaggctacaggtgctgcacgctccagcgcccactctagacatcagcctcca  10645
LOC102725072.start-FAM230F.start.18846286-18865042.NEW.ref                      ggtgatgcacaggctacaggtgctgcacgctccagcgcccactctagacatcagcctcca  10645
chimp.100000-160000.Pan.troglodytes.isolate.Yerkes.chimp.pedigree.#C0471        ggtgatgaacaggctacaggtgctgcacgctccagcgcccactctagacatcagcctcca  14095
BCRP2.ref.rev.compl                                                             ggtgatgcacaggctacaggtgctgcacgctccagcgcccactctagacatcagcctcca  4648

exon11.USP18.human                                                              ------------------------------------------------------------  0
LOC102725072.(18846286-18861064new.ref                                          ggttgactaagggtcaggtcatgtttgaaaccatgcttggctggaccaggacccatggca  10705
LOC102725072.start-FAM230F.start.18846286-18865042.NEW.ref                      ggttgactaagggtcaggtcatgtttgaaaccatgcttggctggaccaggacccatggca  10705
chimp.100000-160000.Pan.troglodytes.isolate.Yerkes.chimp.pedigree.#C0471        ggttgactaagggtcaggtcatgtttgaaaccatgcttggctggaccaggacccatggca  14155
BCRP2.ref.rev.compl                                                             ggttgactaagggtcaggtcatgtttgaaaccatgcttggctggaccaggacccatggca  4708

exon11.USP18.human                                                              ------------------------------------------------------------  0
LOC102725072.(18846286-18861064new.ref                                          agagcacctgggcaccagtgtttagccctggtctgcaggaaggaggacagcagactttag  10765
LOC102725072.start-FAM230F.start.18846286-18865042.NEW.ref                      agagcacctgggcaccagtgtttagccctggtctgcaggaaggaggacagcagactttag  10765
chimp.100000-160000.Pan.troglodytes.isolate.Yerkes.chimp.pedigree.#C0471        agagcacctgggcaccagtgtttagccctggtctgcaggaaggaggacagcagactttag  14215
```

```
BCRP2.ref.rev.compl                                                             agagcacctgggcaccagtgtttagccctggtctgcaggaaggaggacagcagactttag  4768

exon11.USP18.human                                                              ------------------------------------------------------------  0
LOC102725072.(18846286-18861064new.ref                                          gaccccacagcacggcagtgctgaccatttcacccacttggcctccttgagaaatatgga  10825
LOC102725072.start-FAM230F.start.18846286-18865042.NEW.ref                      gaccccacagcacggcagtgctgaccatttcacccacttggcctccttgagaaatatgga  10825
chimp.100000-160000.Pan.troglodytes.isolate.Yerkes.chimp.pedigree.#C0471        gaccccacagcacggcagtgctgaccatttcacccacttggcctccttgagaaatatgga  14275
BCRP2.ref.rev.compl                                                             gaccccacagcacggcagtgctgaccatttcacccacttggcctccttgagaaatatgga  4828

exon11.USP18.human                                                              ------------------------------------------------------------  0
LOC102725072.(18846286-18861064new.ref                                          tggggacccctctggggatgggcaaggccttccaggataggctcagttttggtcccctgc  10885
LOC102725072.start-FAM230F.start.18846286-18865042.NEW.ref                      tggggacccctctggggatgggcaaggccttccaggataggctcagttttggtcccctgc  10885
chimp.100000-160000.Pan.troglodytes.isolate.Yerkes.chimp.pedigree.#C0471        tggggagccctctggggatgggcaaggccttccaggataggctcagttttggtcccctgc  14335
BCRP2.ref.rev.compl                                                             tggggagccctctggggatgggcaaggccttccaggataggctcagttttggtcccctgc  4888

exon11.USP18.human                                                              ------------------------------------------------------------  0
LOC102725072.(18846286-18861064new.ref                                          tttttgaggttgggttaaaattccgaccatggcagaggaagcacagctcaggttcccaca  10945
LOC102725072.start-FAM230F.start.18846286-18865042.NEW.ref                      tttttgaggttgggttaaaattccgaccatggcagaggaagcacagctcaggttcccaca  10945
chimp.100000-160000.Pan.troglodytes.isolate.Yerkes.chimp.pedigree.#C0471        tttttgaggttgggttaaaattccgaccatggcagaggaagcacagctcaggttcccaca  14395
BCRP2.ref.rev.compl                                                             tttttgaggttgggttaaaattccgaccatggcagaggaagcacagctcgggttcccaca  4948

exon11.USP18.human                                                              ------------------------------------------------------------  0
LOC102725072.(18846286-18861064new.ref                                          cctcacttttcacagcctctgagggcagcagtgcacgtggaggagacgtctcccatgagg  11005
LOC102725072.start-FAM230F.start.18846286-18865042.NEW.ref                      cctcacttttcacagcctctgagggcagcagtgcacgtggaggagacgtctcccatgagg  11005
chimp.100000-160000.Pan.troglodytes.isolate.Yerkes.chimp.pedigree.#C0471        cctcacttttcacagcctctgagggcagcagtgcacgtggaggagacgtctcccatgagg  14455
BCRP2.ref.rev.compl                                                             cctcacttttcacagcctctgagggcagcagtgcacgtggaggagacgtctcccatgagg  5008

exon11.USP18.human                                                              ------------------------------------------------------------  0
LOC102725072.(18846286-18861064new.ref                                          ccaaggcctccagtgctcaccgatgccctctgcgaagttggggtagaactcgtcagtgaa  11065
LOC102725072.start-FAM230F.start.18846286-18865042.NEW.ref                      ccaaggcctccagtgctcaccgatgccctctgcgaagttggggtagaactcgtcagtgaa  11065
chimp.100000-160000.Pan.troglodytes.isolate.Yerkes.chimp.pedigree.#C0471        ccaaggcctccagtgctcaccgatgccctctgtgaagttgggatagaactcatcagtgaa  14515
BCRP2.ref.rev.compl                                                             ccaaggcctccagtgctcaccgatgccctctgcgaagttggggtagaactcgtcagtgaa  5068

exon11.USP18.human                                                              ------------------------------------------------------------  0
LOC102725072.(18846286-18861064new.ref                                          gaggggctcgggcagctcacggaaatacagcttcagcgtgcctgcgatggcgttcacgtc  11125
LOC102725072.start-FAM230F.start.18846286-18865042.NEW.ref                      gaggggctcgggcagctcacggaaatacagcttcagcgtgcctgcgatggcgttcacgtc  11125
chimp.100000-160000.Pan.troglodytes.isolate.Yerkes.chimp.pedigree.#C0471        gaggggctcgggcagctcacggaagtacagcttcagcgtgcctgcaatggcgttcacgtc  14575
BCRP2.ref.rev.compl                                                             gaggggctcgggcagctcacggaagtacagcttcagcatgcctgcgatggcgttcacgtc  5128

exon11.USP18.human                                                              ------------------------------------------------------------  0
LOC102725072.(18846286-18861064new.ref                                          catctcgctcatcatcaccgacacgtccttgttatctggaaagagcacggaaatgcagcg  11185
LOC102725072.start-FAM230F.start.18846286-18865042.NEW.ref                      catctcgctcatcatcaccgacacgtccttgttatctggaaagagcacggaaatgcagcg  11185
chimp.100000-160000.Pan.troglodytes.isolate.Yerkes.chimp.pedigree.#C0471        catctcgctcatcatcactgacacgtccttgttatctggaaagagcacggaaatgcagcg  14635
BCRP2.ref.rev.compl                                                             catctcgctcatcatcactgacacgtccttgttatctggaaagagcacggaaatgcagcg  5188

exon11.USP18.human                                                              ------------------------------------------------------------  0
LOC102725072.(18846286-18861064new.ref                                          gcctccttgaagatcctgagtgagtcacccaccatccctgccttggctaaagcaccgtcc  11245
LOC102725072.start-FAM230F.start.18846286-18865042.NEW.ref                      gcctccttgaagatcctgagtgagtcacccaccatccctgccttggctaaagcaccgtcc  11245
chimp.100000-160000.Pan.troglodytes.isolate.Yerkes.chimp.pedigree.#C0471        gcctccttaaagatcctgagtgagtcacccaccatccctgccttggctaaagcaccgtcc  14695
BCRP2.ref.rev.compl                                                             gcctccttgaagatcctgagtgagtcacccaccatccctgccttggctaaagcaccgtcc  5248

exon11.USP18.human                                                              ------------------------------------------------------------  0
LOC102725072.(18846286-18861064new.ref                                          ctgccatcctgaccactgtgtgggtccctcctgggctttgagcagctcatctgactcctc  11305
LOC102725072.start-FAM230F.start.18846286-18865042.NEW.ref                      ctgccatcctgaccactgtgtgggtccctcctgggctttgagcagctcatctgactcctc  11305
chimp.100000-160000.Pan.troglodytes.isolate.Yerkes.chimp.pedigree.#C0471        ctgccatgctgaccactgtgcggatccctcctgggctttgagcagctcatctgactcctc  14755
BCRP2.ref.rev.compl                                                             ctgccatgctgaccactgtgtgggtccctcctgggctttgagcagctcatctgactcctc  5308

exon11.USP18.human                                                              ------------------------------------------------------------  0
LOC102725072.(18846286-18861064new.ref                                          ccaagagctgtgcatggttctgtgtctgcagagttgataggggtgcgtgggcattcccat  11365
LOC102725072.start-FAM230F.start.18846286-18865042.NEW.ref                      ccaagagctgtgcatggttctgtgtctgcagagttgataggggtgcgtgggcattcccat  11365
chimp.100000-160000.Pan.troglodytes.isolate.Yerkes.chimp.pedigree.#C0471        ccaagagctgtgcatggttctgtgtctgcagagttgataggggtgcgtgggcattcccat  14815
BCRP2.ref.rev.compl                                                             ccaagagctgtgcatggttctgtgtctgcagagttgataggggtgcgtgggcattcccat  5368

exon11.USP18.human                                                              ------------------------------------------------------------  0
LOC102725072.(18846286-18861064new.ref                                          tcctctcccctgcttggcctgatgtgatggccaggaggaggccagcatggcaggacacag  11425
LOC102725072.start-FAM230F.start.18846286-18865042.NEW.ref                      tcctctcccctgcttggcctgatgtgatggccaggaggaggccagcatggcaggacacag  11425
chimp.100000-160000.Pan.troglodytes.isolate.Yerkes.chimp.pedigree.#C0471        tcctctcccctgcttggcctgatgtgatggccaggaggaggccagcatggcaggacacag  14875
BCRP2.ref.rev.compl                                                             tcctctcccctgcttggcctgatgtgatggccaggaggaggccagcatggcaggacacag  5428

exon11.USP18.human                                                              ------------------------------------------------------------  0
LOC102725072.(18846286-18861064new.ref                                          cgcctgcgtggggattgggtggctctgccctgtacatagcaaccacccctgcaccagtgt  11485
LOC102725072.start-FAM230F.start.18846286-18865042.NEW.ref                      cgcctgcgtggggattgggtggctctgccctgtacatagcaaccacccctgcaccagtgt  11485
chimp.100000-160000.Pan.troglodytes.isolate.Yerkes.chimp.pedigree.#C0471        cgcctgcgtggggattgggtggctctgccctgtacatagcaaccacccctgcaccagtgt  14935
BCRP2.ref.rev.compl                                                             cgcctgcgtggggattgggtggctctgccctgtacatagcaaccacccctgcaccagtgt  5488

exon11.USP18.human                                                              ------------------------------------------------------------  0
LOC102725072.(18846286-18861064new.ref                                          catctgatagcaggaaggccgtgggagaatctgattggtttcagtgtttgaaccggtgtc  11545
LOC102725072.start-FAM230F.start.18846286-18865042.NEW.ref                      catctgatagcaggaaggccgtgggagaatctgattggtttcagtgtttgaaccggtgtc  11545
chimp.100000-160000.Pan.troglodytes.isolate.Yerkes.chimp.pedigree.#C0471        cttctgatagcaggaaggccgtgggagaatatgattggtttcagtgtttgaaccggtgtc  14995
BCRP2.ref.rev.compl                                                             cttctgatagcaggaaggccgtgggagaatctgattggtttcagtgtttgaaccggtgtc  5548

exon11.USP18.human                                                              ------------------------------------------------------------  0
LOC102725072.(18846286-18861064new.ref                                          ttcctttggacccaattggccattggtgcttacatcctcaccacaggccaggttcattct  11605
LOC102725072.start-FAM230F.start.18846286-18865042.NEW.ref                      ttcctttggacccaattggccattggtgcttacatcctcaccacaggccaggttcattct  11605
chimp.100000-160000.Pan.troglodytes.isolate.Yerkes.chimp.pedigree.#C0471        ttcctttggacccaattggccattggtgcttacatcctcaccacaggccaggtccattct  15055
BCRP2.ref.rev.compl                                                             ttcctttggacccaattggccattggtgcttacatcctcaccacaggccaggttcattct  5608

exon11.USP18.human                                                              ------------------------------------------------------------  0
LOC102725072.(18846286-18861064new.ref                                          gggccctcagagggagctgaaactaccacagggccctcccagggatgctgggcattctag  11665
LOC102725072.start-FAM230F.start.18846286-18865042.NEW.ref                      gggccctcagagggagctgaaactaccacagggccctcccagggatgctgggcattctag  11665
chimp.100000-160000.Pan.troglodytes.isolate.Yerkes.chimp.pedigree.#C0471        gggccctcagagggagctgaaactaccac-gggccctcccagggatgctgggcattctag  15114
BCRP2.ref.rev.compl                                                             gggccctcagagggagctgaaactaccacagggccctcccagggatgctgggcattctag  5668

exon11.USP18.human                                                              ------------------------------------------------------------  0
LOC102725072.(18846286-18861064new.ref                                          gggtcctggtcagggtgggtggtctgtgctgcaaagaagggtctgcaggcacaaaatcct  11725
LOC102725072.start-FAM230F.start.18846286-18865042.NEW.ref                      gggtcctggtcagggtgggtggtctgtgctgcaaagaagggtctgcaggcacaaaatcct  11725
chimp.100000-160000.Pan.troglodytes.isolate.Yerkes.chimp.pedigree.#C0471        gtgtcctggtcagggtgggtggtgtgtgctgcaaagaagggtctgcaggcacaaaatcct  15174
BCRP2.ref.rev.compl                                                             gggtcctggtcagggtgggtggtgtgtgctgcaaagaaggatctgcaggcacaaaatcct  5728

exon11.USP18.human                                                              ------------------------------------------------------------  0
LOC102725072.(18846286-18861064new.ref                                          gttgctttgaagatgctgggaaggaccctctggggtctcagtgccctcccctggcatttg  11785
LOC102725072.start-FAM230F.start.18846286-18865042.NEW.ref                      gttgctttgaagatgctgggaaggaccctctggggtctcagtgccctcccctggcatttg  11785
chimp.100000-160000.Pan.troglodytes.isolate.Yerkes.chimp.pedigree.#C0471        gttgctttgaagatgctgggaaggaccctctggggtctcagtgccctcccctggcatttg  15234
BCRP2.ref.rev.compl                                                             gttgctttgaagatgctgggaaggaccctctggggtctcagtgccctcccctggcatttg  5788

exon11.USP18.human                                                              ------------------------------------------------------------  0
LOC102725072.(18846286-18861064new.ref                                          aggcaggtccgggtccttcaaagcctgtgagggttggtgagatggaggcggagaggctgc  11845
LOC102725072.start-FAM230F.start.18846286-18865042.NEW.ref                      aggcaggtccgggtccttcaaagcctgtgagggttggtgagatggaggcggagaggctgc  11845
chimp.100000-160000.Pan.troglodytes.isolate.Yerkes.chimp.pedigree.#C0471        aggcaggtccgggtccttcaaagcctgtgagggttggtgagatggaggcggagaggctgc  15294
BCRP2.ref.rev.compl                                                             aggcaggtccgggtccttcaaagcctgtgagggttggtgagatggaggcggagaggctgc  5848

exon11.USP18.human                                                              ------------------------------------------------------------  0
LOC102725072.(18846286-18861064new.ref                                          agccccggcctgcgctgaatttcatcagtgccctctgcccaccacatcctcatacagggc  11905
LOC102725072.start-FAM230F.start.18846286-18865042.NEW.ref                      agccccggcctgcgctgaatttcatcagtgccctctgcccaccacatcctcatacagggc  11905
chimp.100000-160000.Pan.troglodytes.isolate.Yerkes.chimp.pedigree.#C0471        agccccggcctgcactgaatttcatcagtgccctctgcccaccacatcctcatacagggc  15354
BCRP2.ref.rev.compl                                                             agccccggcctgcgctgaatttcatcagtgccctctgcccaccacatcctcatacagggc  5908

exon11.USP18.human                                                              ------------------------------------------------------------  0
LOC102725072.(18846286-18861064new.ref                                          agtggacagaccgcactgagtcctgggcttccacctcctgtccaaccccaaggcaggaag  11965
LOC102725072.start-FAM230F.start.18846286-18865042.NEW.ref                      agtggacagaccgcactgagtcctgggcttccacctcctgtccaaccccaaggcaggaag  11965
chimp.100000-160000.Pan.troglodytes.isolate.Yerkes.chimp.pedigree.#C0471        agtggacagaccgcactgagtcctgggcttccacctcctgtccacccccaaggcaggaag  15414
BCRP2.ref.rev.compl                                                             agtggacagaccgcactgagtcctgggcttccacctcctgtccaaccccaaggcaggaag  5968

exon11.USP18.human                                                              ------------------------------------------------------------  0
LOC102725072.(18846286-18861064new.ref                                          gccaaggccccgcagaagcccctggtccactgcaccaagtggcacgagtgggtacgatgg  12025
```

```
LOC102725072.start-FAM230F.start.18846286-18865042.NEW.ref                 gccaaggccccgcagaagcccctggtccactgcaccaagtggcacgagtgggtacgatgg  12025
chimp.100000-160000.Pan.troglodytes.isolate.Yerkes.chimp.pedigree.#C0471   gccaagtccccacagaagcccctggtccaccgcaccaagtgccacgagtgggtacgatgg  15474
BCRP2.ref.rev.compl                                                        gccaaggccccgcagaagcccctggtccactgcaccaagtggcacgagtgggtacgatgg  6028


exon11.USP18.human                                                         ------------------------------------------------------------  0
LOC102725072.(18846286-18861064new.ref                                     tgtaaaaactggcttctatagaagctgtttgtacaactcttgttttctctttttaaaaa  12085
LOC102725072.start-FAM230F.start.18846286-18865042.NEW.ref                 tgtaaaaactggcttctatagaagctgtttgtacaactcttgttttctctttttaaaaa  12085
chimp.100000-160000.Pan.troglodytes.isolate.Yerkes.chimp.pedigree.#C0471   tgtaaaaactggcttctatagaagctgtttgtacaactcttgttttctctttttaaaaa  15534
BCRP2.ref.rev.compl                                                        tgtaaaaactggcttctatagaagctgtttgtacaactcttgttttctctttttaaaaa  6088


exon11.USP18.human                                                         ------------------------------------------------------------  0
LOC102725072.(18846286-18861064new.ref                                     taataaaacagtaaatgaagaaaagacacagagaaggatgtgacatgcctgggccgtgga  12145
LOC102725072.start-FAM230F.start.18846286-18865042.NEW.ref                 taataaaacagtaaatgaagaaaagacacagagaaggatgtgacatgcctgggccgtgga  12145
chimp.100000-160000.Pan.troglodytes.isolate.Yerkes.chimp.pedigree.#C0471   taataaaacagtaaatgaagaaaagacacagagaaggatgtgacatgcctgggccatgga  15594
BCRP2.ref.rev.compl                                                        taataaaacagtaaatgaagaaaagacacagagaaggatgtgacatgcctgggccatgga  6148


exon11.USP18.human                                                         ------------------------------------------------------------  0
LOC102725072.(18846286-18861064new.ref                                     gcactctgagatctcatcgcggacaccactgcccacacctccatcccgtcctgcgcaggc  12205
LOC102725072.start-FAM230F.start.18846286-18865042.NEW.ref                 gcactctgagatctcatcgcggacaccactgcccacacctccatcccgtcctgcgcaggc  12205
chimp.100000-160000.Pan.troglodytes.isolate.Yerkes.chimp.pedigree.#C0471   gcactctgagatctcatcgtggacaccactgcccacacctccatcccgtcctgcgcaggc  15654
BCRP2.ref.rev.compl                                                        gcactctgagatctcatcgtggacaccactgcccacacctccatcccgtcctgcgcaggc  6208


exon11.USP18.human                                                         ------------------------------------------------------------  0
LOC102725072.(18846286-18861064new.ref                                     cgacactcactgacgttgaaggctgccttcagtgcctggatgtctgcggccac----ccc  12261
LOC102725072.start-FAM230F.start.18846286-18865042.NEW.ref                 cgacactcactgacgttgaaggctgccttcagtgcctggatgtctgcggccac----ccc  12261
chimp.100000-160000.Pan.troglodytes.isolate.Yerkes.chimp.pedigree.#C0471   cgacactcactggcgtcgaaggctgccttcagtgcctggatgtctgcggccacaccggcc  15714
BCRP2.ref.rev.compl                                                        cgacactcactgacgttgaagcctgccttcagtgcctggatgtctgcggccac----ccc  6264


exon11.USP18.human                                                         ------------------------------------------------------------  0
LOC102725072.(18846286-18861064new.ref                                     agacatgcggtagatgcccacctcctccatgcctcggcgctcgatctcctccacgcactg  12321
LOC102725072.start-FAM230F.start.18846286-18865042.NEW.ref                 agacatgcggtagatgcccacctcctccatgcctcggcgctcgatctcctccacgcactg  12321
chimp.100000-160000.Pan.troglodytes.isolate.Yerkes.chimp.pedigree.#C0471   ggacacgcggtagatgcccacctcctccatgcctcggcgctcgatctcctccacgcactg  15774
BCRP2.ref.rev.compl                                                        agacatgcggtagatgcccacctcctccatgcctcggcgctcgatctcctccacgcactg  6324


exon11.USP18.human                                                         ------------------------------------------------------------  0
LOC102725072.(18846286-18861064new.ref                                     gcgcacgatgtagggcaccttggacctctctcctgcgggaggagggaatgttctcagt  12381
LOC102725072.start-FAM230F.start.18846286-18865042.NEW.ref                 gcgcacgatgtagggcaccttggacctctctcctgcgggaggagggaatgttctcagt  12381
chimp.100000-160000.Pan.troglodytes.isolate.Yerkes.chimp.pedigree.#C0471   gcgcacgatgtagggcaccttggacctctctcctgcgggaggagggaatgctctcagt  15834
BCRP2.ref.rev.compl                                                        acgcacgatgtagggcaccttggacctctctcctgcgggaggagggaatgttctcagt  6384


exon11.USP18.human                                                         ------------------------------------------------------------  0
LOC102725072.(18846286-18861064new.ref                                     gtcctaacagccctgcttgggccataacacaggagacctgctccctatgtgcacacccgg  12441
LOC102725072.start-FAM230F.start.18846286-18865042.NEW.ref                 gtcctaacagccctgcttgggccataacacaggagacctgctccctatgtgcacacccgg  12441
chimp.100000-160000.Pan.troglodytes.isolate.Yerkes.chimp.pedigree.#C0471   gtcctaacagccctgctggggccataacacaggagacctgctccctatctgcacacccgg  15894
BCRP2.ref.rev.compl                                                        gtcctaacagccctgcttgggccataacacaggagacctgctccctatctgcgcacccgg  6444


exon11.USP18.human                                                         ------------------------------------------------------------  0
LOC102725072.(18846286-18861064new.ref                                     aggtggggtgaggacggtgatgaaggtacccaggtctggggctgcacacagagccttctg  12501
LOC102725072.start-FAM230F.start.18846286-18865042.NEW.ref                 aggtggggtgaggacggtgatgaaggtacccaggtctggggctgcacacagagccttctg  12501
chimp.100000-160000.Pan.troglodytes.isolate.Yerkes.chimp.pedigree.#C0471   aggtggggtgaggacggtgacgaaggtacccaggtctggggctgcacacagagccttctg  15954
BCRP2.ref.rev.compl                                                        aggtggggtgaggacggtgacgaaggtacccaggtctggggctgcacacagagccttctg  6504


exon11.USP18.human                                                         ------------------------------------------------------------  0
LOC102725072.(18846286-18861064new.ref                                     catgcctgtcctccctctgcaagctctgtcctcattgcatgtgctttctcaggaaccttt  12561
LOC102725072.start-FAM230F.start.18846286-18865042.NEW.ref                 catgcctgtcctccctctgcaagctctgtcctcattgcatgtgctttctcaggaaccttt  12561
chimp.100000-160000.Pan.troglodytes.isolate.Yerkes.chimp.pedigree.#C0471   catgcctgtcctccctctgcaagctctgtcctcattgcatgtactttctcaggaaccttt  16014
BCRP2.ref.rev.compl                                                        catgcctgtcctccctctgcaagctctgtcctcattgcatgtactttctcaggaaccttt  6564


exon11.USP18.human                                                         ------------------------------------------------------------  0
LOC102725072.(18846286-18861064new.ref                                     caagcggccagaacccctgcaaatcacacatgacctttgtgggaaggtcaggaggcctgt  12621
LOC102725072.start-FAM230F.start.18846286-18865042.NEW.ref                 caagcggccagaacccctgcaaatcacacatgacctttgtgggaaggtcaggaggcctgt  12621
chimp.100000-160000.Pan.troglodytes.isolate.Yerkes.chimp.pedigree.#C0471   caagtggccagaacccctgcgaatcacgcatgatctttgcgggaaggtcaggaggcctgt  16074
BCRP2.ref.rev.compl                                                        caagcggccagaacccctgcgaatcacacatgacctttgtgggaaggtcaggaggcctgt  6624


exon11.USP18.human                                                         ------------------------------------------------------------  0
LOC102725072.(18846286-18861064new.ref                                     ctaagtcaagtcagcacgggaagggcatctgacagattccaggcctggggtgagcagcct  12681
LOC102725072.start-FAM230F.start.18846286-18865042.NEW.ref                 ctaagtcaagtcagcacgggaagggcatctgacagattccaggcctggggtgagcagcct  12681
chimp.100000-160000.Pan.troglodytes.isolate.Yerkes.chimp.pedigree.#C0471   ctaagtcaagtcagcacaggaagggcatctgacagattccaggcctggggttagctgcct  16134
BCRP2.ref.rev.compl                                                        ctaagtcaagtcagcacgggaagggcatctgacagattccaggcctggggttagcagcct  6684


exon11.USP18.human                                                         ------------------------------------------------------------  0
LOC102725072.(18846286-18861064new.ref                                     gtgcccccggctgggaggtcagacccggtgttggtcctgccacccacgtgctgtgtgaga  12741
LOC102725072.start-FAM230F.start.18846286-18865042.NEW.ref                 gtgcccccggctgggaggtcagacccggtgttggtcctgccacccacgtgctgtgtgaga  12741
chimp.100000-160000.Pan.troglodytes.isolate.Yerkes.chimp.pedigree.#C0471   gtgcccccagctgggagatcagacccggtgttggttctgccacccacgtgctgtgtgaga  16194
BCRP2.ref.rev.compl                                                        gtgcccccggctgggaggtcagacccggtgttggtcctgccacccacgtgctgtgtgaga  6744


exon11.USP18.human                                                         ------------------------------------------------------------  0
LOC102725072.(18846286-18861064new.ref                                     ggagaatccgtgacccctgccctgggccttaacacacatccgacgaatgaatgaagggtt  12801
LOC102725072.start-FAM230F.start.18846286-18865042.NEW.ref                 ggagaatccgtgacccctgccctgggccttaacacacatccgacgaatgaatgaagggtt  12801
chimp.100000-160000.Pan.troglodytes.isolate.Yerkes.chimp.pedigree.#C0471   ggagaatccctgacccctgccctgggccttaacacacatccgacgaatgaatgaagggtt  16254
BCRP2.ref.rev.compl                                                        ggagaatccctgacccctgccctgggccttaacacacatccgacgaatgaatgaagggtt  6804


exon11.USP18.human                                                         ------------------------------------------------------------  0
LOC102725072.(18846286-18861064new.ref                                     gcctcagcaccggtgctccaagtcctgcgatgctaagtgcttttctcctctgagtcttag  12861
LOC102725072.start-FAM230F.start.18846286-18865042.NEW.ref                 gcctcagcaccggtgctccaagtcctgcgatgctaagtgcttttctcctctgagtcttag  12861
chimp.100000-160000.Pan.troglodytes.isolate.Yerkes.chimp.pedigree.#C0471   gcctcagcaccggtgctccaagtcctgcgatgctaagtgcttttctcctctgagtcttag  16314
BCRP2.ref.rev.compl                                                        gcctcagcaccggtgctccaagtcctgcgatgctaagtgcttttctcctctgagtcttag  6864


exon11.USP18.human                                                         ------------------------------------------------------------  0
LOC102725072.(18846286-18861064new.ref                                     caatggacaattccaatacctccacacaggacactagagtaagaatccttcacagttaga  12921
LOC102725072.start-FAM230F.start.18846286-18865042.NEW.ref                 caatggacaattccaatacctccacacaggacactagagtaagaatccttcacagttaga  12921
chimp.100000-160000.Pan.troglodytes.isolate.Yerkes.chimp.pedigree.#C0471   caatggacaattccaatacctccacacaggacactagagtaagaatccttcacagttaga  16374
BCRP2.ref.rev.compl                                                        caatggacaattccaatacctccacacaggacactagagtaagaatccttcacagttaga  6924


exon11.USP18.human                                                         ------------------------------------------------------------  0
LOC102725072.(18846286-18861064new.ref                                     acgcagtgctgtgcggaggccttaacttgagttctgttttgcacctggatttaccagcac  12981
LOC102725072.start-FAM230F.start.18846286-18865042.NEW.ref                 acgcagtgctgtgcggaggccttaacttgagttctgttttgcacctggatttaccagcac  12981
chimp.100000-160000.Pan.troglodytes.isolate.Yerkes.chimp.pedigree.#C0471   acgcagtgctgtgcggaggccttaacttgagttctgttttgcacctggatttaccagcac  16434
BCRP2.ref.rev.compl                                                        acgcagtgctgtgcggaggccttaacttgagttctgttttgcacctggatttaccagcac  6984


exon11.USP18.human                                                         ------------------------------------------------------------  0
LOC102725072.(18846286-18861064new.ref                                     atcaaagctgcttcgcaagccccctcatcagcagggctcatgtgggggagctgctgatgg  13041
LOC102725072.start-FAM230F.start.18846286-18865042.NEW.ref                 atcaaagctgcttcgcaagccccctcatcagcagggctcatgtgggggagctgctgatgg  13041
chimp.100000-160000.Pan.troglodytes.isolate.Yerkes.chimp.pedigree.#C0471   atcaaagctgctttgcaagccccctcattagcagggcttatctgggggagctgctgatgg  16494
BCRP2.ref.rev.compl                                                        atcaaagctgcttcgcaagccccctcatcagcagggcttatgtgggggagctgctgatgg  7044


exon11.USP18.human                                                         ------------------------------------------------------------  0
LOC102725072.(18846286-18861064new.ref                                     agtcctcgctgctcatgcccacagccctcccagagtgctatgcgagtggctgccgtgcag  13101
LOC102725072.start-FAM230F.start.18846286-18865042.NEW.ref                 agtcctcgctgctcatgcccacagccctcccagagtgctatgcgagtggctgccgtgcag  13101
chimp.100000-160000.Pan.troglodytes.isolate.Yerkes.chimp.pedigree.#C0471   agtcctcgctgctcatgcccacagccctcccagagtgctatgcgagtggctgccgtgcag  16554
BCRP2.ref.rev.compl                                                        agtcctcgctgctcatgcccacagccctcccagagtgctatgcgagtggctgccgtgcag  7104


exon11.USP18.human                                                         ------------------------------------------------------------  0
LOC102725072.(18846286-18861064new.ref                                     ttgggggtggggcgtggtgtttagacacagataggagtccagggtatgactgatggaggc  13161
LOC102725072.start-FAM230F.start.18846286-18865042.NEW.ref                 ttgggggtggggcgtggtgtttagacacagataggagtccagggtatgactgatggaggc  13161
chimp.100000-160000.Pan.troglodytes.isolate.Yerkes.chimp.pedigree.#C0471   ttaggggtggggcgtggtatttagacacagataggagtccacggtatgactgatggaggc  16614
BCRP2.ref.rev.compl                                                        ttgggggtggggcgtggtgtttagacacagataggagtccagggtatgactgatggaggc  7164


exon11.USP18.human                                                         ------------------------------------------------------------  0
LOC102725072.(18846286-18861064new.ref                                     cccggcccacgtgaccagcaaggtcagaggcccagccagattccatcctgaggaagcaaa  13221
LOC102725072.start-FAM230F.start.18846286-18865042.NEW.ref                 cccggcccacgtgaccagcaaggtcagaggcccagccagattccatcctgaggaagcaaa  13221
chimp.100000-160000.Pan.troglodytes.isolate.Yerkes.chimp.pedigree.#C0471   cccggcccacgtgaccagcaaggtcagaggcccagccagattccatcctggggaagcaaa  16674
BCRP2.ref.rev.compl                                                        cccggcccacgtgaccagcaaggtcagaggcccagccagattccatcctggggaagcaaa  7224
```

```
exon11.USP18.human                                                       ------------------------------------------------------------  0
LOC102725072.(18846286-18861064new.ref                                   tgaattctcagaggaagtggtctgtgtctgcatgaactgctctcaaaccaacaaataggc  13281
LOC102725072.start-FAM230F.start.18846286-18865042.NEW.ref               tgaattctcagaggaagtggtctgtgtctgcatgaactgctctcaaaccaacaaataggc  13281
chimp.100000-160000.Pan.troglodytes.isolate.Yerkes.chimp.pedigree.#C0471 tgaattctcagaggaagtggtctgtgtctgcatgaactgctctcaaaccaacaaataggc  16734
BCRP2.ref.rev.compl                                                      tgaattctcagaggaagtggtctgtgtctgtatgaactgctctcaaaccaacaaataggc  7284

exon11.USP18.human                                                       ------------------------------------------------------------  0
LOC102725072.(18846286-18861064new.ref                                   ttctcttggcaactgactcgtgacaaagggttcaagattgtttgaaaaaaaaaaaggggg  13341
LOC102725072.start-FAM230F.start.18846286-18865042.NEW.ref               ttctcttggcaactgactcgtgacaaagggttcaagattgtttgaaaaaaaaaaaggggg  13341
chimp.100000-160000.Pan.troglodytes.isolate.Yerkes.chimp.pedigree.#C0471 ttttcttggcaactgactcgtgacaaagggttcaagattgtttg--aaaaaaaaa-aagg  16791
BCRP2.ref.rev.compl                                                      ttctcttggcaactgactcgtgacaaagggttcaagattgtttgaaaaaaaaaaa-aaag  7343

exon11.USP18.human                                                       ------------------------------------------------------------  0
LOC102725072.(18846286-18861064new.ref                                   gggggggacagggaggcagtaggtcctggaaaagtaaattctttattttacaataagaaa  13401
LOC102725072.start-FAM230F.start.18846286-18865042.NEW.ref               gggggggacagggaggcagtaggtcctggaaaagtaaattctttattttacaataagaaa  13401
chimp.100000-160000.Pan.troglodytes.isolate.Yerkes.chimp.pedigree.#C0471 ggggggaacagggaggcagtaggtcctggaaaagtaaattctttattttacaataagaaa  16851
BCRP2.ref.rev.compl                                                      ggggggaacagggaggcagtaggtcctggaaaagtaaattctttattttacaataagaaa  7403

exon11.USP18.human                                                       ------------------------------------------------------------  0
LOC102725072.(18846286-18861064new.ref                                   gtgattacatatttatttttttttttacaatggtggaaaattagaagtgattgtgaaaatg  13461
LOC102725072.start-FAM230F.start.18846286-18865042.NEW.ref               gtgattacatatttatttttttttttacaatggtggaaaattagaagtgattgtgaaaatg  13461
chimp.100000-160000.Pan.troglodytes.isolate.Yerkes.chimp.pedigree.#C0471 gtgattacatatttatttttttttttacaatggtggaaaattagaagtgattgtgaaaatg  16911
BCRP2.ref.rev.compl                                                      gtgattacatattta-tttttttttacaatggtggaaaattagaagtgattgtgaaaatg  7462

exon11.USP18.human                                                       ------------------------------------------------------------  0
LOC102725072.(18846286-18861064new.ref                                   atgtctacccgccttgctgatgagtaggatgtgatttggctctttaggaaactgaatttg  13521
LOC102725072.start-FAM230F.start.18846286-18865042.NEW.ref               atgtctacccgccttgctgatgagtaggatgtgatttggctctttaggaaactgaatttg  13521
chimp.100000-160000.Pan.troglodytes.isolate.Yerkes.chimp.pedigree.#C0471 atgtctacccgccttgctgatgagtaggatgtgatttggctctttaggaaactgaatttg  16971
BCRP2.ref.rev.compl                                                      atgtctacccgccttgctgatgagtaggatgtgatttggctctttaggaaactgaatttg  7522

exon11.USP18.human                                                       ------------------------------------------------------------  0
LOC102725072.(18846286-18861064new.ref                                   cagaacttaagaatattgatttataaagggcatggccattgacccagtccatcttatgca  13581
LOC102725072.start-FAM230F.start.18846286-18865042.NEW.ref               cagaacttaagaatattgatttataaagggcatggccattgacccagtccatcttatgca  13581
chimp.100000-160000.Pan.troglodytes.isolate.Yerkes.chimp.pedigree.#C0471 cagaacttaagaatattgatttataaagggcatggccattgacccagtccatcttatgca  17031
BCRP2.ref.rev.compl                                                      cagaacttaagaatattgatttataaagggcatggccattgacccagtccatcttatgca  7582

exon11.USP18.human                                                       ------------------------------------------------------------  0
LOC102725072.(18846286-18861064new.ref                                   aatctggatgccataaataatatttaaaaatgaaagtattggggtggaggttgcagtgag  13641
LOC102725072.start-FAM230F.start.18846286-18865042.NEW.ref               aatctggatgccataaataatatttaaaaatgaaagtattggggtggaggttgcagtgag  13641
chimp.100000-160000.Pan.troglodytes.isolate.Yerkes.chimp.pedigree.#C0471 aatctggatgccataaataatatttaaaaatgaaagtattggggtggaggttgcagtgag  17091
BCRP2.ref.rev.compl                                                      aatctggatgccataaataatatttaaaaatgaaagtattggggtggaggttgcagtgag  7642

exon11.USP18.human                                                       ------------------------------------------------------------  0
LOC102725072.(18846286-18861064new.ref                                   cggagatcgccccactacactccagcctggtcaacagagtgagactctgtctcaaaac-a  13700
LOC102725072.start-FAM230F.start.18846286-18865042.NEW.ref               cggagatcgccccactacactccagcctggtcaacagagtgagactctgtctcaaaac-a  13700
chimp.100000-160000.Pan.troglodytes.isolate.Yerkes.chimp.pedigree.#C0471 cggagatcgccccactacactccagcctggtcaacagagtgagactctgtctcaaaacaa  17151
BCRP2.ref.rev.compl                                                      cggagatcgccccactacactccagcctggtcaacagagtgagactctgtctcaaaacaa  7702

exon11.USP18.human                                                       ------------------------------------------------------------  0
LOC102725072.(18846286-18861064new.ref                                   aaaaaaaaaaaaaaaaaagaagaagtcattcccaacattgctcatcaaattatgaccata  13760
LOC102725072.start-FAM230F.start.18846286-18865042.NEW.ref               aaaaaaaaaaaaaaaaaagaagaagtcattcccaacattgctcatcaaattatgaccata  13760
chimp.100000-160000.Pan.troglodytes.isolate.Yerkes.chimp.pedigree.#C0471 aaaaaaaaaaaaaaaaaagaagaagtcattcccaacattgctcatcaaattatgaccata  17211
BCRP2.ref.rev.compl                                                      aaaaaaaaaaaaaaaaaggaagaagtcattcccaacattgctcatcaaattatgaccata  7762

exon11.USP18.human                                                       ------------------------------------------------------------  0
LOC102725072.(18846286-18861064new.ref                                   aatttccaggatcagactaacggctaaagagactgatgcatcaacaccaggcagagaata  13820
LOC102725072.start-FAM230F.start.18846286-18865042.NEW.ref               aatttccaggatcagactaacggctaaagagactgatgcatcaacaccaggcagagaata  13820
chimp.100000-160000.Pan.troglodytes.isolate.Yerkes.chimp.pedigree.#C0471 aatttccaggatcagactaacggctaaagagactgatgcatcaacaccaggcagagaata  17271
BCRP2.ref.rev.compl                                                      aatttccaggatcagactaacggctaaagagactgatgcatcaacaccaggcagagaata  7822

exon11.USP18.human                                                       ------------------------------------------------------------  0
LOC102725072.(18846286-18861064new.ref                                   aagcagattttttttgttttgttttggagagcctctaggaacttgaaaaatacatatgcc  13880
LOC102725072.start-FAM230F.start.18846286-18865042.NEW.ref               aagcagattttttttgttttgttttggagagcctctaggaacttgaaaaatacatatgcc  13880
chimp.100000-160000.Pan.troglodytes.isolate.Yerkes.chimp.pedigree.#C0471 aagcagatttttttgttgttgttctggagagcttctaggaacttgaaaaatacatatgcc  17331
BCRP2.ref.rev.compl                                                      aagcagattttttttgttttgttttggagagcctctaggaacttgaaaaatacatatgcc  7882

exon11.USP18.human                                                       ------------------------------------------------------------  0
LOC102725072.(18846286-18861064new.ref                                   acactcttaagacccggtggttcttaatcagggatgttcattaaaatgctggaaaactct  13940
LOC102725072.start-FAM230F.start.18846286-18865042.NEW.ref               acactcttaagacccggtggttcttaatcagggatgttcattaaaatgctggaaaactct  13940
chimp.100000-160000.Pan.troglodytes.isolate.Yerkes.chimp.pedigree.#C0471 acactcttaagacccggtggttcttaatcagggatgttcattaaaatgctggaaaactgt  17391
BCRP2.ref.rev.compl                                                      acactcttaagacccggtggttcttaatcagggatgttcattaaaatgctggaaaactgt  7942

exon11.USP18.human                                                       ------------------------------------------------------------  0
LOC102725072.(18846286-18861064new.ref                                   aaagatttccaggtcccatccaaggagattttgcttctgattgactggctagtggcctgg  14000
LOC102725072.start-FAM230F.start.18846286-18865042.NEW.ref               aaagatttccaggtcccatccaaggagattttgcttctgattgactggctagtggcctgg  14000
chimp.100000-160000.Pan.troglodytes.isolate.Yerkes.chimp.pedigree.#C0471 aaagacttccaggtcccatccaaggagattttgctgctgattgactggctagtggcctgg  17451
BCRP2.ref.rev.compl                                                      aaagacttccaggtcccatccaaggagattttgcttctgattgactggctagtggcctgg  8002

exon11.USP18.human                                                       ------------------------------------------------------------  0
LOC102725072.(18846286-18861064new.ref                                   ccattggtattttgaaaaatccctccaagtgattcttttacatcccagctagaaaaccct  14060
LOC102725072.start-FAM230F.start.18846286-18865042.NEW.ref               ccattggtattttgaaaaatccctccaagtgattcttttacatcccagctagaaaaccct  14060
chimp.100000-160000.Pan.troglodytes.isolate.Yerkes.chimp.pedigree.#C0471 ccattggtattttgaaaaatccctccaagtgattcttttacatcccagccagaaaaccct  17511
BCRP2.ref.rev.compl                                                      ccattggtattttgaaaaatccctccaagtgattcttttacatcccagctagaaaaccct  8062

exon11.USP18.human                                                       ------------------------------------------------------------  0
LOC102725072.(18846286-18861064new.ref                                   aaattaaaggtgaaaaaccagacaccaagtggcatttaaataaatgtcaactttaactcc  14120
LOC102725072.start-FAM230F.start.18846286-18865042.NEW.ref               aaattaaaggtgaaaaaccagacaccaagtggcatttaaataaatgtcaactttaactcc  14120
chimp.100000-160000.Pan.troglodytes.isolate.Yerkes.chimp.pedigree.#C0471 aaattaaaggtgaaaaaccagacaccaagtggcatttaaataaatgtcaactttaactcc  17571
BCRP2.ref.rev.compl                                                      aaattaaaggtgaaaaaccagacaccaagtggcatttaaataaatgtcaactttaactcc  8122

exon11.USP18.human                                                       ------------------------------------------------------------  0
LOC102725072.(18846286-18861064new.ref                                   acaaagcatctggttgcatgtggacagaaagagaaggaaagagggccctatatctggata  14180
LOC102725072.start-FAM230F.start.18846286-18865042.NEW.ref               acaaagcatctggttgcatgtggacagaaagagaaggaaagagggccctatatctggata  14180
chimp.100000-160000.Pan.troglodytes.isolate.Yerkes.chimp.pedigree.#C0471 acaaagcatctggttgcatgtggacagaaagagaaggaaagagggccctatatctggata  17631
BCRP2.ref.rev.compl                                                      acaaagcatctggttgcacgtggacagaaagagaaggaaagagggccctatatctggata  8182

exon11.USP18.human                                                       ------------------------------------------------------------  0
LOC102725072.(18846286-18861064new.ref                                   acttggaaatgtgctccccctagcaagatatctaccaaaattaaaaccatatttgaggat  14240
LOC102725072.start-FAM230F.start.18846286-18865042.NEW.ref               acttggaaatgtgctccccctagcaagatatctaccaaaattaaaaccatatttgaggat  14240
chimp.100000-160000.Pan.troglodytes.isolate.Yerkes.chimp.pedigree.#C0471 acttggaaatgtgctccccctagcaagatatctaccaaaattaaaaccatatttgaggat  17691
BCRP2.ref.rev.compl                                                      acttggaaatgtgctccccctagcaagatatctaccaaaattaaaaccatatttgaggat  8242

exon11.USP18.human                                                       ------------------------------------------------------------  0
LOC102725072.(18846286-18861064new.ref                                   gctggcactgtgagcaatatataaatgatgcatgtaacatcatttaatatgatcttattt  14300
LOC102725072.start-FAM230F.start.18846286-18865042.NEW.ref               gctggcactgtgagcaatatataaatgatgcatgtaacatcatttaatatgatcttattt  14300
chimp.100000-160000.Pan.troglodytes.isolate.Yerkes.chimp.pedigree.#C0471 gctggcactgcgagcaatatataaatgatgcatgtaacatcacttaatatgatcttattt  17751
BCRP2.ref.rev.compl                                                      gctggcactgcgagcaatatataaatgatgcatgtaacatcatttaatatgatcttattt  8302

exon11.USP18.human                                                       ------------------------------------------------------------  0
LOC102725072.(18846286-18861064new.ref                                   ttaaaaatgagtagagtggtgttttctagctgttagtgtttccaaatatcaatgtagaaa  14360
LOC102725072.start-FAM230F.start.18846286-18865042.NEW.ref               ttaaaaatgagtagagtggtgttttctagctgttagtgtttccaaatatcaatgtagaaa  14360
chimp.100000-160000.Pan.troglodytes.isolate.Yerkes.chimp.pedigree.#C0471 ttaaaaatgagtagagtggtgttttctagctgttagtttttccaaatatcaatatagaaa  17811
BCRP2.ref.rev.compl                                                      ttaaaaatgagtagagtggtgttttctagctgttagtgtttccaaatatcaatgtagaaa  8362

exon11.USP18.human                                                       ------------------------------------------------------------  0
LOC102725072.(18846286-18861064new.ref                                   ttagccttctgcagctgcagaggcaattcagtttgcagcttgcttgcatgtggcctagag  14420
LOC102725072.start-FAM230F.start.18846286-18865042.NEW.ref               ttagccttctgcagctgcagaggcaattcagtttgcagcttgcttgcatgtggcctagag  14420
chimp.100000-160000.Pan.troglodytes.isolate.Yerkes.chimp.pedigree.#C0471 ttagccttctgcagctgcagaggcaattcagtttgcagcttgcttgcatgtggcctagag  17871
BCRP2.ref.rev.compl                                                      ttagccttctgcagctgcagaggcaattcagtttgcagcttgcttgcatgtggcctagag  8422

exon11.USP18.human                                                       ------------------------------------------------------------  0
LOC102725072.(18846286-18861064new.ref                                   ccacccagcctgatatgtactaattttttatgtttaacttgccagagtagaaactcagtt  14480
LOC102725072.start-FAM230F.start.18846286-18865042.NEW.ref               ccacccagcctgatatgtactaattttttatgtttaacttgccagagtagaaactcagtt  14480
chimp.100000-160000.Pan.troglodytes.isolate.Yerkes.chimp.pedigree.#C0471 ccacccagcctgatatgtactaattttttatgtttaacttgccagagtagaaactcagtt  17931
BCRP2.ref.rev.compl                                                      ccacccagcctgatatgtactaattttt--tgtttaacttgccagagtagaaactcagtt  8480
```

```
exon11.USP18.human                                                          ------------------------------------------------------------  0
LOC102725072.(18846286-18861064new.ref                                      tctgggcgaggcatagtggctcatgcctgtaatcccagaactttgggaggccaaggcaag  14540
LOC102725072.start-FAM230F.start.18846286-18865042.NEW.ref                  tctgggcgaggcatagtggctcatgcctgtaatcccagaactttgggaggccaaggcaag  14540
chimp.100000-160000.Pan.troglodytes.isolate.Yerkes.chimp.pedigree.#C0471    tctgggccaggcgtagtgtctcatgcctgtaatcccagaactttgggaggccaaggcaag  17991
BCRP2.ref.rev.compl                                                         tctgggccaggcatagtggctcatgcctgtaatcccagaactttgggaggccaaggcaag  8540

exon11.USP18.human                                                          ------------------------------------------------------------  0
LOC102725072.(18846286-18861064new.ref                                      cagatcacaaggtcgggagttcaagaccagcctggctgacatagtgaaaccctatctttg  14600
LOC102725072.start-FAM230F.start.18846286-18865042.NEW.ref                  cagatcacaaggtcgggagttcaagaccagcctggctgacatagtgaaaccctatctttg  14600
chimp.100000-160000.Pan.troglodytes.isolate.Yerkes.chimp.pedigree.#C0471    cagatcacaaggtcgggagttcaagaccagcctggctgacatagtgaaaccctatctttg  18051
BCRP2.ref.rev.compl                                                         cagatcacaaggtcgggagttcaagaccagcctggctgacatagtgaaaccctatctttg  8600

exon11.USP18.human                                                          ------------------------------------------------------------  0
LOC102725072.(18846286-18861064new.ref                                      ctaaaaatacaaaaattagcccggcatggtggcaggcacctgtagtcccagctacttggg  14660
LOC102725072.start-FAM230F.start.18846286-18865042.NEW.ref                  ctaaaaatacaaaaattagcccggcatggtggcaggcacctgtagtcccagctacttggg  14660
chimp.100000-160000.Pan.troglodytes.isolate.Yerkes.chimp.pedigree.#C0471    ctaaaaatacaaaaattagcccggcatggtggcaggcacctgtagtcccagctacttggg  18111
BCRP2.ref.rev.compl                                                         ctaaaaatacaaaaattagcccggcatgctggcaggcacctgtagtcccagctacttggg  8660

exon11.USP18.human                                                          ------------------------------------------------------------  0
LOC102725072.(18846286-18861064new.ref                                      aggctgaggcaggagaatcacttgaacctgggaggcagaggttgtggtgaactgagatca  14720
LOC102725072.start-FAM230F.start.18846286-18865042.NEW.ref                  aggctgaggcaggagaatcacttgaacctgggaggcagaggttgtggtgaactgagatca  14720
chimp.100000-160000.Pan.troglodytes.isolate.Yerkes.chimp.pedigree.#C0471    aggctcaggcaggagaatcacttgaacctgggaggcagaggttgtggtgaactgagatca  18171
BCRP2.ref.rev.compl                                                         aggctgaggcaggagaatcacttgaacctgggaggcagaggttgtggtgaactgagatca  8720

exon11.USP18.human                                                          ------------------------------------------------------------  0
LOC102725072.(18846286-18861064new.ref                                      tgcccctgcactccagcctgggcaacagagtgagactccatctc-aaaaaaaaaaaaaaa  14779
LOC102725072.start-FAM230F.start.18846286-18865042.NEW.ref                  tgcccctgcactccagcctgggcaacagagtgagactccatctc-aaaaaaaaaaaaaaa  14779
chimp.100000-160000.Pan.troglodytes.isolate.Yerkes.chimp.pedigree.#C0471    tgcccctgcactccagcctgggcaacagagtgagactccatctcaaaaaaaaaaaaaaaa  18231
BCRP2.ref.rev.compl                                                         tgcccctgcactccagcctgggcaacagagtgagactccatctc--aaaaaaaaaaaaaa  8778
```

end of LOC102725072

```
exon11.USP18.human                                                          ------------------------------------------------------------  0
LOC102725072.(18846286-18861064new.ref                                      ------------------------------------------------------------  14779
LOC102725072.start-FAM230F.start.18846286-18865042.NEW.ref                  gaaactcagtttctggttacatctgatctttatttttatatatcatctaagctataaag  14839
chimp.100000-160000.Pan.troglodytes.isolate.Yerkes.chimp.pedigree.#C0471    gaaactcagtttctggttacatctgatctttatttttatatatcatctaagctataaag  18291
BCRP2.ref.rev.compl                                                         gaaactcagtttctggttacatctgatctttatttttatatatcatctaagctataaag  8838
```

start of BCRP2 in *linc-UR-B1*

```
exon11.USP18.human                                                          ------------------------------------------------------------  0
LOC102725072.(18846286-18861064new.ref                                      ------------------------------------------------------------  14779
LOC102725072.start-FAM230F.start.18846286-18865042.NEW.ref                  ttatattccctatttgtgatcttaaaagaaggactccaggaaagtgttcaaatattcata  14899
chimp.100000-160000.Pan.troglodytes.isolate.Yerkes.chimp.pedigree.#C0471    ttatattccctatttgtgatcttaaaagaaggactccaggaaagtgttcaaatattcata  18351
BCRP2.ref.rev.compl                                                         ttatattccctatttgtgatcttaaaagaaggactccaggaaagtgttcaaatattcata  8898

exon11.USP18.human                                                          ------------------------------------------------------------  0
LOC102725072.(18846286-18861064new.ref                                      ------------------------------------------------------------  14779
LOC102725072.start-FAM230F.start.18846286-18865042.NEW.ref                  tatctaaactggaacatatgtttatattttaaaagtagcctgagaggttggcaactaaa  14959
chimp.100000-160000.Pan.troglodytes.isolate.Yerkes.chimp.pedigree.#C0471    tatctaaactggaacatatgtttatatttttaaagtagcctgagaggttggcaactaaa  18411
BCRP2.ref.rev.compl                                                         tatctaaactggaacatatgtttatattttaaaagtagcctgagaggttggcaactaaa  8958

exon11.USP18.human                                                          ------------------------------------------------------------  0
LOC102725072.(18846286-18861064new.ref                                      ------------------------------------------------------------  14779
LOC102725072.start-FAM230F.start.18846286-18865042.NEW.ref                  gtcatatgttgaatgatcatttctcaagagtttcattttatggtctttctcttgttctgt  15019
chimp.100000-160000.Pan.troglodytes.isolate.Yerkes.chimp.pedigree.#C0471    gtcatatgttgaatgatcatttctcaagagtttcattttatggtctttctcttgttctgt  18471
BCRP2.ref.rev.compl                                                         gtcatatgttgaatgatcatttctcaagagtttcattttatggtctttctcttgttctgt  9018

exon11.USP18.human                                                          ------------------------------------------------------------  0
LOC102725072.(18846286-18861064new.ref                                      ------------------------------------------------------------  14779
LOC102725072.start-FAM230F.start.18846286-18865042.NEW.ref                  aaaatgtgggcatggatagatataaagtgcctggtgtccatgcttttgtgaaatcccttc  15079
chimp.100000-160000.Pan.troglodytes.isolate.Yerkes.chimp.pedigree.#C0471    aaaatgtgggcatggatagatataaagtgcctggtgtccatgcttttgtgaaatcccttc  18531
BCRP2.ref.rev.compl                                                         aaaatgtgggcatggatagatataaagtgcctggtgtccatgcttttgtgaaatcccttc  9078

exon11.USP18.human                                                          ------------------------------------------------------------  0
LOC102725072.(18846286-18861064new.ref                                      ------------------------------------------------------------  14779
LOC102725072.start-FAM230F.start.18846286-18865042.NEW.ref                  ctcttccatgtgaatgggacctgtgactttcttctaacccagagaacacagcaaaaatga  15139
chimp.100000-160000.Pan.troglodytes.isolate.Yerkes.chimp.pedigree.#C0471    ctcttccatgtgaatgggacctgtgactttcttctaacccagagaacacagcaaaaatga  18591
BCRP2.ref.rev.compl                                                         ctcttccatgtgaatgggacctgtgactttcttctaacccagagaacacagcaaaaatga  9138

exon11.USP18.human                                                          ------------------------------------------------------------  0
LOC102725072.(18846286-18861064new.ref                                      ------------------------------------------------------------  14779
LOC102725072.start-FAM230F.start.18846286-18865042.NEW.ref                  tgtgatttatctgagtccattgatgacattgattacgacttcccttcaccacattattta  15199
chimp.100000-160000.Pan.troglodytes.isolate.Yerkes.chimp.pedigree.#C0471    tgtgatttatctgagtccattgatgacattgattacgacttcccttcaccacattattta  18651
BCRP2.ref.rev.compl                                                         tgtgatttatctgagtccattgatgacattgattacgacttcccttcaccacattattta  9198

exon11.USP18.human                                                          ------------------------------------------------------------  0
LOC102725072.(18846286-18861064new.ref                                      ------------------------------------------------------------  14779
LOC102725072.start-FAM230F.start.18846286-18865042.NEW.ref                  ggactgcgtcgtaggagactgggacacatatccactttgctggcttgatgaagtaaactg  15259
chimp.100000-160000.Pan.troglodytes.isolate.Yerkes.chimp.pedigree.#C0471    ggactgcgtcgtaggagactgggacacatatccactttgctggcttgatgaagtaaactg  18711
BCRP2.ref.rev.compl                                                         ggactgcgtcgtaggagactgggacacatatccactttgctggcttgatgaagtaaactg  9258

exon11.USP18.human                                                          ------------------------------------------------------------  0
LOC102725072.(18846286-18861064new.ref                                      ------------------------------------------------------------  14779
LOC102725072.start-FAM230F.start.18846286-18865042.NEW.ref                  ctaagttgaggaagcccacatggcaaggaactgtgggcagccttcagccaacaggcagca  15319
chimp.100000-160000.Pan.troglodytes.isolate.Yerkes.chimp.pedigree.#C0471    ctaagttgaggaagcccacatggcaaggaattgtgggcagccttcagccaacaggcagca  18771
BCRP2.ref.rev.compl                                                         ctaagttgaggaagcccacatggcaaggaactgtgggcagccttcagccaacaggcagca  9318

exon11.USP18.human                                                          ------------------------------------------------------------  0
LOC102725072.(18846286-18861064new.ref                                      ------------------------------------------------------------  14779
LOC102725072.start-FAM230F.start.18846286-18865042.NEW.ref                  aaaagctgagctcctcggagctacagcctcaaggaagttacttctgctaacaacctgaaa  15379
chimp.100000-160000.Pan.troglodytes.isolate.Yerkes.chimp.pedigree.#C0471    aaaagctgagctcctcggagctacagcctcaaggaagttacttctgctaacaacctgaaa  18831
BCRP2.ref.rev.compl                                                         aaaagctgagctccttggagctacagcctcaaggaagttacttctgctaacaacctgaaa  9378

exon11.USP18.human                                                          ------------------------------------------------------------  0
LOC102725072.(18846286-18861064new.ref                                      ------------------------------------------------------------  14779
LOC102725072.start-FAM230F.start.18846286-18865042.NEW.ref                  aagcttggaagctagtttctctctagtggaatttttagggaagagcatggcccaggcaag  15439
chimp.100000-160000.Pan.troglodytes.isolate.Yerkes.chimp.pedigree.#C0471    aagcttggaagctagtttctctctagtggaatttttagggaagagcatggcccaggcaag  18891
BCRP2.ref.rev.compl                                                         aagcttggaagctagtttctctctagtggaatttttagggaagagcatggcccaggcaag  9438

exon11.USP18.human                                                          ------------------------------------------------------------  0
LOC102725072.(18846286-18861064new.ref                                      ------------------------------------------------------------  14779
LOC102725072.start-FAM230F.start.18846286-18865042.NEW.ref                  ataaataatgtaagtggaaaaacctgtcaacaataaaggttttaaaggaaaaaacaaaaa  15499
chimp.100000-160000.Pan.troglodytes.isolate.Yerkes.chimp.pedigree.#C0471    ataaataatgtaagtggaaaaacctgtcaacaataaaggttttaaaggaaa----aaaac  18947
BCRP2.ref.rev.compl                                                         ataaataatgtaagtggaaaaacctgtcaacaataaaggttttaaaggaaaaaacaaaaa  9498

exon11.USP18.human                                                          ------------------------------------------------------------  0
LOC102725072.(18846286-18861064new.ref                                      ------------------------------------------------------------  14779
LOC102725072.start-FAM230F.start.18846286-18865042.NEW.ref                  acatgtaatttagaaagtaactgccaggaaaaaaaaaaaaaagagactgggc-cagtggct  15558
chimp.100000-160000.Pan.troglodytes.isolate.Yerkes.chimp.pedigree.#C0471    acatgtaatttagaaagtaactgccagg--aaaaaaaaaaaagagactgggcgcagtggct  19005
BCRP2.ref.rev.compl                                                         acatgtaatttagaaagtaactgccagg--aaaaaaaaaaaagagactgggccagtggct  9556

exon11.USP18.human                                                          ------------------------------------------------------------  0
LOC102725072.(18846286-18861064new.ref                                      ------------------------------------------------------------  14779
LOC102725072.start-FAM230F.start.18846286-18865042.NEW.ref                  tactacacctgtaatcccagcacttggggaggctgaggcgggtgaatcacaaggtcagga  15618
chimp.100000-160000.Pan.troglodytes.isolate.Yerkes.chimp.pedigree.#C0471    tactacgcctgtaatcccagaacttggggaggctgaggcgggtgaatcacaaggtcagga  19065
BCRP2.ref.rev.compl                                                         tactacacctgtaatcccagcacttggggaggctgaggcgggtgaatcacaaggtcagga  9616

exon11.USP18.human                                                          ------------------------------------------------------------  0
LOC102725072.(18846286-18861064new.ref                                      ------------------------------------------------------------  14779
LOC102725072.start-FAM230F.start.18846286-18865042.NEW.ref                  gatcgagaccatcctggctaacacggtgaaaccccgtctctactaaaaatac-aaaaaaa  15677
chimp.100000-160000.Pan.troglodytes.isolate.Yerkes.chimp.pedigree.#C0471    gatcgagaccatcctggctaacacggtgaaaccccgtctctactaaaaatac--aaaaaa  19123
BCRP2.ref.rev.compl                                                         gatcgagaccatcctggctaacacggtgaaaccccgtctctactaaaaatacaaaaaaaa  9676

exon11.USP18.human                                                          ------------------------------------------------------------  0
```

```
LOC102725072.(18846286-18861064new.ref                                     ------------------------------------------------------------  14779
LOC102725072.start-FAM230F.start.18846286-18865042.NEW.ref                 aaaaaaaaattagccgggcgtggtggtgggcgcctgtggtcccagctactggggaggttg  15737
chimp.100000-160000.Pan.troglodytes.isolate.Yerkes.chimp.pedigree.#C0471   aaaaaaaaattagccgggcgtggtggtgggcgcctgtggtcccagctactggggaggttg  19183
BCRP2.ref.rev.compl                                                        aaaaaaaaattagccgggcgtggtggtgggcgcctgtggtcccagctactggggaggttg  9736


exon11.USP18.human                                                         ------------------------------------------------------------  0
LOC102725072.(18846286-18861064new.ref                                     ------------------------------------------------------------  14779
LOC102725072.start-FAM230F.start.18846286-18865042.NEW.ref                 agacaggagaatggcgtgaaccccgggacgtggagcttgcagtgagccgagatcatgcca  15797
chimp.100000-160000.Pan.troglodytes.isolate.Yerkes.chimp.pedigree.#C0471   agacaggagaatggcgtgaaccccgggatgtggagcttgcagtgagccgagatcatgcca  19243
BCRP2.ref.rev.compl                                                        agacaggagaatggcgtgaaccccgggacgtggagcttgcagtgagccgagatcatgcca  9796


exon11.USP18.human                                                         ------------------------------------------------------------  0
LOC102725072.(18846286-18861064new.ref                                     ------------------------------------------------------------  14779
LOC102725072.start-FAM230F.start.18846286-18865042.NEW.ref                 ctgcactccagcctgggcaacagagcgagactccatctcaaaataaataaataaataaaa  15857
chimp.100000-160000.Pan.troglodytes.isolate.Yerkes.chimp.pedigree.#C0471   ctgcgctccagcctgggcaacagagcgagactccatctcaaaataaataaataaataaaa  19303
BCRP2.ref.rev.compl                                                        ctgcactccagcctgggcaacagagcgagactccatctcaaaataaataaataaataaaa  9856


exon11.USP18.human                                                         ------------------------------------------------------------  0
LOC102725072.(18846286-18861064new.ref                                     ------------------------------------------------------------  14779
LOC102725072.start-FAM230F.start.18846286-18865042.NEW.ref                 tataaggtctcaggaacgtaaagattgacatttactcccaaactattaatatatgtccac  15917
chimp.100000-160000.Pan.troglodytes.isolate.Yerkes.chimp.pedigree.#C0471   tataaggtctcaggaacgtaaagattgacatttactcccaaactattaatatatgtccac  19363
BCRP2.ref.rev.compl                                                        tataaggtctcaggaacgtaaagattgacatttactcccaaactattaatatatgtccac  9916


exon11.USP18.human                                                         ------------------------------------------------------------  0
LOC102725072.(18846286-18861064new.ref                                     ------------------------------------------------------------  14779
LOC102725072.start-FAM230F.start.18846286-18865042.NEW.ref                 ccacctttcttgtagcaaaatcttaacttgacgtttgtttcaatagttattaaatttaat  15977
chimp.100000-160000.Pan.troglodytes.isolate.Yerkes.chimp.pedigree.#C0471   ccacctttcttgtagcaaaatcttaacttgacgtttgtttcaatagttattaaatttaat  19423
BCRP2.ref.rev.compl                                                        ccacctttcttgtagcaaaatcttaacttgacgtttgtttcaatagttattaaatttaat  9976


exon11.USP18.human                                                         ------------------------------------------------------------  0
LOC102725072.(18846286-18861064new.ref                                     ------------------------------------------------------------  14779
LOC102725072.start-FAM230F.start.18846286-18865042.NEW.ref                 tataatgtcctagcccaaaatacagtagaggtaaacatccaaggtactggcttgaggcca  16037
chimp.100000-160000.Pan.troglodytes.isolate.Yerkes.chimp.pedigree.#C0471   tataatgtcctagcccaaaatacagtagaggtaaacatccaagctactggcttgaggcca  19483
BCRP2.ref.rev.compl                                                        tataatgtcctagcccaaaatacagtagaggtaaacatccaaggtactggcttgaggcca  10036


exon11.USP18.human                                                         ------------------------------------------------------------  0
LOC102725072.(18846286-18861064new.ref                                     ------------------------------------------------------------  14779
LOC102725072.start-FAM230F.start.18846286-18865042.NEW.ref                 ctggccctgtatctataaaggagagggagaccatcaggggagggagggtaagaaagggag  16097
chimp.100000-160000.Pan.troglodytes.isolate.Yerkes.chimp.pedigree.#C0471   ctggccctgtatctataaaggagagggagaccatcaggggagggagggtaagaaagggag  19543
BCRP2.ref.rev.compl                                                        ctggccctgtatctataaaggagagggagaccatcaggggagggagggtaagaaagggag  10096


exon11.USP18.human                                                         ------------------------------------------------------------  0
LOC102725072.(18846286-18861064new.ref                                     ------------------------------------------------------------  14779
LOC102725072.start-FAM230F.start.18846286-18865042.NEW.ref                 gaagagggcaaacaagttatcaaaaaacaacagtaggcagggcactatggctcatgcttg  16157
chimp.100000-160000.Pan.troglodytes.isolate.Yerkes.chimp.pedigree.#C0471   gaagagggcaaacaagttatcaaaaaacaacagtaggcagggcactatggctcatgctta  19603
BCRP2.ref.rev.compl                                                        gaagagggcagacaagttatcaaaaaacaacagtaggcagggcactatggctcatgcttg  10156


exon11.USP18.human                                                         ------------------------------------------------------------  0
LOC102725072.(18846286-18861064new.ref                                     ------------------------------------------------------------  14779
LOC102725072.start-FAM230F.start.18846286-18865042.NEW.ref                 taatcctagcattttgggaagccaaggtgggcaaattgcttgcactcaggagttcaagac  16217
chimp.100000-160000.Pan.troglodytes.isolate.Yerkes.chimp.pedigree.#C0471   taatcctagcattttgggaagccaaggtgggcaaattgcttgcactcaggagttcaagac  19663
BCRP2.ref.rev.compl                                                        taatcctagcattttgggaagccaaggtgggcaaattgcttgcactcaggagttcaagac  10216


exon11.USP18.human                                                         ------------------------------------------------------------  0
LOC102725072.(18846286-18861064new.ref                                     ------------------------------------------------------------  14779
LOC102725072.start-FAM230F.start.18846286-18865042.NEW.ref                 cagcctggacaacatggcaaaaccccatctctacaaaaaatcggccaggcctggtggtgt  16277
chimp.100000-160000.Pan.troglodytes.isolate.Yerkes.chimp.pedigree.#C0471   cagcctggacaacatggcaaaaccccatctctacaaaaaatcggccaggcctggtggtgt  19723
BCRP2.ref.rev.compl                                                        cagcctggacaacatggcaaaaccccatctctacaaaaaatcggccaggcctggtggtgt  10276


exon11.USP18.human                                                         ------------------------------------------------------------  0
LOC102725072.(18846286-18861064new.ref                                     ------------------------------------------------------------  14779
LOC102725072.start-FAM230F.start.18846286-18865042.NEW.ref                 acgcttgtaattccagctacttgggaggctgaggtaggaggatcacttgagcctggaagg  16337
chimp.100000-160000.Pan.troglodytes.isolate.Yerkes.chimp.pedigree.#C0471   acgcttgtaattccagctacttgggaggctgaggtaggaggatcacttgagcctggaagg  19783
BCRP2.ref.rev.compl                                                        atgcttgtaattccagctacttgggaggctgaggtaggaggatcacttgagcctggaagg  10336


exon11.USP18.human                                                         ------------------------------------------------------------  0
LOC102725072.(18846286-18861064new.ref                                     ------------------------------------------------------------  14779
LOC102725072.start-FAM230F.start.18846286-18865042.NEW.ref                 cagaggttgcagtgagccgacatcatgccactgcacttcagcctgagtgacagagtaaga  16397
chimp.100000-160000.Pan.troglodytes.isolate.Yerkes.chimp.pedigree.#C0471   cagaggttgcagtgagccgacatgatgccactgcacttcagcctgagtgacagagtaaga  19843
BCRP2.ref.rev.compl                                                        cagaggttgcagtgagccgacatcatgccactgcacttcagcctgagtgacagagtaaga  10396


exon11.USP18.human                                                         ------------------------------------------------------------  0
LOC102725072.(18846286-18861064new.ref                                     ------------------------------------------------------------  14779
LOC102725072.start-FAM230F.start.18846286-18865042.NEW.ref                 ccctgtctcaaaaacaaacacatcagttattcatatttcagagtaaggacaaaacatttt  16457
chimp.100000-160000.Pan.troglodytes.isolate.Yerkes.chimp.pedigree.#C0471   ccctgtctcaaaaacaaacacatcagttattcatatttcagagtaaggacaaaacatttt  19903
BCRP2.ref.rev.compl                                                        ccctgtctcaaaaacaaacacatcagttattcatatttcagagtaaggacaaaacatttt  10456


exon11.USP18.human                                                         ------------------------------------------------------------  0
LOC102725072.(18846286-18861064new.ref                                     ------------------------------------------------------------  14779
LOC102725072.start-FAM230F.start.18846286-18865042.NEW.ref                 taagtagctggcaaaggacatcactatatttcagagtaaaacaaataggaaatgcttatc  16517
chimp.100000-160000.Pan.troglodytes.isolate.Yerkes.chimp.pedigree.#C0471   taagtagctggcaaaggacatcactatatttcagagtaaaacaaataggaaatgcttatc  19963
BCRP2.ref.rev.compl                                                        taagtagctggcaaaggacatccctatatttcagagtaaaacaaataggcaatgcttatc  10516


exon11.USP18.human                                                         ------------------------------------------------------------  0
LOC102725072.(18846286-18861064new.ref                                     ------------------------------------------------------------  14779
LOC102725072.start-FAM230F.start.18846286-18865042.NEW.ref                 atttgacatattttaaacattgtatctgaaaagtgaacaaagaagtgaatgtgcttatg  16577
chimp.100000-160000.Pan.troglodytes.isolate.Yerkes.chimp.pedigree.#C0471   atttgacatattttaaacattgtatctgaaaagtgaacaaagaaataaatgtgcttatg  20023
BCRP2.ref.rev.compl                                                        atttgacatattttaaacattgtatctgaaaagtgaacaaagaaatgaatgtgcttatg  10576


exon11.USP18.human                                                         ------------------------------------------------------------  0
LOC102725072.(18846286-18861064new.ref                                     ------------------------------------------------------------  14779
LOC102725072.start-FAM230F.start.18846286-18865042.NEW.ref                 attaaattgactttgttactttgtaaacttgtagctttagacctgtctcttagcatcacc  16637
chimp.100000-160000.Pan.troglodytes.isolate.Yerkes.chimp.pedigree.#C0471   attaaattgactttgttactttgtaaacttgtagctttagacctgtctcttagcatcacc  20083
BCRP2.ref.rev.compl                                                        attaaattgactttgttactttgtaaacttgtagctttagacctgtctcttagcatcacc  10636


exon11.USP18.human                                                         ------------------------------------------------------------  0
LOC102725072.(18846286-18861064new.ref                                     ------------------------------------------------------------  14779
LOC102725072.start-FAM230F.start.18846286-18865042.NEW.ref                 aagccttgatcttttcatctataaaatgggcatggtaatgccagccttgctatgtttata  16697
chimp.100000-160000.Pan.troglodytes.isolate.Yerkes.chimp.pedigree.#C0471   aagccttgatcttttcatctataaaatgggcatggtaatgccagccttgctatgtttata  20143
BCRP2.ref.rev.compl                                                        aagccttgatcttttcatctataaaatgggcatggtaatgccagccttgctatgtttata  10696


exon11.USP18.human                                                         ------------------------------------------------------------  0
LOC102725072.(18846286-18861064new.ref                                     ------------------------------------------------------------  14779
LOC102725072.start-FAM230F.start.18846286-18865042.NEW.ref                 ggtcacttaggaatgaggtatgtatggtgttgaccatggtttctggcaagtggcatatat  16757
chimp.100000-160000.Pan.troglodytes.isolate.Yerkes.chimp.pedigree.#C0471   ggtcacttaggaatgaggtatgtatggtgttgaccatggtttctggcaagtggcatatat  20203
BCRP2.ref.rev.compl                                                        ggtcacttaggaatgaggtatgtatggtgttgaccatggtttctggcaagtggcatatat  10756


exon11.USP18.human                                                         ------------------------------------------------------------  0
LOC102725072.(18846286-18861064new.ref                                     ------------------------------------------------------------  14779
LOC102725072.start-FAM230F.start.18846286-18865042.NEW.ref                 tcattataccatagctcttttcagaaagctaagtcaccacgtacatgttaatgcaacctg  16817
chimp.100000-160000.Pan.troglodytes.isolate.Yerkes.chimp.pedigree.#C0471   tcattataccatagctcttttcagaaagctaagtcaccacgtacatgttaatgcaacctg  20263
BCRP2.ref.rev.compl                                                        tcattataccatagctcttttcagaaagctaagtcaccacgtacatgtcaatgcaacctg  10816


exon11.USP18.human                                                         ------------------------------------------------------------  0
LOC102725072.(18846286-18861064new.ref                                     ------------------------------------------------------------  14779
LOC102725072.start-FAM230F.start.18846286-18865042.NEW.ref                 ctgaaaataggcatggaaaaactagaaaatctagaaaaattagaaaatcactgaagagga  16877
chimp.100000-160000.Pan.troglodytes.isolate.Yerkes.chimp.pedigree.#C0471   ctgaaaataggcatggaaaaactagaaaatctagaaaaactagaaaatcactgaagagga  20323
BCRP2.ref.rev.compl                                                        ctgaaaataggcatggaaaaactagaaaatctagaaaaattagaaaatcactgaagagga  10876
```

end of BCRP2 in LOC102725072-FAM230F but continuation in chimp sequence

```
exon11.USP18.human                                                         ------------------------------------------------------------  0
LOC102725072.(18846286-18861064new.ref                                     ------------------------------------------------------------  14779
LOC102725072.start-FAM230F.start.18846286-18865042.NEW.ref                 ttttctttaaaaaaatacatactttaa---------------------------------  16904
chimp.100000-160000.Pan.troglodytes.isolate.Yerkes.chimp.pedigree.#C0471   ttttctttaaaaaaatacatacttcagtgtttgagtcatgaagtccttgcccgtgcctat  20383
BCRP2.ref.rev.compl                                                        ttttctttaaaaaaatacatactttagtgtttgagtcatgaagtccttgcccgtgcctat  10936
```

```
exon11.USP18.human                                                         ------------------------------------------------------------  0
LOC102725072.(18846286-18861064new.ref                                     ------------------------------------------------------------  14779
LOC102725072.start-FAM230F.start.18846286-18865042.NEW.ref                 ------------------------------------------------------------  16904
chimp.100000-160000.Pan.troglodytes.isolate.Yerkes.chimp.pedigree.#C0471   gtcctaagtggtattgcctgggttttcttctagggtttttatggttttaggtctaacatt  20443
BCRP2.ref.rev.compl                                                        gtcctaagtggtattgcctgggttttcttctagggtttttatggttttaggtctaacatt  10996

exon11.USP18.human                                                         ------------------------------------------------------------  0
LOC102725072.(18846286-18861064new.ref                                     ------------------------------------------------------------  14779
LOC102725072.start-FAM230F.start.18846286-18865042.NEW.ref                 ------------------------------------------------------------  16904
chimp.100000-160000.Pan.troglodytes.isolate.Yerkes.chimp.pedigree.#C0471   taagtctttaatccatcttgaattaatttttgtataaggtgtaaggaagggatccagttt  20503
BCRP2.ref.rev.compl                                                        taagtctttaatccatcttgaattaatttttgtataaggtgtaaggaagggatccagttt  11056

exon11.USP18.human                                                         ------------------------------------------------------------  0
LOC102725072.(18846286-18861064new.ref                                     ------------------------------------------------------------  14779
LOC102725072.start-FAM230F.start.18846286-18865042.NEW.ref                 ------------------------------------------------------------  16904
chimp.100000-160000.Pan.troglodytes.isolate.Yerkes.chimp.pedigree.#C0471   cagctttctaaatatggctagccagtttagttcatgtcctttgtagcgacatggatgaag  20563
BCRP2.ref.rev.compl                                                        cagctttctaaatatggctagccagtttagttcatgtcctttgtagcgacagggatgaag  11116

exon11.USP18.human                                                         ------------------------------------------------------------  0
LOC102725072.(18846286-18861064new.ref                                     ------------------------------------------------------------  14779
LOC102725072.start-FAM230F.start.18846286-18865042.NEW.ref                 ------------------------------------------------------------  16904
chimp.100000-160000.Pan.troglodytes.isolate.Yerkes.chimp.pedigree.#C0471   ctggaaaccatcattctgagcaaactatcgcaaggacagaaaaccaaacagcgcatgttc  20623
BCRP2.ref.rev.compl                                                        ctggaaaccatcattctgagcaaactatcgcaaggacagaaaaccaaacagcgcatgttc  11176

exon11.USP18.human                                                         ------------------------------------------------------------  0
LOC102725072.(18846286-18861064new.ref                                     ------------------------------------------------------------  14779
LOC102725072.start-FAM230F.start.18846286-18865042.NEW.ref                 ------------------------------------------------------------  16904
chimp.100000-160000.Pan.troglodytes.isolate.Yerkes.chimp.pedigree.#C0471   tcactcataggtggtaattaaacaataagaacacgtggacacagggtggggaacatcaca  20683
BCRP2.ref.rev.compl                                                        tcactcataggtgggaattaaacaataagaacacgtggacacagggtggggaacatcaca  11236

exon11.USP18.human                                                         ------------------------------------------------------------  0
LOC102725072.(18846286-18861064new.ref                                     ------------------------------------------------------------  14779
LOC102725072.start-FAM230F.start.18846286-18865042.NEW.ref                 ------------------------------------------------------------  16904
chimp.100000-160000.Pan.troglodytes.isolate.Yerkes.chimp.pedigree.#C0471   caccggggcctgtcgtggggtggggcgataggggagggatagcattagaataaataccta  20743
BCRP2.ref.rev.compl                                                        caccggggcctgtcgtggggtgggggataggggagggatagcattagaataaataccta  11296

exon11.USP18.human                                                         ------------------------------------------------------------  0
LOC102725072.(18846286-18861064new.ref                                     ------------------------------------------------------------  14779
LOC102725072.start-FAM230F.start.18846286-18865042.NEW.ref                 ------------------------------------------------------------  16904
chimp.100000-160000.Pan.troglodytes.isolate.Yerkes.chimp.pedigree.#C0471   atgtaaatgatgagttaatgggtgcagcaaaccaacacagcacatgtatacatatgtaac  20803
BCRP2.ref.rev.compl                                                        atgtaaatgatgagttaatgggtgcagcaaaccaacacagcacatgtatacatatgtaac  11356

exon11.USP18.human                                                         ------------------------------------------------------------  0
LOC102725072.(18846286-18861064new.ref                                     ------------------------------------------------------------  14779
LOC102725072.start-FAM230F.start.18846286-18865042.NEW.ref                 ------------------------------------------------------------  16904
chimp.100000-160000.Pan.troglodytes.isolate.Yerkes.chimp.pedigree.#C0471   aaatctgcacgttgtgcacatgtaccctagaacttaaaaggataataatatatatataca  20863
BCRP2.ref.rev.compl                                                        aaatctgcacgttgtgcacatgtaccctaggacttaaaaggataataatatatatataca  11416

exon11.USP18.human                                                         ------------------------------------------------------------  0
LOC102725072.(18846286-18861064new.ref                                     ------------------------------------------------------------  14779
LOC102725072.start-FAM230F.start.18846286-18865042.NEW.ref                 ------------------------------------------------------------  16904
chimp.100000-160000.Pan.troglodytes.isolate.Yerkes.chimp.pedigree.#C0471   tacacacatacactttatccatgcatctgttgatagacacttaggttgttccctatcttg  20923
BCRP2.ref.rev.compl                                                        tacacacatacactttatccatgcatctgttgatagacacttaggttgttccctatcttg  11476

exon11.USP18.human                                                         ------------------------------------------------------------  0
LOC102725072.(18846286-18861064new.ref                                     ------------------------------------------------------------  14779
LOC102725072.start-FAM230F.start.18846286-18865042.NEW.ref                 ------------------------------------------------------------  16904
chimp.100000-160000.Pan.troglodytes.isolate.Yerkes.chimp.pedigree.#C0471   gctgttgtgaataatgtgccataaatatgggggtgcaggtacctctctgacatactgatt  20983
BCRP2.ref.rev.compl                                                        gctgttgtgaataatgtgccataaatatgggggtgcaggtacctctctgacatactgatt  11536

exon11.USP18.human                                                         ------------------------------------------------------------  0
LOC102725072.(18846286-18861064new.ref                                     ------------------------------------------------------------  14779
LOC102725072.start-FAM230F.start.18846286-18865042.NEW.ref                 ------------------------------------------------------------  16904
chimp.100000-160000.Pan.troglodytes.isolate.Yerkes.chimp.pedigree.#C0471   tcaatttccttggatatatagccagaagtaggattgctgggtcatgtggtaattttttttt  21043
BCRP2.ref.rev.compl                                                        tcaatttccttgg--atatagccagaagtaggattgctgggtcatgtggtaa--tttttt  11592

exon11.USP18.human                                                         ------------------------------------------------------------  0
LOC102725072.(18846286-18861064new.ref                                     ------------------------------------------------------------  14779
LOC102725072.start-FAM230F.start.18846286-18865042.NEW.ref                 ------------------------------------------------------------  16904
chimp.100000-160000.Pan.troglodytes.isolate.Yerkes.chimp.pedigree.#C0471   tttttttttttttgagacaaagtcttgctctgttgcccaggctggagtgcagtggcatga  21103
BCRP2.ref.rev.compl                                                        tttttttttttttgagacaaagtcttgctctgttgcccaggctggagtgcagtggcatga  11652

exon11.USP18.human                                                         ------------------------------------------------------------  0
LOC102725072.(18846286-18861064new.ref                                     ------------------------------------------------------------  14779
LOC102725072.start-FAM230F.start.18846286-18865042.NEW.ref                 ------------------------------------------------------------  16904
chimp.100000-160000.Pan.troglodytes.isolate.Yerkes.chimp.pedigree.#C0471   tctcggctcactgcagcctccacctcacgggctcaagcagtcctcccacctcagcctccc  21163
BCRP2.ref.rev.compl                                                        tctcggctcactgcagcctccacctcacgggctcaagcagtcctcccacctcagcctccc  11712

exon11.USP18.human                                                         ------------------------------------------------------------  0
LOC102725072.(18846286-18861064new.ref                                     ------------------------------------------------------------  14779
LOC102725072.start-FAM230F.start.18846286-18865042.NEW.ref                 ------------------------------------------------------------  16904
chimp.100000-160000.Pan.troglodytes.isolate.Yerkes.chimp.pedigree.#C0471   gaagtgctgggattacagtgtgagccactgcacctgacctcatgtggtaattgtattttt  21223
BCRP2.ref.rev.compl                                                        gaagtgctgggattacagtgtgagccactgcacctgacctcatgtggtaattgtattttt  11772

exon11.USP18.human                                                         ------------------------------------------------------------  0
LOC102725072.(18846286-18861064new.ref                                     ------------------------------------------------------------  14779
LOC102725072.start-FAM230F.start.18846286-18865042.NEW.ref                 ------------------------------------------------------------  16904
chimp.100000-160000.Pan.troglodytes.isolate.Yerkes.chimp.pedigree.#C0471   agttatttgattaattttttgtgccgtttatcataatgcctgtactgatttacattcactc  21283
BCRP2.ref.rev.compl                                                        agttatttgattaattttttgtgccgtttttcataatgcctgtactgatttacattcactc  11832

exon11.USP18.human                                                         ------------------------------------------------------------  0
LOC102725072.(18846286-18861064new.ref                                     ------------------------------------------------------------  14779
LOC102725072.start-FAM230F.start.18846286-18865042.NEW.ref                 ------------------------------------------------------------  16904
chimp.100000-160000.Pan.troglodytes.isolate.Yerkes.chimp.pedigree.#C0471   caacatgtactagggttccattttctccacatcctcttcaacacttgttacctttctttt  21343
BCRP2.ref.rev.compl                                                        caacatgtactagggttccattttctccacatcctcttcaacacttgttacctttctttt  11892

exon11.USP18.human                                                         ------------------------------------------------------------  0
LOC102725072.(18846286-18861064new.ref                                     ------------------------------------------------------------  14779
LOC102725072.start-FAM230F.start.18846286-18865042.NEW.ref                 ------------------------------------------------------------  16904
chimp.100000-160000.Pan.troglodytes.isolate.Yerkes.chimp.pedigree.#C0471   tttaatattaatcattctaacagatgtaaagtgatattgttgtggttttaatttgcattt  21403
BCRP2.ref.rev.compl                                                        tttaatattaatcattctaacagatataaagtgatattgttgtggttttaatttgcattt  11952

exon11.USP18.human                                                         ------------------------------------------------------------  0
LOC102725072.(18846286-18861064new.ref                                     ------------------------------------------------------------  14779
LOC102725072.start-FAM230F.start.18846286-18865042.NEW.ref                 ------------------------------------------------------------  16904
chimp.100000-160000.Pan.troglodytes.isolate.Yerkes.chimp.pedigree.#C0471   ccctaatgattagtgctgctgggcatttttttaaaagaaggaaattatgtcatttgcaac  21463
BCRP2.ref.rev.compl                                                        ccctaatgattagtgctgctgggcatttttttaaaagaaggaaattatgtcatttgcaac  12012

exon11.USP18.human                                                         ------------------------------------------------------------  0
LOC102725072.(18846286-18861064new.ref                                     ------------------------------------------------------------  14779
LOC102725072.start-FAM230F.start.18846286-18865042.NEW.ref                 ------------------------------------------------------------  16904
chimp.100000-160000.Pan.troglodytes.isolate.Yerkes.chimp.pedigree.#C0471   aaaatgaataaacctggagaacattacactaagtgaaagaagccagacaggacagataag  21523
BCRP2.ref.rev.compl                                                        aaaatgaataaacctggagaacattacactaagtgaaagaagccagacaggatagataag  12072

exon11.USP18.human                                                         ------------------------------------------------------------  0
LOC102725072.(18846286-18861064new.ref                                     ------------------------------------------------------------  14779
LOC102725072.start-FAM230F.start.18846286-18865042.NEW.ref                 ------------------------------------------------------------  16904
chimp.100000-160000.Pan.troglodytes.isolate.Yerkes.chimp.pedigree.#C0471   tacatgatctcatgtatatgtggaatctaaaaaagccaaatttatagaaataacaggtag  21583
BCRP2.ref.rev.compl                                                        tacatgatctcatgtatatgtggaatctaaaaaagccaaatttatagaaataacaggtag  12132

exon11.USP18.human                                                         ------------------------------------------------------------  0
LOC102725072.(18846286-18861064new.ref                                     ------------------------------------------------------------  14779
LOC102725072.start-FAM230F.start.18846286-18865042.NEW.ref                 ------------------------------------------------------------  16904
```

```
chimp.100000-160000.Pan.troglodytes.isolate.Yerkes.chimp.pedigree.#C0471      aaaa-gggttatgagaggctggggtgtgttgggggg tacagcagacagggagatgctgtt  21642
BCRP2.ref.rev.compl                                                           aaaaggggttatgagaggctggggtgtgttggggggtacagcagacagggagatgctgtt  12192

exon11.USP18.human                                                            ------------------------------------------------------------  0
LOC102725072.(18846286-18861064new.ref                                        ------------------------------------------------------------  14779
LOC102725072.start-FAM230F.start.18846286-18865042.NEW.ref                    ------------------------------------------------------------  16904
chimp.100000-160000.Pan.troglodytes.isolate.Yerkes.chimp.pedigree.#C0471      caaagggtacaaagttttcattaggaggaataaagttttgagatctattgcacagtatgg  21702
BCRP2.ref.rev.compl                                                           caaagggtacaaagttttcattaggaggaataaagttttgagatctattgcacagtatgg  12252

exon11.USP18.human                                                            ------------------------------------------------------------  0
LOC102725072.(18846286-18861064new.ref                                        ------------------------------------------------------------  14779
LOC102725072.start-FAM230F.start.18846286-18865042.NEW.ref                    ------------------------------------------------------------  16904
chimp.100000-160000.Pan.troglodytes.isolate.Yerkes.chimp.pedigree.#C0471      tggctatagtaaataataatctatcatatatttcaaaattgctaaaagtaaatttcaaat  21762
BCRP2.ref.rev.compl                                                           tggctatagtaaataataatctatcatatatttcaaaattgctaagagtaaatttcaaat  12312

exon11.USP18.human                                                            ------------------------------------------------------------  0
LOC102725072.(18846286-18861064new.ref                                        ------------------------------------------------------------  14779
LOC102725072.start-FAM230F.start.18846286-18865042.NEW.ref                    ------------------------------------------------------------  16904
chimp.100000-160000.Pan.troglodytes.isolate.Yerkes.chimp.pedigree.#C0471      gtcccaccacaaaggtgagctggatgatggatatgttaattagctttatttaatcattcc  21822
BCRP2.ref.rev.compl                                                           gtcccaccacaaaggtgagctggatgatggatatgttaattagctttatttaatcattcc  12372

exon11.USP18.human                                                            ------------------------------------------------------------  0
LOC102725072.(18846286-18861064new.ref                                        ------------------------------------------------------------  14779
LOC102725072.start-FAM230F.start.18846286-18865042.NEW.ref                    ------------------------------------------------------------  16904
chimp.100000-160000.Pan.troglodytes.isolate.Yerkes.chimp.pedigree.#C0471      acattgtgtacacatatcaaaacatcacattgtactcaagacatctatacaattgacttg  21882
BCRP2.ref.rev.compl                                                           acattgtgtacacatatcaaaacatcacattgtactcaagacatctatacaattgacttg  12432

exon11.USP18.human                                                            ------------------------------------------------------------  0
LOC102725072.(18846286-18861064new.ref                                        ------------------------------------------------------------  14779
LOC102725072.start-FAM230F.start.18846286-18865042.NEW.ref                    ------------------------------------------------------------  16904
chimp.100000-160000.Pan.troglodytes.isolate.Yerkes.chimp.pedigree.#C0471      tcaaccaaaataataaaaataaagtctatgaaatgtaaaatagttttgttacacagttta  21942
BCRP2.ref.rev.compl                                                           tcaaccaaaataataaaaataaagtctataaaatgtaaaatagttttgttacacagttta  12492

exon11.USP18.human                                                            ------------------------------------------------------------  0
LOC102725072.(18846286-18861064new.ref                                        ------------------------------------------------------------  14779
LOC102725072.start-FAM230F.start.18846286-18865042.NEW.ref                    ------------------------------------------------------------  16904
chimp.100000-160000.Pan.troglodytes.isolate.Yerkes.chimp.pedigree.#C0471      gtgttctttaaaccatattggctgagcttcctctttgtcttgcccctttctcaagtctgg  22002
BCRP2.ref.rev.compl                                                           gtgttctttaaaccatattggctgagcttcctctttgccttgcccctttctcaagtctgg  12552

exon11.USP18.human                                                            ------------------------------------------------------------  0
LOC102725072.(18846286-18861064new.ref                                        ------------------------------------------------------------  14779
LOC102725072.start-FAM230F.start.18846286-18865042.NEW.ref                    ------------------------------------------------------------  16904
chimp.100000-160000.Pan.troglodytes.isolate.Yerkes.chimp.pedigree.#C0471      gtgctgaatggtaaatatagaatcagttaatgtttactgaggatctagtatgtgcctcac  22062
BCRP2.ref.rev.compl                                                           gtgctgaatggtaaatatagaatcagttaatgtttactgaggatctagtatgtgcctcac  12612

exon11.USP18.human                                                            ------------------------------------------------------------  0
LOC102725072.(18846286-18861064new.ref                                        ------------------------------------------------------------  14779
LOC102725072.start-FAM230F.start.18846286-18865042.NEW.ref                    ------------------------------------------------------------  16904
chimp.100000-160000.Pan.troglodytes.isolate.Yerkes.chimp.pedigree.#C0471      actgacctaaacattttataactacttgtttcaataccctggtgaggtggtttttatgat  22122
BCRP2.ref.rev.compl                                                           actgacctaaacattttataactaatttttcaataccctggtgaggtggtttttatgat  12672

exon11.USP18.human                                                            ------------------------------------------------------------  0
LOC102725072.(18846286-18861064new.ref                                        ------------------------------------------------------------  14779
LOC102725072.start-FAM230F.start.18846286-18865042.NEW.ref                    ------------------------------------------------------------  16904
chimp.100000-160000.Pan.troglodytes.isolate.Yerkes.chimp.pedigree.#C0471      tatcccatttggcctgagctagatgatctcagagaggttaagaaatcttcaaatcttcac  22182
BCRP2.ref.rev.compl                                                           tatcccatttggcttgagctagatgatctcagagaggttaagaaatcttcaaatcttcac  12732

exon11.USP18.human                                                            ------------------------------------------------------------  0
LOC102725072.(18846286-18861064new.ref                                        ------------------------------------------------------------  14779
LOC102725072.start-FAM230F.start.18846286-18865042.NEW.ref                    ------------------------------------------------------------  16904
chimp.100000-160000.Pan.troglodytes.isolate.Yerkes.chimp.pedigree.#C0471      aaggctgcagaactactaaaaggcaaggcctgtataattacttgttgaaggaatgaatga  22242
BCRP2.ref.rev.compl                                                           aaggctgcagaactactaaaaggcaaggcctgtataattacttgttgaaggaatgaatga  12792

exon11.USP18.human                                                            ------------------------------------------------------------  0
LOC102725072.(18846286-18861064new.ref                                        ------------------------------------------------------------  14779
LOC102725072.start-FAM230F.start.18846286-18865042.NEW.ref                    ------------------------------------------------------------  16904
chimp.100000-160000.Pan.troglodytes.isolate.Yerkes.chimp.pedigree.#C0471      tatgagactgtttattgttttaaattatggaacaatttatactttgtaaaatgcacaagt  22302
BCRP2.ref.rev.compl                                                           tatgagactgtttattgttttaaattatggaacaatttatactttgtaaaatgcagaagt  12852

exon11.USP18.human                                                            ------------------------------------------------------------  0
LOC102725072.(18846286-18861064new.ref                                        ------------------------------------------------------------  14779
LOC102725072.start-FAM230F.start.18846286-18865042.NEW.ref                    ------------------------------------------------------------  16904
chimp.100000-160000.Pan.troglodytes.isolate.Yerkes.chimp.pedigree.#C0471      tttaaatgttcacttctgtgagctgtgacagttatagaccctcatgttactatcccagtc  22362
BCRP2.ref.rev.compl                                                           tttaaatgttcacttctgtgagctttgacagttatagaccctcatgttactatcccagtc  12912

exon11.USP18.human                                                            ------------------------------------------------------------  0
LOC102725072.(18846286-18861064new.ref                                        ------------------------------------------------------------  14779
LOC102725072.start-FAM230F.start.18846286-18865042.NEW.ref                    ------------------------------------------------------------  16904
chimp.100000-160000.Pan.troglodytes.isolate.Yerkes.chimp.pedigree.#C0471      aaaatatagaacatttccagaaaatcctctcacatctctttccagtcagtaccttcatgg  22422
BCRP2.ref.rev.compl                                                           aaaatatagaacatttccagaaaatcctctcacatctctttccagtcagtaccttcatgg  12972

exon11.USP18.human                                                            ------------------------------------------------------------  0
LOC102725072.(18846286-18861064new.ref                                        ------------------------------------------------------------  14779
LOC102725072.start-FAM230F.start.18846286-18865042.NEW.ref                    ------------------------------------------------------------  16904
chimp.100000-160000.Pan.troglodytes.isolate.Yerkes.chimp.pedigree.#C0471      gtcgaaaacaactaccttctgagctctgtcaccaaagataaatttttcttatggctgcat  22482
BCRP2.ref.rev.compl                                                           gtcaaaaacaactaccttctgagctctgtcaccaaagataaatttttcttatggctgcat  13032

exon11.USP18.human                                                            ------------------------------------------------------------  0
LOC102725072.(18846286-18861064new.ref                                        ------------------------------------------------------------  14779
LOC102725072.start-FAM230F.start.18846286-18865042.NEW.ref                    ------------------------------------------------------------  16904
chimp.100000-160000.Pan.troglodytes.isolate.Yerkes.chimp.pedigree.#C0471      ttcaaataaatggaatcacacatccacacttttcaatgtctggcttccttctctaaatac  22542
BCRP2.ref.rev.compl                                                           ttcaaataaatggaatcacacatccacacttttcaatgtctggcttccttctctaaatac  13092

exon11.USP18.human                                                            ------------------------------------------------------------  0
LOC102725072.(18846286-18861064new.ref                                        ------------------------------------------------------------  14779
LOC102725072.start-FAM230F.start.18846286-18865042.NEW.ref                    ------------------------------------------------------------  16904
chimp.100000-160000.Pan.troglodytes.isolate.Yerkes.chimp.pedigree.#C0471      atttttctgttttgagattcacccatgctgttgtgcgattctatagttctattttaatca  22602
BCRP2.ref.rev.compl                                                           atttttctgttttgagattcacccatgctgttgtgcgattctatagttctcttttaatca  13152

exon11.USP18.human                                                            ------------------------------------------------------------  0
LOC102725072.(18846286-18861064new.ref                                        ------------------------------------------------------------  14779
LOC102725072.start-FAM230F.start.18846286-18865042.NEW.ref                    ------------------------------------------------------------  16904
chimp.100000-160000.Pan.troglodytes.isolate.Yerkes.chimp.pedigree.#C0471      ctgaatatatactgttgtgtgatacaccatggtttatttatttattcactgtcaaaggac  22662
BCRP2.ref.rev.compl                                                           ctgaatatatactgttgtgtgatacaccatggtttatttatttattcactgtcaaaggac  13212

exon11.USP18.human                                                            ------------------------------------------------------------  0
LOC102725072.(18846286-18861064new.ref                                        ------------------------------------------------------------  14779
LOC102725072.start-FAM230F.start.18846286-18865042.NEW.ref                    ------------------------------------------------------------  16904
chimp.100000-160000.Pan.troglodytes.isolate.Yerkes.chimp.pedigree.#C0471      attggctcatttccaagtttgggttaacgtaaataagactgctatgttagattctagtac  22722
BCRP2.ref.rev.compl                                                           attggctcatttccaagtttgggttaacgtaaataagactgctatgttagattctagtac  13272

exon11.USP18.human                                                            ------------------------------------------------------------  0
LOC102725072.(18846286-18861064new.ref                                        ------------------------------------------------------------  14779
LOC102725072.start-FAM230F.start.18846286-18865042.NEW.ref                    ------------------------------------------------------------  16904
chimp.100000-160000.Pan.troglodytes.isolate.Yerkes.chimp.pedigree.#C0471      aagtctacgtaggtgtacgtgtgtatacatacacatacatacatatacatacacacatag  22782
BCRP2.ref.rev.compl                                                           aagtctacgtaggtgtacgtgtgtatacatacacatacatacacatacatacacacatag  13332

exon11.USP18.human                                                            ------------------------------------------------------------  0
LOC102725072.(18846286-18861064new.ref                                        ------------------------------------------------------------  14779
LOC102725072.start-FAM230F.start.18846286-18865042.NEW.ref                    ------------------------------------------------------------  16904
chimp.100000-160000.Pan.troglodytes.isolate.Yerkes.chimp.pedigree.#C0471      acttgtactagaaaaaatgtactaaatgtactaaaaaatgtactaaaaatgtacattttt  22842
BCRP2.ref.rev.compl                                                           acttgtactagaaaaaatgtactaaatgtactaaaaaatgtact-aaaatgtacattttt  13391

exon11.USP18.human                                                            ------------------------------------------------------------  0
```

```
LOC102725072.(18846286-18861064new.ref                                   ------------------------------------------------------------  14779
LOC102725072.start-FAM230F.start.18846286-18865042.NEW.ref               ------------------------------------------------------------  16904
chimp.100000-160000.Pan.troglodytes.isolate.Yerkes.chimp.pedigree.#C0471 attttcttggatcaacagcttggagtaaaagtgctgagtcataggttaagtgtatattt  22902
BCRP2.ref.rev.compl                                                      attttcttggatcaacagcttggagtaaaagtgctgagtcataggttaagtgtatattt  13451

exon11.USP18.human                                                       ------------------------------------------------------------  0
LOC102725072.(18846286-18861064new.ref                                   ------------------------------------------------------------  14779
LOC102725072.start-FAM230F.start.18846286-18865042.NEW.ref               ------------------------------------------------------------  16904
chimp.100000-160000.Pan.troglodytes.isolate.Yerkes.chimp.pedigree.#C0471 aatattataagaaattgctaaaccatttccaaagtagttgttttacaattccaccagttt  22962
BCRP2.ref.rev.compl                                                      aatattacaagaaattgctacaccatttccaaagtagttgttttacaattccaccagttt  13511

exon11.USP18.human                                                       ------------------------------------------------------------  0
LOC102725072.(18846286-18861064new.ref                                   ------------------------------------------------------------  14779
LOC102725072.start-FAM230F.start.18846286-18865042.NEW.ref               ------------------------------------------------------------  16904
chimp.100000-160000.Pan.troglodytes.isolate.Yerkes.chimp.pedigree.#C0471 tatgagagtgttggttaattcttattttcatcaacatttagtattatcagtcgttttgat  23022
BCRP2.ref.rev.compl                                                      tatgagagtgttggttaattcttattttcatcaacatttagtattatcagtcgttttgat  13571

exon11.USP18.human                                                       ------------------------------------------------------------  0
LOC102725072.(18846286-18861064new.ref                                   ------------------------------------------------------------  14779
LOC102725072.start-FAM230F.start.18846286-18865042.NEW.ref               ------------------------------------------------------------  16904
chimp.100000-160000.Pan.troglodytes.isolate.Yerkes.chimp.pedigree.#C0471 tttgccattacagtggctaaaatgtgaaatcaaatgaaattgtatcttgagattttaact  23082
BCRP2.ref.rev.compl                                                      tttgccattacagtggctaaaatgtgaaatcaaatgaaattgtatcttgagattttaact  13631

exon11.USP18.human                                                       ------------------------------------------------------------  0
LOC102725072.(18846286-18861064new.ref                                   ------------------------------------------------------------  14779
LOC102725072.start-FAM230F.start.18846286-18865042.NEW.ref               ------------------------------------------------------------  16904
chimp.100000-160000.Pan.troglodytes.isolate.Yerkes.chimp.pedigree.#C0471 tgcattttcctgactgttagtgatgttgaccattgtttaatatacccactggctatttgt  23142
BCRP2.ref.rev.compl                                                      tgcatcttcctgactgttagtgatgttgaccattgtttaatatacccactggctatttgt  13691

exon11.USP18.human                                                       ------------------------------------------------------------  0
LOC102725072.(18846286-18861064new.ref                                   ------------------------------------------------------------  14779
LOC102725072.start-FAM230F.start.18846286-18865042.NEW.ref               ------------------------------------------------------------  16904
chimp.100000-160000.Pan.troglodytes.isolate.Yerkes.chimp.pedigree.#C0471 atgtcttgctttgcaaactgtctgttcaagtttttctacttttaattggcctgtttgcc  23202
BCRP2.ref.rev.compl                                                      atgtcttgctttgcaaactgtctgttcaagtttttctacttttaattggcctgtttgcc  13751

exon11.USP18.human                                                       ------------------------------------------------------------  0
LOC102725072.(18846286-18861064new.ref                                   ------------------------------------------------------------  14779
LOC102725072.start-FAM230F.start.18846286-18865042.NEW.ref               ------------------------------------------------------------  16904
chimp.100000-160000.Pan.troglodytes.isolate.Yerkes.chimp.pedigree.#C0471 tctgtattgttaacttgtaaaagttctttatatgagatgaattatttgacaaatgcatgt  23262
BCRP2.ref.rev.compl                                                      tctgtattgttaacttgtaaaagttctttatatgagatgaattatttgacaaatgcatgt  13811

exon11.USP18.human                                                       ------------------------------------------------------------  0
LOC102725072.(18846286-18861064new.ref                                   ------------------------------------------------------------  14779
LOC102725072.start-FAM230F.start.18846286-18865042.NEW.ref               ------------------------------------------------------------  16904
chimp.100000-160000.Pan.troglodytes.isolate.Yerkes.chimp.pedigree.#C0471 attacatatatttccccaagtctgtggcttgcatattcatgtttatagtgtcttttgata  23322
BCRP2.ref.rev.compl                                                      attacatatatttccccaagtctgtggcttgcatattcatgtttatagtgtcttttgata  13871

exon11.USP18.human                                                       ------------------------------------------------------------  0
LOC102725072.(18846286-18861064new.ref                                   ------------------------------------------------------------  14779
LOC102725072.start-FAM230F.start.18846286-18865042.NEW.ref               ------------------------------------------------------------  16904
chimp.100000-160000.Pan.troglodytes.isolate.Yerkes.chimp.pedigree.#C0471 acctttaattttcactgaagtttaacctataaatgtctttatgtagggttagagctttct  23382
BCRP2.ref.rev.compl                                                      acctttaattttcactgaagtttaacctataaatgtctttatgtagggttagagctttct  13931

exon11.USP18.human                                                       ------------------------------------------------------------  0
LOC102725072.(18846286-18861064new.ref                                   ------------------------------------------------------------  14779
LOC102725072.start-FAM230F.start.18846286-18865042.NEW.ref               ------------------------------------------------------------  16904
chimp.100000-160000.Pan.troglodytes.isolate.Yerkes.chimp.pedigree.#C0471 gtgtccttacaagaaagttctacctgttccaaggttagaaagaagtacccatttgttttc  23442
BCRP2.ref.rev.compl                                                      gtgtccttacaagaaacttctacctgttccaaggttagaaagaagtacccatttgttttc  13991

exon11.USP18.human                                                       ------------------------------------------------------------  0
LOC102725072.(18846286-18861064new.ref                                   ------------------------------------------------------------  14779
LOC102725072.start-FAM230F.start.18846286-18865042.NEW.ref               ------------------------------------------------------------  16904
chimp.100000-160000.Pan.troglodytes.isolate.Yerkes.chimp.pedigree.#C0471 cgtatggagtgagaatgggatgcaaatatttatatttgttattcaagcagcatttggta  23502
BCRP2.ref.rev.compl                                                      cgtatggagtgagaatgggatgcaaatatttatatttgttattcaagcagcatttggta  14051

exon11.USP18.human                                                       ------------------------------------------------------------  0
LOC102725072.(18846286-18861064new.ref                                   ------------------------------------------------------------  14779
LOC102725072.start-FAM230F.start.18846286-18865042.NEW.ref               ------------------------------------------------------------  16904
chimp.100000-160000.Pan.troglodytes.isolate.Yerkes.chimp.pedigree.#C0471 caaaaattcttcttccctctttgaattcccttgtcaacttctgagttcacttttttcctca  23562
BCRP2.ref.rev.compl                                                      caaaaattcttcttccctctttgaattcccttgtcaacttctgagttcacttttttcctca  14111

exon11.USP18.human                                                       ------------------------------------------------------------  0
LOC102725072.(18846286-18861064new.ref                                   ------------------------------------------------------------  14779
LOC102725072.start-FAM230F.start.18846286-18865042.NEW.ref               ------------------------------------------------------------  16904
chimp.100000-160000.Pan.troglodytes.isolate.Yerkes.chimp.pedigree.#C0471 tgcacattattggtaagcattttgtgtaaaataaactgcctttatcagaagtgtacaatt  23622
BCRP2.ref.rev.compl                                                      tgcacattattggtaagcattttgtgtaaaataaactgcctttatcagaagtgtacaatt  14171

exon11.USP18.human                                                       ------------------------------------------------------------  0
LOC102725072.(18846286-18861064new.ref                                   ------------------------------------------------------------  14779
LOC102725072.start-FAM230F.start.18846286-18865042.NEW.ref               ------------------------------------------------------------  16904
chimp.100000-160000.Pan.troglodytes.isolate.Yerkes.chimp.pedigree.#C0471 ttttcagatatctttccataatcctctagatttgaaaggtgattttttggttctcttgctg  23682
BCRP2.ref.rev.compl                                                      ttttcagatatctttccataatcctctagatttgaaaggtgattttttggttctcttgctg  14231

exon11.USP18.human                                                       ------------------------------------------------------------  0
LOC102725072.(18846286-18861064new.ref                                   ------------------------------------------------------------  14779
LOC102725072.start-FAM230F.start.18846286-18865042.NEW.ref               ------------------------------------------------------------  16904
chimp.100000-160000.Pan.troglodytes.isolate.Yerkes.chimp.pedigree.#C0471 acacgttataattatagctcccttctgctgccatgtgatttgcaatcctttttaatcttt  23742
BCRP2.ref.rev.compl                                                      acatgttataattatagctcccttctgctgccatgtgatttgcaatcctttttaatcttt  14291

exon11.USP18.human                                                       ------------------------------------------------------------  0
LOC102725072.(18846286-18861064new.ref                                   ------------------------------------------------------------  14779
LOC102725072.start-FAM230F.start.18846286-18865042.NEW.ref               ------------------------------------------------------------  16904
chimp.100000-160000.Pan.troglodytes.isolate.Yerkes.chimp.pedigree.#C0471 ccatttaagacttttcttactcctatatgaaaactataattgtggccaggcatggtggct  23802
BCRP2.ref.rev.compl                                                      ccatttaagacctttcttactcctatataaaaactataattgtggccaggcatggtggct  14351

exon11.USP18.human                                                       ------------------------------------------------------------  0
LOC102725072.(18846286-18861064new.ref                                   ------------------------------------------------------------  14779
LOC102725072.start-FAM230F.start.18846286-18865042.NEW.ref               ------------------------------------------------------------  16904
chimp.100000-160000.Pan.troglodytes.isolate.Yerkes.chimp.pedigree.#C0471 cacacctgtaatcccagcactttgggaggccgaggtgggtggatcatctgaggtcaggag  23862
BCRP2.ref.rev.compl                                                      cacacctgtaatcccagcactttgggaggccgaggtgggtggatcatctgaggtcaggag  14411

exon11.USP18.human                                                       ------------------------------------------------------------  0
LOC102725072.(18846286-18861064new.ref                                   ------------------------------------------------------------  14779
LOC102725072.start-FAM230F.start.18846286-18865042.NEW.ref               ------------------------------------------------------------  16904
chimp.100000-160000.Pan.troglodytes.isolate.Yerkes.chimp.pedigree.#C0471 ttcgagaccagcctgggcaacatggtgaaaccctgtctctactaaaaatacgaaaataag  23922
BCRP2.ref.rev.compl                                                      ttcgagaccagcctgggcaacatggtgaaaccctgtctctactaaaaataccaaaataag  14471

exon11.USP18.human                                                       ------------------------------------------------------------  0
LOC102725072.(18846286-18861064new.ref                                   ------------------------------------------------------------  14779
LOC102725072.start-FAM230F.start.18846286-18865042.NEW.ref               ---------------------------------actcgggaggctgaggagggagaatcg  16931
chimp.100000-160000.Pan.troglodytes.isolate.Yerkes.chimp.pedigree.#C0471 ctgggcgtggtgtgtgtgcctgtagtcccagctactccggaggctgaggcaggagaatcg  23982
BCRP2.ref.rev.compl                                                      ctgggtgtggtgtgtgtgcctgtagtcccagctactcgggaggctgaggcaggagaatcg  14531

exon11.USP18.human                                                       ------------------------------------------------------------  0
LOC102725072.(18846286-18861064new.ref                                   ------------------------------------------------------------  14779
LOC102725072.start-FAM230F.start.18846286-18865042.NEW.ref               tttgaacctgggaggcggaggttgcagtgagccaagattgtgccactgcactctagcctg  16991
chimp.100000-160000.Pan.troglodytes.isolate.Yerkes.chimp.pedigree.#C0471 cttaaagccaggaggtggaggttgcagtgagtcaagatcacgcccctgcactcaggcctg  24042
BCRP2.ref.rev.compl                                                      cttaaagccaggaggtggaggttgcagtgagtcaagatcgcgcccctgcactcaggcctg  14591

exon11.USP18.human                                                       ------------------------------------------------------------  0
LOC102725072.(18846286-18861064new.ref                                   ------------------------------------------------------------  14779
LOC102725072.start-FAM230F.start.18846286-18865042.NEW.ref               ggctacagggcaagactccattaaaaaaaaaaaaaaaaccagcaaaaaacaa--------  17043
chimp.100000-160000.Pan.troglodytes.isolate.Yerkes.chimp.pedigree.#C0471 ggcgacagagcaagactctgtctc-aaaaaaaaaaaaaaaaaaaatcctataattgcttt  24101
BCRP2.ref.rev.compl                                                      ggtgacagagcaagattctgtctcaaaaaaaaaaaaaaaaaaaaatcctataattgcttt  14651
```

```
exon11.USP18.human                                                        ------------------------------------------------------------  0
LOC102725072.(18846286-18861064new.ref                                    ------------------------------------------------------------  14779
LOC102725072.start-FAM230F.start.18846286-18865042.NEW.ref                ------------------------------------------------------------  17043
chimp.100000-160000.Pan.troglodytes.isolate.Yerkes.chimp.pedigree.#C0471  agtcatgcatatattgaaacactgtgtacaataattaggcaattgaatttttgtattttt  24161
BCRP2.ref.rev.compl                                                       agtcatgcatatattgaaacactgtgtacaataattaggcaattgaatttttgtattttt  14711

exon11.USP18.human                                                        ------------------------------------------------------------  0
LOC102725072.(18846286-18861064new.ref                                    ------------------------------------------------------------  14779
LOC102725072.start-FAM230F.start.18846286-18865042.NEW.ref                ------------------------------------------------------------  17043
chimp.100000-160000.Pan.troglodytes.isolate.Yerkes.chimp.pedigree.#C0471  ctaaaattcaaaaggagaaaagctaatctactttgtaatgatatagattcactaagacac  24221
BCRP2.ref.rev.compl                                                       ctaaaattcaaaaggagaaaagctaatctagtttgtaatgatatagattcactaagacac  14771

exon11.USP18.human                                                        ------------------------------------------------------------  0
LOC102725072.(18846286-18861064new.ref                                    ------------------------------------------------------------  14779
LOC102725072.start-FAM230F.start.18846286-18865042.NEW.ref                ------------------------------------------------------------  17043
chimp.100000-160000.Pan.troglodytes.isolate.Yerkes.chimp.pedigree.#C0471  ttgtttctgtctttaggatagagcattcatactaacagtgtgagtagtatggataaacca  24281
BCRP2.ref.rev.compl                                                       ttgtttctgtctttaggatagagcattcatactaacagtgtgagtagtatggataaacca  14831

exon11.USP18.human                                                        ------------------------------------------------------------  0
LOC102725072.(18846286-18861064new.ref                                    ------------------------------------------------------------  14779
LOC102725072.start-FAM230F.start.18846286-18865042.NEW.ref                ------------------------------------------------------------  17043
chimp.100000-160000.Pan.troglodytes.isolate.Yerkes.chimp.pedigree.#C0471  gcatatatatttttattatgttgaatttcaatatccagccgaattctgtctagttgcat  24341
BCRP2.ref.rev.compl                                                       gcatatatatttttattatgttgaatttcaatatccagccgaattctgtctagttgcat  14891

exon11.USP18.human                                                        ------------------------------------------------------------  0
LOC102725072.(18846286-18861064new.ref                                    ------------------------------------------------------------  14779
LOC102725072.start-FAM230F.start.18846286-18865042.NEW.ref                ------------------------------------------------------------  17043
chimp.100000-160000.Pan.troglodytes.isolate.Yerkes.chimp.pedigree.#C0471  tacttacaacattgtgttacagtttgaaatcaagaagaaacttgtgaatgacttttctac  24401
BCRP2.ref.rev.compl                                                       tacttacaacattgtgttacagtttgaaatcaagaagaaacttgtgaatgacttttctac  14951

exon11.USP18.human                                                        ------------------------------------------------------------  0
LOC102725072.(18846286-18861064new.ref                                    ------------------------------------------------------------  14779
LOC102725072.start-FAM230F.start.18846286-18865042.NEW.ref                ---------------------------acaaaacataatgcatgttctctcttataaatg  17076
chimp.100000-160000.Pan.troglodytes.isolate.Yerkes.chimp.pedigree.#C0471  aatttctctgtaaaacatagatttgtcagataacatagtaccttatgtggtaattaaaga  24461
BCRP2.ref.rev.compl                                                       aatttctctgtaaaacatagatttgtcagataacatagtaccttatgtggtaattaaaga  15011

exon11.USP18.human                                                        ------------------------------------------------------------  0
LOC102725072.(18846286-18861064new.ref                                    ------------------------------------------------------------  14779
LOC102725072.start-FAM230F.start.18846286-18865042.NEW.ref                ggagctaaacatggggactcattgacttaaagatggcaacaactgggaactgctggatgg  17136
chimp.100000-160000.Pan.troglodytes.isolate.Yerkes.chimp.pedigree.#C0471  aattgttacaatgagaacacatggacacagagaggggaacatcacacaccatggcatgtc  24521
BCRP2.ref.rev.compl                                                       aattgttacaatgagaacacatggacacagagaggggaacatcacacaccatggcatgtc  15071

exon11.USP18.human                                                        ------------------------------------------------------------  0
LOC102725072.(18846286-18861064new.ref                                    ------------------------------------------------------------  14779
LOC102725072.start-FAM230F.start.18846286-18865042.NEW.ref                ggagggaggggaggggtgaaaggccaactgttggggagtatgct----------------  17180
chimp.100000-160000.Pan.troglodytes.isolate.Yerkes.chimp.pedigree.#C0471  aggggggttgggggcaaggggagggagagcattagggcacatacctaatgcataccgggct  24581
BCRP2.ref.rev.compl                                                       aggggggttgggggcaaggggagggagagcattagggcacatacctaatgcataccaggct  15131

exon11.USP18.human                                                        ------------------------------------------------------------  0
LOC102725072.(18846286-18861064new.ref                                    ------------------------------------------------------------  14779
LOC102725072.start-FAM230F.start.18846286-18865042.NEW.ref                --------------------------------------------------catatccacg  17190
chimp.100000-160000.Pan.troglodytes.isolate.Yerkes.chimp.pedigree.#C0471  tgaaacctagatgatgggttgataggtgcagcaaaccaccatggcacatgtatacctatg  24641
BCRP2.ref.rev.compl                                                       tgaaacctagatgacgggttgatagatgcagcaaaccaccatggcacatgtatacctatg  15191

exon11.USP18.human                                                        ------------------------------------------------------------  0
LOC102725072.(18846286-18861064new.ref                                    ------------------------------------------------------------  14779
LOC102725072.start-FAM230F.start.18846286-18865042.NEW.ref                tgacaaacctgcacatgtgcccgctgaatc--------taaaataaaagttgaaagtaga  17242
chimp.100000-160000.Pan.troglodytes.isolate.Yerkes.chimp.pedigree.#C0471  taacaaacctgcacattctgcacatgtatcccaggagttaaagtaaaatttaaaaagaaa  24701
BCRP2.ref.rev.compl                                                       taacaaacctgcacattctgcacatgtatcccaggacttaaagtaaaatttaaaaagaaa  15251

exon11.USP18.human                                                        ------------------------------------------------------------  0
LOC102725072.(18846286-18861064new.ref                                    ------------------------------------------------------------  14779
LOC102725072.start-FAM230F.start.18846286-18865042.NEW.ref                tt----------------------------------------------------------  17244
chimp.100000-160000.Pan.troglodytes.isolate.Yerkes.chimp.pedigree.#C0471  ttgttacataaatgttttagtggctttaatttttaattaaattgtgttaactttttagga  24761
BCRP2.ref.rev.compl                                                       ttgttacataaatgttttagtggctttaatttttaattaaattgtgttaactttttagga  15311

exon11.USP18.human                                                        ------------------------------------------------------------  0
LOC102725072.(18846286-18861064new.ref                                    ------------------------------------------------------------  14779
LOC102725072.start-FAM230F.start.18846286-18865042.NEW.ref                ------------------------------------------------------------  17244
chimp.100000-160000.Pan.troglodytes.isolate.Yerkes.chimp.pedigree.#C0471  cacgaaaaattaaaatatgaaaagtaaaattttactatatgcacggaaaaatctcattta  24821
BCRP2.ref.rev.compl                                                       cacgaaaaattaaaatatgaaaagtaaaattttactatatgcacagaaaaatctcattta  15371

exon11.USP18.human                                                        ------------------------------------------------------------  0
LOC102725072.(18846286-18861064new.ref                                    ------------------------------------------------------------  14779
LOC102725072.start-FAM230F.start.18846286-18865042.NEW.ref                ------------------------------------------------------------  17244
chimp.100000-160000.Pan.troglodytes.isolate.Yerkes.chimp.pedigree.#C0471  cttctgtgaggcaggaagtcttatactctgagtggggaaatataaatggcacctaattcc  24881
BCRP2.ref.rev.compl                                                       cttctgtgaggcaggaagtcttatactctgagtggggaaatataaatggcacctaattcc  15431

exon11.USP18.human                                                        ------------------------------------------------------------  0
LOC102725072.(18846286-18861064new.ref                                    ------------------------------------------------------------  14779
LOC102725072.start-FAM230F.start.18846286-18865042.NEW.ref                ------------------------------------------------------------  17244
chimp.100000-160000.Pan.troglodytes.isolate.Yerkes.chimp.pedigree.#C0471  tgcatttgattattaccgtttttggctgttgccacaataggtttatctttttacacatca  24941
BCRP2.ref.rev.compl                                                       tgcatttgattattaccgttcttggctgttgccacaataggtttatctttttacacatca  15491

exon11.USP18.human                                                        ------------------------------------------------------------  0
LOC102725072.(18846286-18861064new.ref                                    ------------------------------------------------------------  14779
LOC102725072.start-FAM230F.start.18846286-18865042.NEW.ref                ------------------------------------------------------------  17244
chimp.100000-160000.Pan.troglodytes.isolate.Yerkes.chimp.pedigree.#C0471  aaaaaaggaaattatgaggaatcatatatctgaaggggagtgttatctgacaatagcaga  25001
BCRP2.ref.rev.compl                                                       aaaaaaggaaattatgaggaatcatatatctgaaagggagtgttatctgacaacagcaga  15551

exon11.USP18.human                                                        ------------------------------------------------------------  0
LOC102725072.(18846286-18861064new.ref                                    ------------------------------------------------------------  14779
LOC102725072.start-FAM230F.start.18846286-18865042.NEW.ref                ------------------------------------------------------------  17244
chimp.100000-160000.Pan.troglodytes.isolate.Yerkes.chimp.pedigree.#C0471  aactaacactacagaggggcaaaacatacaagacccaatacccccaaaaaaactgggcaa  25061
BCRP2.ref.rev.compl                                                       aactaacactacagagggacaaaacatacaagacccaatacccccaaaaaaactgggc-a  15610

exon11.USP18.human                                                        ------------------------------------------------------------  0
LOC102725072.(18846286-18861064new.ref                                    ------------------------------------------------------------  14779
LOC102725072.start-FAM230F.start.18846286-18865042.NEW.ref                ------------------------------------------------------------  17244
chimp.100000-160000.Pan.troglodytes.isolate.Yerkes.chimp.pedigree.#C0471  aaaaaacctgaataacagacatttctgaaaagaaggtatgatattgattaaaaggtaaaa  25121
BCRP2.ref.rev.compl                                                       aaaaaacctgaataacagacatttctgaaaagaaggtatgatattgattaaaaggtaaaa  15670

exon11.USP18.human                                                        ------------------------------------------------------------  0
LOC102725072.(18846286-18861064new.ref                                    ------------------------------------------------------------  14779
LOC102725072.start-FAM230F.start.18846286-18865042.NEW.ref                ------------------------------------------------------------  17244
chimp.100000-160000.Pan.troglodytes.isolate.Yerkes.chimp.pedigree.#C0471  aaggtgtttctcaacatcactggtcatcacaaaactgtaaataaagaccacactcagata  25181
BCRP2.ref.rev.compl                                                       aaggtgtttctcaacatcactggtcatcacaaaactgtaaataaagaccacactcagata  15730

exon11.USP18.human                                                        ------------------------------------------------------------  0
LOC102725072.(18846286-18861064new.ref                                    ------------------------------------------------------------  14779
LOC102725072.start-FAM230F.start.18846286-18865042.NEW.ref                ------------------------------------------------------------  17244
chimp.100000-160000.Pan.troglodytes.isolate.Yerkes.chimp.pedigree.#C0471  ctatctaacgctaagtaaaatgaataataccaaaacagaaataatttaaaaataaaagtt  25241
BCRP2.ref.rev.compl                                                       ctatctaacgctaagtaaaatgaataataccaaaacagaaataatttaaaaataaaagtt  15790

exon11.USP18.human                                                        ------------------------------------------------------------  0
LOC102725072.(18846286-18861064new.ref                                    ------------------------------------------------------------  14779
LOC102725072.start-FAM230F.start.18846286-18865042.NEW.ref                ------------------------------------------------------------  17244
chimp.100000-160000.Pan.troglodytes.isolate.Yerkes.chimp.pedigree.#C0471  cagggtgctgttagatatagagatcctcttatacactattgggagaaatttcaatgatga  25301
BCRP2.ref.rev.compl                                                       cagggtgctgttagatatagagatcctcttatacactattgggagaaatttcaatgatga  15850

exon11.USP18.human                                                        ------------------------------------------------------------  0
LOC102725072.(18846286-18861064new.ref                                    ------------------------------------------------------------  14779
LOC102725072.start-FAM230F.start.18846286-18865042.NEW.ref                ------------------------------------------------------------  17244
chimp.100000-160000.Pan.troglodytes.isolate.Yerkes.chimp.pedigree.#C0471  aacgcactgagaaaaccacttggagcatcctcagaaattaaaaatgcaagaactgtttca  25361
```

```
BCRP2.ref.rev.compl                                                 aacgcactgagaaaaccacttggagcaccctcagaaattaaaaatgcaagaactgtttca  15910

exon11.USP18.human                                                  ------------------------------------------------------------  0
LOC102725072.(18846286-18861064new.ref                              ------------------------------------------------------------  14779
LOC102725072.start-FAM230F.start.18846286-18865042.NEW.ref          ------------------------------------------------------------  17244
chimp.100000-160000.Pan.troglodytes.isolate.Yerkes.chimp.pedigree.#C0471  tctagtaattctaaccgtggttatgcgcttaaagaaaatgagatcggtctgttgaagcaa  25421
BCRP2.ref.rev.compl                                                 tctagtaattctaaccgtggttgtgagcttaaagaaaatgagatcggtctgttgaagcaa  15970

exon11.USP18.human                                                  ------------------------------------------------------------  0
LOC102725072.(18846286-18861064new.ref                              ------------------------------------------------------------  14779
LOC102725072.start-FAM230F.start.18846286-18865042.NEW.ref          ------------------------------------------------------------  17244
chimp.100000-160000.Pan.troglodytes.isolate.Yerkes.chimp.pedigree.#C0471  cagctgccttctgaggtttagtgacctactactcactaacaatgagtaaccaaggtatga  25481
BCRP2.ref.rev.compl                                                 cagctgccttctgaggtttagtgacctactactcactaacaatgagtaaccaaggtatga  16030

exon11.USP18.human                                                  ------------------------------------------------------------  0
LOC102725072.(18846286-18861064new.ref                              ------------------------------------------------------------  14779
LOC102725072.start-FAM230F.start.18846286-18865042.NEW.ref          ------------------------------------------------------------  17244
chimp.100000-160000.Pan.troglodytes.isolate.Yerkes.chimp.pedigree.#C0471  aatcaacctacctgtccaccaaccaatgaaaggatgaagggacttcagtatacagacatg  25541
BCRP2.ref.rev.compl                                                 aatcaacctacctgtccaccaaccaatgaaaggatgaagggacttcagcatacagacatg  16090

exon11.USP18.human                                                  ------------------------------------------------------------  0
LOC102725072.(18846286-18861064new.ref                              ------------------------------------------------------------  14779
LOC102725072.start-FAM230F.start.18846286-18865042.NEW.ref          ------------------------------------------------------------  17244
chimp.100000-160000.Pan.troglodytes.isolate.Yerkes.chimp.pedigree.#C0471  atgaaatatgcctcatgcataaaaattcatgacaacatgtcatttgcaaaaacacagatg  25601
BCRP2.ref.rev.compl                                                 atgaaatatgcctcatgcataaaaattcatgacaacatgtcatttgcaaaaacacagacg  16150

exon11.USP18.human                                                  ------------------------------------------------------------  0
LOC102725072.(18846286-18861064new.ref                              ------------------------------------------------------------  14779
LOC102725072.start-FAM230F.start.18846286-18865042.NEW.ref          ------------------------------------------------------------  17244
chimp.100000-160000.Pan.troglodytes.isolate.Yerkes.chimp.pedigree.#C0471  aacctggaagacattacattaaatgaaatcgactaggtagagaaaggcaaacactacgtg  25661
BCRP2.ref.rev.compl                                                 aacctggaagacattacattaaatgaaatcgactaggtagagaaaggcaaacactatgtg  16210

exon11.USP18.human                                                  ------------------------------------------------------------  0
LOC102725072.(18846286-18861064new.ref                              ------------------------------------------------------------  14779
LOC102725072.start-FAM230F.start.18846286-18865042.NEW.ref          ------------------------------------------------------------  17244
chimp.100000-160000.Pan.troglodytes.isolate.Yerkes.chimp.pedigree.#C0471  atctcacttatatggaatccaaaaaactttaccttcaagcagaaggtacagtagcggttt  25721
BCRP2.ref.rev.compl                                                 atctcacttatatggaatccaaaaaactttaccttcaagcagaaggtacagtagcggttt  16270

exon11.USP18.human                                                  ------------------------------------------------------------  0
LOC102725072.(18846286-18861064new.ref                              ------------------------------------------------------------  14779
LOC102725072.start-FAM230F.start.18846286-18865042.NEW.ref          ------------------------------------------------------------  17244
chimp.100000-160000.Pan.troglodytes.isolate.Yerkes.chimp.pedigree.#C0471  cctgaggagaggggaaggaggggggataggagggggatgggagggatcggttatggaaaca  25781
BCRP2.ref.rev.compl                                                 cctgaggagaggggaaggaggggggataggagggggatgggagggatcggttatggaaaca  16330

exon11.USP18.human                                                  ------------------------------------------------------------  0
LOC102725072.(18846286-18861064new.ref                              ------------------------------------------------------------  14779
LOC102725072.start-FAM230F.start.18846286-18865042.NEW.ref          ------------------------------------------------------------  17244
chimp.100000-160000.Pan.troglodytes.isolate.Yerkes.chimp.pedigree.#C0471  aagttacagtgagtttggagaaacacattttggttttctatgccacagctgggtaactct  25841
BCRP2.ref.rev.compl                                                 aagttacagtgagtttggcgaaacacattttggttttctatgccacagctgggtaactct  16390

exon11.USP18.human                                                  ------------------------------------------------------------  0
LOC102725072.(18846286-18861064new.ref                              ------------------------------------------------------------  14779
LOC102725072.start-FAM230F.start.18846286-18865042.NEW.ref          ------------------------------------------------------------  17244
chimp.100000-160000.Pan.troglodytes.isolate.Yerkes.chimp.pedigree.#C0471  ggttaacacaatattgtatttttcaaaaaactagaagggagcaatttgaatgtactcaca  25901
BCRP2.ref.rev.compl                                                 ggttaacaaaatattgtatttttcaaaaaactagaagggagcaatttgaatgtactcaca  16450

exon11.USP18.human                                                  ------------------------------------------------------------  0
LOC102725072.(18846286-18861064new.ref                              ------------------------------------------------------------  14779
LOC102725072.start-FAM230F.start.18846286-18865042.NEW.ref          ------------------------------------------------------------  17244
chimp.100000-160000.Pan.troglodytes.isolate.Yerkes.chimp.pedigree.#C0471  acaaagaaataatacctgaatgagggaatagttatgatgagtaccctgatttgatcatta  25961
BCRP2.ref.rev.compl                                                 acaaagaaataatacctcaatgagggaatagttatgataagtaccctgatttgatcatta  16510

exon11.USP18.human                                                  ------------------------------------------------------------  0
LOC102725072.(18846286-18861064new.ref                              ------------------------------------------------------------  14779
LOC102725072.start-FAM230F.start.18846286-18865042.NEW.ref          ----------------------------taaaaaaccccaagagggctgggtttggcttg  17276
chimp.100000-160000.Pan.troglodytes.isolate.Yerkes.chimp.pedigree.#C0471  ctcaaaatatacacgtatcaaaatgtccccaaagaaaaaaacatgtcccagcataacctc  26021
BCRP2.ref.rev.compl                                                 ctcaaagtatacatgtatcaaaatgtccccaaagaaaaaaacatgtcccagcataacttc  16570

exon11.USP18.human                                                  ------------------------------------------------------------  0
LOC102725072.(18846286-18861064new.ref                              ------------------------------------------------------------  14779
LOC102725072.start-FAM230F.start.18846286-18865042.NEW.ref          tgtgtccatagcttgttaacctccgctttagatattaactaatagaaacctagtgcttat  17336
chimp.100000-160000.Pan.troglodytes.isolate.Yerkes.chimp.pedigree.#C0471  taattgtgtatgttattatacatggcaaaaatttaagaaatatgaatataaaatgctaat  26081
BCRP2.ref.rev.compl                                                 taattgtgtatgttattatacatggcaaaaatttaagaaatatgaatataaaatgctaat  16630

exon11.USP18.human                                                  ------------------------------------------------------------  0
LOC102725072.(18846286-18861064new.ref                              ------------------------------------------------------------  14779
LOC102725072.start-FAM230F.start.18846286-18865042.NEW.ref          cttcccaggccacctatttgttcctctcc-------------------------------  17366
chimp.100000-160000.Pan.troglodytes.isolate.Yerkes.chimp.pedigree.#C0471  attcacatagaaccactaatggccctgaacctctaaggaatcctgagaaatacaaacaaa  26141
BCRP2.ref.rev.compl                                                 attcacatagaaccactaatggccctgaacctctaaggaatcctgagaaatacaaactaa  16690

exon11.USP18.human                                                  ------------------------------------------------------------  0
LOC102725072.(18846286-18861064new.ref                              ------------------------------------------------------------  14779
LOC102725072.start-FAM230F.start.18846286-18865042.NEW.ref          ------------------------------------------------------------  17366
chimp.100000-160000.Pan.troglodytes.isolate.Yerkes.chimp.pedigree.#C0471  gttgggggactcacaatccctgatagcaaattacattgcaaagctgtaggtagccaatat  26201
BCRP2.ref.rev.compl                                                 gttgggagactcacaattcctgatagcaaattacattgcaaagctgtaggtagccaatat  16750

exon11.USP18.human                                                  ------------------------------------------------------------  0
LOC102725072.(18846286-18861064new.ref                              ------------------------------------------------------------  14779
LOC102725072.start-FAM230F.start.18846286-18865042.NEW.ref          ------------------------------------------------------------  17366
chimp.100000-160000.Pan.troglodytes.isolate.Yerkes.chimp.pedigree.#C0471  acatggtcctgtgatgtgctactgtgctctccagcctgggtgagagaatgagactctgtc  26261
BCRP2.ref.rev.compl                                                 atatggtcatgtgatgtgctactgtgctctccagcctgggtgagagaatgagactctgtc  16810

exon11.USP18.human                                                  ------------------------------------------------------------  0
LOC102725072.(18846286-18861064new.ref                              ------------------------------------------------------------  14779
LOC102725072.start-FAM230F.start.18846286-18865042.NEW.ref          ----------------aaggtgatggatagatgaaggcctaatccagccgcctggaagtt  17410
chimp.100000-160000.Pan.troglodytes.isolate.Yerkes.chimp.pedigree.#C0471  tcaaaaaaaaaaaagaaaagaaagggaatcatgtaagcgataactcaataactggttgtt  26321
BCRP2.ref.rev.compl                                                 tcaaaaaaaaa----aaaagaaagggaatcatgtaagcgataactcaatcactggttgtt  16866

exon11.USP18.human                                                  ------------------------------------------------------------  0
LOC102725072.(18846286-18861064new.ref                              ------------------------------------------------------------  14779
LOC102725072.start-FAM230F.start.18846286-18865042.NEW.ref          tgctgacgcttgtcctgtcacagattaatgaagcattgttttctgatgaagctttcatgc  17470
chimp.100000-160000.Pan.troglodytes.isolate.Yerkes.chimp.pedigree.#C0471  atcaaacatata------cacagaaaaaaaatacaatgtagcaaaatgcatatccaaacc  26375
BCRP2.ref.rev.compl                                                 atcaaacatata------cacagaaaaaaaatacaatgtagcaaaatacatatccaaacc  16920
```

start of Exon11.USP18 sequence in LOC102725072-FAM230F, *linc-UR-B1* and absence in chimp sequence

```
exon11.USP18.human                                                  --------------------------------gcaggaaactgcatatcttctggtttac  28
LOC102725072.(18846286-18861064new.ref                              ------------------------------------------------------------  14779
LOC102725072.start-FAM230F.start.18846286-18865042.NEW.ref          cgctgtgctgatgtgtcttctcttctctctaggcaggaaactgcatatcttctggtttac  17530
chimp.100000-160000.Pan.troglodytes.isolate.Yerkes.chimp.pedigree.#C0471  aatacaaagaacttgagtagacatctctgcaaagatgacacaaaattgaccaacatgtaa  26435
BCRP2.ref.rev.compl                                                 aatacaaagaacttgagtagacatctctgcaaagatgacacaaaattgaccaacatgtaa  16980

exon11.USP18.human                                                  atgaagatggagtgctaatggaaatgcccaaaaccttcagagattgacacg-ctgtcatt  87
LOC102725072.(18846286-18861064new.ref                              ------------------------------------------------------------  14779
LOC102725072.start-FAM230F.start.18846286-18865042.NEW.ref          atgaagatggagtgctaatggaaatgcccaaaaccttcagagattgacacg-ctgtcatt  17589
chimp.100000-160000.Pan.troglodytes.isolate.Yerkes.chimp.pedigree.#C0471  gagaaacggtcctcaacgtctctcatcagaaaaaaaatgtacctcaaaaccacattcaga  26495
BCRP2.ref.rev.compl                                                 gagaaacggtcctcaacgtctctcatcagaaaaaaaatgtacctcaaaaccacattcaga  17040

exon11.USP18.human                                                  ttccatttc-cgttcctggatctacggagtcttctaagagattttgcaatgaggagaagc  146
LOC102725072.(18846286-18861064new.ref                              ------------------------------------------------------------  14779
LOC102725072.start-FAM230F.start.18846286-18865042.NEW.ref          ttccatttc-cattcctggatctacggagtcttctaagagattttgcaatgaggagaagc  17648
chimp.100000-160000.Pan.troglodytes.isolate.Yerkes.chimp.pedigree.#C0471  tgccatttcacttttattgaaataaatgttactaaaaagtgtttttttaatttttttgaga  26555
BCRP2.ref.rev.compl                                                 tgccgtttcacttttattgaaataaatgttactaaaaagtttttttttaatttttttgaga  17100

exon11.USP18.human                                                  attgttttcaaactatataactgagccttatttataattagg------------------  188
```

```
LOC102725072.(18846286-18861064new.ref                                   ------------------------------------------------------------  14779
LOC102725072.start-FAM230F.start.18846286-18865042.NEW.ref               attgttttcaaactatataactgagccttatttataattagg------------------  17690
chimp.100000-160000.Pan.troglodytes.isolate.Yerkes.chimp.pedigree.#C0471 cagggactcagactttgtcacccaggctgaagtgcagtgtcgtgatcccagctcactgca  26615
BCRP2.ref.rev.compl                                                      cagggactcagactttgtcacccaggctgaagtgcagtgtcgtgatcccagctcactgca  17160

exon11.USP18.human                                                       ------------------------------------------------------------  188
LOC102725072.(18846286-18861064new.ref                                   ------------------------------------------------------------  14779
LOC102725072.start-FAM230F.start.18846286-18865042.NEW.ref               ------------------------------------------------------------  17690
chimp.100000-160000.Pan.troglodytes.isolate.Yerkes.chimp.pedigree.#C0471 gctttgacctccctagctcaagtgatcatcccacttcagcctctcaagtatctgggacta  26675
BCRP2.ref.rev.compl                                                      gctttgacctccctagctcaggtgatcatcccacttcagcctctcaagtatctgggacta  17220

exon11.USP18.human                                                       ------------------------------------------------------------  188
LOC102725072.(18846286-18861064new.ref                                   ------------------------------------------------------------  14779
LOC102725072.start-FAM230F.start.18846286-18865042.NEW.ref               ------------------------------------------------------------  17690
chimp.100000-160000.Pan.troglodytes.isolate.Yerkes.chimp.pedigree.#C0471 caggtgcacaccaccatgcctcatcctcccaaagtgctgggattacaggcatgagccact  26735
BCRP2.ref.rev.compl                                                      caggtgcacaccaccatgcctcatcctcccaaagtgctgggattacaggcatgagccact  17280

exon11.USP18.human                                                       ------------------------------------------------------------  188
LOC102725072.(18846286-18861064new.ref                                   ------------------------------------------------------------  14779
LOC102725072.start-FAM230F.start.18846286-18865042.NEW.ref               ------------------------------------------------------------  17690
chimp.100000-160000.Pan.troglodytes.isolate.Yerkes.chimp.pedigree.#C0471 gttcctggccacaaaattttaaaaagaatgctggtatggatttggagaaaagggaactct  26795
BCRP2.ref.rev.compl                                                      gttcctggccacaaaattttaaaaagaatgctggtatggatttggagaaaagggaactct  17340

exon11.USP18.human                                                       -----------------------------------------------------------g  189
LOC102725072.(18846286-18861064new.ref                                   ------------------------------------------------------------  14779
LOC102725072.start-FAM230F.start.18846286-18865042.NEW.ref               -----------------------------------------------------------g  17691
chimp.100000-160000.Pan.troglodytes.isolate.Yerkes.chimp.pedigree.#C0471 tataccctgttgatgggaagataaattagtacatattctatggtaaaaagttggaagttg  26855
BCRP2.ref.rev.compl                                                      tataccctgttgatgggaagataaattagtacatattctatggtaaaaagttggaagttg  17400

exon11.USP18.human                                                       atattatcaaaatatgtaaccatgaggcccctcaggtcctgatcagtcagaatggatgct  249
LOC102725072.(18846286-18861064new.ref                                   ------------------------------------------------------------  14779
LOC102725072.start-FAM230F.start.18846286-18865042.NEW.ref               atattatcaaaatatgtaaccatgaggcccctcaggtcctgatcagtcagaatggatgct  17751
chimp.100000-160000.Pan.troglodytes.isolate.Yerkes.chimp.pedigree.#C0471 ccattttcaaaaaactgaaaatacaattaccagaaggtcaatgtgggcagattgcttgag  26915
BCRP2.ref.rev.compl                                                      ccattttcaaaaaactgaaaatacaattaccagaaggtcaatgtgggcagattacttgag  17460

exon11.USP18.human                                                       ttcaccag--------cagacccggccatgtggctgctcggtcctgggtgctcgctgctg  301
LOC102725072.(18846286-18861064new.ref                                   ------------------------------------------------------------  14779
LOC102725072.start-FAM230F.start.18846286-18865042.NEW.ref               ttcaccag--------cagacccggccatgtggctgctcggtcctgggtgctcgctgctg  17803
chimp.100000-160000.Pan.troglodytes.isolate.Yerkes.chimp.pedigree.#C0471 ctcaggagttcgagaccagcctgggcaacatggcaaaaccctgtctctgcaaaaaattta  26975
BCRP2.ref.rev.compl                                                      ctcaggagttcgagaccagcctgggcaacatggcaaaaccctgtctctacaaaaaattta  17520

exon11.USP18.human                                                       tgcaagacattagccctttagttatgagcctgtgggaacttcaggggttcccagtgggga  361
LOC102725072.(18846286-18861064new.ref                                   ------------------------------------------------------------  14779
LOC102725072.start-FAM230F.start.18846286-18865042.NEW.ref               tgcgagacattagccctttagttatgagcctgtgggaacttcaggggttcccagtgggga  17863
chimp.100000-160000.Pan.troglodytes.isolate.Yerkes.chimp.pedigree.#C0471 aaaacttagtcaggcatggtggtgtgtgcctgtggtctcagctaccatagactgaggtgg  27035
BCRP2.ref.rev.compl                                                      aaaacttagccaggcatggtggtgtgtgcctgtggtctcagctaccatagactgaggtgg  17580

exon11.USP18.human                                                       gagcagtggcagtgggaggcatctggggccaaaggtcagtggca----------------  406
LOC102725072.(18846286-18861064new.ref                                   ------------------------------------------------------------  14779
LOC102725072.start-FAM230F.start.18846286-18865042.NEW.ref               gagcagtggcagtgggaggcatctggggccaaaggtcagtggca----------------  17908
chimp.100000-160000.Pan.troglodytes.isolate.Yerkes.chimp.pedigree.#C0471 gagggctgcttgggctgtggaggtagaggccacagtgagctgtgattgcaccactgcact  27095
BCRP2.ref.rev.compl                                                      gagggctgcttgggctgtggaggtagaggccacagtgagctgtgattgcaccactgcact  17640

exon11.USP18.human                                                       ------------------------------------------------------------  406
LOC102725072.(18846286-18861064new.ref                                   ------------------------------------------------------------  14779
LOC102725072.start-FAM230F.start.18846286-18865042.NEW.ref               ------------------------------------------------------------  17908
chimp.100000-160000.Pan.troglodytes.isolate.Yerkes.chimp.pedigree.#C0471 ccagcctgggtgacagaagaaaccccgtctcaaagaaaatgacagaaaatgcaaatactg  27155
BCRP2.ref.rev.compl                                                      ccagcctgggtgacagaagaaaccccgtctcaaagaaaatgacagaaaatgcaaatactg  17700

exon11.USP18.human                                                       ------------------------------------------------------------  406
LOC102725072.(18846286-18861064new.ref                                   ------------------------------------------------------------  14779
LOC102725072.start-FAM230F.start.18846286-18865042.NEW.ref               ------------------------------------------------------------  17908
chimp.100000-160000.Pan.troglodytes.isolate.Yerkes.chimp.pedigree.#C0471 taagatctagcaatcccactactgggtataactccagagcaaatgaaatcggcaccagag  27215
BCRP2.ref.rev.compl                                                      taagatctagcaatcccactactgggtataactccagagcaaatgaaatcggcaccagag  17760

exon11.USP18.human                                                       ------------------------------------------------------------  406
LOC102725072.(18846286-18861064new.ref                                   ------------------------------------------------------------  14779
LOC102725072.start-FAM230F.start.18846286-18865042.NEW.ref               ------------------------------------------------------------  17908
chimp.100000-160000.Pan.troglodytes.isolate.Yerkes.chimp.pedigree.#C0471 atcagaaagacacaatggggacaggagagtttcttcatgaaattattttgaggaaactga  27275
BCRP2.ref.rev.compl                                                      atcagaaagacacaatggggacaggagagtttcttcatgaaatcattttgaggaaactga  17820

exon11.USP18.human                                                       ------------------------------------------------------------  406
LOC102725072.(18846286-18861064new.ref                                   ------------------------------------------------------------  14779
LOC102725072.start-FAM230F.start.18846286-18865042.NEW.ref               ------------------------------------------------------------  17908
chimp.100000-160000.Pan.troglodytes.isolate.Yerkes.chimp.pedigree.#C0471 tatccatatgcaaaaaagaaaagaaaagaaaagaaagaaatagaacccttctgttacacg  27335
BCRP2.ref.rev.compl                                                      tatccatatgcaaaaaagaaaaga---------aaagaaataggacccttctgttacacg  17871

exon11.USP18.human                                                       ------------------------------------------------------------  406
LOC102725072.(18846286-18861064new.ref                                   ------------------------------------------------------------  14779
LOC102725072.start-FAM230F.start.18846286-18865042.NEW.ref               ------------------------------------------------------------  17908
chimp.100000-160000.Pan.troglodytes.isolate.Yerkes.chimp.pedigree.#C0471 atacacaaaaatcaactcaaaatggattaaagatttcaacacaaaacctgaaaccacaaa  27395
BCRP2.ref.rev.compl                                                      atacacaaaaatcaactcaaaatggattaaagatttcaacacaaaacctgaaaccacaaa  17931

exon11.USP18.human                                                       ------------------------------------------------------------  406
LOC102725072.(18846286-18861064new.ref                                   ------------------------------------------------------------  14779
LOC102725072.start-FAM230F.start.18846286-18865042.NEW.ref               ------------------------------------------------------------  17908
chimp.100000-160000.Pan.troglodytes.isolate.Yerkes.chimp.pedigree.#C0471 acattataaaattcctgtaagaaaagatagggggagcttactttcaggacgacttgaatc  27455
BCRP2.ref.rev.compl                                                      acattattaaattcctgtaagaaaagatagggggagcttactttcaggacaacttgaatc  17991

exon11.USP18.human                                                       ---------------gggggtatttcagtattatacaactgctgtgaccagacttgtata  451
LOC102725072.(18846286-18861064new.ref                                   ------------------------------------------------------------  14779
LOC102725072.start-FAM230F.start.18846286-18865042.NEW.ref               ---------------gggggtacttcagtattatacaactgctgtgaccagacttgtata  17953
chimp.100000-160000.Pan.troglodytes.isolate.Yerkes.chimp.pedigree.#C0471 catgctcatcagttggaaagagtttcaaaaagaaaaagaggaatacacaaggggtaaaaa  27515
BCRP2.ref.rev.compl                                                      catgctcatcagttggaaagagtttcaaaaagaaaaagaggaatacataaggggtaaaaa  18051

exon11.USP18.human                                                       ctggctgaatatcagtgctgtttgtaa-----tttttcactttgagaaccaacattaatt  506
LOC102725072.(18846286-18861064new.ref                                   ------------------------------------------------------------  14779
LOC102725072.start-FAM230F.start.18846286-18865042.NEW.ref               ctggccgaatatcagtgctgtttgtaa-----tttttcactttgagaaccaacattaatt  18008
chimp.100000-160000.Pan.troglodytes.isolate.Yerkes.chimp.pedigree.#C0471 cctcatggagaatggaatggtttgtgaatgatttttttaaattggggaccaaaatcacaa  27575
BCRP2.ref.rev.compl                                                      cctcatggagaatggaatggtttgtgaatgatttttttaaattggggaccaaaatcacaa  18111
```

end of exon 11 in *linc-UR-B1* and end of *linc-UR-B1*

```
exon11.USP18.human                                                       ccatatgaatcaa-----------------------------------------------  519
LOC102725072.(18846286-18861064new.ref                                   ------------------------------------------------------------  14779
LOC102725072.start-FAM230F.start.18846286-18865042.NEW.ref               ccatatgaatcaa-----------------------------------------------  18021
chimp.100000-160000.Pan.troglodytes.isolate.Yerkes.chimp.pedigree.#C0471 gcaaggaaagcaagaacaaccatgtgaggctacaagaaactgaaaagcttctgtgccaca  27635
BCRP2.ref.rev.compl                                                      gcaaggaaagcaagaacaaccatgtgaggctacaagaaactgaaaagcttctgtgccaca  18171

exon11.USP18.human                                                       ------------------------------------------------------------  519
LOC102725072.(18846286-18861064new.ref                                   ------------------------------------------------------------  14779
LOC102725072.start-FAM230F.start.18846286-18865042.NEW.ref               ------------------------------------------------------------  18021
chimp.100000-160000.Pan.troglodytes.isolate.Yerkes.chimp.pedigree.#C0471 acggaaaaaatgagcccaaagtttggacgaaagttttgaggaatcatatatctgctaagg  27695
BCRP2.ref.rev.compl                                                      acggaaaaaatgagcccgaagtttggacgaaagttttgaggaatcatataactgctaagg  18231

exon11.USP18.human                                                       ------------------------------------------------------------  519
LOC102725072.(18846286-18861064new.ref                                   ------------------------------------------------------------  14779
LOC102725072.start-FAM230F.start.18846286-18865042.NEW.ref               ------------------------------------------------------------  18021
chimp.100000-160000.Pan.troglodytes.isolate.Yerkes.chimp.pedigree.#C0471 ggttcttaccgaaaatatacaacacattaacagtactaaatagcaatttaaaacaacaac  27755
BCRP2.ref.rev.compl                                                      ggttcttaccgaaaatatacaacacattaacagtactaaatagcaatttaaaacaacaac  18291

exon11.USP18.human                                                       ------------------------------------------------------------  519
LOC102725072.(18846286-18861064new.ref                                   ------------------------------------------------------------  14779
LOC102725072.start-FAM230F.start.18846286-18865042.NEW.ref               ------------------------------------------------------------  18021
chimp.100000-160000.Pan.troglodytes.isolate.Yerkes.chimp.pedigree.#C0471 aaaaaataatccaccccaaactgggcagaggcccagaacagatatttctgcaaagaggac  27815
BCRP2.ref.rev.compl                                                      aaaaaataatccaccccaaactgggcagaggcccagaacagacatttctgcaaagaggac  18351
```

end of BCRP2 sequence in chimp sequence

```
exon11.USP18.human                                          ------------------------------------------------------------  519
LOC102725072.(18846286-18861064new.ref                      ------------------------------------------------------------  14779
LOC102725072.start-FAM230F.start.18846286-18865042.NEW.ref  ------------------------------------------------------------  18021
chimp.100000-160000.Pan.troglodytes.isolate.Yerkes.chimp.pedigree.#C0471  atagacac----------------------------------------------------  27823
BCRP2.ref.rev.compl                                         aaaaaacccacaccgaggagtccctcgcccctggtcaggcggccacggtgggagggacac  18411

exon11.USP18.human                                          ------------------------------------------------------------  519
LOC102725072.(18846286-18861064new.ref                      ------------------------------------------------------------  14779
LOC102725072.start-FAM230F.start.18846286-18865042.NEW.ref  ------------------------------------------------------------  18021
chimp.100000-160000.Pan.troglodytes.isolate.Yerkes.chimp.pedigree.#C0471  ------------------------------------------------------------  27823
BCRP2.ref.rev.compl                                         gagaggacacgcgctgcccaaaagtgaagaagagtgacccctgggtcctgatggcaggaa  18471

exon11.USP18.human                                          ------------------------------------------------------------  519
LOC102725072.(18846286-18861064new.ref                      ------------------------------------------------------------  14779
LOC102725072.start-FAM230F.start.18846286-18865042.NEW.ref  ------------------------------------------------------------  18021
chimp.100000-160000.Pan.troglodytes.isolate.Yerkes.chimp.pedigree.#C0471  ---------atagatacatagacacagtaacagtctgatctctctttcttttccctacag  27874
BCRP2.ref.rev.compl                                         cgaagatttaaaaatgcagcaaggcccaatgcaaatcctccctctgaggcttggacacga  18531

exon11.USP18.human                                          ------------------------------------------------------------  519
LOC102725072.(18846286-18861064new.ref                      ------------------------------------------------------------  14779
LOC102725072.start-FAM230F.start.18846286-18865042.NEW.ref  ------------------------------------------------------------  18021
chimp.100000-160000.Pan.troglodytes.isolate.Yerkes.chimp.pedigree.#C0471  gcctggaaacaggaactgagacatgccaggaaatc-------------------------  27909
BCRP2.ref.rev.compl                                         ggcagccagccagaactgggcctggccctgactccaacctgagcggggcttccctctctc  18591

exon11.USP18.human                                          -----------------------------------gtgttttgtaactgctattcattta  544
LOC102725072.(18846286-18861064new.ref                      ------------------------------------------------------------  14779
LOC102725072.start-FAM230F.start.18846286-18865042.NEW.ref  -----------------------------------gtgttttgtaactgctattcattta  18046
chimp.100000-160000.Pan.troglodytes.isolate.Yerkes.chimp.pedigree.#C0471  ------------------------------taagatgtatttttctgtgtctctcattct  27940
BCRP2.ref.rev.compl                                         tccctctacacaggggaggttccctcagaaaaagtctttttctgttgttggtttggtttt  18651

exon11.USP18.human                                          ttcagcaaatatttattgatcatctcttctccataagatagtgtgataaacacagtcatg  604
LOC102725072.(18846286-18861064new.ref                      ------------------------------------------------------------  14779
LOC102725072.start-FAM230F.start.18846286-18865042.NEW.ref  ttcagcaaatatttattgatcatctcttctccataagatagtgtgataaacacagtcatg  18106
chimp.100000-160000.Pan.troglodytes.isolate.Yerkes.chimp.pedigree.#C0471  tttttctcctctatgagcacca-cttcccttcgttaactagtttcctgttcacag----g  27995
BCRP2.ref.rev.compl                                         ttttttttttttcagaactaga-aatgtatttatttatttatttattttttaataa----a  18706
```

end of exon11 3'UTR.USP18 sequence and end of *linc-UR-B1* gene

```
exon11.USP18.human                                          aataaagttattttccacaaaa--------------------------------------  626
LOC102725072.(18846286-18861064new.ref                      ------------------------------------------------------------  14779
LOC102725072.start-FAM230F.start.18846286-18865042.NEW.ref  aataaagttattttccacaaaagg--actttgcagttttaacgggggcagtagggattg  18164
chimp.100000-160000.Pan.troglodytes.isolate.Yerkes.chimp.pedigree.#C0471  aaaaataatggtgtcaaca----gctcctctaaatactcaagctgggtcagtaggatccc  28051
BCRP2.ref.rev.compl                                         aaccaagatttttttattattattatactttaaagttttagggtacatgtgcacaa----  18762

exon11.USP18.human                                          ------------------------------------------------------------  626
LOC102725072.(18846286-18861064new.ref                      ------------------------------------------------------------  14779
LOC102725072.start-FAM230F.start.18846286-18865042.NEW.ref  tgctatagaaatt-----caaaggcaaggaagtcacttctgttgtggggccctgggagg  18219
chimp.100000-160000.Pan.troglodytes.isolate.Yerkes.chimp.pedigree.#C0471  ttctcttaaacttaattccaaaatcttgaggaagagctccattggtccagcttgggttgg  28111
BCRP2.ref.rev.compl                                         ------------------------------------------------------------  18762

exon11.USP18.human                                          ------------------------------------------------------------  626
LOC102725072.(18846286-18861064new.ref                      ------------------------------------------------------------  14779
LOC102725072.start-FAM230F.start.18846286-18865042.NEW.ref  agcctac--------------------------------------aggctggaaagggtt  18241
chimp.100000-160000.Pan.troglodytes.isolate.Yerkes.chimp.pedigree.#C0471  accctacccacagtccaatcaacaacatccatgagtggggccatgaggttttcagtggct  28171
BCRP2.ref.rev.compl                                         ------------------------------------------------------------  18762

exon11.USP18.human                                          ------------------------------------------------------------  626
LOC102725072.(18846286-18861064new.ref                      ------------------------------------------------------------  14779
LOC102725072.start-FAM230F.start.18846286-18865042.NEW.ref  aaggtggaggtctccgataggggcagcgtacacagtggactggctgcaaaaggccgtgct  18301
chimp.100000-160000.Pan.troglodytes.isolate.Yerkes.chimp.pedigree.#C0471  tggcttaagatgggctctatctctagtccagtcaacagcagccattggtagggccatgtt  28231
BCRP2.ref.rev.compl                                         ------------------------------------------------------------  18762

exon11.USP18.human                                          ------------------------------------------------------------  626
LOC102725072.(18846286-18861064new.ref                      ------------------------------------------------------------  14779
LOC102725072.start-FAM230F.start.18846286-18865042.NEW.ref  cagcattcagacagcatcacacactccgcttttctctaccagggaggcaggtggggaagg  18361
chimp.100000-160000.Pan.troglodytes.isolate.Yerkes.chimp.pedigree.#C0471  gcacaaatatagtggctcctactatagctatgt---------ggatgcagaaagtaaagg  28282
BCRP2.ref.rev.compl                                         -------------------------------------------catgcaggtttgttaca  18779

exon11.USP18.human                                          ------------------------------------------------------------  626
LOC102725072.(18846286-18861064new.ref                      ------------------------------------------------------------  14779
LOC102725072.start-FAM230F.start.18846286-18865042.NEW.ref  atagcgatgggaaggcaggcggagctcagaatgtggaa----------------------  18399
chimp.100000-160000.Pan.troglodytes.isolate.Yerkes.chimp.pedigree.#C0471  acagtctccagaagacagctgggtatg-----ccaaacaacaggtgacttgctaagagca  28337
BCRP2.ref.rev.compl                                         tatgtatacatgtgccatgttggtgtgctgcacccatcaactcgtcatttagcatcaggt  18839

exon11.USP18.human                                          ------------------------------------------------------------  626
LOC102725072.(18846286-18861064new.ref                      ------------------------------------------------------------  14779
LOC102725072.start-FAM230F.start.18846286-18865042.NEW.ref  ------------------------------------------------------------  18399
chimp.100000-160000.Pan.troglodytes.isolate.Yerkes.chimp.pedigree.#C0471  atgagaaataatacctgccctcaaggagcttatagtttcacgagggattcagacccagag  28397
BCRP2.ref.rev.compl                                         atatctcctaatgctatccctc--------------------------------------  18861

exon11.USP18.human                                          ------------------------------------------------------------  626
LOC102725072.(18846286-18861064new.ref                      ------------------------------------------------------------  14779
LOC102725072.start-FAM230F.start.18846286-18865042.NEW.ref  ------------------------------------------------------------  18399
chimp.100000-160000.Pan.troglodytes.isolate.Yerkes.chimp.pedigree.#C0471  atacacaaataccattcctactcagaagaggctactacattgatatgactcagagacatc  28457
BCRP2.ref.rev.compl                                         -----------------------------------------------ccccctcccccca  18875

exon11.USP18.human                                          ------------------------------------------------------------  626
LOC102725072.(18846286-18861064new.ref                      ------------------------------------------------------------  14779
LOC102725072.start-FAM230F.start.18846286-18865042.NEW.ref  ------------------------------------------------------------  18399
chimp.100000-160000.Pan.troglodytes.isolate.Yerkes.chimp.pedigree.#C0471  cacataaccagagtttgatgacagaagttaccagaagttgcaccaactgctgccccaaag  28517
BCRP2.ref.rev.compl                                         cccagaaaaagtcttaaacccctaaaaataccacagtctgctacacttgacaccctcaaa  18935

exon11.USP18.human                                          ------------------------------------------------------------  626
LOC102725072.(18846286-18861064new.ref                      ------------------------------------------------------------  14779
LOC102725072.start-FAM230F.start.18846286-18865042.NEW.ref  ------------------------------------------------------------  18399
chimp.100000-160000.Pan.troglodytes.isolate.Yerkes.chimp.pedigree.#C0471  ccatcctaggacctgatgagctaattagtcacaattattattgatgacagc---------  28568
BCRP2.ref.rev.compl                                         agccttcagagaatgaataccactgtaaaataaatactaaaacactacaagagaaagagg  18995

exon11.USP18.human                                          ------------------------------------------------------------  626
LOC102725072.(18846286-18861064new.ref                      ------------------------------------------------------------  14779
LOC102725072.start-FAM230F.start.18846286-18865042.NEW.ref  ------------------------------------------------------------  18399
chimp.100000-160000.Pan.troglodytes.isolate.Yerkes.chimp.pedigree.#C0471  ---------------------------------------------accaatccacctgtt  28583
BCRP2.ref.rev.compl                                         aaaaccagggagaacaggagaggaatgaacagaacataccagaaaactaaagcttctgta  19055

exon11.USP18.human                                          ------------------------------------------------------------  626
LOC102725072.(18846286-18861064new.ref                      ------------------------------------------------------------  14779
LOC102725072.start-FAM230F.start.18846286-18865042.NEW.ref  ------------------------------------------------------------  18399
chimp.100000-160000.Pan.troglodytes.isolate.Yerkes.chimp.pedigree.#C0471  tgtgtcacaagacagttattgctgggtgaggtggctcacacctgtaatcccagcattttg  28643
BCRP2.ref.rev.compl                                         ctcaccatgcaggaaaaatgccagggaaaagtgcatggcagcttcctcctcagcact---  19112

exon11.USP18.human                                          ------------------------------------------------------------  626
LOC102725072.(18846286-18861064new.ref                      ------------------------------------------------------------  14779
LOC102725072.start-FAM230F.start.18846286-18865042.NEW.ref  --------------ggggatccagtaaggcttggaagtttgcacctgatctggtgggtgg  18445
chimp.100000-160000.Pan.troglodytes.isolate.Yerkes.chimp.pedigree.#C0471  gaaggccaaggtgggaggatcacttgagcccaggaggtcaagaccagcctgggcaacata  28703
BCRP2.ref.rev.compl                                         ------------------------------------------------------------  19112

exon11.USP18.human                                          ------------------------------------------------------------  626
LOC102725072.(18846286-18861064new.ref                      ------------------------------------------------------------  14779
LOC102725072.start-FAM230F.start.18846286-18865042.NEW.ref  tgaggagcccttgaaggggcaaggaggtgagaagcactcagctgtgttcttacactgatc  18505
chimp.100000-160000.Pan.troglodytes.isolate.Yerkes.chimp.pedigree.#C0471  atgagagcccatttctactaaaaataggaactaaaaaattaaccaggcatggctgtggtg  28763
BCRP2.ref.rev.compl                                         -------------------------------------------cagggcccagacaggag  19129

exon11.USP18.human                                          ------------------------------------------------------------  626
LOC102725072.(18846286-18861064new.ref                      ------------------------------------------------------------  14779
```

```
LOC102725072.start-FAM230F.start.18846286-18865042.NEW.ref             tgccactggggttagagacaaacgtggtgggaatggaaagccacgcacagtcactagcgc  18565
chimp.100000-160000.Pan.troglodytes.isolate.Yerkes.chimp.pedigree.#C0471      tgcctgtgctcccagcccacgaggtcaaggctgcggtgagccacgttca-----------  28812
BCRP2.ref.rev.compl                                                    cggttttctccccaggacacagctccaagtcgtccagaagcctccgtga-----------  19178

exon11.USP18.human                                                     ------------------------------------------------------------  626
LOC102725072.(18846286-18861064new.ref                                 ------------------------------------------------------------  14779
LOC102725072.start-FAM230F.start.18846286-18865042.NEW.ref             ctctgggggagaatggatgtggctggtgagagaacagggggcccagggagagtccggc  18625
chimp.100000-160000.Pan.troglodytes.isolate.Yerkes.chimp.pedigree.#C0471      ------------------------------------------------------------  28812
BCRP2.ref.rev.compl                                                    ------------------------------------------------------------  19178

exon11.USP18.human                                                     ------------------------------------------------------------  626
LOC102725072.(18846286-18861064new.ref                                 ------------------------------------------------------------  14779
LOC102725072.start-FAM230F.start.18846286-18865042.NEW.ref             accaacctggcgggggagcccagtgggtgtgagcacccccactttagagatgaagtgat  18685
chimp.100000-160000.Pan.troglodytes.isolate.Yerkes.chimp.pedigree.#C0471      --------------------------cgtcactgcactccagcctgggcgacagagcaag  28846
BCRP2.ref.rev.compl                                                    --------------------------tgcccctcctggcgggtcctgagggtatcagagc  19212

exon11.USP18.human                                                     ------------------------------------------------------------  626
LOC102725072.(18846286-18861064new.ref                                 ------------------------------------------------------------  14779
LOC102725072.start-FAM230F.start.18846286-18865042.NEW.ref             ggagacattcagatgtttaaccccttgttcaagattccatacttgataaatggcagatca  18745
chimp.100000-160000.Pan.troglodytes.isolate.Yerkes.chimp.pedigree.#C0471      accttgtctcaaaaaaaaaaaaggatgctcaaaactaataaattgaaacattacaggagg  28906
BCRP2.ref.rev.compl                                                    tcctctcctc--------ctcacggtggatgccgccactcactccatgcagcacctgcgg  19264

exon11.USP18.human                                                     ------------------------------------------------------------  626
LOC102725072.(18846286-18861064new.ref                                 ------------------------------------------------------------  14779
LOC102725072.start-FAM230F.start.18846286-18865042.NEW.ref             aactcccaacat------------------------------------------------  18757
chimp.100000-160000.Pan.troglodytes.isolate.Yerkes.chimp.pedigree.#C0471      gtatgccatgaaaagtgaaagcctccttcctcctagttactataaatattttgcttgttt  28966
BCRP2.ref.rev.compl                                                    ggagaac-----------------------------------------------------  19271

exon11.USP18.human                                                     --------------------------------     626
LOC102725072.(18846286-18861064new.ref                                 --------------------------------     14779
LOC102725072.start-FAM230F.start.18846286-18865042.NEW.ref             --------------------------------     18757
chimp.100000-160000.Pan.troglodytes.isolate.Yerkes.chimp.pedigree.#C0471      tttattttaaggtatagtctagactttttcca     28998
BCRP2.ref.rev.compl                                                    --------------------------------     19271
```

.
