# Supplementary material for: Birth of a Regulatory Long Non-coding RNA/Gene, linc-UR-UB
Source: Front Genet. 2021 Apr 30;12:661425. doi: 10.3389/fgene.2021.661425 (PMC8120154; doi:10.3389/fgene.2021.661425)
Supplement: Supplementary file 3 [file Data_Sheet_3.PDF]

Birth of a regulatory long non-coding RNA gene, *linc-UR-UB*

Nicholas Delihas

Department of Microbiology and Immunology
Renaissance School of Medicine
Stony Brook University
Stony Brook, New York, 11794-5222

Email: Nicholas.delihas@stonybook.edu

Supplementary Figures S3.a-c. Gene/sequence motifs present in non-coding regions of chr 20, chr 13, and chr 22.

Supplementary Figure S3.a

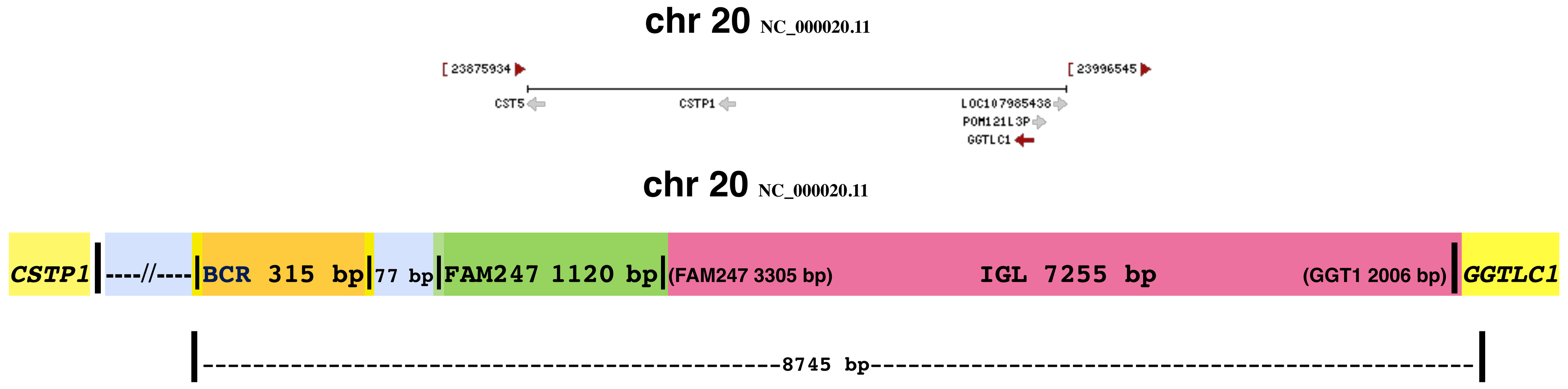

Supplementary Figure S3.a. The top schematic represents a segment of chr 20 and is taken from the NCBI website: https://www.ncbi.nlm.nih.gov/gene/92086 (O'Leary, et al, 2016). The diagram below shows the motifs in the non-coding region between genes *CSTP1 and GGTLC1*. The region between genes *CSTP1 and GGTLC1* contains no gene annotations and is at chr 20 coordinates 23922170-23985053 and consists of 62883 bp. The region of 8745 bp, the sequence to the left of gene *GGTLC1* is shown in detail. The IGL sequence **IGL-7255bp** is homologous to part of a segment of **Homo sapiens chromosome 22, GRCh38.p13 Primary Assembly.** NCBI Reference Sequence: NC_000022.11.896838 bp. The **IGL-7255bp** sequence is homologous to positions **621829 to 629042 of the IGL locus.** The numbers (e.g., **IGL 7255bp**)show the bp segments of the non-coding region of *CSTP1-GGTLC1* that contain sections of a gene/sequence, in this case the IGL locus. *BCR* is the BCR activator of RhoGEF and GTPase and *FAM247* represents the long intergenic non-coding RNA gene *FAM247A*. Part of the region of FAM247 shown in the drawing is in the IGL locus of chr22 as the *CSTP1-GGTLC1* region appears to contain a copy of the IGL locus, which has part of the FAM247 sequence. The chromosomal coordinates of the chr20 non-coding region are 23922170-23985052. The region designated |---//---|, which consists of 54154bp shows no detectable gene/sequence motifs. The *CSTP1-GGTLC1* region carries a copy of the 3' end of GGT1, 50251-52255 (2006 bp) and a copy of the FAM247, positions 1-4425 . The BCR (315 bp) is a copy of the 3' end sequence of the BCR activator of RhoGEF and GTPase gene.

Supplementary Figure S3.b

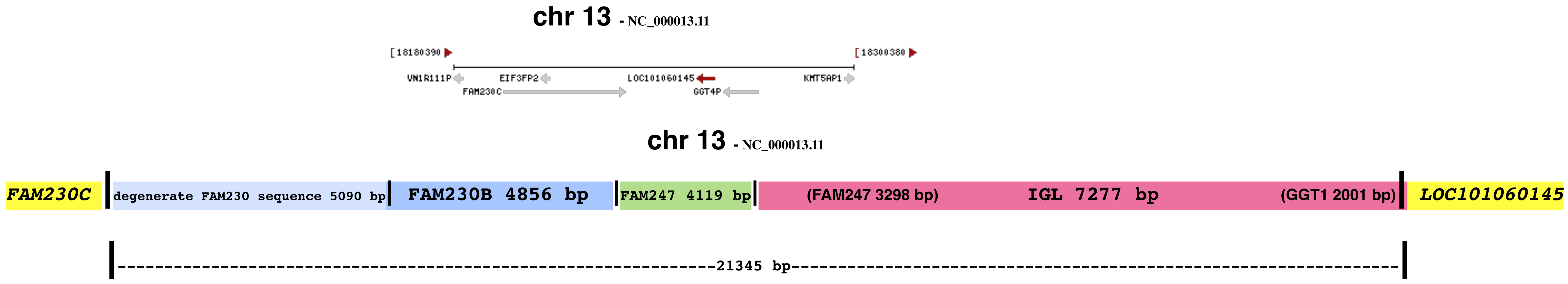

Supplementary Figure S3.b. The top schematic is from the NCBI ((O'Leary, et al, 2016). The bottom drawing shows the gene/sequence motifs that are present in the non-coding region between genes *FAM230C* and *LOC101060145.*
Positions 1-5090 in the non-coding region display a 62% identity with *FAM230A* and 65% with *FAM230B* and thus appears to be a degenerate sequence. The FAM247 sequence (4119 bp) is contiguous with the IGL sequence copy that itself has a portion of the FAM247. **(FAM247 3298 bp)** represents the total number positions of the IGL copy within the *FAM230C-LOC101060145* devoted to the FAM247 sequence bp positions, not the total number of FAM247 positions due to insertions/deletions between sequences. The total sequence of the FAM247 present in the *FAM230C-LOC101060145* region are the positions 1-7417 bp. FAM230B gene positions 19374 to 24299 bp are in *FAM230C-LOC101060145.* The *FAM230C-LOC101060145* carries the homologous sequence to the IGL Locus positions **621843 to 629047 f**rom: **Homo sapiens chromosome 22, GRCh38.p13 Primary Assembly.** NCBI Reference Sequence: NC_000022.11.896838 bp. This IGL sequence does not overlap with the *BCRP3* gene sequence that contains the **IGL 590380-594292.** This a similar copy of the IGL locus that is present in the non-coding region of chr20. The GGT1 sequence carried by the IGL copy consists of the 3' end positions 50264-52264 bp of the *GGT1* gene. The IGL locus can present difficulties in determining homologous sequences due to the large number of repeats. In addition, precise sequence/gene start/stop sites are difficult to determine by sequence alignments in cases where there is imperfect alignment.

Supplementary Figure S3.c

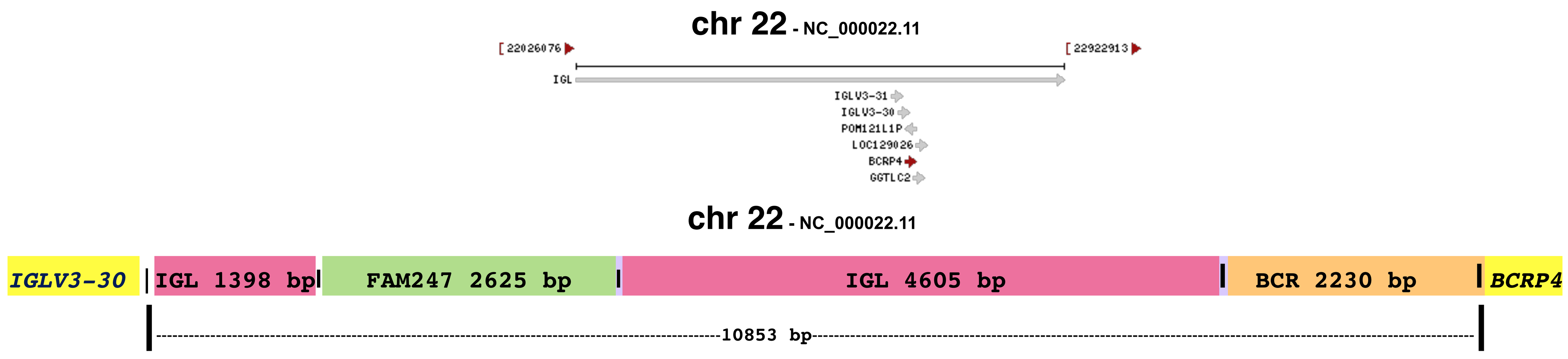

Supplementary Figure S3.c. The IGL locus sequence positions represents the 10853bp non-coding region and are at IGL gene positions 593137 - 603989bp from Homo sapiens chromosome 22, GRCh38.p13 Primary Assembly NCBI Reference Sequence: NC_000022.11, LOCUS NC_000022, 896838 bp . Part of the FAM247A gene, positions 3327-5958bp and part of the *BCR* gene, positions 126343-128915 bp of the gene are present in the non-coding region *IGLV3-30-BCRP4*.
